# Supplementary material for: The Evolution and Characterization of the RNA Interference Pathways in Lophotrochozoa
Source: Genome Biol Evol. 2024 May 7;16(5):evae098. doi: 10.1093/gbe/evae098 (PMC11114477; doi:10.1093/gbe/evae098)
Supplement: evae098_Supplementary_Data [file evae098_supplementary_data.zip › Supplementary_materials.zip!/selected_miRNAs/NoGe_selected_miRNAs/mirdeep_pdf_files/NMRB01000765.1_Notospermus_geniculatus_strain_Ushimado_scaffold765,_whole_genome_shotgun_sequence_20624.pdf]

Provisional ID	: NMRB01000765.1_Notospermus_geniculatus_strain_Ushimado_scaffold765,_whole_genome_shotgun_sequence_20624
Score total	: 4960496.9
Score for star read(s)	: 3.9
Score for read counts	: 4960488.5
Score for mfe	: 1.5
Score for randfold	: 0
Score for cons. seed	: 3
Total read count	: 9729796
Mature read count	: 9611509
Loop read count	: 0
Star read count	: 118287

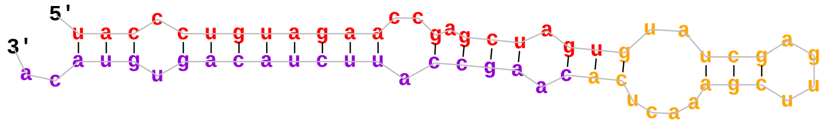

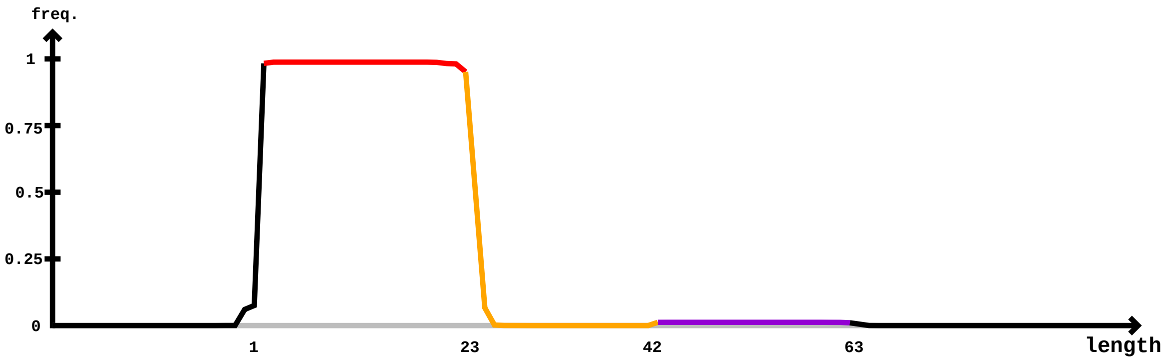

Mature Star

5'- aaauguggugaguuucugugguacccuguagaaccgagcuaguguaucgaguucgaaacucacaagccauucuacaguguacaaagaaacgucgcuucuuucaacaaugaga -3'	obs		
aaauguggugaguuucugugguacccuguagaaccgagcuaguguaucgaguucgaaacucacaagccauucuacaguguacaaagaaacgucgcuucuuucaacaaugaga	exp		
....(.(((((((((((.(((((.((((((((..(.(((.(((..(((....)))....))).)))).)))))))).))))).)))))).))))).)(((((....)))))	reads	mm	sample
...............cuguguacccuguagaaccgagcu..	3	0	Mm1
................uguguacccuguagaaAcgagc...	1	1	Mm1
................uguguacccuguagaaccgagc...	6	0	Mm1
................uguguacccuguagaacGgagcu..	2	1	Mm1
................uguguacccuguagaaccgagcu..	78	0	Mm1
................uguguacccuguagaaGcgagcu..	1	1	Mm1
................uguguacccuguGgaaccgagcu..	4	1	Mm1
................Nguguacccuguagaaccgagcu..	1	1	Mm1
................uguguacccuguagaaAcgagcu..	1	1	Mm1
.................guguacccuguagaaccgagcu..	1	0	Mm1
..................uguacccuguagaaccgagc...	172	0	Mm1
..................uguacccuguagaaccgagA...	4	1	Mm1
..................uguacccuguagaaccgUgc...	1	1	Mm1
..................uguacccuguagaaccAagc...	1	1	Mm1
..................uguacccuguagaaccgaUc...	1	1	Mm1
..................uguacccuguagaaccgCgc...	1	1	Mm1
..................Aguacccuguagaaccgagc...	1	1	Mm1
..................uguacccuguagaaccgagcu..	660	0	Mm1
..................ugCacccuguagaaccgagcu..	1	1	Mm1
..................uguacccCguagaaccgagcu..	1	1	Mm1
..................uguacccuguagaaccgGgcu..	1	1	Mm1
..................uguacccuguGgaaccgagcu..	1	1	Mm1
..................uguacccuguagaaccgCgcu..	3	1	Mm1
..................uguacccuguagaaGcgagcu..	1	1	Mm1
..................ugAacccuguagaaccgagcu..	1	1	Mm1
..................uguacccuguagaaccCagcu..	3	1	Mm1
..................uguacccuguagaaccgaAcu..	1	1	Mm1
..................uguacUcuguagaaccgagcu..	2	1	Mm1
..................uguacccuguagaaccUagcu..	2	1	Mm1
..................uguacccuguagaaccgUgcu..	1	1	Mm1
..................uguacccuguagaaccgUgcua...	1	1	Mm1
..................uguacccuguagaaccgagcua...	104	0	Mm1
..................uguacccuguagaaccgagcuU...	22	1	Mm1

```
                    Mature                    Star
aaauguggugaguuucugugguacccuguagaaccgagcuaguguaucgaguucgaaacucacaagccauucuacaguguacaaagaaacgucgcuucuuucaacaaugaga
..................uguacccuguagaaccgCgcua.......................................................................  2     1  Mm1
..................uguacccuguagaaccAagcua.......................................................................  1     1  Mm1
..................uguacccuguagaaccgagcuGg......................................................................  9     1  Mm1
..................uguacccugCagaaccgagcuag......................................................................  1     1  Mm1
..................uguacccuguagaaccUagcuag......................................................................  6     1  Mm1
..................uguacccuguagaaccCagcuag......................................................................  3     1  Mm1
..................uguacccuguCgaaccgagcuag......................................................................  3     1  Mm1
..................uguacccuguagaaccgagcuaU......................................................................  129   1  Mm1
..................uguacccuguUgaaccgagcuag......................................................................  1     1  Mm1
..................uguacccuguaUaaccgagcuag......................................................................  1     1  Mm1
..................uguacccuAuagaaccgagcuag......................................................................  1     1  Mm1
..................uguacGcuguagaaccgagcuag......................................................................  1     1  Mm1
..................uguaUccuguagaaccgagcuag......................................................................  1     1  Mm1
..................uguacccuguGgaaccgagcuag......................................................................  6     1  Mm1
..................uguacccuguagaaccgGgcuag......................................................................  14    1  Mm1
..................ugAacccuguagaaccgagcuag......................................................................  1     1  Mm1
..................uguacUcuguagaaccgagcuag......................................................................  1     1  Mm1
..................uguacAcuguagaaccgagcuag......................................................................  1     1  Mm1
..................Aguacccuguagaaccgagcuag......................................................................  1     1  Mm1
..................uguacccuguagaaccAagcuag......................................................................  7     1  Mm1
..................uguacccuguagaaccgagcuag......................................................................  2682  0  Mm1
..................uguacccAguagaaccgagcuag......................................................................  1     1  Mm1
..................uguacccuguagaaccgCgcuag......................................................................  9     1  Mm1
..................uguacccuguagaaccgUgcuag......................................................................  9     1  Mm1
..................uguacccuguagaaccgaUcuag......................................................................  2     1  Mm1
..................ugGacccuguagaaccgagcuag......................................................................  1     1  Mm1
..................uguacccuguagaaccgagcuaA......................................................................  16    1  Mm1
..................uguaccAuguagaaccgagcuag......................................................................  1     1  Mm1
..................Nguacccuguagaaccgagcuag......................................................................  2     1  Mm1
..................uguacccuguagaaccgagcGag......................................................................  1     1  Mm1
..................uguUcccuguagaaccgagcuagu.....................................................................  2     1  Mm1
..................ugCacccuguagaaccgagcuagu.....................................................................  2     1  Mm1
..................uguacccuguagaUccgagcuagu.....................................................................  2     1  Mm1
..................uguacUcuguagaaccgagcuagu.....................................................................  23    1  Mm1
..................uguaAccuguagaaccgagcuagu.....................................................................  7     1  Mm1
..................uguacccuguUgaaccgagcuagu.....................................................................  16    1  Mm1
..................uguacccuguagaaccgagcAagu.....................................................................  1     1  Mm1
..................uguacccugCagaaccgagcuagu.....................................................................  2     1  Mm1
..................uguacccugAagaaccgagcuagu.....................................................................  4     1  Mm1
..................uguacccuUuagaaccgagcuagu.....................................................................  4     1  Mm1
..................uCuacccuguagaaccgagcuagu.....................................................................  3     1  Mm1
..................uguacccuguagaaccgaAcuagu.....................................................................  2     1  Mm1
..................uguacccAguagaaccgagcuagu.....................................................................  4     1  Mm1
..................uguacccuguGgaaccgagcuagu.....................................................................  43    1  Mm1
..................uguacccuguagaaccgUgcuagu.....................................................................  84    1  Mm1
..................Cguacccuguagaaccgagcuagu.....................................................................  2     1  Mm1
..................uguacccuguagaaccgagcuGgu.....................................................................  23    1  Mm1
..................uguaGccuguagaaccgagcuagu.....................................................................  2     1  Mm1
..................uguacccuguaAaaccgagcuagu.....................................................................  1     1  Mm1
..................uguacccuguCgaaccgagcuagu.....................................................................  29    1  Mm1
..................uguacccuguagaaccgagcuaAu.....................................................................  7     1  Mm1
..................uguacccuguagaaccgagcCagu.....................................................................  4     1  Mm1
..................uAuacccuguagaaccgagcuagu.....................................................................  17    1  Mm1
..................uguacccuguagaaccUagcuagu.....................................................................  57    1  Mm1
..................uguacccuguagaaccgagUuagu.....................................................................  2     1  Mm1
..................uguacccuguagaaccgagcuagC.....................................................................  1     1  Mm1
..................uguacGcuguagaaccgagcuagu.....................................................................  3     1  Mm1
..................uguacAcuguagaaccgagcuagu.....................................................................  2     1  Mm1
..................uguacccuguagaaccgagcuaUu.....................................................................  41    1  Mm1
..................uguGcccuguagaaccgagcuagu.....................................................................  1     1  Mm1
..................uguacccuguagUaccgagcuagu.....................................................................  2     1  Mm1
..................Gguacccuguagaaccgagcuagu.....................................................................  2     1  Mm1
..................ugAacccuguagaaccgagcuagu.....................................................................  6     1  Mm1
..................Nguacccuguagaaccgagcuagu.....................................................................  14    1  Mm1
..................uguaccGuguagaaccgagcuagu.....................................................................  2     1  Mm1
..................uguacccuAuagaaccgagcuagu.....................................................................  2     1  Mm1
..................uguacccuguagaacUgagcuagu.....................................................................  7     1  Mm1
..................uguacccuguagaaccgGgcuagu.....................................................................  69    1  Mm1
..................uguacccuguagCaccgagcuagu.....................................................................  1     1  Mm1
..................uguacccuguagaaccgagcuagA.....................................................................  67    1  Mm1
```

Mature Star

```
aaauguggugaguuucugugUACCCUGUAGAACCGAGCUAGUguaucgaguucgaaacucacaagccauucuacaguguacaaagaaacgucgcuucuuucaacaaugaga
```

```
aaauguggugaguuucuguguacccuguagaaccgagcuaguguaucgaguucgaaacucacaagccauucuacaguguacaaagaaacgucgcuucuuucaacaaugaga
..................uguacccuguagaaccgaUcuagu.....................................................................    5      1   Mm1
..................uguacccuguagaaccgagcGagu.....................................................................    2      1   Mm1
..................uguacccuguagaGccgagcuagu.....................................................................    7      1   Mm1
..................uguacccuguagaaccgagcuagu.....................................................................    27916  0   Mm1
..................ugGacccuguagaaccgagcuagu.....................................................................    6      1   Mm1
..................uguacccuguagaCccgagcuagu.....................................................................    2      1   Mm1
..................uguaccNuguagaaccgagcuagu.....................................................................    2      1   Mm1
..................uguacccuguagaacAgagcuagu.....................................................................    2      1   Mm1
..................uguaccAuguagaaccgagcuagu.....................................................................    4      1   Mm1
..................uguacccuguagaaUcgagcuagu.....................................................................    1      1   Mm1
..................uguacccuguagaaccgagcuagG.....................................................................    20     1   Mm1
..................uguacccuguagaaccgagcuUgu.....................................................................    2      1   Mm1
..................uguacccuguagaaccCagcuagu.....................................................................    66     1   Mm1
..................uguacccuguagGaccgagcuagu.....................................................................    3      1   Mm1
..................Aguacccuguagaaccgagcuagu.....................................................................    2      1   Mm1
..................uguacccuguagaaccgCgcuagu.....................................................................    113    1   Mm1
..................uguacNcuguagaaccgagcuagu.....................................................................    1      1   Mm1
..................uguacccCguagaaccgagcuagu.....................................................................    3      1   Mm1
..................uguacccuCuagaaccgagcuagu.....................................................................    2      1   Mm1
..................uguaUccuguagaaccgagcuagu.....................................................................    3      1   Mm1
..................uguacccuguagaaccAagcuagu.....................................................................    56     1   Mm1
..................uguacccuguagaaccgagAuagu.....................................................................    3      1   Mm1
..................uguacccuguaCaaccgagcuagu.....................................................................    3      1   Mm1
..................uUuacccuguagaaccgagcuagu.....................................................................    6      1   Mm1
..................uguaccUuguagaaccgagcuagu.....................................................................    2      1   Mm1
..................uguacccuguaUaaccgagcuagu.....................................................................    4      1   Mm1
..................uguacccugGagaaccgagcuagu.....................................................................    1      1   Mm1
..................uguacccuguagaaccUagcuagug....................................................................    1      1   Mm1
..................uguacccuguagaaccCagcuagug....................................................................    1      1   Mm1
..................uguacccuguagaaccgUgcuagug....................................................................    3      1   Mm1
..................Cguacccuguagaaccgagcuagug....................................................................    1      1   Mm1
..................uguacccuguagaaccgagcuaguA....................................................................    34     1   Mm1
..................uguacccuguagaaccgCgcuagug....................................................................    1      1   Mm1
..................uguacccuguagaaccgagcuaguC....................................................................    1      1   Mm1
..................uguacccuguagaaccgagcuaguU....................................................................    1144   1   Mm1
..................uguacUcuguagaaccgagcuagug....................................................................    1      1   Mm1
..................uguacccuguagaaccgGgcuagug....................................................................    1      1   Mm1
..................uguacccuguagaaccgagcuagug....................................................................    392    0   Mm1
..................uguacccuguGgaaccgagcuagug....................................................................    3      1   Mm1
..................uCuacccuguagaaccgagcuagugu...................................................................    7      1   Mm1
..................Nguacccuguagaaccgagcuagugu...................................................................    1      1   Mm1
..................uguacccuguagaaccgagcuUgugu...................................................................    1      1   Mm1
..................Cguacccuguagaaccgagcuagugu...................................................................    1      1   Mm1
..................uguacccuguagaaccgagcuagugA...................................................................    11     1   Mm1
..................uguacccuguagaaccgagcuaguUu...................................................................    77     1   Mm1
..................uguacccCguagaaccgagcuagugu...................................................................    1      1   Mm1
..................ugAacccuguagaaccgagcuagugu...................................................................    1      1   Mm1
..................uguacccuguagaaccgagcuaguCu...................................................................    5      1   Mm1
..................uguacccuguagaaccUagcuagugu...................................................................    1      1   Mm1
..................uguacccuguagaaccgagcuaguAu...................................................................    2      1   Mm1
..................uguacUcuguagaaccgagcuagugu...................................................................    1      1   Mm1
..................uguacccuguagCaccgagcuagugu...................................................................    1      1   Mm1
..................uguacccuguagaaccgagcuagugC...................................................................    1      1   Mm1
..................uguacccuguGgaaccgagcuagugu...................................................................    1      1   Mm1
..................uUuacccuguagaaccgagcuagugu...................................................................    1      1   Mm1
..................uguacccuguagaaccgCgcuagugu...................................................................    2      1   Mm1
..................uguacccuguagaaccAagcuagugu...................................................................    1      1   Mm1
..................uguacccuguagaaccgUgcuagugu...................................................................    1      1   Mm1
..................uguacccuguagaaccCagcuagugu...................................................................    1      1   Mm1
..................uguacccuguagaaccgagcuGgugu...................................................................    1      1   Mm1
..................uguacccuguagaaccgGgcuagugu...................................................................    1      1   Mm1
..................ugCacccuguagaaccgagcuagugu...................................................................    1      1   Mm1
..................uguacccuguagaaccgagcuagugG...................................................................    13     1   Mm1
..................uguacccuguagaaccgagcuagugu...................................................................    744    0   Mm1
..................uguacccuguagaaccgagcuaguguU..................................................................    28     1   Mm1
..................uguacccuguagaaccgagcuaguguauc................................................................    3      0   Mm1
..................uguacccuguagaaccgagcuaguguauU................................................................    1      1   Mm1
..................uguacccuguagaaccgagcuaguguaucgaguu...........................................................    4      0   Mm1
...................Uuacccuguagaaccgagc.........................................................................    1      1   Mm1
...................guacccuguagaaccgagc.........................................................................    9      0   Mm1
```

Mature **Star**

```
aaauguggugaguuucuguguacccuguagaaccgagcuaguguaucgaguucgaaacucacaagccauucuacaguguacaaagaaacgucgcuucuuucaacaaugaga
...................guaAccuguagaaccgagcu........................................................................   1      1   Mm1
...................guacccuguagaaccgaAcu........................................................................   2      1   Mm1
...................guacccuguagaaccgaUcu........................................................................   1      1   Mm1
...................guacccuguagaaccgUgcu........................................................................   1      1   Mm1
...................Nuacccuguagaaccgagcu........................................................................   1      1   Mm1
...................guacccuguagaaccgagcu........................................................................   295    0   Mm1
...................Auacccuguagaaccgagcu........................................................................   83     1   Mm1
...................guacccuguagaaccgaCcu........................................................................   1      1   Mm1
...................guacccuguagaaccgGgcu........................................................................   1      1   Mm1
...................guacccuguagaaccgagcGa.......................................................................   2      1   Mm1
...................guacccuguagaaccgagcuU.......................................................................   4      1   Mm1
...................Auacccuguagaaccgagcua.......................................................................   2      1   Mm1
...................gCacccuguagaaccgagcuag......................................................................   13     1   Mm1
...................guacccuguagaaccgagcuaU......................................................................   5      1   Mm1
...................guacccuguagaaccgagAuag......................................................................   1      1   Mm1
...................guacccuguagaaAcgagcuag......................................................................   1      1   Mm1
...................guacccuguagaaccgaUcuag......................................................................   1      1   Mm1
...................guacccuguagaaccgCgcuag......................................................................   1      1   Mm1
...................guacccuguagaaccUagcuag......................................................................   1      1   Mm1
...................guacccuguagaaccgUgcuag......................................................................   2      1   Mm1
...................guacccuguagaaccgagcGag......................................................................   14     1   Mm1
...................Auacccuguagaaccgagcuag......................................................................   1      1   Mm1
...................guacAcuguagaaccgagcuag......................................................................   1      1   Mm1
...................Cuacccuguagaaccgagcuag......................................................................   3      1   Mm1
...................guacccuguagaaccgaAcuag......................................................................   2      1   Mm1
...................Uuacccuguagaaccgagcuag......................................................................   1      1   Mm1
...................guacccuguaAaaccgagcuag......................................................................   2      1   Mm1
...................guacccuguagaaccgagcuag......................................................................   313    0   Mm1
...................guacccuguagaaccgagcuagG.....................................................................   5      1   Mm1
...................guUcccuguagaaccgagcuagu.....................................................................   3      1   Mm1
...................guacccuguagaaccgagAuagu.....................................................................   1      1   Mm1
...................Nuacccuguagaaccgagcuagu.....................................................................   3      1   Mm1
...................guacccuguaCaaccgagcuagu.....................................................................   8      1   Mm1
...................guacccuguagaacGgagcuagu.....................................................................   2      1   Mm1
...................guaccAuguagaaccgagcuagu.....................................................................   1      1   Mm1
...................guacccuguagaaccgaCcuagu.....................................................................   43     1   Mm1
...................guacccCguagaaccgagcuagu.....................................................................   1      1   Mm1
...................guacccuguagaaccgaUcuagu.....................................................................   38     1   Mm1
...................gAacccuguagaaccgagcuagu.....................................................................   4      1   Mm1
...................Uuacccuguagaaccgagcuagu.....................................................................   11     1   Mm1
...................guGcccuguagaaccgagcuagu.....................................................................   2      1   Mm1
...................guacccuguagaaccgagUuagu.....................................................................   2      1   Mm1
...................guacccuguagaaccgCgcuagu.....................................................................   24     1   Mm1
...................guacccuguaAaaccgagcuagu.....................................................................   20     1   Mm1
...................guacccugGagaaccgagcuagu.....................................................................   1      1   Mm1
...................guCcccuguagaaccgagcuagu.....................................................................   1      1   Mm1
...................guacccugAagaaccgagcuagu.....................................................................   1      1   Mm1
...................guacccuguagaaAcgagcuagu.....................................................................   3      1   Mm1
...................guacccGguagaaccgagcuagu.....................................................................   1      1   Mm1
...................guaccUuguagaaccgagcuagu.....................................................................   2      1   Mm1
...................gCacccuguagaaccgagcuagu.....................................................................   1      1   Mm1
...................guacUcuguagaaccgagcuagu.....................................................................   2      1   Mm1
...................guacccuguagaaccgagcuagC.....................................................................   1      1   Mm1
...................guacccuguagaaccgagcuagu.....................................................................   8784   0   Mm1
...................Auacccuguagaaccgagcuagu.....................................................................   96     1   Mm1
...................guaAccuguagaaccgagcuagu.....................................................................   3      1   Mm1
...................guacccuguagaaccgagcuaCu.....................................................................   1      1   Mm1
...................guacccuguagaaccgagcuGgu.....................................................................   16     1   Mm1
...................guacccuguUgaaccgagcuagu.....................................................................   1      1   Mm1
...................guacccuguaUaaccgagcuagu.....................................................................   5      1   Mm1
...................guacccuguagaaGcgagcuagu.....................................................................   3      1   Mm1
...................guaGccuguagaaccgagcuagu.....................................................................   3      1   Mm1
...................guacccuguagaaccUagcuagu.....................................................................   2      1   Mm1
...................guacccuguagaacUgagcuagu.....................................................................   11     1   Mm1
...................guacccuguagUaccgagcuagu.....................................................................   1      1   Mm1
...................guacccuguagaaccgUgcuagu.....................................................................   13     1   Mm1
...................guacccuguGgaaccgagcuagu.....................................................................   6      1   Mm1
...................guacccugCagaaccgagcuagu.....................................................................   1      1   Mm1
...................guaccNuguagaaccgagcuagu.....................................................................   4      1   Mm1
...................guacGcuguagaaccgagcuagu.....................................................................   2      1   Mm1
```

```
                   Mature                      Star
aaauguggugaguuucuguguacccuguagaaccgagcuaguguaucgaguucgaaacucacaagccauucuacaguguacaaagaaacgucgcuucuuucaacaaugaga
...................guacccuguagaaUcgagcuagu.....................................................................   2     1   Mm1
...................Cuacccuguagaaccgagcuagu.....................................................................   139   1   Mm1
...................guacccuguagaaccgagcuaAu.....................................................................   9     1   Mm1
...................guacccuguagaaccgagcuagA.....................................................................   11    1   Mm1
...................guacccuguagaaccgaAcuagu.....................................................................   44    1   Mm1
...................guacccuguagaaccgGgcuagu.....................................................................   4     1   Mm1
...................guacccNguagaaccgagcuagu.....................................................................   1     1   Mm1
...................guacccuguagaaccgagcGagu.....................................................................   84    1   Mm1
...................guacccAguagaaccgagcuagu.....................................................................   1     1   Mm1
...................Auacccuguagaaccgagcuagug....................................................................   3     1   Mm1
...................guacccugCagaaccgagcuagug....................................................................   1     1   Mm1
...................guacccuguagaaccgCgcuagug....................................................................   1     1   Mm1
...................guacccuguagaaccgaAcuagug....................................................................   1     1   Mm1
...................guacccuguagaGccgagcuagug....................................................................   1     1   Mm1
...................guacccuguagaaccgagcuaguU....................................................................   321   1   Mm1
...................guacccuguagaaccgagcuagug....................................................................   412   0   Mm1
...................guacccuguagaaccgaCcuagug....................................................................   3     1   Mm1
...................guacccuguagaaccgUgcuagug....................................................................   1     1   Mm1
...................Cuacccuguagaaccgagcuagug....................................................................   5     1   Mm1
...................guacccuguUgaaccgagcuagug....................................................................   1     1   Mm1
...................guacccuguaAaaccgagcuagug....................................................................   1     1   Mm1
...................guacccuguagaaccgagcGagug....................................................................   1     1   Mm1
...................guacccuguagaaccgaUcuagug....................................................................   2     1   Mm1
...................guacccuguagaaccgGgcuagug....................................................................   1     1   Mm1
...................Auacccuguagaaccgagcuagugu...................................................................   2     1   Mm1
...................guacccuguagaaccgaUcuagugu...................................................................   6     1   Mm1
...................Uuacccuguagaaccgagcuagugu...................................................................   2     1   Mm1
...................Nuacccuguagaaccgagcuagugu...................................................................   1     1   Mm1
...................guacccuguagaacUgagcuagugu...................................................................   1     1   Mm1
...................guacccuguagaaccgagcuagugu...................................................................   775   0   Mm1
...................guacccuguaUaaccgagcuagugu...................................................................   1     1   Mm1
...................guacccuguagaaccgagcuagugG...................................................................   11    1   Mm1
...................guacccuguagaaccgCgcuagugu...................................................................   7     1   Mm1
...................guacccuguagaaccgagcGagugu...................................................................   11    1   Mm1
...................guacccuguagaaccgaAcuagugu...................................................................   5     1   Mm1
...................guacccuguagaGccgagcuagugu...................................................................   1     1   Mm1
...................guacccuguagaacAgagcuagugu...................................................................   1     1   Mm1
...................guacccuguagaaccgagcuaguUu...................................................................   28    1   Mm1
...................guacccuguagaaccUagcuagugu...................................................................   1     1   Mm1
...................guacccuguagaaccgagcuGgugu...................................................................   6     1   Mm1
...................guacccuguagaaccgagcuagugA...................................................................   23    1   Mm1
...................guacccuguagaaccgUgcuagugu...................................................................   1     1   Mm1
...................guacccuguagaaccgaCcuagugu...................................................................   2     1   Mm1
...................guacccuguaAaaccgagcuagugu...................................................................   1     1   Mm1
...................gAacccuguagaaccgagcuagugu...................................................................   2     1   Mm1
...................guacccuguagaaccgagcuUgugu...................................................................   1     1   Mm1
...................Cuacccuguagaaccgagcuagugu...................................................................   11    1   Mm1
...................guacccuguagaaccgagcuagugua..................................................................   15    0   Mm1
...................guacccuguagaaccgagcuaguguU..................................................................   58    1   Mm1
...................guacccuguagaaccgaAcuagugua..................................................................   1     1   Mm1
...................guacccuguagaaccgagcuaguguGucg...............................................................   1     1   Mm1
...................guacccuguagaaccgaCcuaguguaucg...............................................................   1     1   Mm1
...................guacccuguagaaccgagcuaguguaucg...............................................................   8     0   Mm1
...................guacccuguagaaccgagcuaguguaucgagu............................................................   9     0   Mm1
....................uacccuguagaaccgGgc.........................................................................   1     1   Mm1
....................uacccuguagaUccgagc.........................................................................   1     1   Mm1
....................uacccuguagaaccgagU.........................................................................   5     1   Mm1
....................uaUccuguagaaccgagc.........................................................................   1     1   Mm1
....................uUcccuguagaaccgagc.........................................................................   1     1   Mm1
....................uacccuguagaaccgaUc.........................................................................   2     1   Mm1
....................uacccuguagaaccgagA.........................................................................   21    1   Mm1
....................uacccuguagaaccgagc.........................................................................   471   0   Mm1
....................uacccuguagaaccgaCc.........................................................................   1     1   Mm1
....................uacccuguagaaccAagc.........................................................................   1     1   Mm1
....................uacccuguagaaccgagG.........................................................................   2     1   Mm1
....................uacccuguagUaccgagc.........................................................................   1     1   Mm1
....................uacccuguagaaUcgagcu........................................................................   1     1   Mm1
....................uacccuguagaaccgagcu........................................................................   2225  0   Mm1
....................uacccuguagaaccgaAcu........................................................................   6     1   Mm1
....................uacccuguagaaccgagGu........................................................................   7     1   Mm1
```

Mature **Star**

```
aaauguggugaguuucugugTacccuguagaaccgagcuaguguaucgaguucgaaacucacaagccauucuacaguguacaaagaaacgucgcuucuuucaacaaugaga
```

```
Mature                                          Star
aaauguggugaguuucuguguacccuguagaaccgagcuaguguaucgaguucgaaacucacaagccauucuacaguguacaaagaaacgucgcuucuuucaacaaugaga
....................uacUcuguagaaccgagcu........................................................................  2      1   Mm1
....................uacccuguagaacAgagcu........................................................................  1      1   Mm1
....................uacccuguagaaccgagUu........................................................................  10     1   Mm1
....................uacccuguaUaaccgagcu........................................................................  1      1   Mm1
....................uacAcuguagaaccgagcu........................................................................  1      1   Mm1
....................uacccuguagaaccgaCcu........................................................................  1      1   Mm1
....................uacccuguagaaccgaUcu........................................................................  3      1   Mm1
....................uacccuguagaaccgagcA........................................................................  6      1   Mm1
....................uacccuguagaacUgagcu........................................................................  1      1   Mm1
....................uacccuguagCaccgagcu........................................................................  1      1   Mm1
....................uaGccuguagaaccgagcu........................................................................  1      1   Mm1
....................uacccuAuagaaccgagcu........................................................................  2      1   Mm1
....................uacccAguagaaccgagcu........................................................................  1      1   Mm1
....................uacccuguagaaccgagAu........................................................................  23     1   Mm1
....................uacccuguagaaccgGgcu........................................................................  1      1   Mm1
....................uacccuguagaaccgaAcua.......................................................................  3      1   Mm1
....................uacccuguagaaUcgagcua.......................................................................  6      1   Mm1
....................uacccuguagaaccgagcAa.......................................................................  3      1   Mm1
....................uacccuguagaaccgagAua.......................................................................  2      1   Mm1
....................uacAcuguagaaccgagcua.......................................................................  1      1   Mm1
....................uacccuguagaGccgagcua.......................................................................  1      1   Mm1
....................uacccuguagaaccgagcua.......................................................................  435    0   Mm1
....................uacccuguagaaccgagcuU.......................................................................  101    1   Mm1
....................uacccuguagaaccgagcuC.......................................................................  2      1   Mm1
....................uGcccuguagaaccgagcua.......................................................................  1      1   Mm1
....................uacccuguagaaccgUgcua.......................................................................  1      1   Mm1
....................uacccuguagaaccgagUua.......................................................................  2      1   Mm1
....................uacccuguagaaccgagGua.......................................................................  1      1   Mm1
....................uaAccuguagaaccgagcua.......................................................................  1      1   Mm1
....................uacccCguagaaccgagcuag......................................................................  2      1   Mm1
....................uacccuguagaaUcgagcuag......................................................................  3      1   Mm1
....................uaccUuguagaaccgagcuag......................................................................  3      1   Mm1
....................uGcccuguagaaccgagcuag......................................................................  2      1   Mm1
....................uaUccuguagaaccgagcuag......................................................................  4      1   Mm1
....................uaccGuguagaaccgagcuag......................................................................  1      1   Mm1
....................uacccuguagCaccgagcuag......................................................................  10     1   Mm1
....................uacccuguagaaccgaUcuag......................................................................  31     1   Mm1
....................uCcccuguagaaccgagcuag......................................................................  2      1   Mm1
....................uacccugAagaaccgagcuag......................................................................  2      1   Mm1
....................uacccuguagaaccgagcuUg......................................................................  1      1   Mm1
....................Cacccuguagaaccgagcuag......................................................................  3      1   Mm1
....................uacccuguaAaaccgagcuag......................................................................  1      1   Mm1
....................uacccuguagaaccgagcuCg......................................................................  2      1   Mm1
....................uacccuguagaCccgagcuag......................................................................  1      1   Mm1
....................uacccuguagaaccgaAcuag......................................................................  21     1   Mm1
....................uacGcuguagaaccgagcuag......................................................................  1      1   Mm1
....................uacccugGagaaccgagcuag......................................................................  1      1   Mm1
....................uacccuguagaaccgagcuag......................................................................  12336  0   Mm1
....................uaAccuguagaaccgagcuag......................................................................  5      1   Mm1
....................uacccuguagaaccgagGuag......................................................................  42     1   Mm1
....................uacccuguagaaccAagcuag......................................................................  1      1   Mm1
....................uacccuguagaaccgGgcuag......................................................................  1      1   Mm1
....................Nacccuguagaaccgagcuag......................................................................  6      1   Mm1
....................uacccuguagaaccgagcuaU......................................................................  458    1   Mm1
....................uacccNguagaaccgagcuag......................................................................  2      1   Mm1
....................uacccugCagaaccgagcuag......................................................................  5      1   Mm1
....................uacccuguagaUccgagcuag......................................................................  2      1   Mm1
....................Gacccuguagaaccgagcuag......................................................................  2      1   Mm1
....................uacccuguagaaccUagcuag......................................................................  1      1   Mm1
....................uUcccuguagaaccgagcuag......................................................................  3      1   Mm1
....................uacccuguagaacUgagcuag......................................................................  2      1   Mm1
....................uacccuguagGaccgagcuag......................................................................  10     1   Mm1
....................uacccuguagaaccgaCcuag......................................................................  29     1   Mm1
....................uacccuguagaaccgUgcuag......................................................................  2      1   Mm1
....................uacccuguagUaccgagcuag......................................................................  13     1   Mm1
....................uacccuguagaaccgagUuag......................................................................  68     1   Mm1
....................uacAcuguagaaccgagcuag......................................................................  1      1   Mm1
....................uaccAuguagaaccgagcuag......................................................................  2      1   Mm1
....................uacccuguagaaccgagcuaA......................................................................  68     1   Mm1
....................uaGccuguagaaccgagcuag......................................................................  2      1   Mm1
```

Mature **Star**

```
aaauguggugaguuucugugUACCCUGUAGAACCGAGCUAGUguaucgaguucgaaacucacaagccauucuacaguguacaaagaaacgucgcuucuuucaacaaugaga
....................uacccuguagaaccgagcCag......................................................................  2       1  Mm1
....................uacccuguagaGccgagcuag......................................................................  6       1  Mm1
....................uacccuguUgaaccgagcuag......................................................................  2       1  Mm1
....................uacccuguagaaccgagcuGg......................................................................  13      1  Mm1
....................uacccuguagaaccgCgcuag......................................................................  1       1  Mm1
....................uacccuguagaaccgagAuag......................................................................  121     1  Mm1
....................uacccuUuagaaccgagcuag......................................................................  4       1  Mm1
....................uacAcuguagaaccgagcuagu.....................................................................  144     1  Mm1
....................uaccGuguagaaccgagcuagu.....................................................................  27      1  Mm1
....................uacccuNuagaaccgagcuagu.....................................................................  20      1  Mm1
....................uacccuguagaaccgagcuagC.....................................................................  46      1  Mm1
....................uacccuguagaaccgaUcuagu.....................................................................  838     1  Mm1
....................uacccuguagaaccgagcuCgu.....................................................................  28      1  Mm1
....................uacccuguagaaccgagcuUgu.....................................................................  222     1  Mm1
....................uUcccuguagaaccgagcuagu.....................................................................  86      1  Mm1
....................uacccuguagaacAgagcuagu.....................................................................  104     1  Mm1
....................uacccugGagaaccgagcuagu.....................................................................  15      1  Mm1
....................uacccuCuagaaccgagcuagu.....................................................................  14      1  Mm1
....................uCcccuguagaaccgagcuagu.....................................................................  34      1  Mm1
....................uacccuguagaaccgagcuagu.....................................................................  468090  0  Mm1
....................uacccugAagaaccgagcuagu.....................................................................  30      1  Mm1
....................uaGccuguagaaccgagcuagu.....................................................................  96      1  Mm1
....................uacccuguagaaAcgagcuagu.....................................................................  14      1  Mm1
....................uacccuguagaaccgUgcuagu.....................................................................  114     1  Mm1
....................Cacccuguagaaccgagcuagu.....................................................................  85      1  Mm1
....................uacccuguaCaaccgagcuagu.....................................................................  15      1  Mm1
....................uacccuguagaaccgagAuagu.....................................................................  4522    1  Mm1
....................Nacccuguagaaccgagcuagu.....................................................................  215     1  Mm1
....................uNcccuguagaaccgagcuagu.....................................................................  5       1  Mm1
....................uacccuguagaacGgagcuagu.....................................................................  23      1  Mm1
....................uacccuguagaGccgagcuagu.....................................................................  54      1  Mm1
....................uacccuguagaaccgGgcuagu.....................................................................  49      1  Mm1
....................uacccuguagaaccgagUuagu.....................................................................  2398    1  Mm1
....................uacccuguGgaaccgagcuagu.....................................................................  73      1  Mm1
....................uaUccuguagaaccgagcuagu.....................................................................  68      1  Mm1
....................uacccuguagaaGcgagcuagu.....................................................................  6       1  Mm1
....................uacGcuguagaaccgagcuagu.....................................................................  30      1  Mm1
....................uacccuguagGaccgagcuagu.....................................................................  499     1  Mm1
....................uacccuguagaaccgagcAagu.....................................................................  20      1  Mm1
....................uaAccuguagaaccgagcuagu.....................................................................  96      1  Mm1
....................uacccuguagaaccgagcuagG.....................................................................  176     1  Mm1
....................uacccuguCgaaccgagcuagu.....................................................................  11      1  Mm1
....................uacccuguaAaaccgagcuagu.....................................................................  38      1  Mm1
....................Gacccuguagaaccgagcuagu.....................................................................  12      1  Mm1
....................uacccuguagaaccUagcuagu.....................................................................  32      1  Mm1
....................uacccuguagaaccgagcuaAu.....................................................................  46      1  Mm1
....................uaNccuguagaaccgagcuagu.....................................................................  3       1  Mm1
....................uacccuguagaUccgagcuagu.....................................................................  104     1  Mm1
....................uacccGguagaaccgagcuagu.....................................................................  27      1  Mm1
....................uacccuguagaaccgaAcuagu.....................................................................  961     1  Mm1
....................uacccuguagaaccgaCcuagu.....................................................................  1062    1  Mm1
....................uacccuguagaaccgagcuagA.....................................................................  812     1  Mm1
....................uacccugCagaaccgagcuagu.....................................................................  49      1  Mm1
....................uGcccuguagaaccgagcuagu.....................................................................  80      1  Mm1
....................uacccuguUgaaccgagcuagu.....................................................................  75      1  Mm1
....................uacccuguagaCccgagcuagu.....................................................................  3       1  Mm1
....................uacccNguagaaccgagcuagu.....................................................................  66      1  Mm1
....................uacccuguagaaccgagcuaUu.....................................................................  79      1  Mm1
....................uacccuguagaaccgagcuGgu.....................................................................  498     1  Mm1
....................uacccuguagaaccgagcCagu.....................................................................  52      1  Mm1
....................Aacccuguagaaccgagcuagu.....................................................................  46      1  Mm1
....................uacccuguagaaUcgagcuagu.....................................................................  76      1  Mm1
....................uacUcuguagaaccgagcuagu.....................................................................  82      1  Mm1
....................uacccuguagaaccgCgcuagu.....................................................................  38      1  Mm1
....................uaccUuguagaaccgagcuagu.....................................................................  66      1  Mm1
....................uacccuUuagaaccgagcuagu.....................................................................  39      1  Mm1
....................uacccuguaUaaccgagcuagu.....................................................................  45      1  Mm1
....................uacccuguagUaccgagcuagu.....................................................................  424     1  Mm1
....................uacccuAuagaaccgagcuagu.....................................................................  43      1  Mm1
....................uaccAuguagaaccgagcuagu.....................................................................  66      1  Mm1
```

```
                   Mature                   Star
aaauguggugaguuucuguguacccuguagaaccgagcuaguguaucgaguucgaaacucacaagccauucuacaguguacaaagaaacgucgcuucuuucaacaaugaga
....................uacccuguagCaccgagcuagu.....................................................................  425    1  Mm1
....................uacccuguagaacUgagcuagu.....................................................................  164    1  Mm1
....................uacccuguagaaccAagcuagu.....................................................................  54     1  Mm1
....................uacccuguagaaccCagcuagu.....................................................................  9      1  Mm1
....................uacccCguagaaccgagcuagu.....................................................................  45     1  Mm1
....................uacccAguagaaccgagcuagu.....................................................................  19     1  Mm1
....................uacccuguagaaccgagcuaCu.....................................................................  10     1  Mm1
....................uacccuguagaaccgagGuagu.....................................................................  1592   1  Mm1
....................uacccuguagaaccgagcuGgug....................................................................  35     1  Mm1
....................uacUcuguagaaccgagcuagug....................................................................  4      1  Mm1
....................uacccuguagaaccgCgcuagug....................................................................  2      1  Mm1
....................uaccAuguagaaccgagcuagug....................................................................  1      1  Mm1
....................uacccuguagaaccgaCcuagug....................................................................  41     1  Mm1
....................uacccuguagaaccgUgcuagug....................................................................  5      1  Mm1
....................uacccuguagaaccgGgcuagug....................................................................  1      1  Mm1
....................Nacccuguagaaccgagcuagug....................................................................  6      1  Mm1
....................uacccuguUgaaccgagcuagug....................................................................  2      1  Mm1
....................uacccGguagaaccgagcuagug....................................................................  1      1  Mm1
....................uaUccuguagaaccgagcuagug....................................................................  3      1  Mm1
....................uacccuguagaaccgagcuaguU....................................................................  6985   1  Mm1
....................uaccUuguagaaccgagcuagug....................................................................  4      1  Mm1
....................Gacccuguagaaccgagcuagug....................................................................  3      1  Mm1
....................uacccuguagaaccgagcuaUug....................................................................  2      1  Mm1
....................uGcccuguagaaccgagcuagug....................................................................  1      1  Mm1
....................uacccuguaAaaccgagcuagug....................................................................  2      1  Mm1
....................uacccuguagaaccgagcuaguA....................................................................  1012   1  Mm1
....................uaAccuguagaaccgagcuagug....................................................................  5      1  Mm1
....................uacccugCagaaccgagcuagug....................................................................  3      1  Mm1
....................uacccuguagaaccgaUcuagug....................................................................  29     1  Mm1
....................uacccuguagaaccgagcuagAg....................................................................  1      1  Mm1
....................uacccuguagGaccgagcuagug....................................................................  17     1  Mm1
....................uacccuguagaaccgagUuagug....................................................................  76     1  Mm1
....................uacccuguagaaccgagcuagug....................................................................  16677  0  Mm1
....................uacAcuguagaaccgagcuagug....................................................................  4      1  Mm1
....................uacccuguagaaccgaAcuagug....................................................................  34     1  Mm1
....................uacccuguaUaaccgagcuagug....................................................................  3      1  Mm1
....................uacccuguagUaccgagcuagug....................................................................  15     1  Mm1
....................uCcccuguagaaccgagcuagug....................................................................  1      1  Mm1
....................uacccuguagaaUcgagcuagug....................................................................  1      1  Mm1
....................uacccuguCgaaccgagcuagug....................................................................  1      1  Mm1
....................uacccuguagaaccgagcuUgug....................................................................  3      1  Mm1
....................uacccuguagCaccgagcuagug....................................................................  20     1  Mm1
....................uaGccuguagaaccgagcuagug....................................................................  3      1  Mm1
....................uUcccuguagaaccgagcuagug....................................................................  3      1  Mm1
....................uacccuNuagaaccgagcuagug....................................................................  1      1  Mm1
....................uacccuguagaaccgagcAagug....................................................................  2      1  Mm1
....................uacccuguagaacGgagcuagug....................................................................  1      1  Mm1
....................uacccuguagaaccgagGuagug....................................................................  53     1  Mm1
....................uaccGuguagaaccgagcuagug....................................................................  2      1  Mm1
....................uacccuguagaacAgagcuagug....................................................................  5      1  Mm1
....................uacccNguagaaccgagcuagug....................................................................  2      1  Mm1
....................uacccuguGgaaccgagcuagug....................................................................  4      1  Mm1
....................uacccuguagaaccgagcuagCg....................................................................  4      1  Mm1
....................uacccuguagaacUgagcuagug....................................................................  3      1  Mm1
....................uacccuAuagaaccgagcuagug....................................................................  1      1  Mm1
....................uacccCguagaaccgagcuagug....................................................................  3      1  Mm1
....................uacccuguagaaccgagcuagGg....................................................................  2      1  Mm1
....................uacccuguagaaccgagAuagug....................................................................  118    1  Mm1
....................uacccuguagaaccgagcuaguC....................................................................  10     1  Mm1
....................uacccuCuagaaccgagcuagugu...................................................................  3      1  Mm1
....................uacccuguagaaccCagcuagugu...................................................................  3      1  Mm1
....................uacccuguagaaccgagcuagugG...................................................................  531    1  Mm1
....................uacccuguaAaaccgagcuagugu...................................................................  2      1  Mm1
....................uacccuguagaaAcgagcuagugu...................................................................  1      1  Mm1
....................uacccNguagaaccgagcuagugu...................................................................  8      1  Mm1
....................uacccuguagaGccgagcuagugu...................................................................  6      1  Mm1
....................uacccuguagaaccgagcuaguUu...................................................................  893    1  Mm1
....................uacccuguagaaccgagUuagugu...................................................................  217    1  Mm1
....................uacccuguagaaccgagcGagugu...................................................................  28     1  Mm1
....................uacccAguagaaccgagcuagugu...................................................................  4      1  Mm1
```

Mature **Star**

```
aaauguggugaguuucuguguacccuguagaaccgagcuaguguaucgaguucgaaacucacaagccauucuacaguguacaaagaaacgucgcuucuuucaacaaugaga
....................uacccuguagaaccUagcuagugu...................................................................  5      1  Mm1
....................uacccuUuagaaccgagcuagugu...................................................................  3      1  Mm1
....................uacccugCagaaccgagcuagugu...................................................................  4      1  Mm1
....................uacUcuguagaaccgagcuagugu...................................................................  2      1  Mm1
....................uacccuguagaaccgagcuagGgu...................................................................  2      1  Mm1
....................uaAccuguagaaccgagcuagugu...................................................................  11     1  Mm1
....................uacccuguagaaccgagGuagugu...................................................................  152    1  Mm1
....................uacGcuguagaaccgagcuagugu...................................................................  5      1  Mm1
....................uacAcuguagaaccgagcuagugu...................................................................  8      1  Mm1
....................uacccuguGgaaccgagcuagugu...................................................................  7      1  Mm1
....................uacccuguaCaaccgagcuagugu...................................................................  2      1  Mm1
....................uacccuguagaaUcgagcuagugu...................................................................  5      1  Mm1
....................uUcccuguagaaccgagcuagugu...................................................................  9      1  Mm1
....................uacccuguagGaccgagcuagugu...................................................................  43     1  Mm1
....................uacccuguagaaccgagcuCgugu...................................................................  2      1  Mm1
....................uacccuguagaaccgaUcuagugu...................................................................  93     1  Mm1
....................uacccuguagaaccgCgcuagugu...................................................................  2      1  Mm1
....................uacccuguagCaccgagcuagugu...................................................................  48     1  Mm1
....................uacccuguagaaGcgagcuagugu...................................................................  2      1  Mm1
....................uacccuguagaaccAagcuagugu...................................................................  6      1  Mm1
....................uacccuguagaaccgagcuaUugu...................................................................  4      1  Mm1
....................uacccuguagaaccgagcuaAugu...................................................................  7      1  Mm1
....................uacccuguagaaccgUgcuagugu...................................................................  6      1  Mm1
....................uacccuguagaaccgagcuaguAu...................................................................  766    1  Mm1
....................uaccGuguagaaccgagcuagugu...................................................................  3      1  Mm1
....................Nacccuguagaaccgagcuagugu...................................................................  24     1  Mm1
....................uacccuguaUaaccgagcuagugu...................................................................  4      1  Mm1
....................uacccuguagaacAgagcuagugu...................................................................  9      1  Mm1
....................uGcccuguagaaccgagcuagugu...................................................................  4      1  Mm1
....................uacccuguagaaccgGgcuagugu...................................................................  6      1  Mm1
....................uacccuguUgaaccgagcuagugu...................................................................  11     1  Mm1
....................uacccuguagaCccgagcuagugu...................................................................  1      1  Mm1
....................uacccuNuagaaccgagcuagugu...................................................................  2      1  Mm1
....................uacccuguagaaccgagcuaguCu...................................................................  7      1  Mm1
....................uacccuguagaaccgagcCagugu...................................................................  2      1  Mm1
....................uacccuguCgaaccgagcuagugu...................................................................  1      1  Mm1
....................uNcccuguagaaccgagcuagugu...................................................................  1      1  Mm1
....................uacccuguagaaccgaAcuagugu...................................................................  84     1  Mm1
....................Cacccuguagaaccgagcuagugu...................................................................  14     1  Mm1
....................uacccuguagaUccgagcuagugu...................................................................  8      1  Mm1
....................uacccuguagaaccgagAuagugu...................................................................  458    1  Mm1
....................uaNccuguagaaccgagcuagugu...................................................................  1      1  Mm1
....................uaGccuguagaaccgagcuagugu...................................................................  12     1  Mm1
....................Aacccuguagaaccgagcuagugu...................................................................  4      1  Mm1
....................uaUccuguagaaccgagcuagugu...................................................................  6      1  Mm1
....................uacccuguagaaccgagcuGgugu...................................................................  70     1  Mm1
....................uacccuguagaaccgagcAagugu...................................................................  2      1  Mm1
....................uacccuguagaaccgagcuagugu...................................................................  45757  0  Mm1
....................uaccUuguagaaccgagcuagugu...................................................................  4      1  Mm1
....................uacccuguagaaccgagcuagugA...................................................................  1034   1  Mm1
....................Gacccuguagaaccgagcuagugu...................................................................  1      1  Mm1
....................uacccCguagaaccgagcuagugu...................................................................  5      1  Mm1
....................uacccuguagaaccgagcuagCgu...................................................................  16     1  Mm1
....................uacccuguagaaccgagcuagugC...................................................................  51     1  Mm1
....................uacccGguagaaccgagcuagugu...................................................................  3      1  Mm1
....................uacccuguagUaccgagcuagugu...................................................................  43     1  Mm1
....................uacccugAagaaccgagcuagugu...................................................................  4      1  Mm1
....................uacccugGagaaccgagcuagugu...................................................................  1      1  Mm1
....................uacccuguagaaccgaCcuagugu...................................................................  105    1  Mm1
....................uacccuguagaaccgagcuUgugu...................................................................  10     1  Mm1
....................uacccuguagaacGgagcuagugu...................................................................  2      1  Mm1
....................uaccAuguagaaccgagcuagugu...................................................................  9      1  Mm1
....................uacccuguagaaccgagcuagAgu...................................................................  5      1  Mm1
....................uacccuguagaacUgagcuagugu...................................................................  7      1  Mm1
....................uacccuAuagaaccgagcuagugu...................................................................  6      1  Mm1
....................uCcccuguagaaccgagcuagugu...................................................................  1      1  Mm1
....................uacccuguagaaccgagcuagugAa..................................................................  10     1  Mm1
....................uacccuguagaaccgagcuaguguG..................................................................  5      1  Mm1
....................uacccuguagaaccgagcuagugua..................................................................  17     0  Mm1
....................uacccuguagaaccgagcuaguguU..................................................................  1615   1  Mm1
```

Mature Star

```
aaauguggugaguuucugug uacccuguagaaccgagcuagu guaucgaguucgaaacuca caagccauucuacaguguaca aagaaacgucgcuucuuucaacaaugaga
....................uacccuguagaaccgagcuagugCa..................................................................  6     1  Mm1
....................uacccuguagaaccgagcuaguguau.................................................................  16    0  Mm1
....................uUcccuguagaaccgagcuaguguau.................................................................  1     1  Mm1
....................uacccuguagaaccgagcuaguguUu.................................................................  36    1  Mm1
....................uacccuguagaaccgagUuaguguau.................................................................  1     1  Mm1
....................uacccuguagaaccgagcuaUuguauc................................................................  1     1  Mm1
....................uacccuguagGaccgagcuaguguauc................................................................  1     1  Mm1
....................uacccuguagaaccgagAuaguguauc................................................................  2     1  Mm1
....................uacccuguagaaccgagcuaguguauc................................................................  71    0  Mm1
....................uacccuguagaaccgagcuaguguaucg...............................................................  5     0  Mm1
....................uacccuguagaaccgagcuaguguaucgag.............................................................  3     0  Mm1
....................uacccuguagaaccgagcuaguguaucgagu............................................................  4     0  Mm1
....................uacccuguagaaccgagcuaguguaucgaguu...........................................................  7     0  Mm1
.....................acccuguagaaccgagcu........................................................................  26    0  Mm1
.....................acccuguagaaccgagcC........................................................................  1     1  Mm1
.....................acccuguagaaccgagcua.......................................................................  2     0  Mm1
.....................acccuguagaaccgagcuag......................................................................  75    0  Mm1
.....................acccuguagaaccgagcuaU......................................................................  5     1  Mm1
.....................acGcuguagaaccgagcuagu.....................................................................  1     1  Mm1
.....................acccuguagUaccgagcuagu.....................................................................  1     1  Mm1
.....................acccuguaAaaccgagcuagu.....................................................................  1     1  Mm1
.....................acccuguagaaccgagcCagu.....................................................................  11    1  Mm1
.....................acccuguagaaccgaCcuagu.....................................................................  1     1  Mm1
.....................acccuguagaaccgagcAagu.....................................................................  8     1  Mm1
.....................acccuguagaacUgagcuagu.....................................................................  2     1  Mm1
.....................acccuguaUaaccgagcuagu.....................................................................  3     1  Mm1
.....................acccuUuagaaccgagcuagu.....................................................................  3     1  Mm1
.....................acccuguagaaAcgagcuagu.....................................................................  1     1  Mm1
.....................Gcccuguagaaccgagcuagu.....................................................................  1     1  Mm1
.....................acccuguagaaccgCgcuagu.....................................................................  6     1  Mm1
.....................acccuguagaaccgagcuCgu.....................................................................  1     1  Mm1
.....................acAcuguagaaccgagcuagu.....................................................................  1     1  Mm1
.....................acccuguagaaccgagcuGgu.....................................................................  3     1  Mm1
.....................acccugAagaaccgagcuagu.....................................................................  2     1  Mm1
.....................acccuguagaaUcgagcuagu.....................................................................  2     1  Mm1
.....................Ucccuguagaaccgagcuagu.....................................................................  3     1  Mm1
.....................acccuAuagaaccgagcuagu.....................................................................  1     1  Mm1
.....................acccuguagaUccgagcuagu.....................................................................  3     1  Mm1
.....................acccuguagaaccgagcuagu.....................................................................  2292  0  Mm1
.....................acccuguagaaccgagAuagu.....................................................................  3     1  Mm1
.....................acccuguagaacAgagcuagu.....................................................................  3     1  Mm1
.....................acccuguagaGccgagcuagu.....................................................................  2     1  Mm1
.....................acccugNagaaccgagcuagu.....................................................................  1     1  Mm1
.....................acccuguagaaccgaUcuagu.....................................................................  1     1  Mm1
.....................acccuCuagaaccgagcuagu.....................................................................  1     1  Mm1
.....................acccuguaCaaccgagcuagu.....................................................................  1     1  Mm1
.....................acccuguagaaccgagcuaAu.....................................................................  8     1  Mm1
.....................acccuguagaaccgUgcuagu.....................................................................  1     1  Mm1
.....................acccuguCgaaccgagcuagu.....................................................................  1     1  Mm1
.....................accAuguagaaccgagcuagu.....................................................................  3     1  Mm1
.....................acccuguagaaccgagcuagG.....................................................................  1     1  Mm1
.....................acccuguagCaccgagcuagu.....................................................................  1     1  Mm1
.....................acccuguagaaccgagcuaCu.....................................................................  2     1  Mm1
.....................accGuguagaaccgagcuagu.....................................................................  1     1  Mm1
.....................acccuguagaaccgagUuagu.....................................................................  2     1  Mm1
.....................acccuguUgaaccgagcuagu.....................................................................  1     1  Mm1
.....................acccuguagaCccgagcuagu.....................................................................  2     1  Mm1
.....................acccuguagaaccgagcuUgu.....................................................................  13    1  Mm1
.....................aAccuguagaaccgagcuagu.....................................................................  1     1  Mm1
.....................acccugCagaaccgagcuagu.....................................................................  1     1  Mm1
.....................acccuguagaaccgaAcuagu.....................................................................  1     1  Mm1
.....................accUuguagaaccgagcuagu.....................................................................  1     1  Mm1
.....................acccuguagaaccgagcuagug....................................................................  88    0  Mm1
.....................acccuguagaGccgagcuagug....................................................................  1     1  Mm1
.....................acccuguagaaccgagcuaguU....................................................................  46    1  Mm1
.....................acccuguagaaccgagcuaguAu...................................................................  5     1  Mm1
.....................acccuguagaaccgCgcuagugu...................................................................  1     1  Mm1
.....................acccuguagaaccgagcuagugG...................................................................  7     1  Mm1
.....................acccuguagaaccgagcAagugu...................................................................  3     1  Mm1
.....................acUcuguagaaccgagcuagugu...................................................................  1     1  Mm1
```

Mature **Star**

```
aaauguggugagauuucuguguacccuguagaaccgagcuaguguaucgaguucgaaacucacaagccauucuacaguguacaaagaaacgucgcuucuuucaacaaugaga
......................acccuguagaaccgagcuaCugu...................................................................   1     1    Mm1
......................acccuguagaaccgagUuagugu...................................................................   1     1    Mm1
......................acccuguagaCccgagcuagugu...................................................................   1     1    Mm1
......................acccuguagaaccgUgcuagugu...................................................................   1     1    Mm1
......................acccuguagaaccgagcuagCgu...................................................................   1     1    Mm1
......................acccuguagaaccgagcuagugC...................................................................   2     1    Mm1
......................acccuguagaaccgagcuagugu...................................................................   462   0    Mm1
......................acccuguagaaccgagcuaguUu...................................................................   10    1    Mm1
......................acccuguagaaccgagcCagugu...................................................................   1     1    Mm1
......................acccuguagaaccgagAuagugu...................................................................   1     1    Mm1
......................acccuguagaacAgagcuagugu...................................................................   1     1    Mm1
......................acccuguagaaccAagcuagugu...................................................................   1     1    Mm1
......................acccuguagaaccgagcuagugA...................................................................   2     1    Mm1
......................acccuguagaaccgagcGagugu...................................................................   4     1    Mm1
......................Ncccuguagaaccgagcuagugu...................................................................   1     1    Mm1
......................acccuguagaaccgagcuaguguU..................................................................   22    1    Mm1
......................acccuguagaaccgagcuaguguau.................................................................   3     0    Mm1
......................acccuguagaaccgagcuaguguauc................................................................   5     0    Mm1
......................acccuguagaaccgagcuaguguaucU...............................................................   6     1    Mm1
......................acccuguagaaccgagcuaguguaucgagu............................................................   15    0    Mm1
......................acccuguagaaccgagcuaguguaucgaguu...........................................................   7     0    Mm1
......................acccuguagaaccgagcuaguguaucgaguucga........................................................   4     0    Mm1
.......................cccuguagaaccgagcuag......................................................................   3     0    Mm1
.......................cccuguagaaccgaCcuagu.....................................................................   1     1    Mm1
.......................cccuguagaaccgagcuagu.....................................................................   85    0    Mm1
.......................cccuguagaaccgagcuaguU....................................................................   8     1    Mm1
.......................cccuguagaaccgagcuaguUu...................................................................   1     1    Mm1
.......................cccuguagaaccgagcuGgugu...................................................................   1     1    Mm1
.......................cccuguagaaccgagcuagugu...................................................................   2     0    Mm1
........................ccuguagaaccgagcuagu.....................................................................   4     0    Mm1
..............................................................caagccauucuacagugG................................   2     1    Mm1
..............................................................caagccauucGacagugu................................   1     1    Mm1
..............................................................caagccauucuacagugu................................   17    0    Mm1
..............................................................caagccauucuacagugAa...............................   1     1    Mm1
..............................................................caagccauucuacaguguU...............................   8     1    Mm1
..............................................................caagccauucuacagugua...............................   17    0    Mm1
..............................................................Aaagccauucuacaguguac..............................   1     1    Mm1
..............................................................caagccauucuacagugCac..............................   5     1    Mm1
..............................................................caagccauucuacaguguac..............................   605   0    Mm1
..............................................................caagccauucuacaguCuac..............................   2     1    Mm1
..............................................................caagccauucuacaguUuac..............................   2     1    Mm1
..............................................................cUagccauucuacaguguac..............................   1     1    Mm1
..............................................................caagccauucuacaguguaU..............................   1     1    Mm1
..............................................................caagccauucAacaguguac..............................   1     1    Mm1
..............................................................caagccauucuacagugGac..............................   3     1    Mm1
..............................................................Uaagccauucuacaguguac..............................   2     1    Mm1
..............................................................Naagccauucuacaguguac..............................   1     1    Mm1
..............................................................caagccauucuacagugAac..............................   2     1    Mm1
..............................................................caagccauAcuacaguguaca.............................   1     1    Mm1
..............................................................Naagccauucuacaguguaca.............................   1     1    Mm1
..............................................................caGgccauucuacaguguaca.............................   1     1    Mm1
..............................................................caagccauucuacaguUuaca.............................   2     1    Mm1
..............................................................caagccauucuaUaguguaca.............................   1     1    Mm1
..............................................................caagccauucuacaguAuaca.............................   13    1    Mm1
..............................................................caagcAauucuacaguguaca.............................   1     1    Mm1
..............................................................caagccauucAacaguguaca.............................   5     1    Mm1
..............................................................caagccauucuacagugGaca.............................   13    1    Mm1
..............................................................caagccauucCacaguguaca.............................   2     1    Mm1
..............................................................caagcUauucuacaguguaca.............................   2     1    Mm1
..............................................................Aaagccauucuacaguguaca.............................   1     1    Mm1
..............................................................caagccauucuacagugCaca.............................   45    1    Mm1
..............................................................cCagccauucuacaguguaca.............................   3     1    Mm1
..............................................................caagccauucGacaguguaca.............................   7     1    Mm1
..............................................................Uaagccauucuacaguguaca.............................   5     1    Mm1
..............................................................caUgccauucuacaguguaca.............................   1     1    Mm1
..............................................................caagccauucuacaguguCca.............................   1     1    Mm1
..............................................................caagccauucuacagugAaca.............................   28    1    Mm1
..............................................................caagccauucuacagCguaca.............................   1     1    Mm1
..............................................................caagccauucuacaguCuaca.............................   12    1    Mm1
..............................................................caagccauucuacaguguacG.............................   3     1    Mm1
```

```
                    Mature                                   Star
aaauguggugaguuucugug uacccuguagaaccgagcuagu guaucgaguucgaaacuca caagccauucuacaguguaca aagaaacgucgcuucuuucaacaaugaga
.............................................................caagccauucuacaguguaca.............................   5447   0   Mm1
.............................................................caagccUuucuacaguguaca.............................   1      1   Mm1
.............................................................cGagccauucuacaguguaca.............................   2      1   Mm1
.............................................................caagccaCucuacaguguaca.............................   1      1   Mm1
.............................................................caagAcauucuacaguguaca.............................   1      1   Mm1
.............................................................caagccauucuacaguguaUa.............................   2      1   Mm1
.............................................................caagccauucuacagAguaca.............................   1      1   Mm1
.............................................................caagccGuucuacaguguaca.............................   1      1   Mm1
.............................................................caagccauucuacaguguacU.............................   11     1   Mm1
.............................................................caagccauucuacaguguacaa............................   676    0   Mm1
.............................................................caagccauucuacaguguacaU............................   26     1   Mm1
.............................................................caagccauucuacaguguacaC............................   98     1   Mm1
.............................................................caagccauucuacagugAacaa............................   2      1   Mm1
.............................................................Aaagccauucuacaguguacaa............................   1      1   Mm1
.............................................................caagccauucuacaguCuacaa............................   2      1   Mm1
.............................................................caagccauucAacaguguacaa............................   1      1   Mm1
.............................................................caagccauucuacagugGacaa............................   1      1   Mm1
.............................................................caagccauucuacagugCacaa............................   5      1   Mm1
.............................................................caagccauucuacaguguacaG............................   1      1   Mm1
.............................................................caagccauucuacaguguacaaa...........................   9      0   Mm1
.............................................................caagccauucuacaguguacaaU...........................   25     1   Mm1
.............................................................caagccauucuacaguguacaaaU..........................   15     1   Mm1
..............................................................aagccauucuacaguguac..............................   5      0   Mm1
..............................................................aagccauucuacaguguaca.............................   39     0   Mm1
..............................................................aagccauucuUcaguguaca.............................   1      1   Mm1
..............................................................aagccauucuacaguguacaC............................   4      1   Mm1
..............................................................aagccauucuacaguguacaa............................   3      0   Mm1

...............cuguguacccuguagaaccgagc.........................................................................   13     0   Mm2
...............cuguguacccuguagaaAcgagcu........................................................................   1      1   Mm2
...............cuguguacccuguagaaccgagcu........................................................................   27     0   Mm2
...............cuguguacccuguagaaccgagcA........................................................................   1      1   Mm2
................uguguacccuguagaacAgagc.........................................................................   3      1   Mm2
................uguguacccuguagaaccgagc.........................................................................   56     0   Mm2
................uguguacccuguagaaccgagU.........................................................................   1      1   Mm2
................uguguacccuguagaaccgagcu........................................................................   275    0   Mm2
................uguguacccuguagaaccgagcC........................................................................   1      1   Mm2
................uguguacccuguagaaAcgagcu........................................................................   1      1   Mm2
................uguguacccuguagaaccgagcA........................................................................   1      1   Mm2
................uguguacccuguagaacAgagcu........................................................................   1      1   Mm2
................uguguacccuguagaaccgagcG........................................................................   2      1   Mm2
................uguguacccuguagaacUgagcu........................................................................   1      1   Mm2
................uguguacccCguagaaccgagcu........................................................................   1      1   Mm2
................uguguacccuguagaaccgagcuU.......................................................................   8      1   Mm2
................uCuguacccuguagaaccgagcuagu.....................................................................   1      1   Mm2
.................guguacccuguagaaccgagcu........................................................................   1      0   Mm2
.................Cuguacccuguagaaccgagcuag......................................................................   1      1   Mm2
.................guguacccuguagaaccgagcuag......................................................................   1      0   Mm2
.................Nuguacccuguagaaccgagcuagu.....................................................................   1      1   Mm2
.................guguacccuguagaaccgagcuagu.....................................................................   1      0   Mm2
.................Cuguacccuguagaaccgagcuagu.....................................................................   17     1   Mm2
.................guguacccuguagaaccgagcuaguU....................................................................   2      1   Mm2
..................uguacccuguagaaccga...........................................................................   10     0   Mm2
..................uguacccuguagaaccgag..........................................................................   3      0   Mm2
..................uguacccuguagaaccgagc.........................................................................   265    0   Mm2
..................uguacccuguagaaccgagA.........................................................................   1      1   Mm2
..................uguacccuguagaaccgCgc.........................................................................   3      1   Mm2
..................uguacccuguGgaaccgagc.........................................................................   1      1   Mm2
..................uguacccuguagaaccUagc.........................................................................   1      1   Mm2
..................uguacccuguagaaccgagU.........................................................................   1      1   Mm2
..................uguacccuguagaacUgagcu........................................................................   1      1   Mm2
..................uguacccuguagaaccgagUu........................................................................   1      1   Mm2
..................uguacccuguagaaccgagcA........................................................................   2      1   Mm2
..................uguacccuguagaaccgCgcu........................................................................   9      1   Mm2
..................uguacccuguagaaccAagcu........................................................................   7      1   Mm2
..................uguacccuguagaaccgGgcu........................................................................   7      1   Mm2
..................uguacccuguagaaccUagcu........................................................................   2      1   Mm2
..................ugAacccuguagaaccgagcu........................................................................   1      1   Mm2
..................uguacccuguagaaccgUgcu........................................................................   3      1   Mm2
..................Cguacccuguagaaccgagcu........................................................................   2      1   Mm2
```

Mature Star

aaauguggugaguuucugug**uacccuguagaaccgagcuagu**guaucgaguucgaaacuca**caagccauucuacaguguaca**aagaaacgucgcuucuuucaacaaugaga			
..................uguacccuguagaaccgagcG..	7	1	Mm2
..................uguacccuguagaaccCagcu..	5	1	Mm2
..................uguacNcuguagaaccgagcu..	1	1	Mm2
..................uguacccuguagaaccgagcu..	1501	0	Mm2
..................uguacccuguagaaUcgagcu..	1	1	Mm2
..................uguacccCguagaaccgagcu..	1	1	Mm2
..................uCuacccuguagaaccgagcu..	1	1	Mm2
..................AguacccuguagaaccgagcU..	1	1	Mm2
..................uguacccuguUgaaccgagcu..	1	1	Mm2
..................uguacccuguGgaaccgagcu..	1	1	Mm2
..................uguacccuguagaaccgagcua...	447	0	Mm2
..................uguacccuguagaaccgagcuU...	41	1	Mm2
..................uguacccuguagaaccgagUua...	1	1	Mm2
..................uguacccuguagaaccAagcua...	1	1	Mm2
..................uguacccuguagaaUcgagcua...	1	1	Mm2
..................uguacccuguagaaccgaUcua...	1	1	Mm2
..................uguacccuguagaaccgagcAa...	2	1	Mm2
..................uguacccuguagaaccgGgcua...	1	1	Mm2
..................uguaccNuguagaaccgagcuag..	1	1	Mm2
..................uguacccuguagaaccgagcuag..	8999	0	Mm2
..................uguacccuguagaaccgagcuaC..	1	1	Mm2
..................uguacccugGagaaccgagcuag..	1	1	Mm2
..................uguacccuguCgaaccgagcuag..	11	1	Mm2
..................uguacccuguagaaccgGgcuag..	19	1	Mm2
..................uguacccuguGgaaccgagcuag..	12	1	Mm2
..................uguacccuguagaaccgagcuaU..	460	1	Mm2
..................uguaccUuguagaaccgagcuag..	1	1	Mm2
..................uguGcccuguagaaccgagcuag..	3	1	Mm2
..................ugAacccuguagaaccgagcuag..	6	1	Mm2
..................Nguacccuguagaaccgagcuag..	5	1	Mm2
..................uguacccuguagaaccgagcuCg..	1	1	Mm2
..................uguacccuguagaaUcgagcuag..	2	1	Mm2
..................uguacccuguagaaccgCgcuag..	39	1	Mm2
..................uguacccGguagaaccgagcuag..	2	1	Mm2
..................uguacccuguagaaGcgagcuag..	1	1	Mm2
..................uguacccuguagaaccgagcuaA..	32	1	Mm2
..................uguacccuguaAaaccgagcuag..	4	1	Mm2
..................uguacccuguagUaccgagcuag..	1	1	Mm2
..................uguUcccuguagaaccgagcuag..	1	1	Mm2
..................uguacccuguagaaccgaAcuag..	2	1	Mm2
..................uAuacccuguagaaccgagcuag..	6	1	Mm2
..................uguacccuguagaGccgagcuag..	5	1	Mm2
..................uguacGcuguagaaccgagcuag..	1	1	Mm2
..................uguacccuguagaaccCagcuag..	17	1	Mm2
..................uguacNcuguagaaccgagcuag..	6	1	Mm2
..................uguacccuguUgaaccgagcuag..	9	1	Mm2
..................uguacAcuguagaaccgagcuag..	2	1	Mm2
..................uguacccuguagaaccgagcCag..	1	1	Mm2
..................uguacccuguagaaccAagcuag..	26	1	Mm2
..................uguacccuguaUaaccgagcuag..	2	1	Mm2
..................uguacccuguagaaccUagcuag..	9	1	Mm2
..................uguacccuUuagaaccgagcuag..	1	1	Mm2
..................ugCacccuguagaaccgagcuag..	1	1	Mm2
..................uguacccuguagaaccgagAuag..	1	1	Mm2
..................uguacccuguagaaccgagcuGg..	9	1	Mm2
..................ugGacccuguagaaccgagcuag..	2	1	Mm2
..................uguaUccuguagaaccgagcuag..	2	1	Mm2
..................Cguacccuguagaaccgagcuag..	5	1	Mm2
..................uguacccuguagaaccgagUuag..	21	1	Mm2
..................uguacccuguagaaccgagcGag..	7	1	Mm2
..................uguacccuguagaaccgUgcuag..	32	1	Mm2
..................uguacUcuguagaaccgagcuag..	7	1	Mm2
..................uguaAccuguagaaccgagcuag..	1	1	Mm2
..................uUuacccuguagaaccgagcuag..	4	1	Mm2
..................uguaGccuguagaaccgagcuag..	1	1	Mm2
..................uguacccuguagaacUgagcuag..	3	1	Mm2
..................uguaAccuguagaaccgagcuagu...	8	1	Mm2
..................uguacccCguagaaccgagcuagu...	17	1	Mm2
..................uguCcccuguagaaccgagcuagu...	3	1	Mm2
..................uguacccuguaAaaccgagcuagu...	6	1	Mm2

```
                    Mature                                  Star
aaauguggugaguuucugugguacccuguagaaccgagcuaguguaucgaguucgaaacucacaagccauucuacaguguacaaagaaacgucgcuucuuucaacaaugaga
..................uguacccuUuagaaccgagcuagu..................................................................... 10 1 Mm2
..................uguacccuguagCaccgagcuagu..................................................................... 7 1 Mm2
..................uguaUccuguagaaccgagcuagu..................................................................... 5 1 Mm2
..................uguacccuguagaaAcgagcuagu..................................................................... 4 1 Mm2
..................uguaGccuguagaaccgagcuagu..................................................................... 4 1 Mm2
..................Gguacccuguagaaccgagcuagu..................................................................... 1 1 Mm2
..................uguacccuguagaaccgagcuaAu..................................................................... 19 1 Mm2
..................uguacccuguagaaccgagcuCgu..................................................................... 7 1 Mm2
..................uguacNcuguagaaccgagcuagu..................................................................... 5 1 Mm2
..................uguacccuAuagaaccgagcuagu..................................................................... 10 1 Mm2
..................uguacUcuguagaaccgagcuagu..................................................................... 74 1 Mm2
..................uguacccuguagaaccgagcuagG..................................................................... 124 1 Mm2
..................uguacccuguagaaccgagAuagu..................................................................... 5 1 Mm2
..................uguacccuguagaaccgagcuagu..................................................................... 81504 0 Mm2
..................uguacccuguagaaccgagcuGgu..................................................................... 47 1 Mm2
..................uUuacccuguagaaccgagcuagu..................................................................... 16 1 Mm2
..................uguacccuguagGaccgagcuagu..................................................................... 8 1 Mm2
..................uguacccuguagaaccgagcuagA..................................................................... 204 1 Mm2
..................uguaccUuguagaaccgagcuagu..................................................................... 14 1 Mm2
..................uguaccGuguagaaccgagcuagu..................................................................... 3 1 Mm2
..................uguacccuguGgaaccgagcuagu..................................................................... 102 1 Mm2
..................uguacccuguagaaccgagcGagu..................................................................... 170 1 Mm2
..................uguacccuguagaaccgagcuaCu..................................................................... 2 1 Mm2
..................uguacccuguagaaccgUgcuagu..................................................................... 266 1 Mm2
..................uguacccuguagaaccgCgcuagu..................................................................... 336 1 Mm2
..................uguacccuguagaGccgagcuagu..................................................................... 9 1 Mm2
..................uguGcccuguagaaccgagcuagu..................................................................... 7 1 Mm2
..................uCuacccuguagaaccgagcuagu..................................................................... 46 1 Mm2
..................uguacccuguagaaccgagcuUgu..................................................................... 12 1 Mm2
..................uguacccuCuagaaccgagcuagu..................................................................... 2 1 Mm2
..................uguacAcuguagaaccgagcuagu..................................................................... 9 1 Mm2
..................uguacccuguCgaaccgagcuagu..................................................................... 71 1 Mm2
..................ugAacccuguagaaccgagcuagu..................................................................... 21 1 Mm2
..................uguacccuguagaaccgagGuagu..................................................................... 3 1 Mm2
..................uNuacccuguagaaccgagcuagu..................................................................... 1 1 Mm2
..................uguacccuguagaaccgaAcuagu..................................................................... 9 1 Mm2
..................Aguacccuguagaaccgagcuagu..................................................................... 11 1 Mm2
..................uguacccugCagaaccgagcuagu..................................................................... 8 1 Mm2
..................Cguacccuguagaaccgagcuagu..................................................................... 24 1 Mm2
..................uguacccugAagaaccgagcuagu..................................................................... 2 1 Mm2
..................uguacccuguagaaccgagcuaUu..................................................................... 71 1 Mm2
..................uguacccuguagaaccgagcAagu..................................................................... 2 1 Mm2
..................uguacccuguagaaccgaUcuagu..................................................................... 15 1 Mm2
..................uguacccugGagaaccgagcuagu..................................................................... 3 1 Mm2
..................uguacccuguagaacAgagcuagu..................................................................... 5 1 Mm2
..................uguacccuguagaaccgagcCagu..................................................................... 7 1 Mm2
..................uguacGcuguagaaccgagcuagu..................................................................... 3 1 Mm2
..................uguacccuguagaaccgGgcuagu..................................................................... 187 1 Mm2
..................uguacccuguagaaccgaCcuagu..................................................................... 4 1 Mm2
..................uguacccuguagaaUcgagcuagu..................................................................... 8 1 Mm2
..................uguUcccuguagaaccgagcuagu..................................................................... 4 1 Mm2
..................uguacccuguUgaaccgagcuagu..................................................................... 50 1 Mm2
..................uguacccuguagaUccgagcuagu..................................................................... 13 1 Mm2
..................uguacccuguaCaaccgagcuagu..................................................................... 3 1 Mm2
..................uguacccuguagaCccgagcuagu..................................................................... 1 1 Mm2
..................uAuacccuguagaaccgagcuagu..................................................................... 34 1 Mm2
..................uguacccuguagaaccAagcuagu..................................................................... 162 1 Mm2
..................uguaccAuguagaaccgagcuagu..................................................................... 5 1 Mm2
..................Nguacccuguagaaccgagcuagu..................................................................... 38 1 Mm2
..................uguacccuguagUaccgagcuagu..................................................................... 9 1 Mm2
..................ugCacccuguagaaccgagcuagu..................................................................... 14 1 Mm2
..................uguaccNuguagaaccgagcuagu..................................................................... 3 1 Mm2
..................uguacccuguagaaccCagcuagu..................................................................... 132 1 Mm2
..................uguacccuguagaaccgagcuagC..................................................................... 28 1 Mm2
..................uguacccuguagaacUgagcuagu..................................................................... 27 1 Mm2
..................uguacccuguagaacGgagcuagu..................................................................... 2 1 Mm2
..................ugGacccuguagaaccgagcuagu..................................................................... 13 1 Mm2
..................uguacccuguaUaaccgagcuagu..................................................................... 14 1 Mm2
..................uguacccuguagaaccUagcuagu..................................................................... 135 1 Mm2
..................uguacccuguagaaccgagUuagu..................................................................... 43 1 Mm2
```

Mature **Star**

```
aaauguggugaguuucugug uacccuguagaaccgagcuagu guaucgaguucgaaacuca caagccauucuacaguguaca aagaaacgucgcuucuuucaacaaugaga
..................Aguacccuguagaaccgagcuagug....................................................................    1       1      Mm2
..................uguGcccuguagaaccgagcuagug....................................................................    1       1      Mm2
..................uguacccuguagaaccgagcuaguA....................................................................    55      1      Mm2
..................uguacccuguagaaccgGgcuagug....................................................................    3       1      Mm2
..................uguacccuguagaaccgUgcuagug....................................................................    3       1      Mm2
..................uguacccuguagaaccgagcuagAg....................................................................    1       1      Mm2
..................uguacccuguagaUccgagcuagug....................................................................    1       1      Mm2
..................uguacccuguagaaccgagcuagug....................................................................    1179    0      Mm2
..................uguacccuguagaaccCagcuagug....................................................................    2       1      Mm2
..................uguacccuguagaaccAagcuagug....................................................................    3       1      Mm2
..................uguacccuguCgaaccgagcuagug....................................................................    1       1      Mm2
..................uguacccuguagaaccgCgcuagug....................................................................    7       1      Mm2
..................uguacccuguagaaccUagcuagug....................................................................    4       1      Mm2
..................uguacccCguagaaccgagcuagug....................................................................    1       1      Mm2
..................uCuacccuguagaaccgagcuagug....................................................................    1       1      Mm2
..................ugCacccuguagaaccgagcuagug....................................................................    2       1      Mm2
..................uguacccuguagaaccgagcuaguC....................................................................    7       1      Mm2
..................uguacccuguUgaaccgagcuagug....................................................................    2       1      Mm2
..................uguacccuguagaaccgagcuaguU....................................................................    2469    1      Mm2
..................uguacUcuguagaaccgagcuagug....................................................................    1       1      Mm2
..................uUuacccuguagaaccgagcuagug....................................................................    1       1      Mm2
..................uguacccuguagaaccgagcuagugA...................................................................    40      1      Mm2
..................uguacccuguagaaccgagcuagugu...................................................................    1246    0      Mm2
..................uguacccuguUgaaccgagcuagugu...................................................................    3       1      Mm2
..................uguacccuguagaaccgagcuaguAu...................................................................    16      1      Mm2
..................uguaccUuguagaaccgagcuagugu...................................................................    1       1      Mm2
..................Nguacccuguagaaccgagcuagugu...................................................................    1       1      Mm2
..................uguacccuguagaaccgCgcuagugu...................................................................    5       1      Mm2
..................uguacccuguagaaccgGgcuagugu...................................................................    4       1      Mm2
..................uguacccuguagaaccgagcuagugC...................................................................    3       1      Mm2
..................uguacccuguagaaccAagcuagugu...................................................................    1       1      Mm2
..................uguacccuguagGaccgagcuagugu...................................................................    1       1      Mm2
..................Cguacccuguagaaccgagcuagugu...................................................................    1       1      Mm2
..................uguacccuguagaaccgagcuagugG...................................................................    94      1      Mm2
..................uAuacccuguagaaccgagcuagugu...................................................................    1       1      Mm2
..................uguacccuguagaaccgagcuaguCu...................................................................    2       1      Mm2
..................uguacccuguCgaaccgagcuagugu...................................................................    1       1      Mm2
..................uUuacccuguagaaccgagcuagugu...................................................................    1       1      Mm2
..................uguacNcuguagaaccgagcuagugu...................................................................    2       1      Mm2
..................Aguacccuguagaaccgagcuagugu...................................................................    1       1      Mm2
..................uguacccuguagaaccgagcuGgugu...................................................................    3       1      Mm2
..................uguacccuguagaaccgUgcuagugu...................................................................    8       1      Mm2
..................uguacccuguagaaccgagcuaguUu...................................................................    152     1      Mm2
..................uguacUcuguagaaccgagcuagugu...................................................................    1       1      Mm2
..................uguacccuguagaaccCagcuagugu...................................................................    3       1      Mm2
..................uguacccuguGgaaccgagcuagugu...................................................................    1       1      Mm2
..................uguacccuguagaaccgagcuagugua..................................................................    1       0      Mm2
..................uguacccuguagaaccgagcuaguguU..................................................................    51      1      Mm2
..................uguacccuguagaaccgagcuaguguau.................................................................    2       0      Mm2
..................uguacccuguagaaccgagcuaguguaA.................................................................    1       1      Mm2
..................uguacccuguagaaccgagcuaguguUu.................................................................    10      1      Mm2
..................uguacccuguagaaccgagcuaguguauc................................................................    1       0      Mm2
...................guacccuguagaaccgagU.........................................................................    5       1      Mm2
...................guacccuguagaaccgagc.........................................................................    25      0      Mm2
...................Uuacccuguagaaccgagc.........................................................................    4       1      Mm2
...................Nuacccuguagaaccgagc.........................................................................    6       1      Mm2
...................Cuacccuguagaaccgagc.........................................................................    1       1      Mm2
...................guacccuguagaaccgagcG........................................................................    1       1      Mm2
...................Auacccuguagaaccgagcu........................................................................    282     1      Mm2
...................guaccUuguagaaccgagcu........................................................................    1       1      Mm2
...................guacccuguagaaccgagcu........................................................................    130     0      Mm2
...................Cuacccuguagaaccgagcu........................................................................    6       1      Mm2
...................guacccuguagaaccgaAcu........................................................................    1       1      Mm2
...................guacccuguagaaccgagcua.......................................................................    20      0      Mm2
...................guacccuguagaaccgagcAa.......................................................................    1       1      Mm2
...................guacccuguagaaccgagcGa.......................................................................    9       1      Mm2
...................guacccuguagaaccgagcuU.......................................................................    3       1      Mm2
...................Auacccuguagaaccgagcua.......................................................................    14      1      Mm2
...................guacccuguaUaaccgagcuag......................................................................    1       1      Mm2
...................Cuacccuguagaaccgagcuag......................................................................    16      1      Mm2
```

Mature **Star**

aaauguggugaguuucugug**uacccuguagaaccgagcuagu**guaucgaguucgaaacuca**caagccauucuacaguguaca**aagaaacgucgcuucuuucaacaaugaga

...................guacccuguaCaaccgagcuag..	1	1	Mm2
...................Auacccuguagaaccgagcuag..	165	1	Mm2
...................guacccuguagaaccgUgcuag..	1	1	Mm2
...................guacccuguagaaccgaUcuag..	4	1	Mm2
...................guacccuguagaaccgagcGag..	21	1	Mm2
...................guacccuguagaaccgaAcuag..	1	1	Mm2
...................guacccuguagaaccgagcuaA..	1	1	Mm2
...................guacccuguagaaccgagUuag..	3	1	Mm2
...................guacccuguagaaccgagcuaU..	30	1	Mm2
...................guacccuguagaaccgagcuag..	814	0	Mm2
...................guacccuguagaaccgagcuGg..	1	1	Mm2
...................guacccuguagaaccgCgcuag..	4	1	Mm2
...................guGcccuguagaaccgagcuag..	1	1	Mm2
...................guacccuguagaaccgagcAag..	1	1	Mm2
...................guaccAuguagaaccgagcuag..	1	1	Mm2
...................guacccuguagaaccUagcuagu...	1	1	Mm2
...................guacccGguagaaccgagcuagu...	1	1	Mm2
...................gCacccuguagaaccgagcuagu...	2	1	Mm2
...................Auacccuguagaaccgagcuagu...	5383	1	Mm2
...................guaccUuguagaaccgagcuagu...	3	1	Mm2
...................guacccuguagaaccgagcuaUu...	2	1	Mm2
...................guacccNguagaaccgagcuagu...	1	1	Mm2
...................guaccAuguagaaccgagcuagu...	1	1	Mm2
...................guaGccuguagaaccgagcuagu...	1	1	Mm2
...................guacccuguagaacUgagcuagu...	7	1	Mm2
...................Cuacccuguagaaccgagcuagu...	483	1	Mm2
...................guacccuguagUaccgagcuagu...	5	1	Mm2
...................guacccuguCgaaccgagcuagu...	1	1	Mm2
...................guacccuguagaaccgagGuagu...	1	1	Mm2
...................guacccuguagaGccgagcuagu...	1	1	Mm2
...................guacccuguagaaccgagcCagu...	3	1	Mm2
...................guacccCguagaaccgagcuagu...	3	1	Mm2
...................guacccuguagaaccgagcuagC...	8	1	Mm2
...................guacccuguagaaccgaAcuagu...	89	1	Mm2
...................guacccuguagaUccgagcuagu...	2	1	Mm2
...................guacccuCuagaaccgagcuagu...	1	1	Mm2
...................guacccuguagaaccgaCcuagu...	91	1	Mm2
...................guacccuguagaacGgagcuagu...	1	1	Mm2
...................gAacccuguagaaccgagcuagu...	8	1	Mm2
...................guacccuguagaaccgagcuagA...	27	1	Mm2
...................guGcccuguagaaccgagcuagu...	2	1	Mm2
...................guacccuguagaaccgagcuagG...	29	1	Mm2
...................Uuacccuguagaaccgagcuagu...	28	1	Mm2
...................guacccuguUgaaccgagcuagu...	2	1	Mm2
...................guacccuguaUaaccgagcuagu...	18	1	Mm2
...................guacccuguagaaGcgagcuagu...	3	1	Mm2
...................guaAccuguagaaccgagcuagu...	1	1	Mm2
...................guacccuAuagaaccgagcuagu...	3	1	Mm2
...................guacGcuguagaaccgagcuagu...	2	1	Mm2
...................Nuacccuguagaaccgagcuagu...	7	1	Mm2
...................guacccuguagaaUcgagcuagu...	6	1	Mm2
...................guacccAguagaaccgagcuagu...	2	1	Mm2
...................guacccuguGgaaccgagcuagu...	3	1	Mm2
...................guacccuguagaaccgagcuaAu...	7	1	Mm2
...................guacccuguagaaccgagcuGgu...	45	1	Mm2
...................guacccuguagaaccgaUcuagu...	99	1	Mm2
...................guacccuguagaaccgagcuaCu...	1	1	Mm2
...................guCcccuguagaaccgagcuagu...	1	1	Mm2
...................guacccugAagaaccgagcuagu...	2	1	Mm2
...................guacccuguagaaccgGgcuagu...	7	1	Mm2
...................guacccuguagaaccgUgcuagu...	29	1	Mm2
...................guacccuguagaaccCagcuagu...	3	1	Mm2
...................guacAcuguagaaccgagcuagu...	3	1	Mm2
...................guacccugCagaaccgagcuagu...	2	1	Mm2
...................guacccuguagaaccAagcuagu...	2	1	Mm2
...................guaccNuguagaaccgagcuagu...	3	1	Mm2
...................guacccuguagaaccgagcGagu...	261	1	Mm2
...................guacccuguaCaaccgagcuagu...	15	1	Mm2
...................guacccuguagaaccgagUuagu...	7	1	Mm2
...................guacUcuguagaaccgagcuagu...	3	1	Mm2

```
                    Mature                                      Star
aaauguggugaguuucugugguacccuguagaaccgagcuaguguaucgaguucgaaacucacaagccauucuacaguguacaaagaaacgucgcuucuuucaacaaugaga
...................guacccuguagGaccgagcuagu.....................................................................  2      1   Mm2
...................guacccuguagaaAcgagcuagu.....................................................................  4      1   Mm2
...................guacccuguagaaccgagcuagu.....................................................................  18869  0   Mm2
...................guacccuguagaaccgagcuUgu.....................................................................  2      1   Mm2
...................guacccuguagaaccgCgcuagu.....................................................................  40     1   Mm2
...................guacccuguaAaaccgagcuagu.....................................................................  20     1   Mm2
...................guaUccuguagaaccgagcuagu.....................................................................  2      1   Mm2
...................guacccuguagaaccgaUcuagug....................................................................  5      1   Mm2
...................guacccuguagaaccgaCcuagug....................................................................  6      1   Mm2
...................guacccuguaAaaccgagcuagug....................................................................  2      1   Mm2
...................guacccuguagaaccgagcGagug....................................................................  3      1   Mm2
...................guaccUuguagaaccgagcuagug....................................................................  1      1   Mm2
...................guacccuguagaaccgagcuaguU....................................................................  739    1   Mm2
...................guacccuguagaaccgagcuaguA....................................................................  22     1   Mm2
...................guacccuguagaaccgagcuagCg....................................................................  1      1   Mm2
...................guacccuguagaacUgagcuagug....................................................................  1      1   Mm2
...................Cuacccuguagaaccgagcuagug....................................................................  16     1   Mm2
...................guacccuguagaaccgUgcuagug....................................................................  1      1   Mm2
...................guacGcuguagaaccgagcuagug....................................................................  1      1   Mm2
...................guCcccuguagaaccgagcuagug....................................................................  2      1   Mm2
...................guacccuguagaaccgGgcuagug....................................................................  1      1   Mm2
...................guacccuguagaaccgaAcuagug....................................................................  9      1   Mm2
...................guacccuguagaaccgagcuagug....................................................................  974    0   Mm2
...................guacccuguagaaGcgagcuagug....................................................................  1      1   Mm2
...................Auacccuguagaaccgagcuagug....................................................................  91     1   Mm2
...................guacccuguagaaccgCgcuagug....................................................................  2      1   Mm2
...................guacccuguagaaccgaUcuagugu...................................................................  3      1   Mm2
...................Cuacccuguagaaccgagcuagugu...................................................................  28     1   Mm2
...................Nuacccuguagaaccgagcuagugu...................................................................  1      1   Mm2
...................guacAcuguagaaccgagcuagugu...................................................................  1      1   Mm2
...................Auacccuguagaaccgagcuagugu...................................................................  51     1   Mm2
...................guacccuguagaaccgagcuagugA...................................................................  38     1   Mm2
...................guacccuguagaacUgagcuagugu...................................................................  2      1   Mm2
...................guacccuguagaaccgagcuaguUu...................................................................  45     1   Mm2
...................guacccuguagaaGcgagcuagugu...................................................................  2      1   Mm2
...................Uuacccuguagaaccgagcuagugu...................................................................  1      1   Mm2
...................guacccuguagaaccgagcuagugu...................................................................  842    0   Mm2
...................guacccuguagaaccgagcuGgugu...................................................................  5      1   Mm2
...................guacccuguagUaccgagcuagugu...................................................................  1      1   Mm2
...................guacccCguagaaccgagcuagugu...................................................................  1      1   Mm2
...................guacccuguaAaaccgagcuagugu...................................................................  2      1   Mm2
...................guacccuguagaaccgagcuagugC...................................................................  2      1   Mm2
...................guacccuguagaaccgagcuaguCu...................................................................  1      1   Mm2
...................guacccuguagaUccgagcuagugu...................................................................  1      1   Mm2
...................guacccuguagaaccgagcGagugu...................................................................  1      1   Mm2
...................guacccuguaUaaccgagcuagugu...................................................................  1      1   Mm2
...................guacccuguagaaccgagcuagCgu...................................................................  1      1   Mm2
...................guacccuguagaaccgagcuaguAu...................................................................  7      1   Mm2
...................guacccuguagaaccgaCcuagugu...................................................................  2      1   Mm2
...................guacccuguagaaccgagcuagugG...................................................................  84     1   Mm2
...................guacccuguagaaccgaAcuagugu...................................................................  1      1   Mm2
...................guacccuguagaaccgagcuaUugu...................................................................  1      1   Mm2
...................guacccuguagaaccgCgcuagugu...................................................................  5      1   Mm2
...................guacccuguagaaccgagcuagugua..................................................................  5      0   Mm2
...................guacccuguagaaccgagcuagugAa..................................................................  1      1   Mm2
...................guacccuguagaaccgagcuaguguU..................................................................  43     1   Mm2
...................guacccuguagaaccgagcuaguguUu.................................................................  6      1   Mm2
...................guacccuguagaaccgagcuaguguauc................................................................  4      0   Mm2
...................guacccuguagaaccgagcuaguguaucg...............................................................  1      0   Mm2
...................guacccuguagaaccgagcuaguguaucgaguu...........................................................  5      0   Mm2
....................uacGcuguagaaccgagc.........................................................................  1      1   Mm2
....................uacccuguagaaccgaUc.........................................................................  1      1   Mm2
....................uUcccuguagaaccgagc.........................................................................  1      1   Mm2
....................uacccuguagaaccgagc.........................................................................  1275   0   Mm2
....................uacccuguagaacUgagc.........................................................................  1      1   Mm2
....................uacccuguaUaaccgagc.........................................................................  1      1   Mm2
....................uacUcuguagaaccgagc.........................................................................  1      1   Mm2
....................uacccuguagaaccCagc.........................................................................  1      1   Mm2
....................uacccuguagaaccgagU.........................................................................  15     1   Mm2
....................uacccuguagaaccgUgc.........................................................................  2      1   Mm2
```

```
                   Mature                                  Star
aaauguggugaguuucugugguacccuguagaaccgagcuaguguaucgaguucgaaacucacaagccauucuacaguguacaaagaaacgucgcuucuuucaacaaugaga
....................uacccugCagaaccgagc.........................................................................   1      1   Mm2
....................uacccuguagaaccgagG.........................................................................   6      1   Mm2
....................Cacccuguagaaccgagc.........................................................................   1      1   Mm2
....................uacccuguagaaccgaCc.........................................................................   4      1   Mm2
....................uacccuguagaUccgagc.........................................................................   1      1   Mm2
....................uacccuguagaaGcgagc.........................................................................   1      1   Mm2
....................uacccuNuagaaccgagc.........................................................................   1      1   Mm2
....................uaUccuguagaaccgagc.........................................................................   1      1   Mm2
....................uaGccuguagaaccgagc.........................................................................   1      1   Mm2
....................uacccuguagaaccgagA.........................................................................   28     1   Mm2
....................uacccuguUgaaccgagc.........................................................................   1      1   Mm2
....................uacccuguUgaaccgagcu........................................................................   1      1   Mm2
....................uacAcuguagaaccgagcu........................................................................   2      1   Mm2
....................uacccuguagGaccgagcu........................................................................   5      1   Mm2
....................uacccuCuagaaccgagcu........................................................................   1      1   Mm2
....................uaGccuguagaaccgagcu........................................................................   1      1   Mm2
....................Aacccuguagaaccgagcu........................................................................   4      1   Mm2
....................uacccuguagaaccgagcC........................................................................   5      1   Mm2
....................uacccuguagaaUcgagcu........................................................................   2      1   Mm2
....................uacccuguagUaccgagcu........................................................................   7      1   Mm2
....................uacccuguGgaaccgagcu........................................................................   2      1   Mm2
....................uacccuguagaaccgGgcu........................................................................   1      1   Mm2
....................uacccuguagaaccgagcA........................................................................   25     1   Mm2
....................uacUcuguagaaccgagcu........................................................................   1      1   Mm2
....................uaccUuguagaaccgagcu........................................................................   1      1   Mm2
....................uacccuguagaaccgagUu........................................................................   27     1   Mm2
....................uacccuguagaaccgaCcu........................................................................   18     1   Mm2
....................uacccuguagaaccgaUcu........................................................................   16     1   Mm2
....................uacccuguagaacUgagcu........................................................................   1      1   Mm2
....................uacccuguaAaaccgagcu........................................................................   1      1   Mm2
....................uacccuguagaUccgagcu........................................................................   2      1   Mm2
....................uacccNguagaaccgagcu........................................................................   1      1   Mm2
....................Cacccuguagaaccgagcu........................................................................   1      1   Mm2
....................uacccuguagaaAcgagcu........................................................................   2      1   Mm2
....................Nacccuguagaaccgagcu........................................................................   2      1   Mm2
....................uacccuAuagaaccgagcu........................................................................   2      1   Mm2
....................uacGcuguagaaccgagcu........................................................................   1      1   Mm2
....................uacccuguagaaccgagAu........................................................................   55     1   Mm2
....................uacccuguagaaccgagGu........................................................................   12     1   Mm2
....................uUcccuguagaaccgagcu........................................................................   2      1   Mm2
....................uacccuguagaaccAagcu........................................................................   2      1   Mm2
....................uacccuguagaacAgagcu........................................................................   2      1   Mm2
....................uacccuguagCaccgagcu........................................................................   2      1   Mm2
....................uacccuguagaaccgagcu........................................................................   5405   0   Mm2
....................uacccuUuagaaccgagcu........................................................................   1      1   Mm2
....................uGcccuguagaaccgagcu........................................................................   2      1   Mm2
....................uCcccuguagaaccgagcu........................................................................   1      1   Mm2
....................uacccuguagaaccgaAcu........................................................................   17     1   Mm2
....................uacccuguagGaccgagcua.......................................................................   3      1   Mm2
....................Nacccuguagaaccgagcua.......................................................................   2      1   Mm2
....................uacccuguagaaccgaUcua.......................................................................   1      1   Mm2
....................uacccuguagaaUcgagcua.......................................................................   2      1   Mm2
....................uacccuguagaaccgagcua.......................................................................   1712   0   Mm2
....................uacccuguagaaccgagcCa.......................................................................   1      1   Mm2
....................uacccuguagaaccgagcuG.......................................................................   10     1   Mm2
....................uaAccuguagaaccgagcua.......................................................................   1      1   Mm2
....................uacccuguagaacUgagcua.......................................................................   1      1   Mm2
....................uacccuguagaaccgagcuU.......................................................................   297    1   Mm2
....................uacccuguagaaccgagAua.......................................................................   17     1   Mm2
....................uacccuguagaaccgagGua.......................................................................   7      1   Mm2
....................uacccuguagaaccgUgcua.......................................................................   1      1   Mm2
....................uaccUuguagaaccgagcua.......................................................................   1      1   Mm2
....................uacccuguagaaccgagUua.......................................................................   12     1   Mm2
....................uacccuguagaaccgaAcua.......................................................................   3      1   Mm2
....................uacccuguagaaccgaCcua.......................................................................   3      1   Mm2
....................uacccuguagaaccgagcAa.......................................................................   3      1   Mm2
....................uacccuguagaaccUagcua.......................................................................   1      1   Mm2
....................uacccuguagCaccgagcua.......................................................................   3      1   Mm2
....................uacccNguagaaccgagcua.......................................................................   1      1   Mm2
....................uacccuguagUaccgagcua.......................................................................   3      1   Mm2
```

```
                    Mature                      Star
aaauguggugaguuucuguguacccuguagaaccgagcuaguguaucgaguucgaaacucacaagccauucuacaguguacaaagaaacgucgcuucuuucaacaaugaga
....................uacccuNuagaaccgagcua.......................................................................  1      1  Mm2
....................uacccAguagaaccgagcua.......................................................................  1      1  Mm2
....................uacccugCagaaccgagcua.......................................................................  1      1  Mm2
....................uaccAuguagaaccgagcuag......................................................................  4      1  Mm2
....................uGcccuguagaaccgagcuag......................................................................  12     1  Mm2
....................Nacccuguagaaccgagcuag......................................................................  23     1  Mm2
....................uacccuguaCaaccgagcuag......................................................................  1      1  Mm2
....................uacccuguagaaccgagAuag......................................................................  497    1  Mm2
....................uacccuguagaGccgagcuag......................................................................  7      1  Mm2
....................uacccuguagaaccgGgcuag......................................................................  8      1  Mm2
....................uacccuguagaaccgUgcuag......................................................................  8      1  Mm2
....................uacccCguagaaccgagcuag......................................................................  8      1  Mm2
....................uacccugCagaaccgagcuag......................................................................  6      1  Mm2
....................Aacccuguagaaccgagcuag......................................................................  14     1  Mm2
....................uacccuAuagaaccgagcuag......................................................................  6      1  Mm2
....................uacccuguagCaccgagcuag......................................................................  32     1  Mm2
....................uacccuguagaaccgaAcuag......................................................................  116    1  Mm2
....................uacccuguagaaccAagcuag......................................................................  9      1  Mm2
....................uacccuUuagaaccgagcuag......................................................................  2      1  Mm2
....................uacccuguagaacUgagcuag......................................................................  13     1  Mm2
....................uacccuguagaaccgCgcuag......................................................................  2      1  Mm2
....................uaAccuguagaaccgagcuag......................................................................  13     1  Mm2
....................uacccuguagaaccgagUuag......................................................................  217    1  Mm2
....................uacccuguagaaccUagcuag......................................................................  3      1  Mm2
....................uacccuguagaaccgaUcuag......................................................................  100    1  Mm2
....................uacccGguagaaccgagcuag......................................................................  3      1  Mm2
....................uacccuguagaaccgaCcuag......................................................................  113    1  Mm2
....................uacccuguagaaccgagcuag......................................................................  51316  0  Mm2
....................uacccuguagaaccgagcuaU......................................................................  1347   1  Mm2
....................uacccuguagaaccgagcuCg......................................................................  2      1  Mm2
....................uacccuguagaaccCagcuag......................................................................  3      1  Mm2
....................uacccAguagaaccgagcuag......................................................................  1      1  Mm2
....................uacccuguagaCccgagcuag......................................................................  2      1  Mm2
....................uacccuguagaUccgagcuag......................................................................  7      1  Mm2
....................uacccuNuagaaccgagcuag......................................................................  3      1  Mm2
....................uaGccuguagaaccgagcuag......................................................................  6      1  Mm2
....................uacAcuguagaaccgagcuag......................................................................  20     1  Mm2
....................uacccuguagaaccgagcAag......................................................................  1      1  Mm2
....................uacccuguagaaccgagcuUg......................................................................  13     1  Mm2
....................uacccuCuagaaccgagcuag......................................................................  2      1  Mm2
....................uUcccuguagaaccgagcuag......................................................................  9      1  Mm2
....................uacccuguaAaccgagcuag.......................................................................  4      1  Mm2
....................uacUcuguagaaccgagcuag......................................................................  10     1  Mm2
....................uacccuguGgaaccgagcuag......................................................................  9      1  Mm2
....................uacGcuguagaaccgagcuag......................................................................  3      1  Mm2
....................uacccuguagUaccgagcuag......................................................................  48     1  Mm2
....................uacccuguagGaccgagcuag......................................................................  66     1  Mm2
....................uacccuguagaaccgagGuag......................................................................  167    1  Mm2
....................uacccuguagaacGgagcuag......................................................................  9      1  Mm2
....................uaccGuguagaaccgagcuag......................................................................  4      1  Mm2
....................uaUccuguagaaccgagcuag......................................................................  6      1  Mm2
....................Cacccuguagaaccgagcuag......................................................................  13     1  Mm2
....................uacccuguUgaaccgagcuag......................................................................  4      1  Mm2
....................uacccuguagaaccgagcuaC......................................................................  2      1  Mm2
....................uacccuguagaaccgagcuaA......................................................................  73     1  Mm2
....................uacccuguagaaccgagcuGg......................................................................  98     1  Mm2
....................uacccuguaUaaccgagcuag......................................................................  6      1  Mm2
....................uacccuguagaaUcgagcuag......................................................................  17     1  Mm2
....................uacccNguagaaccgagcuag......................................................................  7      1  Mm2
....................uaNccuguagaaccgagcuag......................................................................  1      1  Mm2
....................uacccuguagaaGcgagcuag......................................................................  2      1  Mm2
....................uacccuguagaaccgagcCag......................................................................  5      1  Mm2
....................uacccugAagaaccgagcuag......................................................................  7      1  Mm2
....................uaccUuguagaaccgagcuag......................................................................  7      1  Mm2
....................uacccuguagaacAgagcuag......................................................................  9      1  Mm2
....................uacccuguagaaccgagcuaAu.....................................................................  111    1  Mm2
....................uacccuguagaaccCagcuagu.....................................................................  41     1  Mm2
....................uacccuguagaaccAagcuagu.....................................................................  237    1  Mm2
....................uacccuUuagaaccgagcuagu.....................................................................  153    1  Mm2
....................uCcccuguagaaccgagcuagu.....................................................................  74     1  Mm2
```

```
                    Mature                                   Star
aaauguggugaguuucuguguacccuguagaaccgagcuaguguaucgaguucgaaacucacaagccauucuacaguguacaaagaaacgucgcuucuuucaacaaugaga
....................uacccuguagaCccgagcuagu.....................................................................  17       1   Mm2
....................uacccugCagaaccgagcuagu.....................................................................  196      1   Mm2
....................uacGcuguagaaccgagcuagu.....................................................................  87       1   Mm2
....................uacccuguagGaccgagcuagu.....................................................................  1641     1   Mm2
....................uacccuguagaaccgagcuagA.....................................................................  2059     1   Mm2
....................uNcccuguagaaccgagcuagu.....................................................................  11       1   Mm2
....................Cacccuguagaaccgagcuagu.....................................................................  263      1   Mm2
....................uacccuguagaaGcgagcuagu.....................................................................  28       1   Mm2
....................uacccuguUgaaccgagcuagu.....................................................................  179      1   Mm2
....................uaUccuguagaaccgagcuagu.....................................................................  201      1   Mm2
....................uacccuguagaaccgagcuagu.....................................................................  1466273  0   Mm2
....................uacccuguagaaccgagcuaUu.....................................................................  253      1   Mm2
....................uaAccuguagaaccgagcuagu.....................................................................  285      1   Mm2
....................uacccuguaCaaccgagcuagu.....................................................................  49       1   Mm2
....................uacccuguagaaccgUgcuagu.....................................................................  297      1   Mm2
....................Gacccuguagaaccgagcuagu.....................................................................  30       1   Mm2
....................uacccuguagaaccgagcAagu.....................................................................  48       1   Mm2
....................uacccuguagaaccgagcuagC.....................................................................  377      1   Mm2
....................uacccuguagaaccgagAuagu.....................................................................  13695    1   Mm2
....................uUcccuguagaaccgagcuagu.....................................................................  279      1   Mm2
....................uacccAguagaaccgagcuagu.....................................................................  67       1   Mm2
....................uacccuCuagaaccgagcuagu.....................................................................  53       1   Mm2
....................uacccuguagaaccgagGuagu.....................................................................  5004     1   Mm2
....................uacccuguagaaccgagcuUgu.....................................................................  899      1   Mm2
....................uaNccuguagaaccgagcuagu.....................................................................  6        1   Mm2
....................uacccuguagaUccgagcuagu.....................................................................  431      1   Mm2
....................uacccuguCgaaccgagcuagu.....................................................................  30       1   Mm2
....................uacccuAuagaaccgagcuagu.....................................................................  128      1   Mm2
....................uaccGuguagaaccgagcuagu.....................................................................  56       1   Mm2
....................uacccuguagaaAcgagcuagu.....................................................................  36       1   Mm2
....................Nacccuguagaaccgagcuagu.....................................................................  717      1   Mm2
....................uacccCguagaaccgagcuagu.....................................................................  190      1   Mm2
....................uacccGguagaaccgagcuagu.....................................................................  72       1   Mm2
....................uacccuguagaaccgagUuagu.....................................................................  7297     1   Mm2
....................uacccuguagaaccgGgcuagu.....................................................................  177      1   Mm2
....................uaccAuguagaaccgagcuagu.....................................................................  111      1   Mm2
....................uaGccuguagaaccgagcuagu.....................................................................  259      1   Mm2
....................uacccuguagaaccgagcCagu.....................................................................  139      1   Mm2
....................uacccuguagaaccgaUcuagu.....................................................................  2508     1   Mm2
....................uacccuguagCaccgagcuagu.....................................................................  1365     1   Mm2
....................Aacccuguagaaccgagcuagu.....................................................................  351      1   Mm2
....................uGcccuguagaaccgagcuagu.....................................................................  298      1   Mm2
....................uacccugAagaaccgagcuagu.....................................................................  95       1   Mm2
....................uacccuguaUaaccgagcuagu.....................................................................  170      1   Mm2
....................uacccuguagaaccgaAcuagu.....................................................................  2978     1   Mm2
....................uacUcuguagaaccgagcuagu.....................................................................  279      1   Mm2
....................uacccuguagaacGgagcuagu.....................................................................  69       1   Mm2
....................uacccuguagUaccgagcuagu.....................................................................  1433     1   Mm2
....................uacccuguagaaccgCgcuagu.....................................................................  68       1   Mm2
....................uacccuguaAaaccgagcuagu.....................................................................  101      1   Mm2
....................uacccuNuagaaccgagcuagu.....................................................................  69       1   Mm2
....................uacccuguagaaccgaCcuagu.....................................................................  3238     1   Mm2
....................uacccuguagaaUcgagcuagu.....................................................................  270      1   Mm2
....................uacccuguagaaccgagcuGgu.....................................................................  1857     1   Mm2
....................uacccuguagaaccgagNuagu.....................................................................  2        1   Mm2
....................uaccUuguagaaccgagcuagu.....................................................................  207      1   Mm2
....................uacccuguagaacUgagcuagu.....................................................................  417      1   Mm2
....................uacccuguagaaccUagcuagu.....................................................................  113      1   Mm2
....................uacccNguagaaccgagcuagu.....................................................................  235      1   Mm2
....................uacccuguagaaccgagcuaCu.....................................................................  41       1   Mm2
....................uacccuguagaGccgagcuagu.....................................................................  171      1   Mm2
....................uacccuguagaaccgagcuCgu.....................................................................  95       1   Mm2
....................uacAcuguagaaccgagcuagu.....................................................................  439      1   Mm2
....................uacccuguGgaaccgagcuagu.....................................................................  191      1   Mm2
....................uacccuguagaacAgagcuagu.....................................................................  263      1   Mm2
....................uacccugGagaaccgagcuagu.....................................................................  43       1   Mm2
....................uacccuguagaaccgagcuagG.....................................................................  2246     1   Mm2
....................uacccuguagaaccgGgcuagug....................................................................  3        1   Mm2
....................uacccuguagaaccgagcuagug....................................................................  46557    0   Mm2
....................uaNccuguagaaccgagcuagug....................................................................  1        1   Mm2
```

Mature **Star**

```
aaauguggugaguuucugug uacccuguagaaccgagcuagu guaucgaguucgaaacuca caagccauucuacaguguaca aagaaacgucgcuucuuucaacaaugaga
```

```
aaauguggugaguuucuguguacccuguagaaccgagcuaguguaucgaguucgaaacucacaagccauucuacaguguacaaagaaacgucgcuucuuucaacaaugaga
....................uacccuguagaaccgagGuagug....................................................................  164     1   Mm2
....................uacUcuguagaaccgagcuagug....................................................................  11      1   Mm2
....................uaUccuguagaaccgagcuagug....................................................................  6       1   Mm2
....................uacccuguagaaccgagcuCgug....................................................................  1       1   Mm2
....................uGcccuguagaaccgagcuagug....................................................................  18      1   Mm2
....................uacccuNuagaaccgagcuagug....................................................................  2       1   Mm2
....................uacccuguagaaGcgagcuagug....................................................................  1       1   Mm2
....................uacccuguagaaUcgagcuagug....................................................................  4       1   Mm2
....................uacccuguagaaccgagUuagug....................................................................  215     1   Mm2
....................uacccuguagaaccgagcuaUug....................................................................  5       1   Mm2
....................uacccuguagaacUgagcuagug....................................................................  9       1   Mm2
....................uacccugAagaaccgagcuagug....................................................................  2       1   Mm2
....................uaccUuguagaaccgagcuagug....................................................................  4       1   Mm2
....................uacccuguagaaccUagcuagug....................................................................  2       1   Mm2
....................uacccuguagaaccgagcCagug....................................................................  2       1   Mm2
....................uacccAguagaaccgagcuagug....................................................................  3       1   Mm2
....................uacccGguagaaccgagcuagug....................................................................  2       1   Mm2
....................uacccuguagaaccgagcuagCg....................................................................  8       1   Mm2
....................uacccuguUgaaccgagcuagug....................................................................  12      1   Mm2
....................uacccuguagaaAcgagcuagug....................................................................  2       1   Mm2
....................uacAcuguagaaccgagcuagug....................................................................  16      1   Mm2
....................uaccAuguagaaccgagcuagug....................................................................  8       1   Mm2
....................uacccNguagaaccgagcuagug....................................................................  5       1   Mm2
....................uacccuguagaacGgagcuagug....................................................................  1       1   Mm2
....................uaGccuguagaaccgagcuagug....................................................................  12      1   Mm2
....................Gacccuguagaaccgagcuagug....................................................................  1       1   Mm2
....................uaAccuguagaaccgagcuagug....................................................................  8       1   Mm2
....................uacccugGagaaccgagcuagug....................................................................  1       1   Mm2
....................uacccuguagaaccgagcuagAg....................................................................  6       1   Mm2
....................uacccuguagUaccgagcuagug....................................................................  54      1   Mm2
....................uaccGuguagaaccgagcuagug....................................................................  2       1   Mm2
....................uacccuguagaacAgagcuagug....................................................................  10      1   Mm2
....................uacccuguagaaccgagcuagGg....................................................................  13      1   Mm2
....................uacccuguagaaccgUgcuagug....................................................................  9       1   Mm2
....................uacccuCuagaaccgagcuagug....................................................................  2       1   Mm2
....................uCcccuguagaaccgagcuagug....................................................................  1       1   Mm2
....................uacccuguagGaccgagcuagug....................................................................  56      1   Mm2
....................uacccuguagaaccgagNuagug....................................................................  1       1   Mm2
....................Aacccuguagaaccgagcuagug....................................................................  18      1   Mm2
....................uUcccuguagaaccgagcuagug....................................................................  4       1   Mm2
....................uacccuguagCaccgagcuagug....................................................................  53      1   Mm2
....................uacccCguagaaccgagcuagug....................................................................  4       1   Mm2
....................uacccuguaAaaccgagcuagug....................................................................  3       1   Mm2
....................uacccuguagaGccgagcuagug....................................................................  5       1   Mm2
....................Nacccuguagaaccgagcuagug....................................................................  22      1   Mm2
....................uacccuguagaaccAagcuagug....................................................................  11      1   Mm2
....................uacccuguagaUccgagcuagug....................................................................  11      1   Mm2
....................uacccuguCgaaccgagcuagug....................................................................  1       1   Mm2
....................uacccuguaUaaccgagcuagug....................................................................  7       1   Mm2
....................uacccuUuagaaccgagcuagug....................................................................  2       1   Mm2
....................uacccuguagaaccgagAuagug....................................................................  482     1   Mm2
....................uacccuguGgaaccgagcuagug....................................................................  8       1   Mm2
....................uacccuguagaaccgagcuGgug....................................................................  85      1   Mm2
....................uacccuguagaaccgCgcuagug....................................................................  3       1   Mm2
....................uacccuguagaaccgaCcuagug....................................................................  96      1   Mm2
....................uacGcuguagaaccgagcuagug....................................................................  5       1   Mm2
....................uacccuguagaaccgagcAagug....................................................................  1       1   Mm2
....................uacccuAuagaaccgagcuagug....................................................................  1       1   Mm2
....................uacccuguagaaccgagcuaguU....................................................................  15502   1   Mm2
....................uacccuguagaaccgagcuUgug....................................................................  7       1   Mm2
....................uacccuguaCaaccgagcuagug....................................................................  2       1   Mm2
....................Cacccuguagaaccgagcuagug....................................................................  8       1   Mm2
....................uacccugCagaaccgagcuagug....................................................................  4       1   Mm2
....................uacccuguagaaccgaAcuagug....................................................................  89      1   Mm2
....................uacccuguagaaccgagcuaguA....................................................................  2637    1   Mm2
....................uacccuguagaaccCagcuagug....................................................................  2       1   Mm2
....................uacccuguagaaccgagcuaguC....................................................................  38      1   Mm2
....................uacccuguagaaccgaUcuagug....................................................................  83      1   Mm2
....................uacccugAagaaccgagcuagugu...................................................................  5       1   Mm2
....................uacccuguagaaccgagcuaUugu...................................................................  1       1   Mm2
```

```
                    Mature                                    Star
aaauguggugaguuucugugA uacccuguagaaccgagcuaguguaucgaguucgaaacucacaagccauucuacaguguacaaagaaacgucgcuucuuucaacaaugaga
```

```
                    Mature                                    Star
aaauguggugaguuucuguguacccuguagaaccgagcuaguguaucgaguucgaaacucacaagccauucuacaguguacaaagaaacgucgcuucuuucaacaaugaga
....................uacGcuguagaaccgagcuagugu...................................................................   6       1   Mm2
....................uacccuguagaaGcgagcuagugu...................................................................   2       1   Mm2
....................uaUccuguagaaccgagcuagugu...................................................................   11      1   Mm2
....................uacccuguagaaAcgagcuagugu...................................................................   1       1   Mm2
....................uacccuguagaaccgagcCagugu...................................................................   5       1   Mm2
....................uacccuguagaaccgagcuaAugu...................................................................   4       1   Mm2
....................uacccuguagaaccgagUuagugu...................................................................   324     1   Mm2
....................uacccuguagaaccgUgcuagugu...................................................................   4       1   Mm2
....................uacccCguagaaccgagcuagugu...................................................................   5       1   Mm2
....................uacccuguagaaccgagcuGgugu...................................................................   116     1   Mm2
....................uacccuguagaGccgagcuagugu...................................................................   5       1   Mm2
....................uacccuguagUaccgagcuagugu...................................................................   68      1   Mm2
....................uacccuguaCaaccgagcuagugu...................................................................   2       1   Mm2
....................uacccuguagaaccgCgcuagugu...................................................................   1       1   Mm2
....................uacccuguagaaccgagcuaguUu...................................................................   2174    1   Mm2
....................uacccuguagaaccgagcuagGgu...................................................................   2       1   Mm2
....................uacccuguagaaccgagcuaguCu...................................................................   17      1   Mm2
....................uacccuNuagaaccgagcuagugu...................................................................   3       1   Mm2
....................uacccuguGgaaccgagcuagugu...................................................................   16      1   Mm2
....................uacccuguagaaccgagcuaCugu...................................................................   1       1   Mm2
....................uacccuguagCaccgagcuagugu...................................................................   78      1   Mm2
....................uacccuCuagaaccgagcuagugu...................................................................   3       1   Mm2
....................uacccuguagaaccUagcuagugu...................................................................   3       1   Mm2
....................uacccuguagaaccgagcGagugu...................................................................   46      1   Mm2
....................uacccNguagaaccgagcuagugu...................................................................   13      1   Mm2
....................Aacccuguagaaccgagcuagugu...................................................................   15      1   Mm2
....................Cacccuguagaaccgagcuagugu...................................................................   9       1   Mm2
....................uacccugCagaaccgagcuagugu...................................................................   10      1   Mm2
....................uacccuguagaUccgagcuagugu...................................................................   10      1   Mm2
....................uacccuguagGaccgagcuagugu...................................................................   78      1   Mm2
....................uacccuguagaaccgagcuagugu...................................................................   66986   0   Mm2
....................uacccuUuagaaccgagcuagugu...................................................................   5       1   Mm2
....................uacccuguagaaccgagcuagAgu...................................................................   6       1   Mm2
....................uacccuguagaaccgaUcuagugu...................................................................   111     1   Mm2
....................uacccugGagaaccgagcuagugu...................................................................   3       1   Mm2
....................uUcccuguagaaccgagcuagugu...................................................................   19      1   Mm2
....................uGcccuguagaaccgagcuagugu...................................................................   18      1   Mm2
....................uaccAuguagaaccgagcuagugu...................................................................   8       1   Mm2
....................uacccuguagaaccgagcuCgugu...................................................................   3       1   Mm2
....................uacccuguagaaccCagcuagugu...................................................................   1       1   Mm2
....................uCcccuguagaaccgagcuagugu...................................................................   4       1   Mm2
....................uacccuguagaaUcgagcuagugu...................................................................   7       1   Mm2
....................uacccuguCgaaccgagcuagugu...................................................................   3       1   Mm2
....................uaccGuguagaaccgagcuagugu...................................................................   1       1   Mm2
....................uaccUuguagaaccgagcuagugu...................................................................   9       1   Mm2
....................uacccuguaUaaccgagcuagugu...................................................................   5       1   Mm2
....................uacccuguagaCccgagcuagugu...................................................................   1       1   Mm2
....................uacccuguagaacAgagcuagugu...................................................................   12      1   Mm2
....................uacccuguagaaccgagcuagugC...................................................................   94      1   Mm2
....................uaAccuguagaaccgagcuagugu...................................................................   14      1   Mm2
....................uacccuguUgaaccgagcuagugu...................................................................   12      1   Mm2
....................uacAcuguagaaccgagcuagugu...................................................................   10      1   Mm2
....................uacccuguagaaccgagcuaguAu...................................................................   1172    1   Mm2
....................uacccuguagaaccgagAuagugu...................................................................   663     1   Mm2
....................uacccuguagaaccgagcuUgugu...................................................................   13      1   Mm2
....................uacccuguagaacGgagcuagugu...................................................................   1       1   Mm2
....................uacccuguagaaccgaAcuagugu...................................................................   125     1   Mm2
....................uacccuguagaaccgagcuagugA...................................................................   2072    1   Mm2
....................uacccuguagaaccgaCcuagugu...................................................................   128     1   Mm2
....................Nacccuguagaaccgagcuagugu...................................................................   24      1   Mm2
....................uacccuguagaaccAagcuagugu...................................................................   14      1   Mm2
....................uacccAguagaaccgagcuagugu...................................................................   5       1   Mm2
....................uacUcuguagaaccgagcuagugu...................................................................   13      1   Mm2
....................uacccuguagaaccgagcAagugu...................................................................   3       1   Mm2
....................uacccuguagaacUgagcuagugu...................................................................   16      1   Mm2
....................uacccuguagaaccgagcuagCgu...................................................................   13      1   Mm2
....................uacccuguagaaccgagcuagugG...................................................................   5782    1   Mm2
....................uacccuguaAaaccgagcuagugu...................................................................   5       1   Mm2
....................uaGccuguagaaccgagcuagugu...................................................................   17      1   Mm2
....................uacccuAuagaaccgagcuagugu...................................................................   8       1   Mm2
```

Mature Star

```
aaauguggugaguuucugugguacccuguagaaccgagcuaguguaucgaguucgaaacucacaagccauucuacaguguacaaagaaacgucgcuucuuucaacaaugaga
....................uacccuguagaaccgGgcuagugu...................................................................   2     1   Mm2
....................uacccuguagaaccgagGuagugu...................................................................   245   1   Mm2
....................uacccuguagaaccgagcuagugua..................................................................   43    0   Mm2
....................uacccuguagaaccgagcuaguAua..................................................................   1     1   Mm2
....................uacccuguagaaccgagcuaguguG..................................................................   1     1   Mm2
....................uacccuguagaaccgagcuagugAa..................................................................   27    1   Mm2
....................uacccuguagaaccgagcuaguUua..................................................................   4     1   Mm2
....................uacccuguagaaccgagcuaguguU..................................................................   1444  1   Mm2
....................uacccuguagaaccgagcuaguguC..................................................................   3     1   Mm2
....................uacccuguagaaccgagAuaguguau.................................................................   1     1   Mm2
....................uacccuguagaaccgagcuaguguaA.................................................................   1     1   Mm2
....................uacccuguagaaccgagcuaguguau.................................................................   40    0   Mm2
....................uacccuguagaaccgagcuaguguUu.................................................................   82    1   Mm2
....................uacccuguagaaccgagcuaguguCu.................................................................   2     1   Mm2
....................uacccuguagaaccgagcuagugAau.................................................................   1     1   Mm2
....................uacccuguUgaaccgagcuaguguauc................................................................   1     1   Mm2
....................uacccugCagaaccgagcuaguguauc................................................................   1     1   Mm2
....................uacccuguagaaccgagAuaguguauc................................................................   1     1   Mm2
....................uacccuguagaaccgagcuagugGauc................................................................   1     1   Mm2
....................uacccuguagaaccgagcuaguguauU................................................................   2     1   Mm2
....................uacccuguagaaccgagcuaguguauc................................................................   71    0   Mm2
....................uacccuguagaaccgagcuaguguaucg...............................................................   12    0   Mm2
....................uacccuguagaaccgagcuaguguaucgag.............................................................   2     0   Mm2
....................uacccuguagaaccgagcuaguguaucgaguu...........................................................   1     0   Mm2
.....................acccuguagaaccgagcu........................................................................   31    0   Mm2
.....................acccuguagaCccgagcua.......................................................................   1     1   Mm2
.....................acccuguagaaccgagcCa.......................................................................   1     1   Mm2
.....................acccuguagaaccgagcua.......................................................................   4     0   Mm2
.....................acccuguagaaccgagcuU.......................................................................   2     1   Mm2
.....................acccuguagaaccgagcuaC......................................................................   1     1   Mm2
.....................acccuguagaaccgagcuUg......................................................................   1     1   Mm2
.....................acccuguagaaccgaAcuag......................................................................   1     1   Mm2
.....................acccuguagaaccgagcuCg......................................................................   2     1   Mm2
.....................acccuguaAaaccgagcuag......................................................................   2     1   Mm2
.....................acccuguagaaccgagcAag......................................................................   2     1   Mm2
.....................acccuCuagaaccgagcuag......................................................................   1     1   Mm2
.....................acccuguagaaccgagcCag......................................................................   1     1   Mm2
.....................acccuguagaaccgagcuGg......................................................................   1     1   Mm2
.....................acccuguagaacAgagcuag......................................................................   2     1   Mm2
.....................acccuguagaCccgagcuag......................................................................   1     1   Mm2
.....................acccuguaUaaccgagcuag......................................................................   1     1   Mm2
.....................acccuguagaGccgagcuag......................................................................   1     1   Mm2
.....................acccuguagaaccgagcuaU......................................................................   6     1   Mm2
.....................acccuguagaaccgagcuag......................................................................   279   0   Mm2
.....................acccuguagaaUcgagcuag......................................................................   1     1   Mm2
.....................acUcuguagaaccgagcuag......................................................................   1     1   Mm2
.....................Ncccuguagaaccgagcuag......................................................................   1     1   Mm2
.....................accAuguagaaccgagcuagu.....................................................................   3     1   Mm2
.....................acccuguagaaccgagcuagC.....................................................................   4     1   Mm2
.....................acccuguagaGccgagcuagu.....................................................................   13    1   Mm2
.....................acccuguagaaccgaAcuagu.....................................................................   4     1   Mm2
.....................acAcuguagaaccgagcuagu.....................................................................   2     1   Mm2
.....................acccuguagaaccgagcuaUu.....................................................................   10    1   Mm2
.....................acccuguagaaccgagcuUgu.....................................................................   13    1   Mm2
.....................acccuguagaaccgaCcuagu.....................................................................   1     1   Mm2
.....................acccuguagaaccgagcAagu.....................................................................   24    1   Mm2
.....................acccuguagaaccgagcuaAu.....................................................................   6     1   Mm2
.....................Ucccuguagaaccgagcuagu.....................................................................   5     1   Mm2
.....................acccuguagaaUcgagcuagu.....................................................................   5     1   Mm2
.....................acccuguagaCccgagcuagu.....................................................................   14    1   Mm2
.....................acccAguagaaccgagcuagu.....................................................................   2     1   Mm2
.....................acccuguagaaccgagcuGgu.....................................................................   13    1   Mm2
.....................acccuguagaaccgagcuagA.....................................................................   12    1   Mm2
.....................acccuguagaaccgagUuagu.....................................................................   6     1   Mm2
.....................acccuUuagaaccgagcuagu.....................................................................   2     1   Mm2
.....................acccuguagaaAcgagcuagu.....................................................................   3     1   Mm2
.....................acccuguagaaccgGgcuagu.....................................................................   1     1   Mm2
.....................acccuguagaacUgagcuagu.....................................................................   3     1   Mm2
.....................acccuguagaaccgaUcuagu.....................................................................   4     1   Mm2
.....................acccuguagaacAgagcuagu.....................................................................   5     1   Mm2
```

Mature **Star**

```
aaauguggugaguuucugugcuacccuguagaaccgagcuaguguaucgaguucgaaacucacaagccauucuacaguguacaaagaaacgucgcuucuuucaacaaugaga
.....................acccuCuagaaccgagcuagu.....................................................................   3     1  Mm2
.....................acccuguCgaaccgagcuagu.....................................................................   3     1  Mm2
.....................acccuguagaaccgCgcuagu.....................................................................   6     1  Mm2
.....................acUcuguagaaccgagcuagu.....................................................................   4     1  Mm2
.....................acccuguagaacGgagcuagu.....................................................................   3     1  Mm2
.....................acccuguagaaccgagAuagu.....................................................................   9     1  Mm2
.....................acccugAagaaccgagcuagu.....................................................................   1     1  Mm2
.....................acccCguagaaccgagcuagu.....................................................................   3     1  Mm2
.....................acccuguagCaccgagcuagu.....................................................................   2     1  Mm2
.....................acccuguagGaccgagcuagu.....................................................................   1     1  Mm2
.....................acccGguagaaccgagcuagu.....................................................................   1     1  Mm2
.....................acccugGagaaccgagcuagu.....................................................................   1     1  Mm2
.....................acccuguagUaccgagcuagu.....................................................................   1     1  Mm2
.....................acccuguagaaccgUgcuagu.....................................................................   1     1  Mm2
.....................acccuguagaaccAagcuagu.....................................................................   7     1  Mm2
.....................acccugNagaaccgagcuagu.....................................................................   1     1  Mm2
.....................acccuguagaUccgagcuagu.....................................................................   13    1  Mm2
.....................acccuguagaaccgagcuaCu.....................................................................   4     1  Mm2
.....................acccuguagaaccUagcuagu.....................................................................   3     1  Mm2
.....................acccuguagaaccgagcuagu.....................................................................   7197  0  Mm2
.....................acGcuguagaaccgagcuagu.....................................................................   3     1  Mm2
.....................acccuguagaaccgagcuCgu.....................................................................   21    1  Mm2
.....................acccuguaUaaccgagcuagu.....................................................................   3     1  Mm2
.....................aAccuguagaaccgagcuagu.....................................................................   6     1  Mm2
.....................acccuguagaaccgagcuagG.....................................................................   14    1  Mm2
.....................acccuguaAaaccgagcuagu.....................................................................   6     1  Mm2
.....................acccuguaCaaccgagcuagu.....................................................................   8     1  Mm2
.....................acccuguagaaccgagcCagu.....................................................................   41    1  Mm2
.....................acccuAuagaaccgagcuagu.....................................................................   3     1  Mm2
.....................Gcccuguagaaccgagcuagu.....................................................................   1     1  Mm2
.....................acccuguGgaaccgagcuagu.....................................................................   1     1  Mm2
.....................Ncccuguagaaccgagcuagu.....................................................................   2     1  Mm2
.....................acccuguagaaccgagcuCgug....................................................................   1     1  Mm2
.....................acccuguCgaaccgagcuagug....................................................................   1     1  Mm2
.....................acccuguagaacAgagcuagug....................................................................   2     1  Mm2
.....................acccuguagaaccgagcuaguA....................................................................   14    1  Mm2
.....................acccuguagaaccgagcuGgug....................................................................   2     1  Mm2
.....................accUuguagaaccgagcuagug....................................................................   2     1  Mm2
.....................acccuguagaGccgagcuagug....................................................................   1     1  Mm2
.....................acccuguagaaccgagcAagug....................................................................   2     1  Mm2
.....................aUccuguagaaccgagcuagug....................................................................   1     1  Mm2
.....................acccuguagaaccgagcuaguU....................................................................   77    1  Mm2
.....................acccuNuagaaccgagcuagug....................................................................   1     1  Mm2
.....................acAcuguagaaccgagcuagug....................................................................   1     1  Mm2
.....................acccuguagaaccgagcuagug....................................................................   318   0  Mm2
.....................acUcuguagaaccgagcuagug....................................................................   1     1  Mm2
.....................acccuguagaaccgagcCagug....................................................................   2     1  Mm2
.....................acccuguUgaaccgagcuagug....................................................................   1     1  Mm2
.....................acccuguagaaccgagcuagugu...................................................................   630   0  Mm2
.....................Gcccuguagaaccgagcuagugu...................................................................   1     1  Mm2
.....................acccuguaUaaccgagcuagugu...................................................................   1     1  Mm2
.....................acccuguagaaccgagcAagugu...................................................................   3     1  Mm2
.....................Ncccuguagaaccgagcuagugu...................................................................   1     1  Mm2
.....................acccuguagaaccgagcuagugA...................................................................   32    1  Mm2
.....................acccuguagaaccgagcuagugG...................................................................   50    1  Mm2
.....................acccuguagaaccAagcuagugu...................................................................   2     1  Mm2
.....................acccuguagaaccgagcCagugu...................................................................   9     1  Mm2
.....................acccuguagaaccgagcuaguUu...................................................................   14    1  Mm2
.....................acccuguagaaccgaAcuagugu...................................................................   1     1  Mm2
.....................acccuguagaaccgagcuCgugu...................................................................   1     1  Mm2
.....................acccuguagaaccgagcGagugu...................................................................   8     1  Mm2
.....................acccuguGgaaccgagcuagugu...................................................................   1     1  Mm2
.....................acccuguagaaccgagcuaguAu...................................................................   4     1  Mm2
.....................acccuguagaaccgagcuGgugu...................................................................   1     1  Mm2
.....................acccuguagaaccgagAuagugu...................................................................   2     1  Mm2
.....................acccuguagaaccgagUuagugu...................................................................   1     1  Mm2
.....................acccuguagaaccgagcuagugua..................................................................   2     0  Mm2
.....................acccuguagaaccgagcuaguguU..................................................................   23    1  Mm2
.....................acccuguagaaccgagcuaguguG..................................................................   1     1  Mm2
.....................acccuguagaaccgagcuaguguauc................................................................   3     0  Mm2
```

Mature **Star**

```
aaauguggugaguuucugugacccuguagaaccgagcuaguguaucgaguucgaaacucacaagccauucuacaguguacaaagaaacgucgcuucuuucaacaaugaga
```

```
.....................acccuguagaaccgagcuaguguaucg...............................................................   4      0   Mm2
.....................acccuguagaaccgagcuaguguaucgag.............................................................   1      0   Mm2
.....................acccuguagaaccgagcAaguguaucgag.............................................................   1      1   Mm2
.....................acccuguagaaccgagcuaguguaucgagu............................................................   5      0   Mm2
.....................acccuguagaaccgagcuaguguaucgaguucgaaacucacaagccauuc........................................   1      0   Mm2
......................cccuguagaaccgagcuag......................................................................   5      0   Mm2
......................cccuguagaaccgagcuGgu.....................................................................   3      1   Mm2
......................ccAuguagaaccgagcuagu.....................................................................   1      1   Mm2
......................cccuguagaaccgaCcuagu.....................................................................   2      1   Mm2
......................cccuguagaaccgGgcuagu.....................................................................   1      1   Mm2
......................ccUuguagaaccgagcuagu.....................................................................   1      1   Mm2
......................cccuguagaaccgagcuCgu.....................................................................   1      1   Mm2
......................cccuguagaaccgagcuagu.....................................................................   155    0   Mm2
......................cccuguagaaccgagcuUgu.....................................................................   3      1   Mm2
......................cccuguagaaccgagcuaguU....................................................................   3      1   Mm2
......................cccuguagaaccgagcuagug....................................................................   6      0   Mm2
......................cccuguagaaccgagcuaguUu...................................................................   1      1   Mm2
......................cccuguagaaccgagcuagugG...................................................................   2      1   Mm2
......................cccuguagaaccgagcuagugu...................................................................   11     0   Mm2
.......................ccuguagaaccgagcuagu.....................................................................   13     0   Mm2
.......................ccuguagaacUgagcuagu.....................................................................   1      1   Mm2
.......................ccuguagaaccgagcuUgu.....................................................................   1      1   Mm2
.......................ccuguagaaccgagcuaguU....................................................................   2      1   Mm2
.......................ccuguagaaccgagcuagugu...................................................................   1      0   Mm2
........................cCguagaaccgagcuagu.....................................................................   1      1   Mm2
.........................uguagaaccgagcGagugu...................................................................   1      1   Mm2
..........................................................ucacaagccauucuacagugu................................   1      0   Mm2
.............................................................caagccauucuacagugA................................   1      1   Mm2
.............................................................caagccauucuacagugu................................   52     0   Mm2
.............................................................caagccauucuacagugG................................   8      1   Mm2
.............................................................caagccauucuacagugua...............................   17     0   Mm2
.............................................................caagccauucuacaguguU...............................   2      1   Mm2
.............................................................caagccauucuacagugCac..............................   4      1   Mm2
.............................................................cGagccauucuacaguguac..............................   1      1   Mm2
.............................................................caagccauucuacaUuguac..............................   1      1   Mm2
.............................................................Naagccauucuacaguguac..............................   1      1   Mm2
.............................................................caCgccauucuacaguguac..............................   1      1   Mm2
.............................................................caagccauucCacaguguac..............................   1      1   Mm2
.............................................................caagccauucuacaguUuac..............................   1      1   Mm2
.............................................................caagccauucuacagugAac..............................   2      1   Mm2
.............................................................caagccauucGacaguguac..............................   2      1   Mm2
.............................................................caagccauucuacaguguaA..............................   1      1   Mm2
.............................................................caagccauucuacaguguac..............................   1036   0   Mm2
.............................................................caagccauucuacaguAuac..............................   2      1   Mm2
.............................................................Uaagccauucuacaguguac..............................   3      1   Mm2
.............................................................caagccauucuacaguCuac..............................   4      1   Mm2
.............................................................cUagccauucuacaguguac..............................   1      1   Mm2
.............................................................caagccauucAacaguguac..............................   2      1   Mm2
.............................................................caagccauucuacagugGac..............................   5      1   Mm2
.............................................................cUagccauucuacaguguaca.............................   2      1   Mm2
.............................................................caagccauucuacCguguaca.............................   1      1   Mm2
.............................................................caagccauucuacaguguGca.............................   1      1   Mm2
.............................................................caGgccauucuacaguguaca.............................   1      1   Mm2
.............................................................caagAcauucuacaguguaca.............................   1      1   Mm2
.............................................................caaUccauucuacaguguaca.............................   2      1   Mm2
.............................................................caagccauucuacaguCuaca.............................   17     1   Mm2
.............................................................caagccauucuacaguguaca.............................   5462   0   Mm2
.............................................................caagccauucuacaguguacG.............................   4      1   Mm2
.............................................................caagccauucuacUguguaca.............................   1      1   Mm2
.............................................................caagccauucuaUaguguaca.............................   2      1   Mm2
.............................................................caagccauuUuacaguguaca.............................   6      1   Mm2
.............................................................caagccauucuaAaguguaca.............................   2      1   Mm2
.............................................................caagccauucuacaguguaUa.............................   2      1   Mm2
.............................................................caagccauucuacagugGaca.............................   29     1   Mm2
.............................................................cGagccauucuacaguguaca.............................   2      1   Mm2
.............................................................Naagccauucuacaguguaca.............................   4      1   Mm2
.............................................................caagccauucuacagugCaca.............................   22     1   Mm2
.............................................................Uaagccauucuacaguguaca.............................   7      1   Mm2
.............................................................caagccauucuacaguguUca.............................   2      1   Mm2
.............................................................caagcUauucuacaguguaca.............................   2      1   Mm2
```

```
                     Mature                                     Star
aaauguggugaguuucugug uacccuguagaaccgagcuagu guaucgaguucgaaacuca caagccauucuacaguguaca aagaaacgucgcuucuuucaacaaugaga
.............................................................Gaagccauucuacaguguaca.............................  1     1  Mm2
.............................................................caagccauucuUcaguguaca.............................  1     1  Mm2
.............................................................caagccUuucuacaguguaca.............................  1     1  Mm2
.............................................................caagccauucAacaguguaca.............................  13    1  Mm2
.............................................................caagccauucuacaguUuaca.............................  8     1  Mm2
.............................................................caagccauucuacaguAuaca.............................  14    1  Mm2
.............................................................caagccauucuacaguguacU.............................  14    1  Mm2
.............................................................caagccauucCacaguguaca.............................  1     1  Mm2
.............................................................caagccauucuacagCguaca.............................  1     1  Mm2
.............................................................caagccNuucuacaguguaca.............................  1     1  Mm2
.............................................................caagcNauucuacaguguaca.............................  1     1  Mm2
.............................................................caagccauucuacagugAaca.............................  21    1  Mm2
.............................................................caagccauuGuacaguguaca.............................  1     1  Mm2
.............................................................caagccauuAuacaguguaca.............................  1     1  Mm2
.............................................................caagccauucuacagAguaca.............................  2     1  Mm2
.............................................................caagccauucGacaguguaca.............................  7     1  Mm2
.............................................................caagGcauucuacaguguaca.............................  1     1  Mm2
.............................................................caagccauucuacagugAacaa............................  2     1  Mm2
.............................................................caagAcauucuacaguguacaa............................  1     1  Mm2
.............................................................caagccauucuacGguguacaa............................  1     1  Mm2
.............................................................caagccauucuacaguCuacaa............................  4     1  Mm2
.............................................................caagccauucuacaguguacUa............................  1     1  Mm2
.............................................................caagccauucuacaguAuacaa............................  4     1  Mm2
.............................................................caagccauucAacaguguacaa............................  1     1  Mm2
.............................................................caagccauucuUcaguguacaa............................  1     1  Mm2
.............................................................caagccauucuacagugGacaa............................  7     1  Mm2
.............................................................caagccauucuacaguguacaa............................  1245  0  Mm2
.............................................................caagccauucuacagugCacaa............................  5     1  Mm2
.............................................................caagccauucuacaguguacaC............................  96    1  Mm2
.............................................................caagccauucuacaguguacaU............................  13    1  Mm2
.............................................................Uaagccauucuacaguguacaa............................  3     1  Mm2
.............................................................caagccauucuacaguguacaaa...........................  55    0  Mm2
.............................................................caagccauucuacagugAacaaa...........................  1     1  Mm2
.............................................................caagccauucuacaguguacaaC...........................  29    1  Mm2
.............................................................caagccauucuacaguguacaCa...........................  2     1  Mm2
.............................................................caagccauucuacaguguacaaG...........................  4     1  Mm2
.............................................................caagccauucuacaguguacaaU...........................  45    1  Mm2
.............................................................caagccauucuacaguCuacaaa...........................  1     1  Mm2
.............................................................caagccauucuacaguguacaaaU..........................  3     1  Mm2
..............................................................aagccauucuacagugua...............................  1     0  Mm2
..............................................................aagccauucuacaguguac..............................  2     0  Mm2
..............................................................aagccauucuacaguguaca.............................  31    0  Mm2
..............................................................aagccauucuacaguguacaa............................  4     0  Mm2
..............................................................aagccauucuacaguguacaaU...........................  1     1  Mm2
.................................................................ccauucuacaguguacaaaga.........................  1     0  Mm2
.................................................................ccauucuacaguguacaaagU.........................  1     1  Mm2

...............cuguguacccuguagaaccgagc.........................................................................  23    0  Fm2
...............cuguguacccuguagaaccgagcu........................................................................  33    0  Fm2
...............cuguguacccuguagaaccgagcG........................................................................  2     1  Fm2
................uguguacccuguagaacAgagc.........................................................................  1     1  Fm2
................uguguacccuguagaaccgagc.........................................................................  26    0  Fm2
................uguguacccuguagaacGgagcu........................................................................  1     1  Fm2
................ugGguacccuguagaaccgagcu........................................................................  1     1  Fm2
................uguguacccuguagaacUgagcu........................................................................  2     1  Fm2
................uguguacccuguagaaccgagcu........................................................................  198   0  Fm2
................uguguacccuguagUaccgagcu........................................................................  1     1  Fm2
................uguguacccuguagaaccgagcuagu.....................................................................  35    0  Fm2
................uguguacccuguagaaUcgagcuagu.....................................................................  1     1  Fm2
.................Cuguacccuguagaaccgagcuagu.....................................................................  121   1  Fm2
.................Cuguacccuguagaaccgagcuagug....................................................................  12    1  Fm2
..................uguacccuguagaaccgG...........................................................................  1     1  Fm2
..................uguacccuguagaaccga...........................................................................  12    0  Fm2
..................uguacccuguagaaccgagA.........................................................................  4     1  Fm2
..................uguacccuguagaaccUagc.........................................................................  1     1  Fm2
..................uguacccuguagaaccCagc.........................................................................  1     1  Fm2
..................uguacccuguagaaccgagc.........................................................................  112   0  Fm2
..................uguacccuguagaaccgagU.........................................................................  2     1  Fm2
..................uguUcccuguagaaccgagcu........................................................................  1     1  Fm2
..................uguacccuguagaaccAagcu........................................................................  2     1  Fm2
```

Mature / Star			
aaauguggugaguuucugug**uacccuguagaaccgagcuagu**guaucgaguucgaaacuca**caagccauucuacaguguaca**aagaaacgucgcuucuuucaacaaugaga			
..................uguacccuguagaaccgUgcu..	2	1	Fm2
..................uguacccuguagaaccUagcu..	2	1	Fm2
..................Gguacccuguagaaccgagcu..	1	1	Fm2
..................uguaccNuguagaaccgagcu..	1	1	Fm2
..................uguacccuguUgaaccgagcu..	2	1	Fm2
..................uguacccuguagaaccgCgcu..	3	1	Fm2
..................uguacccugCagaaccgagcu..	1	1	Fm2
..................uAuacccuguagaaccgagcu..	1	1	Fm2
..................uguacccuguagaaccCagcu..	1	1	Fm2
..................uguacccuAuagaaccgagcu..	1	1	Fm2
..................uguacccuguagaaccgGgcu..	3	1	Fm2
..................uguacccuguagaacUgagcu..	1	1	Fm2
..................uguacccuguagaaccgagcu..	1003	0	Fm2
..................uguacccuguaAaaccgagcu..	1	1	Fm2
..................uguacccuguagaaccgagcG..	5	1	Fm2
..................uguacccuguCgaaccgagcu..	2	1	Fm2
..................uguacccuguagaaccUagcua...	1	1	Fm2
..................uguacccuguUgaaccgagcua...	1	1	Fm2
..................uguacccuguagaaccgUgcua...	1	1	Fm2
..................uguacccuguagaaccgagcuU...	13	1	Fm2
..................uguacccuguagaaccgagcua...	293	0	Fm2
..................uguaccUuguagaaccgagcuag..	3	1	Fm2
..................uguacccuguagaaccgagcuaU..	337	1	Fm2
..................uguacccugAagaaccgagcuag..	1	1	Fm2
..................uguacccuguUgaaccgagcuag..	5	1	Fm2
..................uguacccuguagaaccgagcuUg..	1	1	Fm2
..................uAuacccuguagaaccgagcuag..	3	1	Fm2
..................uguacccuguagaaccgUgcuag..	15	1	Fm2
..................uguacccuguagaacUgagcuag..	1	1	Fm2
..................uguacccuguagaaccgagcGag..	1	1	Fm2
..................uguacccuguagaaccgaAcuag..	3	1	Fm2
..................uguUccuguagaaccgagcuag..	1	1	Fm2
..................uguacccuCuagaaccgagcuag..	1	1	Fm2
..................Cguacccuguagaaccgagcuag..	1	1	Fm2
..................uguacccuguaUaaccgagcuag..	1	1	Fm2
..................uguacccuguagaGccgagcuag..	1	1	Fm2
..................uguacccuguagaaccgagUuag..	11	1	Fm2
..................uguacccuAuagaaccgagcuag..	1	1	Fm2
..................uguGcccuguagaaccgagcuag..	1	1	Fm2
..................uguacccuguagaaccCagcuag..	4	1	Fm2
..................uguacccuguGgaaccgagcuag..	4	1	Fm2
..................uguacccuguagaaccgagcuaA..	18	1	Fm2
..................uguacccuguagaaccgGgcuag..	14	1	Fm2
..................uguacccuguagaaccgagcuag..	5275	0	Fm2
..................ugCacccuguagaaccgagcuag..	1	1	Fm2
..................uguacccuguagaaUcgagcuag..	1	1	Fm2
..................uguacNcuguagaaccgagcuag..	1	1	Fm2
..................uguacccugCagaaccgagcuag..	1	1	Fm2
..................Nguacccuguagaaccgagcuag..	3	1	Fm2
..................uguacccuguagaaccgagcuGg..	4	1	Fm2
..................uguacccuguCgaaccgagcuag..	6	1	Fm2
..................uguacAcuguagaaccgagcuag..	1	1	Fm2
..................uguacccuguagaaccAagcuag..	18	1	Fm2
..................uguacUcuguagaaccgagcuag..	6	1	Fm2
..................uguacccuguagaaccgCgcuag..	22	1	Fm2
..................uguaGccuguagaaccgagcuag..	2	1	Fm2
..................uguacccuguagaaccUagcuag..	5	1	Fm2
..................uguacccuguGgaaccgagcuagu...	138	1	Fm2
..................uguacccuguagaaccCagcuagu...	307	1	Fm2
..................uguacccuguagaaccgGgcuagu...	337	1	Fm2
..................uguacccuguagaaccgagcuaAu...	28	1	Fm2
..................uguacccuguagaCccgagcuagu...	6	1	Fm2
..................uguacccuguagaaccgaAcuagu...	20	1	Fm2
..................uguacccuguagaaAcgagcuagu...	5	1	Fm2
..................uguacccuguagaaccgagcCagu...	21	1	Fm2
..................uguacccGguagaaccgagcuagu...	3	1	Fm2
..................uguacNcuguagaaccgagcuagu...	15	1	Fm2
..................uguaccNuguagaaccgagcuagu...	6	1	Fm2
..................uguacccuguaAaaccgagcuagu...	7	1	Fm2
..................uguacccAguagaaccgagcuagu...	10	1	Fm2

Mature **Star**

aaauguggugaguuucuguguacccuguagaaccgagcuaguguaucgaguucgaaacucacaagccauucuacaguguacaaagaaacgucgcuucuuucaacaaugaga			
..................uguacccuguagaaccgUgcuagu...	431	1	Fm2
..................uguacccuguagaaccgagcuagG...	324	1	Fm2
..................uguacccuguagaaccUagcuagu...	275	1	Fm2
..................uguGcccuguagaaccgagcuagu...	31	1	Fm2
..................uguacccuguagaacAgagcuagu...	15	1	Fm2
..................uguCccuguagaaccgagcuagu...	7	1	Fm2
..................uguacccugAagaaccgagcuagu...	10	1	Fm2
..................uguacccuguUgaaccgagcuagu...	95	1	Fm2
..................uguacccuguagaaccgagcuCgu...	4	1	Fm2
..................uguacccuguagaaccgagcuGgu...	143	1	Fm2
..................ugNacccuguagaaccgagcuagu...	1	1	Fm2
..................uguacccuguCgaaccgagcuagu...	140	1	Fm2
..................uguacccuguagaaccgagcuUgu...	19	1	Fm2
..................uguacccuguagaaccgagGuagu...	1	1	Fm2
..................uguacccuguagaaccgagcuagu...	144818	0	Fm2
..................uguacccuguagaaccgagAuagu...	9	1	Fm2
..................uguacccuguagUaccgagcuagu...	21	1	Fm2
..................uguacccuguagCaccgagcuagu...	3	1	Fm2
..................uguacccuguagaaccgagcuagA...	193	1	Fm2
..................uguacccuguagaaccgaUcuagu...	16	1	Fm2
..................uguacccuCuagaaccgagcuagu...	6	1	Fm2
..................uguacccuguaCaaccgagcuagu...	7	1	Fm2
..................Cguacccuguagaaccgagcuagu...	32	1	Fm2
..................uguacccuUuagaaccgagcuagu...	18	1	Fm2
..................Gguacccuguagaaccgagcuagu...	3	1	Fm2
..................uguaccUuguagaaccgagcuagu...	27	1	Fm2
..................uguacccuguagaGccgagcuagu...	31	1	Fm2
..................uguacccuguagaUccgagcuagu...	16	1	Fm2
..................uguacccuguaUaaccgagcuagu...	17	1	Fm2
..................uguacccuguagaacUgagcuagu...	35	1	Fm2
..................uguacccuguagaaccAagcuagu...	311	1	Fm2
..................uguacccuguagaaccgagcGagu...	7	1	Fm2
..................Aguacccuguagaaccgagcuagu...	13	1	Fm2
..................Nguacccuguagaaccgagcuagu...	83	1	Fm2
..................uAuacccuguagaaccgagcuagu...	64	1	Fm2
..................uguaccAuguagaaccgagcuagu...	35	1	Fm2
..................uguacccugCagaaccgagcuagu...	36	1	Fm2
..................uguacccuguagGaccgagcuagu...	10	1	Fm2
..................ugAacccuguagaaccgagcuagu...	24	1	Fm2
..................uguacGcuguagaaccgagcuagu...	7	1	Fm2
..................uguacccuguagaaUcgagcuagu...	14	1	Fm2
..................uguacUcuguagaaccgagcuagu...	153	1	Fm2
..................uguacccuguagaacGgagcuagu...	3	1	Fm2
..................uguaccGuguagaaccgagcuagu...	6	1	Fm2
..................uguacccuguagaaccgagcAagu...	5	1	Fm2
..................uguacccuguagaaccgagcuaCu...	3	1	Fm2
..................uguaUccuguagaaccgagcuagu...	21	1	Fm2
..................uguacAcuguagaaccgagcuagu...	34	1	Fm2
..................ugCacccuguagaaccgagcuagu...	19	1	Fm2
..................uguacccuguagaaccgaCcuagu...	3	1	Fm2
..................uguaGccuguagaaccgagcuagu...	17	1	Fm2
..................uguUccuguagaaccgagcuagu...	13	1	Fm2
..................uguacccugGagaaccgagcuagu...	6	1	Fm2
..................uguacccuguagaaccgagcuaUu...	155	1	Fm2
..................ugGacccuguagaaccgagcuagu...	24	1	Fm2
..................uCuacccuguagaaccgagcuagu...	61	1	Fm2
..................uguacccCguagaaccgagcuagu...	16	1	Fm2
..................uguaAccuguagaaccgagcuagu...	23	1	Fm2
..................uguacccuguagaaccgagcuagC...	20	1	Fm2
..................uguacccuguagaaccgCgcuagu...	580	1	Fm2
..................uguacccuguagaaccgagUuagu...	69	1	Fm2
..................uguacccuAuagaaccgagcuagu...	11	1	Fm2
..................uUuacccuguagaaccgagcuagu...	28	1	Fm2
..................uAuacccuguagaaccgagcuagug..	2	1	Fm2
..................uguacccuguagaaccgagcuaguU..	5796	1	Fm2
..................uguacAcuguagaaccgagcuagug..	2	1	Fm2
..................ugGacccuguagaaccgagcuagug..	1	1	Fm2
..................uguacUcuguagaaccgagcuagug..	3	1	Fm2
..................uguacccuguagaaccgagcuaguA..	50	1	Fm2
..................uUuacccuguagaaccgagcuagug..	1	1	Fm2

Mature **Star**

```
aaauguggugaguuucugug uacccuguagaaccgagcuagu guaucgaguucgaaacuca caagccauucuacaguguaca aagaaacgucgcuucuuucaacaaugaga
```

```
aaauguggugaguuucuguguacccuguagaaccgagcuaguguaucgaguucgaaacucacaagccauucuacaguguacaaagaaacgucgcuucuuucaacaaugaga
..................uguacccuguagaaccgGgcuagug....................................................................  2     1  Fm2
..................uguacccuguagaaccgagcuagug....................................................................  1710  0  Fm2
..................uguacccuguagaaccCagcuagug....................................................................  3     1  Fm2
..................uguacccuguagaaccUagcuagug....................................................................  3     1  Fm2
..................uguacccuguCgaaccgagcuagug....................................................................  2     1  Fm2
..................Cguacccuguagaaccgagcuagug....................................................................  1     1  Fm2
..................uguacccuguagaaccgUgcuagug....................................................................  6     1  Fm2
..................uguacccuguGgaaccgagcuagug....................................................................  2     1  Fm2
..................uguacccuguagaaccAagcuagug....................................................................  1     1  Fm2
..................Nguacccuguagaaccgagcuagug....................................................................  1     1  Fm2
..................uguacNcuguagaaccgagcuagug....................................................................  1     1  Fm2
..................uguacccuguagaaccgagcuaguC....................................................................  5     1  Fm2
..................uguacccuguagaaccgCgcuagug....................................................................  4     1  Fm2
..................uCuacccuguagaaccgagcuagug....................................................................  19    1  Fm2
..................uguacccuguagaaccgagcuagugG...................................................................  360   1  Fm2
..................uguacccuguagaaccgagcuaguCu...................................................................  11    1  Fm2
..................uguaccUuguagaaccgagcuagugu...................................................................  1     1  Fm2
..................uguacccuguagaaccgagcuagAgu...................................................................  2     1  Fm2
..................uguacccuguagaaccgaUcuagugu...................................................................  1     1  Fm2
..................uguacccuguagaaccgUgcuagugu...................................................................  7     1  Fm2
..................uguaUccuguagaaccgagcuagugu...................................................................  1     1  Fm2
..................ugAacccuguagaaccgagcuagugu...................................................................  2     1  Fm2
..................uguacccuguagaacUgagcuagugu...................................................................  2     1  Fm2
..................uguacccuguagaaccgGgcuagugu...................................................................  8     1  Fm2
..................uguUcccuguagaaccgagcuagugu...................................................................  1     1  Fm2
..................ugNacccuguagaaccgagcuagugu...................................................................  1     1  Fm2
..................uAuacccuguagaaccgagcuagugu...................................................................  3     1  Fm2
..................uguacAcuguagaaccgagcuagugu...................................................................  2     1  Fm2
..................uguacUcuguagaaccgagcuagugu...................................................................  3     1  Fm2
..................uguacccuguagaaccgCgcuagugu...................................................................  8     1  Fm2
..................uguacccuguagaaccgagcGagugu...................................................................  3     1  Fm2
..................uguacccuguagaaccgagcuaguAu...................................................................  84    1  Fm2
..................uguacccuguagaaccgagcuGgugu...................................................................  13    1  Fm2
..................uguacccuguagaaccgagcuaguUu...................................................................  578   1  Fm2
..................uguacccuguCgaaccgagcuagugu...................................................................  2     1  Fm2
..................uguacccuguGgaaccgagcuagugu...................................................................  2     1  Fm2
..................uguacccuguUgaaccgagcuagugu...................................................................  2     1  Fm2
..................uguacccuguagaaccCagcuagugu...................................................................  7     1  Fm2
..................uguacccuguaAaaccgagcuagugu...................................................................  1     1  Fm2
..................uguacccuguagaaccAagcuagugu...................................................................  7     1  Fm2
..................uguacccuguagaaccgagcuagugC...................................................................  5     1  Fm2
..................uguacccuguagaaccgagcuagugu...................................................................  3119  0  Fm2
..................Nguacccuguagaaccgagcuagugu...................................................................  4     1  Fm2
..................uguacccugCagaaccgagcuagugu...................................................................  1     1  Fm2
..................uguacccuguagaaccUagcuagugu...................................................................  6     1  Fm2
..................ugCacccuguagaaccgagcuagugu...................................................................  1     1  Fm2
..................uguaccAuguagaaccgagcuagugu...................................................................  2     1  Fm2
..................uguacccuguagaaUcgagcuagugu...................................................................  1     1  Fm2
..................uguacccuAuagaaccgagcuagugu...................................................................  1     1  Fm2
..................uguacccuguagaaccgagcuagugA...................................................................  51    1  Fm2
..................uguacccuguagaaccgagcuaguguU..................................................................  245   1  Fm2
..................uguacccuguagaaccgagcuaguguUu.................................................................  11    1  Fm2
..................uguacccuguagaaccgagcuGguguauc................................................................  1     1  Fm2
..................uguacccuguagaaccgagcuaguguauc................................................................  10    0  Fm2
..................uguacccuguagaaccgGgcuaguguauc................................................................  1     1  Fm2
..................uguacccuguagaaccgagcuaguguaucU...............................................................  8     1  Fm2
...................Nuacccuguagaaccgag..........................................................................  1     1  Fm2
...................guacccuguagaaccgagc.........................................................................  5     0  Fm2
...................Uuacccuguagaaccgagc.........................................................................  1     1  Fm2
...................guacccuguagaaccgagcu........................................................................  70    0  Fm2
...................guacccuguagaaccgagcG........................................................................  3     1  Fm2
...................guacccuguagaGccgagcu........................................................................  1     1  Fm2
...................guacccuguagaaccgaAcu........................................................................  1     1  Fm2
...................Auacccuguagaaccgagcu........................................................................  93    1  Fm2
...................Auacccuguagaaccgagcua.......................................................................  5     1  Fm2
...................guacccuguagaaccgagcua.......................................................................  5     0  Fm2
...................Auacccuguagaaccgagcuag......................................................................  22    1  Fm2
...................guacccuguagaaccgGgcuag......................................................................  1     1  Fm2
...................Uuacccuguagaaccgagcuag......................................................................  1     1  Fm2
...................guaAccuguagaaccgagcuag......................................................................  1     1  Fm2
```

```
                    Mature                  Star
aaauguggugaguuucugugguacccuguagaaccgagcuaguguaucgaguucgaaacucacaagccauucuacaguguacaaagaaacgucgcuucuuucaacaaugaga
...................guacccuguagaaccgagcuag......................................................................  110    0  Fm2
...................guacccuguagaaccgaCcuag......................................................................  1      1  Fm2
...................guacccuguagaaccgagcuaU......................................................................  9      1  Fm2
...................guacccuguagaaccgagAuag......................................................................  1      1  Fm2
...................guacccuguagaaccgagcGag......................................................................  7      1  Fm2
...................Cuacccuguagaaccgagcuag......................................................................  20     1  Fm2
...................gCacccuguagaaccgagcuagu.....................................................................  4      1  Fm2
...................guacccCguagaaccgagcuagu.....................................................................  4      1  Fm2
...................guacccNguagaaccgagcuagu.....................................................................  2      1  Fm2
...................Cuacccuguagaaccgagcuagu.....................................................................  877    1  Fm2
...................Auacccuguagaaccgagcuagu.....................................................................  335    1  Fm2
...................Uuacccuguagaaccgagcuagu.....................................................................  11     1  Fm2
...................guacccuAuagaaccgagcuagu.....................................................................  1      1  Fm2
...................guaccUuguagaaccgagcuagu.....................................................................  2      1  Fm2
...................guNcccuguagaaccgagcuagu.....................................................................  1      1  Fm2
...................guacccuguagaaccgagcuagu.....................................................................  11361  0  Fm2
...................guaccNuguagaaccgagcuagu.....................................................................  2      1  Fm2
...................guacccuguagUaccgagcuagu.....................................................................  1      1  Fm2
...................guacccuguagaacGgagcuagu.....................................................................  4      1  Fm2
...................guacccuguagaaccgUgcuagu.....................................................................  21     1  Fm2
...................Nuacccuguagaaccgagcuagu.....................................................................  5      1  Fm2
...................guacccuguagaaccAagcuagu.....................................................................  3      1  Fm2
...................guacccuguagGaccgagcuagu.....................................................................  1      1  Fm2
...................guacccuguagaaccgagcuagA.....................................................................  24     1  Fm2
...................guacccuguaUaaccgagcuagu.....................................................................  12     1  Fm2
...................guacccuguagaaccgagcuaUu.....................................................................  4      1  Fm2
...................guacccuguagaaccgagAuagu.....................................................................  1      1  Fm2
...................guacccuguagaaccgagcuagC.....................................................................  3      1  Fm2
...................guacccuguagaacUgagcuagu.....................................................................  3      1  Fm2
...................guUcccuguagaaccgagcuagu.....................................................................  1      1  Fm2
...................guacccuguagaaccgGgcuagu.....................................................................  4      1  Fm2
...................guacccuguagaaUcgagcuagu.....................................................................  12     1  Fm2
...................guacccuguagaaAcgagcuagu.....................................................................  1      1  Fm2
...................guacccuguagaaccgagcuGgu.....................................................................  31     1  Fm2
...................gAacccuguagaaccgagcuagu.....................................................................  5      1  Fm2
...................guacccuguagaaccgagcuagG.....................................................................  36     1  Fm2
...................guaAccuguagaaccgagcuagu.....................................................................  5      1  Fm2
...................guacccGguagaaccgagcuagu.....................................................................  1      1  Fm2
...................guacccuguagaaccgaUcuagu.....................................................................  53     1  Fm2
...................guacccuguaAaaccgagcuagu.....................................................................  17     1  Fm2
...................guacccuguagaaccUagcuagu.....................................................................  1      1  Fm2
...................guacccuguagaaccgaAcuagu.....................................................................  48     1  Fm2
...................guacGcuguagaaccgagcuagu.....................................................................  1      1  Fm2
...................guacccuguagaaccgCgcuagu.....................................................................  27     1  Fm2
...................guCcccuguagaaccgagcuagu.....................................................................  1      1  Fm2
...................guacccuguagaaccgagcuaAu.....................................................................  1      1  Fm2
...................guaccAuguagaaccgagcuagu.....................................................................  2      1  Fm2
...................guacccuguagCaccgagcuagu.....................................................................  1      1  Fm2
...................guacccuUuagaaccgagcuagu.....................................................................  1      1  Fm2
...................guacAcuguagaaccgagcuagu.....................................................................  3      1  Fm2
...................guacccuguagaaccgagcGagu.....................................................................  240    1  Fm2
...................guacccuguaCaaccgagcuagu.....................................................................  8      1  Fm2
...................guacccuguagaaccgaCcuagu.....................................................................  38     1  Fm2
...................guacccuguGgaaccgagcuagu.....................................................................  2      1  Fm2
...................guacccuguagaGccgagcuagu.....................................................................  4      1  Fm2
...................guaUccuguagaaccgagcuagu.....................................................................  2      1  Fm2
...................guacUcuguagaaccgagcuagu.....................................................................  2      1  Fm2
...................guacccuguUgaaccgagcuagu.....................................................................  2      1  Fm2
...................guacccuguagaaccgagcuagug....................................................................  748    0  Fm2
...................guacccuguagGaccgagcuagug....................................................................  1      1  Fm2
...................guacccuguaUaaccgagcuagug....................................................................  1      1  Fm2
...................Uuacccuguagaaccgagcuagug....................................................................  2      1  Fm2
...................guacccuguagaaccgaAcuagug....................................................................  4      1  Fm2
...................guacccuguagaaccgagcuaguU....................................................................  701    1  Fm2
...................guacccuguagaaccgGgcuagug....................................................................  1      1  Fm2
...................guacccuguaAaaccgagcuagug....................................................................  2      1  Fm2
...................guacccuguaCaaccgagcuagug....................................................................  2      1  Fm2
...................Nuacccuguagaaccgagcuagug....................................................................  1      1  Fm2
...................guacccuguagaaccgagcGagug....................................................................  6      1  Fm2
...................Auacccuguagaaccgagcuagug....................................................................  7      1  Fm2
```

Mature **Star**

```
aaauguggugaguuucugugguacccuguagaaccgagcuaguguaucgaguucgaaacucacaagccauucuacaguguacaaagaaacgucgcuucuuucaacaaugaga
...................guacccuguagaaccgCgcuagug....................................................................  3     1  Fm2
...................guacccuguagaaccgagcuaguA....................................................................  19    1  Fm2
...................guaccUuguagaaccgagcuagug....................................................................  1     1  Fm2
...................guacccuguagaaccgaCcuagug....................................................................  3     1  Fm2
...................guacccuguagaaccgaUcuagug....................................................................  4     1  Fm2
...................guacccuguagaaccgUgcuagug....................................................................  1     1  Fm2
...................Auacccuguagaaccgagcuagugu...................................................................  4     1  Fm2
...................guacccuguagaaccgagcuGgugu...................................................................  1     1  Fm2
...................guacccuguagaaccgGgcuagugu...................................................................  1     1  Fm2
...................guacccuguagaaccgCgcuagugu...................................................................  1     1  Fm2
...................guacUcuguagaaccgagcuagugu...................................................................  1     1  Fm2
...................guacccuguagaaccgaUcuagugu...................................................................  2     1  Fm2
...................guacccCguagaaccgagcuagugu...................................................................  1     1  Fm2
...................guacccuguagaaccgagcuagugG...................................................................  91    1  Fm2
...................guacccuguaAaccgagcuagugu...................................................................  2     1  Fm2
...................guacccuguagaaccgagcuagugA...................................................................  4     1  Fm2
...................guacccuguGgaaccgagcuagugu...................................................................  2     1  Fm2
...................Cuacccuguagaaccgagcuagugu...................................................................  96    1  Fm2
...................guacccuguagaaccgagcGagugu...................................................................  2     1  Fm2
...................guacccuUuagaaccgagcuagugu...................................................................  1     1  Fm2
...................guacccuguagaaccgaCcuagugu...................................................................  6     1  Fm2
...................guacccuguagaaccgagcuaguUu...................................................................  56    1  Fm2
...................guacccuguagaaccgagcuaAugu...................................................................  1     1  Fm2
...................guacccuguagaaccgagUuagugu...................................................................  1     1  Fm2
...................guacccuguagCaccgagcuagugu...................................................................  1     1  Fm2
...................guacccuguagGaccgagcuagugu...................................................................  1     1  Fm2
...................guacccuguagaaccgaAcuagugu...................................................................  5     1  Fm2
...................guacccuguagaaccgagcuagugu...................................................................  925   0  Fm2
...................Nuacccuguagaaccgagcuagugu...................................................................  1     1  Fm2
...................guUcccuguagaaccgagcuagugu...................................................................  1     1  Fm2
...................guacccuguagaaccgagcuaguguG..................................................................  1     1  Fm2
...................guacccuguagaaccgagcuagugua..................................................................  2     0  Fm2
...................guacccuguagaaccgagcuaguguU..................................................................  142   1  Fm2
...................guacccuguagaaccgagcuaguguUu.................................................................  24    1  Fm2
...................guacccuguagaaccgagcuaguguau.................................................................  1     0  Fm2
...................guacccuguagaaccgagcuaguguaucgagu............................................................  19    0  Fm2
...................guacccuguagaaccgagcuaguguaucgCgu............................................................  1     1  Fm2
....................uacccuguagaaccgagG.........................................................................  2     1  Fm2
....................uacccuguagaaccgaAc.........................................................................  1     1  Fm2
....................uaUccuguagaaccgagc.........................................................................  1     1  Fm2
....................uacccuguagaaccgagA.........................................................................  21    1  Fm2
....................uacccuguagaaccgaCc.........................................................................  1     1  Fm2
....................uacccuguagUaccgagc.........................................................................  1     1  Fm2
....................uacccuguagaaccgGgc.........................................................................  1     1  Fm2
....................uacccuguagaaccgagc.........................................................................  556   0  Fm2
....................uGcccuguagaaccgagc.........................................................................  1     1  Fm2
....................uaccUuguagaaccgagc.........................................................................  1     1  Fm2
....................uacccuguagaaccgagU.........................................................................  3     1  Fm2
....................uacAcuguagaaccgagc.........................................................................  1     1  Fm2
....................uaccAuguagaaccgagc.........................................................................  1     1  Fm2
....................uacccuguagaaccgaUc.........................................................................  1     1  Fm2
....................uacccuguagaacUgagcu........................................................................  5     1  Fm2
....................uacccuguagaaccgagAu........................................................................  22    1  Fm2
....................uacccuguagaaUcgagcu........................................................................  9     1  Fm2
....................uacccuguagUaccgagcu........................................................................  2     1  Fm2
....................uacGcuguagaaccgagcu........................................................................  1     1  Fm2
....................uacccuguagaaccgagGu........................................................................  11    1  Fm2
....................uacccuguagaaccgUgcu........................................................................  1     1  Fm2
....................uacccuguGgaaccgagcu........................................................................  1     1  Fm2
....................uacccuguagaaccgaAcu........................................................................  6     1  Fm2
....................uacccuguagCaccgagcu........................................................................  2     1  Fm2
....................uacccuguagaaccgagcu........................................................................  2883  0  Fm2
....................uacccuguagaaccgagUu........................................................................  9     1  Fm2
....................uaccGuguagaaccgagcu........................................................................  1     1  Fm2
....................uaccAuguagaaccgagcu........................................................................  2     1  Fm2
....................NacccuguagaaccgagcU........................................................................  3     1  Fm2
....................uacccuguagaacAgagcu........................................................................  1     1  Fm2
....................uacccuguagaaccgaCcu........................................................................  10    1  Fm2
....................uacccugAagaaccgagcu........................................................................  1     1  Fm2
....................uacccuguagaaccAagcu........................................................................  1     1  Fm2
```

```
                    Mature                                  Star
aaauguggugaguuucuguguacccuguagaaccgagcuaguguaucgaguucgaaacucacaagccauucuacaguguacaaagaaacgucgcuucuuucaacaaugaga
....................uUcccuguagaaccgagcu........................................................................  1      1   Fm2
....................uacccuguagaaccgaUcu........................................................................  4      1   Fm2
....................uacAcuguagaaccgagcu........................................................................  1      1   Fm2
....................uacccuguagGaccgagcu........................................................................  1      1   Fm2
....................uacccuguagaUccgagcu........................................................................  1      1   Fm2
....................Cacccuguagaaccgagcu........................................................................  1      1   Fm2
....................uacccuguUgaaccgagcu........................................................................  1      1   Fm2
....................uacccuguagaacGgagcu........................................................................  1      1   Fm2
....................uacccuguagaaccgagcA........................................................................  5      1   Fm2
....................uacccuguagaaccgagGua.......................................................................  2      1   Fm2
....................uacccuguagaaccgagcuG.......................................................................  2      1   Fm2
....................uacccuguagaaccgagUua.......................................................................  3      1   Fm2
....................uacccuguagCaccgagcua.......................................................................  2      1   Fm2
....................uacccuguagaaccgagcuU.......................................................................  124    1   Fm2
....................uacccuguagaaccgagcuC.......................................................................  1      1   Fm2
....................uacccuguagUaccgagcua.......................................................................  2      1   Fm2
....................uacccuCuagaaccgagcua.......................................................................  1      1   Fm2
....................uGcccuguagaaccgagcua.......................................................................  1      1   Fm2
....................uacccuguGgaaccgagcua.......................................................................  1      1   Fm2
....................uacccuguagaaccgaCcua.......................................................................  2      1   Fm2
....................uacccuguagaaccgagcua.......................................................................  807    0   Fm2
....................uacccuguagaaccgaAcua.......................................................................  1      1   Fm2
....................uaAccuguagaaccgagcua.......................................................................  1      1   Fm2
....................uacAcuguagaaccgagcua.......................................................................  1      1   Fm2
....................uacccuguagaaccgagAua.......................................................................  8      1   Fm2
....................uacccuguagaaccUagcua.......................................................................  1      1   Fm2
....................uacccuAuagaaccgagcua.......................................................................  1      1   Fm2
....................uaccAuguagaaccgagcua.......................................................................  1      1   Fm2
....................uacccGguagaaccgagcuag......................................................................  2      1   Fm2
....................Aacccuguagaaccgagcuag......................................................................  4      1   Fm2
....................uacccuguUgaaccgagcuag......................................................................  4      1   Fm2
....................uacccuguaCaaccgagcuag......................................................................  1      1   Fm2
....................uacccuguGgaaccgagcuag......................................................................  2      1   Fm2
....................Nacccuguagaaccgagcuag......................................................................  12     1   Fm2
....................uacccuguagaacAgagcuag......................................................................  5      1   Fm2
....................uacccuguagaaccgagUuag......................................................................  118    1   Fm2
....................uacccuguagaaccgagcuaU......................................................................  920    1   Fm2
....................uacccuguagaaccgagGuag......................................................................  61     1   Fm2
....................uacccuguagaaccgagcuUg......................................................................  3      1   Fm2
....................uacccuguagaaccgagcuaA......................................................................  26     1   Fm2
....................uacccuguagCaccgagcuag......................................................................  21     1   Fm2
....................uacccuguagaaccgaUcuag......................................................................  35     1   Fm2
....................uaccAuguagaaccgagcuag......................................................................  9      1   Fm2
....................uacccuguagaGccgagcuag......................................................................  6      1   Fm2
....................uCcccuguagaaccgagcuag......................................................................  3      1   Fm2
....................uacccuguagaaccgagcuag......................................................................  22803  0   Fm2
....................uGcccuguagaaccgagcuag......................................................................  9      1   Fm2
....................uacccugAagaaccgagcuag......................................................................  2      1   Fm2
....................uacccCguagaaccgagcuag......................................................................  7      1   Fm2
....................uacccuUuagaaccgagcuag......................................................................  3      1   Fm2
....................uacccuguagGaccgagcuag......................................................................  21     1   Fm2
....................uaUccuguagaaccgagcuag......................................................................  6      1   Fm2
....................uacccuguagaaUcgagcuag......................................................................  4      1   Fm2
....................uaAccuguagaaccgagcuag......................................................................  3      1   Fm2
....................uaNccuguagaaccgagcuag......................................................................  1      1   Fm2
....................uacccuguagaaccgagcuGg......................................................................  44     1   Fm2
....................uacccuguagaCccgagcuag......................................................................  2      1   Fm2
....................uaccGuguagaaccgagcuag......................................................................  1      1   Fm2
....................uacccuguaAaaccgagcuag......................................................................  1      1   Fm2
....................uacccuguagaaccgGgcuag......................................................................  3      1   Fm2
....................uacccNguagaaccgagcuag......................................................................  1      1   Fm2
....................uacccuNuagaaccgagcuag......................................................................  4      1   Fm2
....................uacccuguagaaccgaAcuag......................................................................  55     1   Fm2
....................uacUcuguagaaccgagcuag......................................................................  4      1   Fm2
....................uacccuguagaaccgagcAag......................................................................  1      1   Fm2
....................uacccuguCgaaccgagcuag......................................................................  1      1   Fm2
....................uacccuguagUaccgagcuag......................................................................  25     1   Fm2
....................uacGcuguagaaccgagcuag......................................................................  3      1   Fm2
....................uUcccuguagaaccgagcuag......................................................................  4      1   Fm2
....................uacccAguagaaccgagcuag......................................................................  1      1   Fm2
```

```
                    Mature                  Star
aaauguggugaguuucuguguacccuguagaaccgagcuaguguaucgaguucgaaacucacaagccauucuacaguguacaaagaaacgucgcuucuuucaacaaugaga
....................uacccuguagaaccgUgcuag......................................................................   4      1   Fm2
....................uacccuguagaaccCagcuag......................................................................   1      1   Fm2
....................uacccuguagaacUgagcuag......................................................................   14     1   Fm2
....................uaccUuguagaaccgagcuag......................................................................   5      1   Fm2
....................uacccuguagaUccgagcuag......................................................................   6      1   Fm2
....................uacccuguagaaccgagcCag......................................................................   2      1   Fm2
....................uacccugCagaaccgagcuag......................................................................   2      1   Fm2
....................uacccuguagaacGgagcuag......................................................................   1      1   Fm2
....................uacccuguagaaccUagcuag......................................................................   2      1   Fm2
....................uacccuguagaaccAagcuag......................................................................   4      1   Fm2
....................uacAcuguagaaccgagcuag......................................................................   14     1   Fm2
....................uacccuguagaaccgaCcuag......................................................................   57     1   Fm2
....................uacccuAuagaaccgagcuag......................................................................   1      1   Fm2
....................uacccuguaUaaccgagcuag......................................................................   4      1   Fm2
....................uacccuguagaaccgagAuag......................................................................   228    1   Fm2
....................Cacccuguagaaccgagcuag......................................................................   2      1   Fm2
....................uaGccuguagaaccgagcuag......................................................................   6      1   Fm2
....................uacccuguagaaccgaUcuagu.....................................................................   2796   1   Fm2
....................uacccuguagaaccUagcuagu.....................................................................   222    1   Fm2
....................uaccUuguagaaccgagcuagu.....................................................................   239    1   Fm2
....................uacccugAagaaccgagcuagu.....................................................................   120    1   Fm2
....................uacccuguagaaccgaCcuagu.....................................................................   3324   1   Fm2
....................uacccuguagUaccgagcuagu.....................................................................   1567   1   Fm2
....................uacccuguagaaccgagcAagu.....................................................................   42     1   Fm2
....................uacccuguagaUccgagcuagu.....................................................................   580    1   Fm2
....................uacUcuguagaaccgagcuagu.....................................................................   287    1   Fm2
....................uacccuguagaaccCagcuagu.....................................................................   67     1   Fm2
....................Nacccuguagaaccgagcuagu.....................................................................   793    1   Fm2
....................uacccuguagaaccAagcuagu.....................................................................   290    1   Fm2
....................uacccuguagaaccgagcuCgu.....................................................................   55     1   Fm2
....................uacccuguagaaccgagAuagu.....................................................................   14993  1   Fm2
....................uacccuguaCaaccgagcuagu.....................................................................   53     1   Fm2
....................uacccuguagaaccgGgcuagu.....................................................................   169    1   Fm2
....................uacccuguagaaccgagcuaUu.....................................................................   335    1   Fm2
....................uacccuguagaaccgCgcuagu.....................................................................   37     1   Fm2
....................uacccuguagaaccgagcCagu.....................................................................   153    1   Fm2
....................uacccNguagaaccgagcuagu.....................................................................   251    1   Fm2
....................uacccuguagaaccgUgcuagu.....................................................................   212    1   Fm2
....................uaAccuguagaaccgagcuagu.....................................................................   304    1   Fm2
....................uacccuguagaaUcgagcuagu.....................................................................   406    1   Fm2
....................uacccuguagaaccgagNuagu.....................................................................   1      1   Fm2
....................uacccuguagaaccgagGuagu.....................................................................   5152   1   Fm2
....................uacccuguagaaccgagcuagA.....................................................................   1919   1   Fm2
....................uacccuguagaaccgagcuagG.....................................................................   3372   1   Fm2
....................uacccuguagaacAgagcuagu.....................................................................   416    1   Fm2
....................uacccuguagaacUgagcuagu.....................................................................   585    1   Fm2
....................uacAcuguagaaccgagcuagu.....................................................................   722    1   Fm2
....................Cacccuguagaaccgagcuagu.....................................................................   290    1   Fm2
....................uaccAuguagaaccgagcuagu.....................................................................   418    1   Fm2
....................uacccuguagaaccgagcuagC.....................................................................   169    1   Fm2
....................uacccuNuagaaccgagcuagu.....................................................................   104    1   Fm2
....................Aacccuguagaaccgagcuagu.....................................................................   144    1   Fm2
....................uacccuguagaaAcgagcuagu.....................................................................   55     1   Fm2
....................uacccuguagaaccgagcuaAu.....................................................................   127    1   Fm2
....................uaGccuguagaaccgagcuagu.....................................................................   384    1   Fm2
....................uacccCguagaaccgagcuagu.....................................................................   217    1   Fm2
....................uacccuguagaGccgagcuagu.....................................................................   194    1   Fm2
....................Gacccuguagaaccgagcuagu.....................................................................   20     1   Fm2
....................uacccuguagaaccgaNcuagu.....................................................................   2      1   Fm2
....................uacccuUuagaaccgagcuagu.....................................................................   137    1   Fm2
....................uNcccuguagaaccgagcuagu.....................................................................   12     1   Fm2
....................uaccGuguagaaccgagcuagu.....................................................................   80     1   Fm2
....................uacccuguagaaccgaAcuagu.....................................................................   3219   1   Fm2
....................uacccuguagaaGcgagcuagu.....................................................................   48     1   Fm2
....................uacccuguGgaaccgagcuagu.....................................................................   210    1   Fm2
....................uacccuguUgaaccgagcuagu.....................................................................   171    1   Fm2
....................uacccuguagaaccgagUuagu.....................................................................   7490   1   Fm2
....................uUcccuguagaaccgagcuagu.....................................................................   350    1   Fm2
....................uGcccuguagaaccgagcuagu.....................................................................   359    1   Fm2
....................uacccuguaAaaccgagcuagu.....................................................................   105    1   Fm2
```

```
                    Mature                  Star
aaauguggugaguuucugug uacccuguagaaccgagcuagu guaucgaguucgaaacuca caagccauucuacaguguaca aagaaacgucgcuucuuucaacaaugaga
....................uacccugGagaaccgagcuagu.....................................................................  44       1   Fm2
....................uacccuCuagaaccgagcuagu.....................................................................  114      1   Fm2
....................uacGcuguagaaccgagcuagu.....................................................................  150      1   Fm2
....................uacccuguagaaccgagcuUgu.....................................................................  495      1   Fm2
....................uacccuguagaaccgagcuagu.....................................................................  1570296  0   Fm2
....................uacccuguagaCccgagcuagu.....................................................................  33       1   Fm2
....................uacccuguagaaccgagcuaCu.....................................................................  44       1   Fm2
....................uCcccuguagaaccgagcuagu.....................................................................  143      1   Fm2
....................uacccuguaUaaccgagcuagu.....................................................................  183      1   Fm2
....................uacccugCagaaccgagcuagu.....................................................................  267      1   Fm2
....................uacccuguCgaaccgagcuagu.....................................................................  22       1   Fm2
....................uacccuAuagaaccgagcuagu.....................................................................  207      1   Fm2
....................uacccuguagGaccgagcuagu.....................................................................  1670     1   Fm2
....................uacccuguagaaccgagcuGgu.....................................................................  1933     1   Fm2
....................uacccGguagaaccgagcuagu.....................................................................  77       1   Fm2
....................uacccuguagCaccgagcuagu.....................................................................  1456     1   Fm2
....................uaNccuguagaaccgagcuagu.....................................................................  7        1   Fm2
....................uacccAguagaaccgagcuagu.....................................................................  85       1   Fm2
....................uaUccuguagaaccgagcuagu.....................................................................  294      1   Fm2
....................uacccuguagaacGgagcuagu.....................................................................  114      1   Fm2
....................uacccuguaCaaccgagcuagug....................................................................  1        1   Fm2
....................uacccuguagaaccgagcuaguA....................................................................  2583     1   Fm2
....................uacccuguagaaccgagcuagCg....................................................................  3        1   Fm2
....................uacccuguagaaAcgagcuagug....................................................................  2        1   Fm2
....................uacccuguagaaccAagcuagug....................................................................  8        1   Fm2
....................uacccNguagaaccgagcuagug....................................................................  9        1   Fm2
....................uacccuguagaaccgagcuagAg....................................................................  7        1   Fm2
....................Gacccuguagaaccgagcuagug....................................................................  2        1   Fm2
....................uacccuguagaaccgagcuGgug....................................................................  70       1   Fm2
....................uacccuguagaaccgaUcuagug....................................................................  96       1   Fm2
....................uacccuguagaaccgagcuaAug....................................................................  6        1   Fm2
....................uacccuguaAaaccgagcuagug....................................................................  5        1   Fm2
....................uacccuguagaaccgagcuUgug....................................................................  8        1   Fm2
....................Aacccuguagaaccgagcuagug....................................................................  3        1   Fm2
....................uacccuguagaaccgagAuagug....................................................................  565      1   Fm2
....................uNcccuguagaaccgagcuagug....................................................................  1        1   Fm2
....................uacccuguagaaccgagcuCgug....................................................................  1        1   Fm2
....................uacccuguagaacUgagcuagug....................................................................  16       1   Fm2
....................uacccuguagaGccgagcuagug....................................................................  7        1   Fm2
....................uacccuguagaaccgCgcuagug....................................................................  1        1   Fm2
....................uacUcuguagaaccgagcuagug....................................................................  8        1   Fm2
....................uacccuguagaaccgagGuagug....................................................................  198      1   Fm2
....................uacccuCuagaaccgagcuagug....................................................................  5        1   Fm2
....................uacccuguagaaccgagcAagug....................................................................  5        1   Fm2
....................uaAccuguagaaccgagcuagug....................................................................  16       1   Fm2
....................uacccuUuagaaccgagcuagug....................................................................  1        1   Fm2
....................Cacccuguagaaccgagcuagug....................................................................  9        1   Fm2
....................uacccuguagCaccgagcuagug....................................................................  47       1   Fm2
....................uacccuguagaacAgagcuagug....................................................................  12       1   Fm2
....................uacccuguagaaccgagcuaUug....................................................................  5        1   Fm2
....................uacccugAagaaccgagcuagug....................................................................  3        1   Fm2
....................uaccAuguagaaccgagcuagug....................................................................  20       1   Fm2
....................uacccuNuagaaccgagcuagug....................................................................  3        1   Fm2
....................uacccuguagaaccgUgcuagug....................................................................  4        1   Fm2
....................uaUccuguagaaccgagcuagug....................................................................  8        1   Fm2
....................uaccUuguagaaccgagcuagug....................................................................  7        1   Fm2
....................uacccuguagaUccgagcuagug....................................................................  7        1   Fm2
....................uacccuguagaaccgagcuagGg....................................................................  15       1   Fm2
....................uacccuguagaaGcgagcuagug....................................................................  1        1   Fm2
....................uacccuguagaaccgagcCagug....................................................................  10       1   Fm2
....................uacccuguUgaaccgagcuagug....................................................................  12       1   Fm2
....................uaccGuguagaaccgagcuagug....................................................................  2        1   Fm2
....................uacccGguagaaccgagcuagug....................................................................  1        1   Fm2
....................uacccuguagaaccUagcuagug....................................................................  13       1   Fm2
....................uacccuguGgaaccgagcuagug....................................................................  10       1   Fm2
....................uacGcuguagaaccgagcuagug....................................................................  9        1   Fm2
....................uacccCguagaaccgagcuagug....................................................................  9        1   Fm2
....................uacccuguaUaaccgagcuagug....................................................................  4        1   Fm2
....................uacccuguagaaccgagcuaguC....................................................................  45       1   Fm2
....................uacccuguCgaaccgagcuagug....................................................................  1        1   Fm2
```

Mature | Star

```
aaauguggugaguuucugugcacccuguagaaccgagcuaguguaucgaguucgaaacucacaagccauucuacaguguacaaagaaacgucgcuucuuucaacaaugaga
```

```
aaauguggugaguuucuguguacccuguagaaccgagcuaguguaucgaguucgaaacucacaagccauucuacaguguacaaagaaacgucgcuucuuucaacaaugaga
....................uacccuguagaaccgagcuaguU....................................................................   22184    1   Fm2
....................uacccuguagGaccgagcuagug....................................................................   62       1   Fm2
....................uCcccuguagaaccgagcuagug....................................................................   3        1   Fm2
....................uGcccuguagaaccgagcuagug....................................................................   10       1   Fm2
....................uacccuguagaaccgaAcuagug....................................................................   124      1   Fm2
....................uUcccuguagaaccgagcuagug....................................................................   7        1   Fm2
....................Nacccuguagaaccgagcuagug....................................................................   25       1   Fm2
....................uacccuguagUaccgagcuagug....................................................................   62       1   Fm2
....................uacccuguagaaccgagcuagug....................................................................   53778    0   Fm2
....................uacccuAuagaaccgagcuagug....................................................................   7        1   Fm2
....................uacccugCagaaccgagcuagug....................................................................   11       1   Fm2
....................uacccuguagaaUcgagcuagug....................................................................   12       1   Fm2
....................uacccAguagaaccgagcuagug....................................................................   4        1   Fm2
....................uaGccuguagaaccgagcuagug....................................................................   5        1   Fm2
....................uacccuguagaaccgagcuaCug....................................................................   3        1   Fm2
....................uacccuguagaacGgagcuagug....................................................................   3        1   Fm2
....................uacccugGagaaccgagcuagug....................................................................   2        1   Fm2
....................uacccuguagaaccgaCcuagug....................................................................   92       1   Fm2
....................uacccuguagaaccgagUuagug....................................................................   237      1   Fm2
....................uacAcuguagaaccgagcuagug....................................................................   19       1   Fm2
....................uacccuguagaaccgGgcuagug....................................................................   5        1   Fm2
....................uacccuguaAaaccgagcuagugu...................................................................   6        1   Fm2
....................uacccuguagaaccgagcuaUugu...................................................................   12       1   Fm2
....................uaAccuguagaaccgagcuagugu...................................................................   34       1   Fm2
....................uacccuguagaaccgagcuagugA...................................................................   2679     1   Fm2
....................uacccuguagaUccgagcuagugu...................................................................   24       1   Fm2
....................uacccAguagaaccgagcuagugu...................................................................   10       1   Fm2
....................uacccuguagaaccgagAuagugu...................................................................   1453     1   Fm2
....................uacGcuguagaaccgagcuagugu...................................................................   18       1   Fm2
....................uacccCguagaaccgagcuagugu...................................................................   13       1   Fm2
....................uacccuguaCaaccgagcuagugu...................................................................   4        1   Fm2
....................uacccuUuagaaccgagcuagugu...................................................................   12       1   Fm2
....................uacccuguagaaUcgagcuagugu...................................................................   47       1   Fm2
....................uacccuguagaaccgagcuagGgu...................................................................   1        1   Fm2
....................Cacccuguagaaccgagcuagugu...................................................................   33       1   Fm2
....................uacccuguagaaccCagcuagugu...................................................................   1        1   Fm2
....................uacccuCuagaaccgagcuagugu...................................................................   13       1   Fm2
....................uaGccuguagaaccgagcuagugu...................................................................   49       1   Fm2
....................uacccuguagaaccUagcuagugu...................................................................   13       1   Fm2
....................uacccuguagaacAgagcuagugu...................................................................   30       1   Fm2
....................uacccuNuagaaccgagcuagugu...................................................................   13       1   Fm2
....................uaccUuguagaaccgagcuagugu...................................................................   17       1   Fm2
....................uUcccuguagaaccgagcuagugu...................................................................   34       1   Fm2
....................uacccuguagCaccgagcuagugu...................................................................   126      1   Fm2
....................uacccuguagaacGgagcuagugu...................................................................   4        1   Fm2
....................uacccuguagaaccgGgcuagugu...................................................................   15       1   Fm2
....................uacccuguagaaccAagcuagugu...................................................................   25       1   Fm2
....................uacccuguagaaccgaAcuagugu...................................................................   287      1   Fm2
....................uacccuguagUaccgagcuagugu...................................................................   149      1   Fm2
....................uaccAuguagaaccgagcuagugu...................................................................   28       1   Fm2
....................uacccuguaUaaccgagcuagugu...................................................................   18       1   Fm2
....................Aacccuguagaaccgagcuagugu...................................................................   15       1   Fm2
....................uacccuAuagaaccgagcuagugu...................................................................   23       1   Fm2
....................uacccugCagaaccgagcuagugu...................................................................   23       1   Fm2
....................Gacccuguagaaccgagcuagugu...................................................................   3        1   Fm2
....................uacccuguagaaccgagcuaguCu...................................................................   113      1   Fm2
....................uacccuguagaaccgagcuaguAu...................................................................   2930     1   Fm2
....................uacccuguagaaccgagcuaAugu...................................................................   6        1   Fm2
....................uCcccuguagaaccgagcuagugu...................................................................   9        1   Fm2
....................uacccuguUgaaccgagcuagugu...................................................................   29       1   Fm2
....................uacccuguagaaccgagGuagugu...................................................................   503      1   Fm2
....................uacccuguagaaccgaCcuagugu...................................................................   281      1   Fm2
....................uacccuguagaaccgagcuUgugu...................................................................   36       1   Fm2
....................uacccuguagaaccgagcuCgugu...................................................................   3        1   Fm2
....................uacccuguagaaccgagcuagCgu...................................................................   14       1   Fm2
....................uacccuguagaaccgUgcuagugu...................................................................   18       1   Fm2
....................uacccuguagaaccgagcuagugu...................................................................   146615   0   Fm2
....................uGcccuguagaaccgagcuagugu...................................................................   27       1   Fm2
....................uacccuguagaacUgagcuagugu...................................................................   59       1   Fm2
....................uacccuguagaaccgagcGagugu...................................................................   108      1   Fm2
```

```
                    Mature                                        Star
aaauguggugaguuucugugUACCCUGUAGAACCGAGCUAGUguaucgaguucgaaacucacaagccauucuacaguguacaaagaaacgucgcuucuuucaacaaugaga
```

```
aaauguggugaguuucuguguacccuguagaaccgagcuaguguaucgaguucgaaacucacaagccauucuacaguguacaaagaaacgucgcuucuuucaacaaugaga
....................uacccuguGgaaccgagcuagugu...................................................................    22     1   Fm2
....................uaccGuguagaaccgagcuagugu...................................................................    10     1   Fm2
....................uaUccuguagaaccgagcuagugu...................................................................    29     1   Fm2
....................uacccuguagaaGcgagcuagugu...................................................................    3      1   Fm2
....................uacccuguagaaccgaNcuagugu...................................................................    1      1   Fm2
....................uacccuguagaGccgagcuagugu...................................................................    17     1   Fm2
....................uNcccuguagaaccgagcuagugu...................................................................    3      1   Fm2
....................uacccuguagaCccgagcuagugu...................................................................    1      1   Fm2
....................uacccuguagGaccgagcuagugu...................................................................    164    1   Fm2
....................uacccuguagaaccgagcAagugu...................................................................    2      1   Fm2
....................uacccuguagaaccgagcCagugu...................................................................    19     1   Fm2
....................uacccugGagaaccgagcuagugu...................................................................    5      1   Fm2
....................uacccuguagaaAcgagcuagugu...................................................................    3      1   Fm2
....................Nacccuguagaaccgagcuagugu...................................................................    74     1   Fm2
....................uacccuguagaaccgagcuaCugu...................................................................    2      1   Fm2
....................uacccNguagaaccgagcuagugu...................................................................    24     1   Fm2
....................uacccuguagaaccgagcuGgugu...................................................................    320    1   Fm2
....................uacccuguagaaccgagUuagugu...................................................................    679    1   Fm2
....................uacccugAagaaccgagcuagugu...................................................................    9      1   Fm2
....................uacccuguCgaaccgagcuagugu...................................................................    3      1   Fm2
....................uacAcuguagaaccgagcuagugu...................................................................    60     1   Fm2
....................uacccuguagaaccgCgcuagugu...................................................................    2      1   Fm2
....................uacccuguagaaccgagcuagAgu...................................................................    10     1   Fm2
....................uacccuguagaaccgagcuaguUu...................................................................    4762   1   Fm2
....................uacccuguagaaccgaUcuagugu...................................................................    280    1   Fm2
....................uacccuguagaaccgagcuagugG...................................................................    15764  1   Fm2
....................uacccGguagaaccgagcuagugu...................................................................    6      1   Fm2
....................uacccuguagaaccgagcuagugC...................................................................    249    1   Fm2
....................uacUcuguagaaccgagcuagugu...................................................................    27     1   Fm2
....................uacccuguagaaccgagcuaguguC..................................................................    18     1   Fm2
....................uacccuguagaaccgagcuagugua..................................................................    57     0   Fm2
....................uacccuguagaaccgagcuagugAa..................................................................    54     1   Fm2
....................uGcccuguagaaccgagcuagugua..................................................................    1      1   Fm2
....................uacccuguagaaccgagcuaguguG..................................................................    29     1   Fm2
....................uacccuguagaaccgagcuaguguU..................................................................    6991   1   Fm2
....................uacccuguagaaccgagcuaguguaC.................................................................    1      1   Fm2
....................uacccuNuagaaccgagcuaguguau.................................................................    1      1   Fm2
....................uacccuguagaaccgagAuaguguau.................................................................    5      1   Fm2
....................uacccuguagaaccgagcuagugAau.................................................................    24     1   Fm2
....................uacccuguagaaccgagUuaguguau.................................................................    1      1   Fm2
....................uacccuguagaaccgagcuaguguau.................................................................    201    0   Fm2
....................uacccuguagaaccgagcuaguguCu.................................................................    2      1   Fm2
....................uacccuguagaaccgagcuaguguUu.................................................................    92     1   Fm2
....................uacccuguagaaccgagcuaguguauU................................................................    9      1   Fm2
....................uacccuguagaaccgagAuaguguauc................................................................    3      1   Fm2
....................uacccuguagGaccgagcuaguguauc................................................................    1      1   Fm2
....................uacccuguagaaccgagUuaguguauc................................................................    3      1   Fm2
....................uacccuguagaaccgaCcuaguguauc................................................................    2      1   Fm2
....................Cacccuguagaaccgagcuaguguauc................................................................    1      1   Fm2
....................uacccuguagaaccgagcuaguguauc................................................................    608    0   Fm2
....................uacccuguagaaccgagGuaguguauc................................................................    3      1   Fm2
....................uaccUuguagaaccgagcuaguguauc................................................................    1      1   Fm2
....................uacccuguagaaccgagcuaguAuauc................................................................    1      1   Fm2
....................uacccuguagaaccgagcuagAguauc................................................................    1      1   Fm2
....................uacccuguCgaaccgagcuaguguauc................................................................    1      1   Fm2
....................uacccuguagaaccgagcuaguUuauc................................................................    1      1   Fm2
....................uacccuguagaaccgagcuaguguaGc................................................................    2      1   Fm2
....................uacccuguagaaccgagcuaguguaAc................................................................    1      1   Fm2
....................uacccuguagUaccgagcuaguguauc................................................................    1      1   Fm2
....................uacccuguagaacUgagcuaguguauc................................................................    1      1   Fm2
....................uacccuguagCaccgagcuaguguauc................................................................    1      1   Fm2
....................Nacccuguagaaccgagcuaguguauc................................................................    1      1   Fm2
....................uacccuguagaaccAagcuaguguaucg...............................................................    1      1   Fm2
....................uacccuguagaaccgagcuaguguaucU...............................................................    416    1   Fm2
....................uacccuguagaaccgagcuaguguaucg...............................................................    16     0   Fm2
....................uacccuguagaaccgagcuaguguaucgagu............................................................    15     0   Fm2
....................uacccuguagaaccgagcuaguguaucgaguucgaaacucacaagccU...........................................    1      1   Fm2
.....................acccuguagaaccgagcu........................................................................    17     0   Fm2
.....................acccuguagaaccgagcuag......................................................................    56     0   Fm2
.....................acccuguagaaccgaUcuag......................................................................    1      1   Fm2
```

```
                     Mature                  Star
aaaugguggugaguuucugugUACCCUGUAGAACCGAGCUAGUguaucgaguucgaaacucaCAAGCCAUUCUACAGUGUACAaagaaacgucgcuucuuucaacaaugaga
......................acccuguagaaccgagAuag......................................................................	1	1	Fm2
......................aGccuguagaaccgagcuag......................................................................	1	1	Fm2
......................acccuguagaaccgagcCag......................................................................	1	1	Fm2
......................aAccuguagaaccgagcuag......................................................................	1	1	Fm2
......................acccuguagaaccgagcuaU......................................................................	1	1	Fm2
......................acccuguagaaccgaUcuagu.....................................................................	2	1	Fm2
......................acccuguCgaaccgagcuagu.....................................................................	1	1	Fm2
......................acccuguagaaccgagcuaUu.....................................................................	7	1	Fm2
......................acccuguaCaaccgagcuagu.....................................................................	5	1	Fm2
......................acccuguagaaccgCgcuagu.....................................................................	3	1	Fm2
......................acccuguagaaccgagcuCgu.....................................................................	6	1	Fm2
......................aUccuguagaaccgagcuagu.....................................................................	1	1	Fm2
......................acccuguagaaccgagcuagu.....................................................................	4395	0	Fm2
......................acccuguagaGccgagcuagu.....................................................................	13	1	Fm2
......................acccuguagaaccCagcuagu.....................................................................	3	1	Fm2
......................aGccuguagaaccgagcuagu.....................................................................	1	1	Fm2
......................acccuguagaaccgagUuagu.....................................................................	1	1	Fm2
......................acccuguagaaccgagcCagu.....................................................................	24	1	Fm2
......................acccuguagaaccgagcuagA.....................................................................	2	1	Fm2
......................acccuguagaaccgagcAagu.....................................................................	12	1	Fm2
......................accAuguagaaccgagcuagu.....................................................................	2	1	Fm2
......................acccuguagaaccgagcuUgu.....................................................................	3	1	Fm2
......................acccuNuagaaccgagcuagu.....................................................................	2	1	Fm2
......................acccuguagaCccgagcuagu.....................................................................	9	1	Fm2
......................acccuguagaaccgaAcuagu.....................................................................	1	1	Fm2
......................acccuguGgaaccgagcuagu.....................................................................	1	1	Fm2
......................acccuguagaaccAagcuagu.....................................................................	4	1	Fm2
......................acccuguagaUccgagcuagu.....................................................................	7	1	Fm2
......................acAcuguagaaccgagcuagu.....................................................................	3	1	Fm2
......................acccuguaUaaccgagcuagu.....................................................................	3	1	Fm2
......................acccugCagaaccgagcuagu.....................................................................	4	1	Fm2
......................acccuguagaaccgaCcuagu.....................................................................	2	1	Fm2
......................acccuguaAaaccgagcuagu.....................................................................	2	1	Fm2
......................acccuguagaacGgagcuagu.....................................................................	2	1	Fm2
......................acccuguagaacAgagcuagu.....................................................................	8	1	Fm2
......................acccuUuagaaccgagcuagu.....................................................................	1	1	Fm2
......................acccuguagaaccgagGuagu.....................................................................	2	1	Fm2
......................acGcuguagaaccgagcuagu.....................................................................	5	1	Fm2
......................acccuAuagaaccgagcuagu.....................................................................	2	1	Fm2
......................acccuguagaaccgagcuaCu.....................................................................	5	1	Fm2
......................acccuguagaaccgUgcuagu.....................................................................	2	1	Fm2
......................acUcuguagaaccgagcuagu.....................................................................	3	1	Fm2
......................acccuguagaaccgagAuagu.....................................................................	1	1	Fm2
......................acccuguagaaAcgagcuagu.....................................................................	1	1	Fm2
......................Ncccuguagaaccgagcuagu.....................................................................	1	1	Fm2
......................acccCguagaaccgagcuagu.....................................................................	1	1	Fm2
......................acccugAagaaccgagcuagu.....................................................................	3	1	Fm2
......................acccuguagaaccgagcuaAu.....................................................................	4	1	Fm2
......................acccuguagaaUcgagcuagu.....................................................................	3	1	Fm2
......................acccuguagaaccgagcuagG.....................................................................	9	1	Fm2
......................acccuguagaaccgagcuagC.....................................................................	1	1	Fm2
......................acccuguagaacUgagcuagu.....................................................................	4	1	Fm2
......................Ucccuguagaaccgagcuagu.....................................................................	4	1	Fm2
......................acccuguagaaccgagcuagug....................................................................	117	0	Fm2
......................acccuguagaaccgagcuaguU....................................................................	67	1	Fm2
......................aGccuguagaaccgagcuagug....................................................................	1	1	Fm2
......................acccuguagaaccgagcuaguA....................................................................	16	1	Fm2
......................acccuguagaaccgagcAagug....................................................................	1	1	Fm2
......................acccuguagCaccgagcuagug....................................................................	1	1	Fm2
......................acAcuguagaaccgagcuagugu...................................................................	1	1	Fm2
......................acccuguagaaccgagUuagugu...................................................................	1	1	Fm2
......................acccuguagaaccgagAuagugu...................................................................	4	1	Fm2
......................acccuguagaacAgagcuagugu...................................................................	1	1	Fm2
......................acccuguagaaccUagcuagugu...................................................................	1	1	Fm2
......................acccuguagaaccgagcAagugu...................................................................	1	1	Fm2
......................acccuguagaaccgagcGagugu...................................................................	1	1	Fm2
......................acccuguagaaccgagcuagugu...................................................................	631	0	Fm2
......................acccuguagUaccgagcuagugu...................................................................	1	1	Fm2
......................acccuguagaaccgagcuaguAu...................................................................	14	1	Fm2
......................Gcccuguagaaccgagcuagugu...................................................................	1	1	Fm2
```

```
                    Mature                                   Star
aaauguggugaguuucuguguacccuguagaaccgagcuaguguaucgaguucgaaacucacaagccauucuacaguguacaaagaaacgucgcuucuuucaacaaugaga
.....................acccuguagaaccgagcuGgugu...................................................................    6     1    Fm2
.....................acccuguaCaaccgagcuagugu...................................................................    1     1    Fm2
.....................acccCguagaaccgagcuagugu...................................................................    2     1    Fm2
.....................acccuguagaaccAagcuagugu...................................................................    1     1    Fm2
.....................acccuguagaaccgagcuagugC...................................................................    2     1    Fm2
.....................acccuguagaaccgagcCagugu...................................................................    5     1    Fm2
.....................acUcuguagaaccgagcuagugu...................................................................    1     1    Fm2
.....................acccuguagaCccgagcuagugu...................................................................    2     1    Fm2
.....................acccuguagaaccgagcuaguUu...................................................................    9     1    Fm2
.....................acccuguagaaccgagcuagugA...................................................................    31    1    Fm2
.....................acccuguagaaUcgagcuagugu...................................................................    1     1    Fm2
.....................acccuguagaaccgagcuagugG...................................................................    71    1    Fm2
.....................acccuguagaaccgagcuaguguU..................................................................    32    1    Fm2
.....................acccuguagaaccgagcuaguguaucgagu............................................................    15    0    Fm2
.....................acccuguagaUccgagcuaguguaucgagu............................................................    1     1    Fm2
......................cccuguagaaccgagcuaU......................................................................    1     1    Fm2
......................cccuguagaaccgagcuag......................................................................    1     0    Fm2
......................cccuguagaaccgagUuagu.....................................................................    1     1    Fm2
......................Gccuguagaaccgagcuagu.....................................................................    1     1    Fm2
......................cccuguagaaccgagcuagu.....................................................................    103   0    Fm2
......................Uccuguagaaccgagcuagu.....................................................................    1     1    Fm2
......................cccuguagaaccgaCcuagu.....................................................................    1     1    Fm2
......................cccuguagaaccgagcuCgu.....................................................................    1     1    Fm2
......................cccuguagaaccgagcuagG.....................................................................    1     1    Fm2
......................cccuguagaaccgagcuagA.....................................................................    1     1    Fm2
......................cccugAagaaccgagcuagu.....................................................................    2     1    Fm2
......................cccuguagaaccgagcuaUu.....................................................................    6     1    Fm2
......................cccuguagaaccgagcuagug....................................................................    2     0    Fm2
......................cccuguagaaccgagcuaguA....................................................................    2     1    Fm2
......................cccuguagaaccgagcuagugu...................................................................    12    0    Fm2
......................cccuguagaaccgagcuagugG...................................................................    1     1    Fm2
......................cccuguagaaccgagcuaguUu...................................................................    3     1    Fm2
.......................ccuguaUaaccgagcuagu.....................................................................    1     1    Fm2
.......................ccuguagaaccgagcuagu.....................................................................    11    0    Fm2
.......................ccuguagaaccgagcuagugu...................................................................    1     0    Fm2
..........................guagaaccgagcuagugu...................................................................    1     0    Fm2
..........................guagaaccgagcuagugG...................................................................    1     1    Fm2
.............................................................caagccauucuacagugu................................    21    0    Fm2
.............................................................caagccauucuacagugG................................    1     1    Fm2
.............................................................caagccauucuacagugAa...............................    2     1    Fm2
.............................................................caagccauucGacaguguac..............................    1     1    Fm2
.............................................................caagccauucuacagugGac..............................    4     1    Fm2
.............................................................caagccauucuacaguguac..............................    478   0    Fm2
.............................................................caagccauAcuacaguguac..............................    1     1    Fm2
.............................................................caagccauucuacagugCac..............................    3     1    Fm2
.............................................................caagccauucAacaguguac..............................    1     1    Fm2
.............................................................caagccauucuacagugAac..............................    1     1    Fm2
.............................................................caagccauucuacaguCuac..............................    1     1    Fm2
.............................................................Uaagccauucuacaguguac..............................    1     1    Fm2
.............................................................caagccauucuaGaguguaca.............................    1     1    Fm2
.............................................................caagAcauucuacaguguaca.............................    2     1    Fm2
.............................................................caagccauucuCcaguguaca.............................    1     1    Fm2
.............................................................caagccUuucuacaguguaca.............................    2     1    Fm2
.............................................................caagcUauucuacaguguaca.............................    3     1    Fm2
.............................................................caagccaCucuacaguguaca.............................    1     1    Fm2
.............................................................caagccauAcuacaguguaca.............................    1     1    Fm2
.............................................................caagccauuUuacaguguaca.............................    6     1    Fm2
.............................................................caagccauucCacaguguaca.............................    4     1    Fm2
.............................................................caagccauucuacaguguacU.............................    12    1    Fm2
.............................................................caagccauucuacagAguaca.............................    1     1    Fm2
.............................................................caagccaAucuacaguguaca.............................    1     1    Fm2
.............................................................caagccauucAacaguguaca.............................    5     1    Fm2
.............................................................caagccauucuacaguguaca.............................    4465  0    Fm2
.............................................................caagccauucuacaguAuaca.............................    13    1    Fm2
.............................................................caagccauucuacaguguacG.............................    3     1    Fm2
.............................................................caaAccauucuacaguguaca.............................    2     1    Fm2
.............................................................Aaagccauucuacaguguaca.............................    1     1    Fm2
.............................................................caaUccauucuacaguguaca.............................    1     1    Fm2
.............................................................caagccauucuacaguCuaca.............................    15    1    Fm2
.............................................................caUgccauucuacaguguaca.............................    2     1    Fm2
```

```
                    Mature                                      Star
aaauguggugaguuucugugtuacccuguagaaccgagcuaguguaucgaguucgaaacucacaagccauucuacaguguacaaagaaacgucgcuucuuucaacaaugaga
```

Mature **Star**

```
aaauguggugaguuucugugUACCCUGUAGAACCGAGCUAGUguaucgaguucgaaacucacaagccauucuacaguguacaaagaaacgucgcuucuuucaacaaugaga
................uCuguacccuguagaaccgagcuagu.....................................................................  1     1  Fm1
.................Cuguacccuguagaaccgagcuag......................................................................  3     1  Fm1
.................Cuguacccuguagaaccgagcuagu.....................................................................  24    1  Fm1
.................guguacccuguagaaccgagcuagu.....................................................................  6     0  Fm1
..................uguacccuguagaaccga...........................................................................  13    0  Fm1
..................uguacccuguagaaccgG...........................................................................  1     1  Fm1
..................uguacccuguagaaccgaA..........................................................................  5     1  Fm1
..................uguacccuguagaaccgag..........................................................................  28    0  Fm1
..................uguacAcuguagaaccgagc.........................................................................  1     1  Fm1
..................uguacGcuguagaaccgagc.........................................................................  1     1  Fm1
..................uguacccuguagaaccgCgc.........................................................................  4     1  Fm1
..................uguacccuguagaaccgagU.........................................................................  6     1  Fm1
..................uguacccuguagaacGgagc.........................................................................  1     1  Fm1
..................uguacccuguagaaccgagG.........................................................................  1     1  Fm1
..................uguacccuguagaaccAagc.........................................................................  1     1  Fm1
..................uguacccuguCgaaccgagc.........................................................................  1     1  Fm1
..................uguacccuguagaaccgUgc.........................................................................  4     1  Fm1
..................uguacccuguagaaccgagc.........................................................................  808   0  Fm1
..................Cguacccuguagaaccgagc.........................................................................  1     1  Fm1
..................uguacccuguagaaccgGgc.........................................................................  1     1  Fm1
..................uguacccCguagaaccgagc.........................................................................  1     1  Fm1
..................uguaccUuguagaaccgagc.........................................................................  1     1  Fm1
..................uguacccuguagaaccUagc.........................................................................  2     1  Fm1
..................uguacUcuguagaaccgagcu........................................................................  1     1  Fm1
..................uguacccuguagaaccgGgcu........................................................................  11    1  Fm1
..................uguacccuguagaaccgagcA........................................................................  8     1  Fm1
..................uguacccuguagaaccgUgcu........................................................................  12    1  Fm1
..................Nguacccuguagaaccgagcu........................................................................  1     1  Fm1
..................uguacccuguaAaaccgagcu........................................................................  2     1  Fm1
..................uguacccuguagaaccgagcG........................................................................  23    1  Fm1
..................uguacccuguGgaaccgagcu........................................................................  6     1  Fm1
..................uguacccuguaUaaccgagcu........................................................................  1     1  Fm1
..................uguacccuguagaacAgagcu........................................................................  1     1  Fm1
..................uguacccuguagaaccgCgcu........................................................................  19    1  Fm1
..................uAuacccuguagaaccgagcu........................................................................  3     1  Fm1
..................uguacccuguagaaccUagcu........................................................................  5     1  Fm1
..................uguacccuguCgaaccgagcu........................................................................  4     1  Fm1
..................uguacccugCagaaccgagcu........................................................................  2     1  Fm1
..................Cguacccuguagaaccgagcu........................................................................  1     1  Fm1
..................uguacccuguagaacGgagcu........................................................................  1     1  Fm1
..................uguacccuguagaaccAagcu........................................................................  6     1  Fm1
..................uguacccuguagaaccgagAu........................................................................  3     1  Fm1
..................uguacccCguagaaccgagcu........................................................................  2     1  Fm1
..................uguacccuguUgaaccgagcu........................................................................  4     1  Fm1
..................uguacccuguagaaccgagcu........................................................................  3435  0  Fm1
..................uguacccuguagaaccCagcu........................................................................  8     1  Fm1
..................uguacNcuguagaaccgagcu........................................................................  1     1  Fm1
..................uguacccuUuagaaccgagcua.......................................................................  1     1  Fm1
..................uguacccuguagaaccgCgcua.......................................................................  4     1  Fm1
..................uguacccuguCgaaccgagcua.......................................................................  1     1  Fm1
..................uguacccuguagaaccgUgcua.......................................................................  3     1  Fm1
..................uguUcccuguagaaccgagcua.......................................................................  1     1  Fm1
..................uguaAccuguagaaccgagcua.......................................................................  1     1  Fm1
..................uguacccuguGgaaccgagcua.......................................................................  1     1  Fm1
..................uguacccuguagaaccgagcuG.......................................................................  1     1  Fm1
..................uguaccAuguagaaccgagcua.......................................................................  1     1  Fm1
..................uguacccuguagaaccgagcuU.......................................................................  97    1  Fm1
..................uguacccuguagaaccgagcua.......................................................................  654   0  Fm1
..................Nguacccuguagaaccgagcua.......................................................................  1     1  Fm1
..................uguacccuguagaaccCagcua.......................................................................  1     1  Fm1
..................uguacNcuguagaaccgagcuag......................................................................  2     1  Fm1
..................uguacccuguagaaccgagGuag......................................................................  1     1  Fm1
..................uguacUcuguagaaccgagcuag......................................................................  10    1  Fm1
..................uguacccuguagaaccUagcuag......................................................................  13    1  Fm1
..................uguacccuguaUaaccgagcuag......................................................................  2     1  Fm1
..................uguacccuUuagaaccgagcuag......................................................................  1     1  Fm1
..................uguacccuguagaaccgUgcuag......................................................................  42    1  Fm1
..................uguacccuguagaaccgagcuaA......................................................................  26    1  Fm1
..................uguacccuguagaaccAagcuag......................................................................  19    1  Fm1
..................uguacccuguagaacAgagcuag......................................................................  1     1  Fm1
```

Mature Star

```
aaauguggugaguuucugugguacccuguagaaccgagcuaguguaucgaguucgaaacucacaagccauucuacaguguacaaagaaacgucgcuucuuucaacaaugaga
..................uguacccuguagaaccgCgcuag......................................................................  46    1  Fm1
..................uguacccuguagaaccgagUuag......................................................................  4     1  Fm1
..................uguacccugGagaaccgagcuag......................................................................  1     1  Fm1
..................uguaGccuguagaaccgagcuag......................................................................  2     1  Fm1
..................uguacccuguagaaccgaCcuag......................................................................  1     1  Fm1
..................uguacccuguagaUccgagcuag......................................................................  1     1  Fm1
..................uguacccuguagaaccCagcuag......................................................................  21    1  Fm1
..................uguacccuguagaaccgagAuag......................................................................  2     1  Fm1
..................Nguacccuguagaaccgagcuag......................................................................  1     1  Fm1
..................uCuacccuguagaaccgagcuag......................................................................  1     1  Fm1
..................uguacccuguagCaccgagcuag......................................................................  1     1  Fm1
..................ugCacccuguagaaccgagcuag......................................................................  2     1  Fm1
..................uguacccuAuagaaccgagcuag......................................................................  3     1  Fm1
..................uguacccuguagaaccgGgcuag......................................................................  27    1  Fm1
..................Aguacccuguagaaccgagcuag......................................................................  2     1  Fm1
..................uguacccuguagaaccgaAcuag......................................................................  1     1  Fm1
..................uguacccuguagaaGcgagcuag......................................................................  1     1  Fm1
..................uguacccuguagaaccgagcuaU......................................................................  432   1  Fm1
..................ugGacccuguagaaccgagcuag......................................................................  1     1  Fm1
..................uguacccuguagaaccgaUcuag......................................................................  8     1  Fm1
..................uguacccuguagaaccgagcGag......................................................................  2     1  Fm1
..................uguCcccuguagaaccgagcuag......................................................................  1     1  Fm1
..................uguaccGuguagaaccgagcuag......................................................................  2     1  Fm1
..................uguacccuguagaaUcgagcuag......................................................................  1     1  Fm1
..................uguacGcuguagaaccgagcuag......................................................................  1     1  Fm1
..................uguacccCguagaaccgagcuag......................................................................  3     1  Fm1
..................uguaUccuguagaaccgagcuag......................................................................  2     1  Fm1
..................uguacccuguUgaaccgagcuag......................................................................  4     1  Fm1
..................uguacccuguagaaAcgagcuag......................................................................  1     1  Fm1
..................uguacccuguagaaccgagcCag......................................................................  3     1  Fm1
..................uguacccuguGgaaccgagcuag......................................................................  12    1  Fm1
..................uguacccugAagaaccgagcuag......................................................................  2     1  Fm1
..................uguacccuguagaaccgagcuag......................................................................  9555  0  Fm1
..................uguacccuguagaaccgagcuUg......................................................................  1     1  Fm1
..................uAuacccuguagaaccgagcuag......................................................................  7     1  Fm1
..................uguacccuguagaaccgagcuGg......................................................................  29    1  Fm1
..................uguacccuguagGaccgagcuag......................................................................  1     1  Fm1
..................Cguacccuguagaaccgagcuag......................................................................  1     1  Fm1
..................uguacccugCagaaccgagcuag......................................................................  2     1  Fm1
..................uUuacccuguagaaccgagcuag......................................................................  1     1  Fm1
..................uguaAccuguagaaccgagcuag......................................................................  4     1  Fm1
..................uguacccuguagaaccgagcuCg......................................................................  5     1  Fm1
..................uguacccuguCgaaccgagcuag......................................................................  9     1  Fm1
..................uguacccuguagaaccgagcuaC......................................................................  2     1  Fm1
..................uguacccAguagaaccgagcuag......................................................................  1     1  Fm1
..................uguacccuguagaacUgagcuag......................................................................  3     1  Fm1
..................uguacccuguagaaccgagcuagA.....................................................................  192   1  Fm1
..................uCuacccuguagaaccgagcuagu.....................................................................  39    1  Fm1
..................uguacccuCuagaaccgagcuagu.....................................................................  5     1  Fm1
..................uguacccuguaAaaccgagcuagu.....................................................................  8     1  Fm1
..................uguacccuguagaaccgagcCagu.....................................................................  19    1  Fm1
..................uguacccuguagaCccgagcuagu.....................................................................  4     1  Fm1
..................uguacccuguCgaaccgagcuagu.....................................................................  123   1  Fm1
..................uguaccAuguagaaccgagcuagu.....................................................................  16    1  Fm1
..................Gguacccuguagaaccgagcuagu.....................................................................  9     1  Fm1
..................uguacccuguagaaccgagcuaAu.....................................................................  27    1  Fm1
..................uguaGccuguagaaccgagcuagu.....................................................................  15    1  Fm1
..................uguacccuguagaUccgagcuagu.....................................................................  28    1  Fm1
..................uguacccuguagaaccgGgcuagu.....................................................................  351   1  Fm1
..................uguaccNuguagaaccgagcuagu.....................................................................  5     1  Fm1
..................uguacccuguagaaccUagcuagu.....................................................................  200   1  Fm1
..................uguacccuguagaaccgagcuagC.....................................................................  50    1  Fm1
..................uguacccGguagaaccgagcuagu.....................................................................  3     1  Fm1
..................uguaccGuguagaaccgagcuagu.....................................................................  2     1  Fm1
..................uguacccuguagaaccgNgcuagu.....................................................................  1     1  Fm1
..................uguacccuguagaaccgagcuCgu.....................................................................  8     1  Fm1
..................uguaccUuguagaaccgagcuagu.....................................................................  22    1  Fm1
..................uguacccuguagaGccgagcuagu.....................................................................  17    1  Fm1
..................uguacccuAuagaaccgagcuagu.....................................................................  10    1  Fm1
..................uguacccuguagaaccgCgcuagu.....................................................................  597   1  Fm1
```

```
                    Mature                        Star
aaauguggugaguuucuguguacccuguagaaccgagcuaguguaucgaguucgaaacucacaagccauucuacaguguacaaagaaacgucgcuucuuucaacaaugaga
.................uguacccuguagaaccgagAuagu......................................................................  12      1  Fm1
.................uguacccuguagaaccCagcuagu......................................................................  273     1  Fm1
.................uguacccuguagaaccgagcGagu......................................................................  14      1  Fm1
.................uguacccuguagaacGgagcuagu......................................................................  3       1  Fm1
.................uguacccuguagUaccgagcuagu......................................................................  14      1  Fm1
.................uguacccugCagaaccgagcuagu......................................................................  26      1  Fm1
.................uguacccuguGgaaccgagcuagu......................................................................  170     1  Fm1
.................ugCacccuguagaaccgagcuagu......................................................................  25      1  Fm1
.................uguacccuguagaaAcgagcuagu......................................................................  9       1  Fm1
.................uguacccuguagGaccgagcuagu......................................................................  15      1  Fm1
.................Cguacccuguagaaccgagcuagu......................................................................  30      1  Fm1
.................ugAacccuguagaaccgagcuagu......................................................................  24      1  Fm1
.................uguacccuguagaaccgaAcuagu......................................................................  8       1  Fm1
.................uguacccuguagaaGcgagcuagu......................................................................  4       1  Fm1
.................uguacccuguagaaccgagcuaUu......................................................................  84      1  Fm1
.................uguaUccuguagaaccgagcuagu......................................................................  18      1  Fm1
.................uguacccuguagaaccgaUcuagu......................................................................  42      1  Fm1
.................uguacccuguagaaccgagGuagu......................................................................  3       1  Fm1
.................uguacUcuguagaaccgagcuagu......................................................................  126     1  Fm1
.................uguacccuguUgaaccgagcuagu......................................................................  98      1  Fm1
.................ugGacccuguagaaccgagcuagu......................................................................  24      1  Fm1
.................uguacNcuguagaaccgagcuagu......................................................................  18      1  Fm1
.................uguacccuguagaaccgagcuagu......................................................................  133850  0  Fm1
.................uguacccugGagaaccgagcuagu......................................................................  7       1  Fm1
.................uguacccuguagaacAgagcuagu......................................................................  13      1  Fm1
.................uguacccuUuagaaccgagcuagu......................................................................  12      1  Fm1
.................uguacccuguagaaccAagcuagu......................................................................  310     1  Fm1
.................uguacccuguagaaUcgagcuagu......................................................................  17      1  Fm1
.................Nguacccuguagaaccgagcuagu......................................................................  66      1  Fm1
.................uguacccuguagaaccgagUuagu......................................................................  59      1  Fm1
.................uguacccuguagaacUgagcuagu......................................................................  45      1  Fm1
.................uAuacccuguagaaccgagcuagu......................................................................  55      1  Fm1
.................uguCcccuguagaaccgagcuagu......................................................................  9       1  Fm1
.................uguacccugAagaaccgagcuagu......................................................................  8       1  Fm1
.................uguacccuguaCaaccgagcuagu......................................................................  4       1  Fm1
.................uguacccCguagaaccgagcuagu......................................................................  21      1  Fm1
.................uguacAcuguagaaccgagcuagu......................................................................  21      1  Fm1
.................uUuacccuguagaaccgagcuagu......................................................................  29      1  Fm1
.................uguUcccuguagaaccgagcuagu......................................................................  9       1  Fm1
.................uguacccuguagaaccgagcAagu......................................................................  4       1  Fm1
.................uguacccuguagaaccgagcuGgu......................................................................  149     1  Fm1
.................uguacccuguaUaaccgagcuagu......................................................................  11      1  Fm1
.................Aguacccuguagaaccgagcuagu......................................................................  16      1  Fm1
.................uguGcccuguagaaccgagcuagu......................................................................  8       1  Fm1
.................uguaAccuguagaaccgagcuagu......................................................................  20      1  Fm1
.................uguacccuguagaaccgagcuagG......................................................................  267     1  Fm1
.................uguacccAguagaaccgagcuagu......................................................................  2       1  Fm1
.................uguacccuguagaaccgagcuaCu......................................................................  2       1  Fm1
.................uguacccuguagaaccgUgcuagu......................................................................  529     1  Fm1
.................uguacGcuguagaaccgagcuagu......................................................................  10      1  Fm1
.................uguacccuguagaaccgagcuUgu......................................................................  21      1  Fm1
.................uguacccuguagaaccgaCcuagu......................................................................  11      1  Fm1
.................uguacccuguagCaccgagcuagu......................................................................  5       1  Fm1
.................uguacccuguagaaccgagcuaguU.....................................................................  4574    1  Fm1
.................uguacccuguagaaccgagcuaAug.....................................................................  1       1  Fm1
.................uguacccuguagaaccCagcuagug.....................................................................  3       1  Fm1
.................uguacccuguagaaccgagcuagGg.....................................................................  1       1  Fm1
.................uguacccuguGgaaccgagcuagug.....................................................................  3       1  Fm1
.................uguacccugCagaaccgagcuagug.....................................................................  1       1  Fm1
.................uguacccuguCgaaccgagcuagug.....................................................................  4       1  Fm1
.................uguacccAguagaaccgagcuagug.....................................................................  1       1  Fm1
.................uguacccuguagaacUgagcuagug.....................................................................  1       1  Fm1
.................uguacccuguUgaaccgagcuagug.....................................................................  1       1  Fm1
.................uguacccuguagaUccgagcuagug.....................................................................  1       1  Fm1
.................uguacccuguagaaccAagcuagug.....................................................................  5       1  Fm1
.................uguacccuguagaaccgagcuaguA.....................................................................  49      1  Fm1
.................uguacUcuguagaaccgagcuagug.....................................................................  6       1  Fm1
.................Nguacccuguagaaccgagcuagug.....................................................................  2       1  Fm1
.................uNuacccuguagaaccgagcuagug.....................................................................  1       1  Fm1
.................uguacccuguagaaccgCgcuagug.....................................................................  7       1  Fm1
```

Mature **Star**

```
aaauguggugaguuucuguguacccuguagaaccgagcuaguguaucgaguucgaaacucacaagccauucuacaguguacaaagaaacgucgcuucuuucaacaaugaga
..................uguacccuguagaaccgagcuagug....................................................................  1891  0  Fm1
..................uUuacccuguagaaccgagcuagug....................................................................  4     1  Fm1
..................uguaccAuguagaaccgagcuagug....................................................................  1     1  Fm1
..................uguacccuguagaaccgUgcuagug....................................................................  8     1  Fm1
..................ugAacccuguagaaccgagcuagug....................................................................  1     1  Fm1
..................uguacNcuguagaaccgagcuagug....................................................................  1     1  Fm1
..................uguacccuguagaaccUagcuagug....................................................................  3     1  Fm1
..................uguacccuguagaaccgagcuaguC....................................................................  12    1  Fm1
..................uguacccuUuagaaccgagcuagug....................................................................  1     1  Fm1
..................uguacccuguagaaUcgagcuagug....................................................................  1     1  Fm1
..................uguacccuguagaaccgGgcuagug....................................................................  2     1  Fm1
..................uguaccUuguagaaccgagcuagug....................................................................  1     1  Fm1
..................uguacccuAuagaaccgagcuagug....................................................................  1     1  Fm1
..................uguacccuguagaaccCagcuagugu...................................................................  4     1  Fm1
..................uguacUcuguagaaccgagcuagugu...................................................................  1     1  Fm1
..................uguacccAguagaaccgagcuagugu...................................................................  1     1  Fm1
..................uguacccuguagaaccgagcuaguUu...................................................................  267   1  Fm1
..................uguacccuguagaaccgagcuaguCu...................................................................  1     1  Fm1
..................uguacccuguagaacUgagcuagugu...................................................................  1     1  Fm1
..................uguacccuguagaaccgGgcuagugu...................................................................  2     1  Fm1
..................uguacccuguagaaccgagcuaguAu...................................................................  27    1  Fm1
..................uguacccuguUgaaccgagcuagugu...................................................................  1     1  Fm1
..................uguacccuguCgaaccgagcuagugu...................................................................  1     1  Fm1
..................uguacNcuguagaaccgagcuagugu...................................................................  1     1  Fm1
..................uguacccuguagaaccgagcuagugG...................................................................  149   1  Fm1
..................ugGacccuguagaaccgagcuagugu...................................................................  1     1  Fm1
..................uguacccuguagaaccgaAcuagugu...................................................................  1     1  Fm1
..................uguacccCguagaaccgagcuagugu...................................................................  1     1  Fm1
..................uguacccuguagaaccAagcuagugu...................................................................  2     1  Fm1
..................uguacccuguagaaccgagcuagAgu...................................................................  2     1  Fm1
..................uguacccuguagaaccgagcuagugA...................................................................  22    1  Fm1
..................uguacccuguagaaccUagcuagugu...................................................................  2     1  Fm1
..................Cguacccuguagaaccgagcuagugu...................................................................  1     1  Fm1
..................uguacccuguagaaccgagcuGgugu...................................................................  7     1  Fm1
..................uguacccuguagaaccgagcCagugu...................................................................  1     1  Fm1
..................uguacccuguagaaccgagcuagugu...................................................................  1193  0  Fm1
..................uguacccuguagaaccgUgcuagugu...................................................................  6     1  Fm1
..................uAuacccuguagaaccgagcuagugu...................................................................  1     1  Fm1
..................uguacccuguagaaccgCgcuagugu...................................................................  4     1  Fm1
..................Nguacccuguagaaccgagcuagugu...................................................................  1     1  Fm1
..................uguacccuguagaaccgagcuagugua..................................................................  2     0  Fm1
..................uguacccuguagaaccgagcuaguguU..................................................................  94    1  Fm1
..................uguacccuguagaaccgagcuaguguaucgagu............................................................  1     0  Fm1
..................uguacccuguagaaccgagcuaguguaucgaguu...........................................................  1     0  Fm1
...................Nuacccuguagaaccgagc.........................................................................  3     1  Fm1
...................Uuacccuguagaaccgagc.........................................................................  12    1  Fm1
...................guacccuguagaaccgagA.........................................................................  4     1  Fm1
...................Cuacccuguagaaccgagc.........................................................................  3     1  Fm1
...................guacccuguagaaccgagc.........................................................................  43    0  Fm1
...................guacAcuguagaaccgagc.........................................................................  1     1  Fm1
...................guacccuguagaaccgaUcu........................................................................  1     1  Fm1
...................guacccuguagaaccgaCcu........................................................................  1     1  Fm1
...................guacccuguagaaccgCgcu........................................................................  2     1  Fm1
...................Uuacccuguagaaccgagcu........................................................................  1     1  Fm1
...................Auacccuguagaaccgagcu........................................................................  538   1  Fm1
...................Cuacccuguagaaccgagcu........................................................................  19    1  Fm1
...................guacccuguagaaccgagcu........................................................................  388   0  Fm1
...................guacccuguagaaccgagcua.......................................................................  18    0  Fm1
...................guacccuguagaaccgaCcua.......................................................................  2     1  Fm1
...................Auacccuguagaaccgagcua.......................................................................  8     1  Fm1
...................guacccuguagaaccgagcuU.......................................................................  14    1  Fm1
...................guacccAguagaaccgagcua.......................................................................  1     1  Fm1
...................Cuacccuguagaaccgagcua.......................................................................  3     1  Fm1
...................guacccuguagaaccgagcGa.......................................................................  15    1  Fm1
...................guacccuguagaaccgagcuGg......................................................................  5     1  Fm1
...................guacccuguagaaccgagcuaA......................................................................  3     1  Fm1
...................guacccuguagaaccgagcuag......................................................................  537   0  Fm1
...................guacccuguagaaccgaUcuag......................................................................  2     1  Fm1
...................guacccuguagaaccgUgcuag......................................................................  1     1  Fm1
...................Cuacccuguagaaccgagcuag......................................................................  20    1  Fm1
```

```
                    Mature                                 Star
aaauguggugaguuucuguguacccuguagaaccgagcuaguguaucgaguucgaaacucacaagccauucuacaguguacaaagaaacgucgcuucuuucaacaaugaga
...................guacccuguagaaccgagcuaU......................................................................    24      1    Fm1
...................Auacccuguagaaccgagcuag......................................................................    14      1    Fm1
...................guacccuguagaaccgaAcuag......................................................................    2       1    Fm1
...................guacccuguagaaccgaCcuag......................................................................    4       1    Fm1
...................guacccuguagaaccgagcGag......................................................................    19      1    Fm1
...................guaAccuguagaaccgagcuag......................................................................    1       1    Fm1
...................guacccuguagaaccgCgcuag......................................................................    1       1    Fm1
...................guacccCguagaaccgagcuag......................................................................    1       1    Fm1
...................guacccGguagaaccgagcuagu.....................................................................    1       1    Fm1
...................guacccuguaCaaccgagcuagu.....................................................................    12      1    Fm1
...................guaUccuguagaaccgagcuagu.....................................................................    3       1    Fm1
...................guaAccuguagaaccgagcuagu.....................................................................    2       1    Fm1
...................guacUcuguagaaccgagcuagu.....................................................................    2       1    Fm1
...................guacccuguagaaccgagcuaAu.....................................................................    2       1    Fm1
...................guacccuguUgaaccgagcuagu.....................................................................    2       1    Fm1
...................guCccuguagaaccgagcuagu.....................................................................    1       1    Fm1
...................guacccNguagaaccgagcuagu.....................................................................    1       1    Fm1
...................guacccuguagaaccgaUcuagu.....................................................................    71      1    Fm1
...................guacccugAagaaccgagcuagu.....................................................................    1       1    Fm1
...................guacccuguagaaccUagcuagu.....................................................................    1       1    Fm1
...................guaccUuguagaaccgagcuagu.....................................................................    5       1    Fm1
...................guaccGuguagaaccgagcuagu.....................................................................    1       1    Fm1
...................Cuacccuguagaaccgagcuagu.....................................................................    517     1    Fm1
...................guaccAuguagaaccgagcuagu.....................................................................    3       1    Fm1
...................guacccuguagaacAgagcuagu.....................................................................    3       1    Fm1
...................guacccuguagaaccgagcuaUu.....................................................................    5       1    Fm1
...................guacccCguagaaccgagcuagu.....................................................................    4       1    Fm1
...................guacccuguagaaccgUgcuagu.....................................................................    38      1    Fm1
...................guacccuguagaaccgCgcuagu.....................................................................    64      1    Fm1
...................Auacccuguagaaccgagcuagu.....................................................................    439     1    Fm1
...................guacccuguGgaaccgagcuagu.....................................................................    2       1    Fm1
...................guacccuguagaacUgagcuagu.....................................................................    7       1    Fm1
...................guacccuguagaaccgagcuagC.....................................................................    2       1    Fm1
...................gGacccuguagaaccgagcuagu.....................................................................    2       1    Fm1
...................guacccuguagaaccgagUuagu.....................................................................    3       1    Fm1
...................guUccuguagaaccgagcuagu.....................................................................    3       1    Fm1
...................guacccuUuagaaccgagcuagu.....................................................................    2       1    Fm1
...................guacccuguagaaUcgagcuagu.....................................................................    1       1    Fm1
...................guGcccuguagaaccgagcuagu.....................................................................    6       1    Fm1
...................guacccuguagaGccgagcuagu.....................................................................    4       1    Fm1
...................guacccAguagaaccgagcuagu.....................................................................    2       1    Fm1
...................guacccuguagaUccgagcuagu.....................................................................    1       1    Fm1
...................guacccuguagaacGgagcuagu.....................................................................    1       1    Fm1
...................guacccugCagaaccgagcuagu.....................................................................    5       1    Fm1
...................guacccuguagaCccgagcuagu.....................................................................    2       1    Fm1
...................guacccuguagaaccgagcuGgu.....................................................................    54      1    Fm1
...................guacccuguagaaccgagcGagu.....................................................................    340     1    Fm1
...................guacccuguagaaccgagcCagu.....................................................................    2       1    Fm1
...................Nuacccuguagaaccgagcuagu.....................................................................    7       1    Fm1
...................guacccuguagaaccgagcuagu.....................................................................    19253   0    Fm1
...................guaGccuguagaaccgagcuagu.....................................................................    3       1    Fm1
...................guacccuguagUaccgagcuagu.....................................................................    3       1    Fm1
...................guacccuguaAaaccgagcuagu.....................................................................    31      1    Fm1
...................Uuacccuguagaaccgagcuagu.....................................................................    24      1    Fm1
...................guacccuguagaaccgagcuagG.....................................................................    42      1    Fm1
...................guacGcuguagaaccgagcuagu.....................................................................    1       1    Fm1
...................guaccNuguagaaccgagcuagu.....................................................................    2       1    Fm1
...................guacccuguaUaaccgagcuagu.....................................................................    14      1    Fm1
...................guacccuguagGaccgagcuagu.....................................................................    4       1    Fm1
...................guacccuguagaaccAagcuagu.....................................................................    1       1    Fm1
...................guacccuguagaaccgGgcuagu.....................................................................    8       1    Fm1
...................guacAcuguagaaccgagcuagu.....................................................................    4       1    Fm1
...................guacccuAuagaaccgagcuagu.....................................................................    1       1    Fm1
...................guacccuguagaaccgagcuagA.....................................................................    23      1    Fm1
...................gCacccuguagaaccgagcuagu.....................................................................    2       1    Fm1
...................guacccuguagaaGcgagcuagu.....................................................................    1       1    Fm1
...................guacccuguagaaccgaAcuagu.....................................................................    87      1    Fm1
...................guacccuguagaaAcgagcuagu.....................................................................    2       1    Fm1
...................gAacccuguagaaccgagcuagu.....................................................................    6       1    Fm1
...................guacccuguagaaccgaCcuagu.....................................................................    69      1    Fm1
```

```
                    Mature                                   Star
aaauguggugaguuucuguguacccuguagaaccgagcuaguguaucgaguucgaaacucacaagccauucuacaguguacaaagaaacgucgcuucuuucaacaaugaga
...................guacccuguagaaccgagAuagu.....................................................................   2      1    Fm1
...................guacccuguagaaccgagcuUgu.....................................................................   2      1    Fm1
...................guacccuguagaaccgagcuagug....................................................................   1138   0    Fm1
...................guacccuguagaaccgaCcuagug....................................................................   8      1    Fm1
...................guacccAguagaaccgagcuagug....................................................................   1      1    Fm1
...................Uuacccuguagaaccgagcuagug....................................................................   1      1    Fm1
...................guacccuguagaaccgagcuaguU....................................................................   875    1    Fm1
...................guacccuguaUaaccgagcuagug....................................................................   2      1    Fm1
...................guacccuguagaaccgagcuUgug....................................................................   1      1    Fm1
...................guacccuguagaaccgagcuGgug....................................................................   5      1    Fm1
...................guacccuguaAaaccgagcuagug....................................................................   1      1    Fm1
...................guacccuguagaaccgaAcuagug....................................................................   7      1    Fm1
...................guacccuguagaaccgagcuaguA....................................................................   17     1    Fm1
...................guacccuguagaaccgCgcuagug....................................................................   3      1    Fm1
...................guGcccuguagaaccgagcuagug....................................................................   1      1    Fm1
...................guacccuguagaaccgagcuagAg....................................................................   1      1    Fm1
...................Auacccuguagaaccgagcuagug....................................................................   10     1    Fm1
...................guacccuguagaaccgaUcuagug....................................................................   3      1    Fm1
...................guacccuguagaaccgGgcuagug....................................................................   1      1    Fm1
...................guacccuguagaaccgagcuaAug....................................................................   1      1    Fm1
...................guacccuguCgaaccgagcuagug....................................................................   1      1    Fm1
...................guacccuguagaaccgagcGagug....................................................................   6      1    Fm1
...................Cuacccuguagaaccgagcuagug....................................................................   44     1    Fm1
...................guacccCguagaaccgagcuagug....................................................................   1      1    Fm1
...................guacccuguagaaccgagcCagug....................................................................   1      1    Fm1
...................guacccuguagaaccgagcuagugA...................................................................   6      1    Fm1
...................guacccuguaAaaccgagcuagugu...................................................................   1      1    Fm1
...................guacccuguagaaccgGgcuagugu...................................................................   1      1    Fm1
...................guacccuguagaaccgCgcuagugu...................................................................   2      1    Fm1
...................guacccuguagaaccgagcuagugu...................................................................   716    0    Fm1
...................guacccuguagaaccgagcuagugG...................................................................   79     1    Fm1
...................guacccuguagaaccgUgcuagugu...................................................................   2      1    Fm1
...................Uuacccuguagaaccgagcuagugu...................................................................   2      1    Fm1
...................Cuacccuguagaaccgagcuagugu...................................................................   23     1    Fm1
...................guacccuguagaaccgagcuaguAu...................................................................   7      1    Fm1
...................guacccuguagaaccgagcuGgugu...................................................................   15     1    Fm1
...................guacccuguagaaccgagcGagugu...................................................................   1      1    Fm1
...................gAacccuguagaaccgagcuagugu...................................................................   1      1    Fm1
...................guacccuguagaaccgagcuaguCu...................................................................   3      1    Fm1
...................guacccuguagaaccgagcuaguUu...................................................................   47     1    Fm1
...................guaccAuguagaaccgagcuagugu...................................................................   1      1    Fm1
...................guacccuguagaaccgaUcuagugu...................................................................   1      1    Fm1
...................guacccAguagaaccgagcuagugu...................................................................   1      1    Fm1
...................guacccuguagaaccgaCcuagugu...................................................................   4      1    Fm1
...................guacccuguagaaccgaAcuagugu...................................................................   2      1    Fm1
...................Auacccuguagaaccgagcuagugu...................................................................   16     1    Fm1
...................guacccuguagaaccgagcuagugC...................................................................   2      1    Fm1
...................guacccuguagaaccgagcuaguguU..................................................................   51     1    Fm1
...................guacccuguagaaccgagcuaguguUu.................................................................   1      1    Fm1
...................guacccuguagaaccgagcuaguguau.................................................................   7      0    Fm1
...................guacccuguagaaccgagcuaguguauc................................................................   14     0    Fm1
...................guacccuguagaaccgaCcuaguguauc................................................................   1      1    Fm1
...................guacccuguagaaccgagcuaguguaucg...............................................................   21     0    Fm1
...................guacccuguagaaccgagcuaguguUucg...............................................................   1      1    Fm1
...................guacccuguagaaccgagcuaguguaucgag.............................................................   1      0    Fm1
...................guacccuguagaaccgagcuaguguaucgagu............................................................   12     0    Fm1
...................guacccuguagaaccgagcuaguguaAcgagu............................................................   1      1    Fm1
...................guacccuguagaaccgagcuaguguaucgaguu...........................................................   16     0    Fm1
...................guacccuguagaaccgagcuaguguaucgaguucgaaacuc...................................................   1      0    Fm1
....................Aacccuguagaaccgagc.........................................................................   1      1    Fm1
....................uacccuguagGaccgagc.........................................................................   3      1    Fm1
....................uNcccuguagaaccgagc.........................................................................   1      1    Fm1
....................uUcccuguagaaccgagc.........................................................................   1      1    Fm1
....................uacccuguaUaaccgagc.........................................................................   1      1    Fm1
....................uacccuguagaacUgagc.........................................................................   2      1    Fm1
....................uacccuguagaaccgaCc.........................................................................   5      1    Fm1
....................uacccugGagaaccgagc.........................................................................   1      1    Fm1
....................uacccuguagUaccgagc.........................................................................   3      1    Fm1
....................uacccuguagaaccgaAc.........................................................................   5      1    Fm1
....................uacccuNuagaaccgagc.........................................................................   1      1    Fm1
```

```
                    Mature                          Star
aaauguggugaguuucuguguacccuguagaaccgagcuaguguaucgaguucgaaacucacaagccauucuacaguguacaaagaaacgucgcuucuuucaacaaugaga
....................uacccuguGgaaccgagc.........................................................................   2      1   Fm1
....................uacccugCagaaccgagc.........................................................................   1      1   Fm1
....................uacccugAagaaccgagc.........................................................................   1      1   Fm1
....................uacccuguagaaccgagc.........................................................................   2644   0   Fm1
....................Nacccuguagaaccgagc.........................................................................   1      1   Fm1
....................uacccuguagaaccgaUc.........................................................................   5      1   Fm1
....................Cacccuguagaaccgagc.........................................................................   1      1   Fm1
....................uGcccuguagaaccgagc.........................................................................   4      1   Fm1
....................uacccuAuagaaccgagc.........................................................................   1      1   Fm1
....................uacccuguagaaccgagU.........................................................................   34     1   Fm1
....................uacccuguagaaccgagA.........................................................................   41     1   Fm1
....................uacccuguagaacAgagc.........................................................................   1      1   Fm1
....................uacccuguagaaccgagG.........................................................................   16     1   Fm1
....................uaccGuguagaaccgagcu........................................................................   1      1   Fm1
....................uacccuguagaaccgaAcu........................................................................   18     1   Fm1
....................uacccuguagaaccgagGu........................................................................   33     1   Fm1
....................uacccuguagaUccgagcu........................................................................   2      1   Fm1
....................uacccuguagaaccAagcu........................................................................   1      1   Fm1
....................uacccuguGgaaccgagcu........................................................................   5      1   Fm1
....................uacccCguagaaccgagcu........................................................................   2      1   Fm1
....................uCcccuguagaaccgagcu........................................................................   1      1   Fm1
....................uacccuguagaacAgagcu........................................................................   2      1   Fm1
....................uacccuCuagaaccgagcu........................................................................   1      1   Fm1
....................uacccuguUgaaccgagcu........................................................................   2      1   Fm1
....................uacccuguagaaccgagcC........................................................................   2      1   Fm1
....................uacccuguagaaccgaUcu........................................................................   23     1   Fm1
....................uGcccuguagaaccgagcu........................................................................   3      1   Fm1
....................uacccuguagaaccgCgcu........................................................................   3      1   Fm1
....................uacUcuguagaaccgagcu........................................................................   3      1   Fm1
....................uacccuguaUaaccgagcu........................................................................   1      1   Fm1
....................uacccuguagaaccCagcu........................................................................   1      1   Fm1
....................uacccuguagaacUgagcu........................................................................   7      1   Fm1
....................uacccuguagaaccgGgcu........................................................................   1      1   Fm1
....................uUcccuguagaaccgagcu........................................................................   2      1   Fm1
....................uacccuguagaaccgagcu........................................................................   11611  0   Fm1
....................uacccuguagaaccgUgcu........................................................................   1      1   Fm1
....................uacccuguagaaAcgagcu........................................................................   1      1   Fm1
....................uacccuguagaaccgagUu........................................................................   47     1   Fm1
....................uaccUuguagaaccgagcu........................................................................   1      1   Fm1
....................uacccuguagGaccgagcu........................................................................   16     1   Fm1
....................uacccuguagaaccgagcA........................................................................   42     1   Fm1
....................uacccuguagaaccgaCcu........................................................................   38     1   Fm1
....................uacccuguagUaccgagcu........................................................................   10     1   Fm1
....................uacccuguagaGccgagcu........................................................................   1      1   Fm1
....................uacccuAuagaaccgagcu........................................................................   3      1   Fm1
....................Cacccuguagaaccgagcu........................................................................   4      1   Fm1
....................uacccGguagaaccgagcu........................................................................   1      1   Fm1
....................uaGccuguagaaccgagcu........................................................................   2      1   Fm1
....................uaccAuguagaaccgagcu........................................................................   1      1   Fm1
....................uacccuNuagaaccgagcu........................................................................   2      1   Fm1
....................uacccuguagCaccgagcu........................................................................   10     1   Fm1
....................Nacccuguagaaccgagcu........................................................................   5      1   Fm1
....................uacccuguagaaUcgagcu........................................................................   5      1   Fm1
....................uacccuUuagaaccgagcu........................................................................   1      1   Fm1
....................uaAccuguagaaccgagcu........................................................................   2      1   Fm1
....................uaUccuguagaaccgagcu........................................................................   1      1   Fm1
....................uacAcuguagaaccgagcu........................................................................   7      1   Fm1
....................uacccuguagaaccgagAu........................................................................   109    1   Fm1
....................uacccuguagaaUcgagcua.......................................................................   4      1   Fm1
....................uacccuguagaaccgagGua.......................................................................   4      1   Fm1
....................uacccuguagUaccgagcua.......................................................................   1      1   Fm1
....................uacccuguagaaccgagUua.......................................................................   5      1   Fm1
....................uacccuguagaaccgagcua.......................................................................   2091   0   Fm1
....................uacccuguagaaccgaCcua.......................................................................   1      1   Fm1
....................uacccuguagaaccgagcuU.......................................................................   662    1   Fm1
....................uacccuguagaaccgagAua.......................................................................   26     1   Fm1
....................uacccuguagaaccgagcuC.......................................................................   5      1   Fm1
....................uaUccuguagaaccgagcua.......................................................................   1      1   Fm1
....................uacccuguagaaccgaAcua.......................................................................   5      1   Fm1
....................uacccuAuagaaccgagcua.......................................................................   2      1   Fm1
```

```
                    Mature                                      Star
aaauguggugaguuucugugguacccuguagaaccgagcuaguguaucgaguucgaaacucacaagccauucuacaguguacaaagaaacgucgcuucuuucaacaaugaga
....................uacccuguagaaccgGgcua.......................................................................   1       1   Fm1
....................uacccuguagaaccgagcuG.......................................................................   16      1   Fm1
....................uacccuguagCaccgagcua.......................................................................   2       1   Fm1
....................uaccAuguagaaccgagcua.......................................................................   1       1   Fm1
....................uacccuguaUaaccgagcua.......................................................................   1       1   Fm1
....................uaGccuguagaaccgagcua.......................................................................   1       1   Fm1
....................uacccuguagaaccgaUcua.......................................................................   3       1   Fm1
....................uacccuguGgaaccgagcua.......................................................................   1       1   Fm1
....................uacccCguagaaccgagcua.......................................................................   1       1   Fm1
....................uacccuguagaaccgagcAa.......................................................................   11      1   Fm1
....................uacccGguagaaccgagcuag......................................................................   3       1   Fm1
....................uacccuguagaacGgagcuag......................................................................   3       1   Fm1
....................uacccuguagaaUcgagcuag......................................................................   8       1   Fm1
....................uaccGuguagaaccgagcuag......................................................................   1       1   Fm1
....................uacccuguagaaccgagAuag......................................................................   380     1   Fm1
....................uacccNguagaaccgagcuag......................................................................   5       1   Fm1
....................uacccuguagaaccgagcuGg......................................................................   113     1   Fm1
....................uacccuguagUaccgagcuag......................................................................   32      1   Fm1
....................Gacccuguagaaccgagcuag......................................................................   1       1   Fm1
....................uaGccuguagaaccgagcuag......................................................................   8       1   Fm1
....................uaccAuguagaaccgagcuag......................................................................   11      1   Fm1
....................uacccugCagaaccgagcuag......................................................................   3       1   Fm1
....................uacccuguagaacUgagcuag......................................................................   36      1   Fm1
....................Aacccuguagaaccgagcuag......................................................................   4       1   Fm1
....................uacccuguCgaaccgagcuag......................................................................   1       1   Fm1
....................uacccuguagaaccgagcuaC......................................................................   8       1   Fm1
....................uacccuguagaaccAagcuag......................................................................   7       1   Fm1
....................uacccuguagaaccgagcAag......................................................................   2       1   Fm1
....................uacccuguaUaaccgagcuag......................................................................   6       1   Fm1
....................uacccuguagaGccgagcuag......................................................................   3       1   Fm1
....................uaAccuguagaaccgagcuag......................................................................   11      1   Fm1
....................uacccuguagaaccgGgcuag......................................................................   9       1   Fm1
....................uGcccuguagaaccgagcuag......................................................................   6       1   Fm1
....................uacccuguGgaaccgagcuag......................................................................   3       1   Fm1
....................uacccuguagaaAcgagcuag......................................................................   3       1   Fm1
....................uacccugAagaaccgagcuag......................................................................   2       1   Fm1
....................Nacccuguagaaccgagcuag......................................................................   18      1   Fm1
....................uacccuguagaaccgaCcuag......................................................................   84      1   Fm1
....................uacccuCuagaaccgagcuag......................................................................   4       1   Fm1
....................uacccuguagaaccgagcuUg......................................................................   23      1   Fm1
....................uacUcuguagaaccgagcuag......................................................................   8       1   Fm1
....................uacccuguagaaccgagcuaU......................................................................   1307    1   Fm1
....................uCcccuguagaaccgagcuag......................................................................   1       1   Fm1
....................uaccUuguagaaccgagcuag......................................................................   11      1   Fm1
....................uacccuUuagaaccgagcuag......................................................................   3       1   Fm1
....................uacccuguUgaaccgagcuag......................................................................   4       1   Fm1
....................uacccuguagaaccgUgcuag......................................................................   8       1   Fm1
....................uacccuguagaaccgaUcuag......................................................................   67      1   Fm1
....................uacccAguagaaccgagcuag......................................................................   1       1   Fm1
....................uUcccuguagaaccgagcuag......................................................................   11      1   Fm1
....................uacccuguaAaaccgagcuag......................................................................   3       1   Fm1
....................uacccuguagaaccgaAcuag......................................................................   90      1   Fm1
....................uacccuguagaaccgagcuag......................................................................   42010   0   Fm1
....................uacccuguagCaccgagcuag......................................................................   36      1   Fm1
....................uacGcuguagaaccgagcuag......................................................................   1       1   Fm1
....................uacccuguagaaccgCgcuag......................................................................   1       1   Fm1
....................uacccuguagaaccgagcuCg......................................................................   5       1   Fm1
....................Cacccuguagaaccgagcuag......................................................................   13      1   Fm1
....................uacccCguagaaccgagcuag......................................................................   4       1   Fm1
....................uacccuguagaacAgagcuag......................................................................   8       1   Fm1
....................uacccuguagaUccgagcuag......................................................................   11      1   Fm1
....................uacccuguagGaccgagcuag......................................................................   43      1   Fm1
....................uacccuguagaaccgagcuaA......................................................................   93      1   Fm1
....................uacccuAuagaaccgagcuag......................................................................   4       1   Fm1
....................uacccuguagaaccgagcCag......................................................................   3       1   Fm1
....................uacccuguagaaccgagGuag......................................................................   126     1   Fm1
....................uaUccuguagaaccgagcuag......................................................................   7       1   Fm1
....................uacAcuguagaaccgagcuag......................................................................   15      1   Fm1
....................uacccuNuagaaccgagcuag......................................................................   3       1   Fm1
....................uacccuguagaaccgagUuag......................................................................   194     1   Fm1
```

Mature Star

```
aaauguggugaguuucugugUACCCUGUAGAACCGAGCUAGUguaucgaguucgaaacucaCAAGCCAUUCUACAGUGUACAaagaaacgucgcuucuuucaacaaugaga
```

```
aaauguggugaguuucuguguacccuguagaaccgagcuaguguaucgaguucgaaacucacaagccauucuacaguguacaaagaaacgucgcuucuuucaacaaugaga
....................uacccuguagaaccUagcuag......................................................................    6        1    Fm1
....................uacccuguagaaccgagcuagG.....................................................................    3390     1    Fm1
....................uacccuguagaaccgagcuGgu.....................................................................    3491     1    Fm1
....................uacccuguagaaccgaUcuagu.....................................................................    3232     1    Fm1
....................uacccAguagaaccgagcuagu.....................................................................    87       1    Fm1
....................uaccAuguagaaccgagcuagu.....................................................................    208      1    Fm1
....................uacccuguagaaccgagcuagu.....................................................................    1693307  0    Fm1
....................uacccuguagaaccUagcuagu.....................................................................    166      1    Fm1
....................Gacccuguagaaccgagcuagu.....................................................................    36       1    Fm1
....................uacccCguagaaccgagcuagu.....................................................................    220      1    Fm1
....................uacccuguagaGccgagcuagu.....................................................................    227      1    Fm1
....................uacccuguagaacAgagcuagu.....................................................................    375      1    Fm1
....................uacccugCagaaccgagcuagu.....................................................................    195      1    Fm1
....................uaAccuguagaaccgagcuagu.....................................................................    334      1    Fm1
....................uGcccuguagaaccgagcuagu.....................................................................    383      1    Fm1
....................uacccuguagaaccgagcuagA.....................................................................    1677     1    Fm1
....................uacccuguagaaccAagcuagu.....................................................................    302      1    Fm1
....................uacccuguagaaccgagcuaCu.....................................................................    41       1    Fm1
....................uUcccuguagaaccgagcuagu.....................................................................    385      1    Fm1
....................Aacccuguagaaccgagcuagu.....................................................................    178      1    Fm1
....................uacccuguagaUccgagcuagu.....................................................................    445      1    Fm1
....................uacccuguaCaaccgagcuagu.....................................................................    58       1    Fm1
....................uCcccuguagaaccgagcuagu.....................................................................    110      1    Fm1
....................uacccuguagaaccgagcuaAu.....................................................................    117      1    Fm1
....................uacccNguagaaccgagcuagu.....................................................................    263      1    Fm1
....................uaccGuguagaaccgagcuagu.....................................................................    81       1    Fm1
....................uacccuguagaacUgagcuagu.....................................................................    559      1    Fm1
....................uacUcuguagaaccgagcuagu.....................................................................    319      1    Fm1
....................uacccuCuagaaccgagcuagu.....................................................................    74       1    Fm1
....................uacccuguGgaaccgagcuagu.....................................................................    250      1    Fm1
....................uacccuguagaaccgagUuagu.....................................................................    8503     1    Fm1
....................uacccuguagaacGgagcuagu.....................................................................    106      1    Fm1
....................uacccuguaUaaccgagcuagu.....................................................................    183      1    Fm1
....................uacccuguCgaaccgagcuagu.....................................................................    44       1    Fm1
....................uacccuguagaaccgaNcuagu.....................................................................    1        1    Fm1
....................uacccuguagaaccgagcuCgu.....................................................................    118      1    Fm1
....................uacccuguUgaaccgagcuagu.....................................................................    224      1    Fm1
....................uacccuguagaaccgagcCagu.....................................................................    190      1    Fm1
....................uacccuguagaaccgagGuagu.....................................................................    5752     1    Fm1
....................uacAcuguagaaccgagcuagu.....................................................................    630      1    Fm1
....................uacccGguagaaccgagcuagu.....................................................................    81       1    Fm1
....................uaccUuguagaaccgagcuagu.....................................................................    241      1    Fm1
....................uacccuUuagaaccgagcuagu.....................................................................    173      1    Fm1
....................uacccuguagaaccCagcuagu.....................................................................    57       1    Fm1
....................uNcccuguagaaccgagcuagu.....................................................................    19       1    Fm1
....................uacccuguagaaccgagcuagC.....................................................................    410      1    Fm1
....................uacccugAagaaccgagcuagu.....................................................................    114      1    Fm1
....................uacccuguagaaccgCgcuagu.....................................................................    90       1    Fm1
....................uacccuguagaaccgagNuagu.....................................................................    1        1    Fm1
....................uacGcuguagaaccgagcuagu.....................................................................    143      1    Fm1
....................uaUccuguagaaccgagcuagu.....................................................................    264      1    Fm1
....................uacccuguagaaccgagcAagu.....................................................................    58       1    Fm1
....................uacccugGagaaccgagcuagu.....................................................................    27       1    Fm1
....................uacccuguagaaccgagAuagu.....................................................................    15923    1    Fm1
....................uacccuguagaaccgaCcuagu.....................................................................    3738     1    Fm1
....................uacccuguagaCccgagcuagu.....................................................................    27       1    Fm1
....................uacccuNuagaaccgagcuagu.....................................................................    108      1    Fm1
....................uacccuguagGaccgagcuagu.....................................................................    1890     1    Fm1
....................uacccuguagUaccgagcuagu.....................................................................    1659     1    Fm1
....................Cacccuguagaaccgagcuagu.....................................................................    329      1    Fm1
....................uacccuguagaaccgagcuUgu.....................................................................    1119     1    Fm1
....................uacccuguaAaaccgagcuagu.....................................................................    102      1    Fm1
....................uacccuguagaaGcgagcuagu.....................................................................    47       1    Fm1
....................Nacccuguagaaccgagcuagu.....................................................................    824      1    Fm1
....................uacccuguagaaccgUgcuagu.....................................................................    403      1    Fm1
....................uacccuguagaaUcgagcuagu.....................................................................    331      1    Fm1
....................uaNccuguagaaccgagcuagu.....................................................................    9        1    Fm1
....................uacccuguagaaccgGgcuagu.....................................................................    207      1    Fm1
....................uacccuAuagaaccgagcuagu.....................................................................    167      1    Fm1
....................uacccuguagCaccgagcuagu.....................................................................    1586     1    Fm1
```

```
                    Mature                 Star
aaauguggugaguuucugugugacccuguagaaccgagcuaguguaucgaguucgaaacucacaagccauucuacaguguacaaagaaacgucgcuucuuucaacaaugaga
....................uacccuguagaaAcgagcuagu.....................................................................  58     1  Fm1
....................uacccuguagaaccgaAcuagu.....................................................................  3456   1  Fm1
....................uaGccuguagaaccgagcuagu.....................................................................  335    1  Fm1
....................uacccuguagaaccgagcuaUu.....................................................................  301    1  Fm1
....................uacccuguagaaccgagcCagug....................................................................  8      1  Fm1
....................uacccuguagaUccgagcuagug....................................................................  18     1  Fm1
....................uaccUuguagaaccgagcuagug....................................................................  10     1  Fm1
....................uacccuguagaaccgagcuCgug....................................................................  2      1  Fm1
....................uacccuNuagaaccgagcuagug....................................................................  6      1  Fm1
....................uacUcuguagaaccgagcuagug....................................................................  18     1  Fm1
....................uNcccuguagaaccgagcuagug....................................................................  1      1  Fm1
....................uacccuguagaaccgagcuaUug....................................................................  8      1  Fm1
....................uacccuguagaaccgagcuaAug....................................................................  6      1  Fm1
....................uacccuguagaaccgUgcuagug....................................................................  15     1  Fm1
....................uacccuguagaGccgagcuagug....................................................................  18     1  Fm1
....................uacccuAuagaaccgagcuagug....................................................................  7      1  Fm1
....................uCcccuguagaaccgagcuagug....................................................................  5      1  Fm1
....................uacccugAagaaccgagcuagug....................................................................  3      1  Fm1
....................uacccuCuagaaccgagcuagug....................................................................  2      1  Fm1
....................uacccuguagaaccgagUuagug....................................................................  378    1  Fm1
....................uaAccuguagaaccgagcuagug....................................................................  17     1  Fm1
....................uaccGuguagaaccgagcuagug....................................................................  5      1  Fm1
....................uacccugGagaaccgagcuagug....................................................................  2      1  Fm1
....................uacccCguagaaccgagcuagug....................................................................  5      1  Fm1
....................GacccuguagaaccgagcuaguG....................................................................  1      1  Fm1
....................uacccuguagaaccgagAuagug....................................................................  746    1  Fm1
....................uacccuguagaaccgagcuagGg....................................................................  23     1  Fm1
....................uacccuguagaaccgagcuagCg....................................................................  10     1  Fm1
....................uacccuguagGaccgagcuagug....................................................................  78     1  Fm1
....................uacccuguagaaccgagGuagug....................................................................  247    1  Fm1
....................uacccuguagaaAcgagcuagug....................................................................  1      1  Fm1
....................uacccuguGgaaccgagcuagug....................................................................  13     1  Fm1
....................uacccuguagaaccUagcuagug....................................................................  11     1  Fm1
....................Cacccuguagaaccgagcuagug....................................................................  17     1  Fm1
....................uaGccuguagaaccgagcuagug....................................................................  19     1  Fm1
....................uacccuguagaaccgagcuaguU....................................................................  22486  1  Fm1
....................uacccuguaUaaccgagcuagug....................................................................  8      1  Fm1
....................uacGcuguagaaccgagcuagug....................................................................  8      1  Fm1
....................uacccuUuagaaccgagcuagug....................................................................  11     1  Fm1
....................uacccuguagaacGgagcuagug....................................................................  6      1  Fm1
....................uUcccuguagaaccgagcuagug....................................................................  22     1  Fm1
....................uacccuguagaaccgagcuaguA....................................................................  2699   1  Fm1
....................uacccuguagaacUgagcuagug....................................................................  30     1  Fm1
....................uaUccuguagaaccgagcuagug....................................................................  9      1  Fm1
....................uGcccuguagaaccgagcuagug....................................................................  17     1  Fm1
....................uacccuguagaaccgagcuUgug....................................................................  29     1  Fm1
....................uacAcuguagaaccgagcuagug....................................................................  21     1  Fm1
....................uacccuguaAaccgagcuagug.....................................................................  9      1  Fm1
....................uacccuguagaaccgagcuagAg....................................................................  10     1  Fm1
....................uacccuguagaaUcgagcuagug....................................................................  11     1  Fm1
....................uacccuguagCaccgagcuagug....................................................................  70     1  Fm1
....................uacccuguCgaaccgagcuagug....................................................................  2      1  Fm1
....................Aacccuguagaaccgagcuagug....................................................................  10     1  Fm1
....................uacccuguaCaaccgagcuagug....................................................................  7      1  Fm1
....................uacccuguagaaccAagcuagug....................................................................  9      1  Fm1
....................uacccGguagaaccgagcuagug....................................................................  7      1  Fm1
....................uacccNguagaaccgagcuagug....................................................................  18     1  Fm1
....................uacccuguagaaccgagcuaCug....................................................................  2      1  Fm1
....................uacccuguagaaccgaCcuagug....................................................................  175    1  Fm1
....................uacccuguagaaccgagcuagug....................................................................  77166  0  Fm1
....................uacccuguagaaccgagcuGgug....................................................................  232    1  Fm1
....................uacccuguagaaccgCgcuagug....................................................................  3      1  Fm1
....................uacccuguagUaccgagcuagug....................................................................  82     1  Fm1
....................uacccuguagaacAgagcuagug....................................................................  19     1  Fm1
....................uacccuguagaaccgaUcuagug....................................................................  144    1  Fm1
....................uacccuguagaaccgagcuaguC....................................................................  49     1  Fm1
....................uacccuguagaaccgagcAagug....................................................................  3      1  Fm1
....................uacccuguagaaccgaNcuagug....................................................................  1      1  Fm1
....................uacccugCagaaccgagcuagug....................................................................  7      1  Fm1
....................uacccAguagaaccgagcuagug....................................................................  4      1  Fm1
```

```
                    Mature                               Star
aaauguggugaguuucugugguacccuguagaaccgagcuaguguaucgaguucgaaacucacaagccauucuacaguguacaaagaaacgucgcuucuuucaacaaugaga
```

Note: in the printed reference line, "uacccuguagaaccgagcuagu" is the Mature region, "guaucgaguucgaaacuca" is the loop, and "caagccauucuacaguguaca" is the Star region; the preceding "aaauguggugaguuucugug" and following "aagaaacgucgcuucuuucaacaaugaga" are flanking sequence.

```
....................uacccuguagaaccgaAcuagug....................................................................  188    1    Fm1
....................uacccuguagaaGcgagcuagug....................................................................  2      1    Fm1
....................uaccAuguagaaccgagcuagug....................................................................  6      1    Fm1
....................uacccuguUgaaccgagcuagug....................................................................  9      1    Fm1
....................Nacccuguagaaccgagcuagug....................................................................  49     1    Fm1
....................uacccuguagaaccgGgcuagug....................................................................  13     1    Fm1
....................uacccuguagUaccgagcuagugu...................................................................  99     1    Fm1
....................uacccuCuagaaccgagcuagugu...................................................................  2      1    Fm1
....................uacccuguagaaccgagcuaAugu...................................................................  7      1    Fm1
....................uacccuguagaaccgagcuaUugu...................................................................  3      1    Fm1
....................uacccuguagaaccgaAcuagugu...................................................................  185    1    Fm1
....................uacccCguagaaccgagcuagugu...................................................................  12     1    Fm1
....................uacccuguagaaccAagcuagugu...................................................................  20     1    Fm1
....................uacccuguagaaccgaUcuagugu...................................................................  190    1    Fm1
....................uacccuguagaaAcgagcuagugu...................................................................  5      1    Fm1
....................uacccuguagaaccgagcuagCgu...................................................................  13     1    Fm1
....................uacccuguagaaUcgagcuagugu...................................................................  13     1    Fm1
....................uacccuguagaaccgagcuCgugu...................................................................  4      1    Fm1
....................uacccugCagaaccgagcuagugu...................................................................  7      1    Fm1
....................uacccuguagaaccgaCcuagugu...................................................................  249    1    Fm1
....................uacccuguagaaccgagcuagAgu...................................................................  13     1    Fm1
....................uacccuAuagaaccgagcuagugu...................................................................  11     1    Fm1
....................uacccuguagaacGgagcuagugu...................................................................  5      1    Fm1
....................uacccuguagaaccgagcuagugC...................................................................  203    1    Fm1
....................Cacccuguagaaccgagcuagugu...................................................................  17     1    Fm1
....................uacccuguaAaaccgagcuagugu...................................................................  5      1    Fm1
....................uacccuguagaaccgagcuagGgu...................................................................  5      1    Fm1
....................uaccGuguagaaccgagcuagugu...................................................................  3      1    Fm1
....................uacccuguagaaccUagcuagugu...................................................................  10     1    Fm1
....................uacccuguagaaccgCgcuagugu...................................................................  4      1    Fm1
....................uacccuguagaaccgagcCagugu...................................................................  6      1    Fm1
....................uacccuguagaaccgagcuagugA...................................................................  1644   1    Fm1
....................uacccuguagaCccgagcuagugu...................................................................  1      1    Fm1
....................uacccuguagaacUgagcuagugu...................................................................  30     1    Fm1
....................uacccuguagaaccgagcuaguAu...................................................................  1534   1    Fm1
....................uacccAguagaaccgagcuagugu...................................................................  9      1    Fm1
....................uacccuguagaaccgagAuagugu...................................................................  884    1    Fm1
....................uacccuguCgaaccgagcuagugu...................................................................  2      1    Fm1
....................uacccuguagaaccgGgcuagugu...................................................................  6      1    Fm1
....................uacccuguaUaaccgagcuagugu...................................................................  7      1    Fm1
....................uacUcuguagaaccgagcuagugu...................................................................  12     1    Fm1
....................Nacccuguagaaccgagcuagugu...................................................................  43     1    Fm1
....................uGcccuguagaaccgagcuagugu...................................................................  25     1    Fm1
....................uacccuguagaacAgagcuagugu...................................................................  13     1    Fm1
....................uUcccuguagaaccgagcuagugu...................................................................  28     1    Fm1
....................uacccuguagaaccgagcuagugG...................................................................  12430  1    Fm1
....................uacccNguagaaccgagcuagugu...................................................................  12     1    Fm1
....................uacccuguagaaccgagcuaguUu...................................................................  2977   1    Fm1
....................uaAccuguagaaccgagcuagugu...................................................................  19     1    Fm1
....................uacccuguagaaccgagcAagugu...................................................................  3      1    Fm1
....................uacccuguaCaaccgagcuagugu...................................................................  5      1    Fm1
....................uaccAuguagaaccgagcuagugu...................................................................  10     1    Fm1
....................Aacccuguagaaccgagcuagugu...................................................................  12     1    Fm1
....................uacccuguagaGccgagcuagugu...................................................................  10     1    Fm1
....................uacccuguagaaccgagcuagugu...................................................................  97609  0    Fm1
....................uaNccuguagaaccgagcuagugu...................................................................  1      1    Fm1
....................uacccuNuagaaccgagcuagugu...................................................................  2      1    Fm1
....................uacccuguagaaGcgagcuagugu...................................................................  1      1    Fm1
....................uacccGguagaaccgagcuagugu...................................................................  3      1    Fm1
....................uacccuguagaaccgagcuUgugu...................................................................  28     1    Fm1
....................uaGccuguagaaccgagcuagugu...................................................................  18     1    Fm1
....................uacccugGagaaccgagcuagugu...................................................................  6      1    Fm1
....................uacccuguagaaccgUgcuagugu...................................................................  10     1    Fm1
....................uacccuguagaaccgagcuaguCu...................................................................  21     1    Fm1
....................uacccuguagaaccgagcuaCugu...................................................................  3      1    Fm1
....................uacGcuguagaaccgagcuagugu...................................................................  2      1    Fm1
....................uacccuguagGaccgagcuagugu...................................................................  102    1    Fm1
....................uacccuguagCaccgagcuagugu...................................................................  76     1    Fm1
....................uCcccuguagaaccgagcuagugu...................................................................  6      1    Fm1
....................uacccugAagaaccgagcuagugu...................................................................  5      1    Fm1
```

```
                    Mature                                    Star
aaauguggugaguuucuguguacccuguagaaccgagcuaguguaucgaguucgaaacucacaagccauucuacaguguacaaagaaacgucgcuucuuucaacaaugaga
....................uacccuguagaaccgagGuagugu...................................................................    339    1    Fm1
....................uacccuguagaaccgagUuagugu...................................................................    472    1    Fm1
....................uacccuguGgaaccgagcuagugu...................................................................    16     1    Fm1
....................uaccUuguagaaccgagcuagugu...................................................................    11     1    Fm1
....................uacccuguUgaaccgagcuagugu...................................................................    21     1    Fm1
....................uacccuguagaUccgagcuagugu...................................................................    15     1    Fm1
....................Gacccuguagaaccgagcuagugu...................................................................    1      1    Fm1
....................uacAcuguagaaccgagcuagugu...................................................................    34     1    Fm1
....................uacccuguagaaccgagcGagugu...................................................................    41     1    Fm1
....................uacccuguagaaccgagcuGgugu...................................................................    292    1    Fm1
....................uacccuUuagaaccgagcuagugu...................................................................    11     1    Fm1
....................uaUccuguagaaccgagcuagugu...................................................................    13     1    Fm1
....................uacccuguagaaccgagcuagugua..................................................................    52     0    Fm1
....................uacccuguagaaccgagcuaguguG..................................................................    12     1    Fm1
....................uacccuguagaaccgagcuagugCa..................................................................    1      1    Fm1
....................uacccuguagaaccgagcuagugAa..................................................................    19     1    Fm1
....................uacccuguagaaccgagcuaguguC..................................................................    12     1    Fm1
....................uacccuguagaaccgagcuaguguU..................................................................    4285   1    Fm1
....................uacccuguagaaccgagAuagugua..................................................................    1      1    Fm1
....................uacccuguagaaccgaAcuagugua..................................................................    1      1    Fm1
....................uacccuguagaaccgagcuaguguUu.................................................................    141    1    Fm1
....................uacccuguagaaccgagcuagugAau.................................................................    11     1    Fm1
....................uacccuguagaaccgagAuaguguau.................................................................    4      1    Fm1
....................uacccuguagaaccgagcuaguguCu.................................................................    3      1    Fm1
....................uacccuguagaaccgagcuaguguau.................................................................    30     0    Fm1
....................uacccuguagaaccgagcuaguguauA................................................................    2      1    Fm1
....................uacccuguagaaccgaCcuaguguauc................................................................    1      1    Fm1
....................uacccuguagaaccgagcuaguguauc................................................................    46     0    Fm1
....................uacccuguagaaccgaUcuaguguauc................................................................    1      1    Fm1
....................uacccuguagaaccgagAuaguguauc................................................................    1      1    Fm1
....................uacccuguagaaccgagcuaguguauU................................................................    3      1    Fm1
....................uacccuguagaaccgagcuaguguaucU...............................................................    14     1    Fm1
....................uacccuguagaaccgagcuaguguaucg...............................................................    42     0    Fm1
....................uacccuguagaaccgagAuaguguaucg...............................................................    1      1    Fm1
....................uacccuguagaaccgagcuaguguaucgagu............................................................    7      0    Fm1
....................uacccuguagaaccgagcuaguguaucgaguu...........................................................    11     0    Fm1
.....................acccuguagaaccgagcu........................................................................    78     0    Fm1
.....................acccuguagaaccgCgcu........................................................................    1      1    Fm1
.....................acccuguagaaccgagcuU.......................................................................    1      1    Fm1
.....................acccuguagaaccgagcua.......................................................................    4      0    Fm1
.....................acccuguagaaccgagcAag......................................................................    1      1    Fm1
.....................acccuguagaUccgagcuag......................................................................    1      1    Fm1
.....................acccuguagCaccgagcuag......................................................................    1      1    Fm1
.....................acccuguagaaccgagcuaU......................................................................    9      1    Fm1
.....................acccuguagaaccgagcuaC......................................................................    1      1    Fm1
.....................acccuguagaaUcgagcuag......................................................................    2      1    Fm1
.....................acccuguagaaccgagcCag......................................................................    2      1    Fm1
.....................acccuguagaCccgagcuag......................................................................    1      1    Fm1
.....................acccuguagaaccgagcuag......................................................................    136    0    Fm1
.....................acccuguagaaccgagcuUg......................................................................    1      1    Fm1
.....................acccuguagaaccgagAuag......................................................................    1      1    Fm1
.....................acccuguagaaccgaCcuag......................................................................    1      1    Fm1
.....................aGccuguagaaccgagcuagu.....................................................................    2      1    Fm1
.....................acccGguagaaccgagcuagu.....................................................................    2      1    Fm1
.....................acccuguagUaccgagcuagu.....................................................................    1      1    Fm1
.....................acccuAuagaaccgagcuagu.....................................................................    5      1    Fm1
.....................acccuguagaaccgagcuCgu.....................................................................    13     1    Fm1
.....................acccuguagaaccgaAcuagu.....................................................................    7      1    Fm1
.....................acccuguagaaccgagcuGgu.....................................................................    21     1    Fm1
.....................acccuguagaaccgagcuagu.....................................................................    7463   0    Fm1
.....................acccuguagaaccAagcuagu.....................................................................    6      1    Fm1
.....................acccuguagaaUcgagcuagu.....................................................................    2      1    Fm1
.....................acccuguagaUccgagcuagu.....................................................................    17     1    Fm1
.....................acccuguagaacGgagcuagu.....................................................................    2      1    Fm1
.....................acccuguagaaccgCgcuagu.....................................................................    16     1    Fm1
.....................acccCguagaaccgagcuagu.....................................................................    1      1    Fm1
.....................acccuguagaaccgGgcuagu.....................................................................    9      1    Fm1
.....................acccuguagCaccgagcuagu.....................................................................    7      1    Fm1
.....................acccuguagaaccgagcuaUu.....................................................................    7      1    Fm1
.....................acccuguagaaccUagcuagu.....................................................................    5      1    Fm1
```

```
                    Mature                                      Star
aaauguggugaguuucugugtacccuguagaaccgagcuaguguaucgaguucgaaacucacaagccauucuacaguguacaaagaaacgucgcuucuuucaacaaugaga
.....................accGuguagaaccgagcuagu.....................................................................  1    1  Fm1
.....................Ccccuguagaaccgagcuagu.....................................................................  1    1  Fm1
.....................acccugCagaaccgagcuagu.....................................................................  7    1  Fm1
.....................acccuguaAaccgagcuagu......................................................................  4    1  Fm1
.....................acAcuguagaaccgagcuagu.....................................................................  5    1  Fm1
.....................acccuguUgaaccgagcuagu.....................................................................  2    1  Fm1
.....................acUcuguagaaccgagcuagu.....................................................................  2    1  Fm1
.....................acGcuguagaaccgagcuagu.....................................................................  3    1  Fm1
.....................acccuguagaaccgagcuUgu.....................................................................  9    1  Fm1
.....................aUccuguagaaccgagcuagu.....................................................................  2    1  Fm1
.....................acccuguagaCccgagcuagu.....................................................................  11   1  Fm1
.....................acccuguagaaccgagAuagu.....................................................................  12   1  Fm1
.....................Gcccuguagaaccgagcuagu.....................................................................  1    1  Fm1
.....................acccuNuagaaccgagcuagu.....................................................................  2    1  Fm1
.....................acccuCuagaaccgagcuagu.....................................................................  1    1  Fm1
.....................acccuguagaaccgaUcuagu.....................................................................  4    1  Fm1
.....................acccuguagaaccgagGuagu.....................................................................  4    1  Fm1
.....................acccuguagaacAgagcuagu.....................................................................  4    1  Fm1
.....................acccugGagaaccgagcuagu.....................................................................  2    1  Fm1
.....................acccuguagaaccgagcAagu.....................................................................  17   1  Fm1
.....................acccuguagaaccgagcuaAu.....................................................................  8    1  Fm1
.....................Ncccuguagaaccgagcuagu.....................................................................  3    1  Fm1
.....................Ucccuguagaaccgagcuagu.....................................................................  12   1  Fm1
.....................acccuguaCaaccgagcuagu.....................................................................  6    1  Fm1
.....................acccugNagaaccgagcuagu.....................................................................  1    1  Fm1
.....................acccuguagaaccgagcuaCu.....................................................................  7    1  Fm1
.....................acccuguagaaccgagUuagu.....................................................................  4    1  Fm1
.....................acccuguagGaccgagcuagu.....................................................................  1    1  Fm1
.....................acccuUuagaaccgagcuagu.....................................................................  2    1  Fm1
.....................acccuguagaaccgUgcuagu.....................................................................  6    1  Fm1
.....................acccuguagaGccgagcuagu.....................................................................  18   1  Fm1
.....................acccuguagaaccgagcuagC.....................................................................  7    1  Fm1
.....................acccuguagaaccgagcuagA.....................................................................  10   1  Fm1
.....................acccugAagaaccgagcuagu.....................................................................  2    1  Fm1
.....................accAuguagaaccgagcuagu.....................................................................  5    1  Fm1
.....................acccuguagaaccgagcCagu.....................................................................  49   1  Fm1
.....................acccuguagaaAcgagcuagu.....................................................................  6    1  Fm1
.....................acccAguagaaccgagcuagu.....................................................................  1    1  Fm1
.....................aAccuguagaaccgagcuagu.....................................................................  3    1  Fm1
.....................acccuguagaaccgagcuagG.....................................................................  18   1  Fm1
.....................acccuguaUaaccgagcuagu.....................................................................  6    1  Fm1
.....................acccuguagaacUgagcuagu.....................................................................  5    1  Fm1
.....................accUuguagaaccgagcuagu.....................................................................  1    1  Fm1
.....................acccuNuagaaccgagcuagug....................................................................  1    1  Fm1
.....................acccuguagaaccgCgcuagug....................................................................  3    1  Fm1
.....................acccuguagaaccgagcCagug....................................................................  4    1  Fm1
.....................acUcuguagaaccgagcuagug....................................................................  1    1  Fm1
.....................acccuguagaaccgagcuaguA....................................................................  10   1  Fm1
.....................acccuguagaaccgagcuagug....................................................................  376  0  Fm1
.....................acccuguagaaccgagcuaguU....................................................................  83   1  Fm1
.....................acccuguagaGccgagcuagug....................................................................  1    1  Fm1
.....................acccuguagaaccgagAuagug....................................................................  2    1  Fm1
.....................acccuguagaaccgagcAagug....................................................................  2    1  Fm1
.....................acccuguagaaccgagcuagCg....................................................................  1    1  Fm1
.....................Ncccuguagaaccgagcuagug....................................................................  1    1  Fm1
.....................acccuguagaaccgagcuagugu...................................................................  654  0  Fm1
.....................acccuguagaaccgagcuUgugu...................................................................  1    1  Fm1
.....................acGcuguagaaccgagcuagugu...................................................................  1    1  Fm1
.....................acccuguagaaccgagcuagugG...................................................................  90   1  Fm1
.....................acccuguagaaccgagcuagugC...................................................................  1    1  Fm1
.....................acccuguagaaccgagcuaguAu...................................................................  10   1  Fm1
.....................acccuguagaaccgagcAagugu...................................................................  2    1  Fm1
.....................acccuguagaaccgagcuaAugu...................................................................  1    1  Fm1
.....................acccuguagaGccgagcuagugu...................................................................  1    1  Fm1
.....................acccuguagaaccgagcuaUugu...................................................................  2    1  Fm1
.....................acccuguagaaccgagcuagugA...................................................................  14   1  Fm1
.....................acccuguagaaccgagcCagugu...................................................................  3    1  Fm1
.....................acccuguagaaccgagAuagugu...................................................................  2    1  Fm1
.....................acccuguagaaccgagcuaguUu...................................................................  20   1  Fm1
.....................acccuguagaaccgagcuGgugu...................................................................  2    1  Fm1
```

Mature **Star**

```
aaauguggugaguuucugug uacccuguagaaccgagcuagu guaucgaguucgaaacuca caagccauucuacaguguaca aagaaacgucgcuucuuucaacaaugaga
.....................acccuguagaaccgagcuagAgu...................................................................	1	1	Fm1
.....................acccuguagaaUcgagcuagugu...................................................................	1	1	Fm1
.....................acUcuguagaaccgagcuagugu...................................................................	1	1	Fm1
.....................acccuguagaaccgagcGagugu...................................................................	4	1	Fm1
.....................acccuguagaaccgaUcuagugu...................................................................	1	1	Fm1
.....................acccCguagaaccgagcuagugu...................................................................	1	1	Fm1
.....................acccuguagaaccgagcuaguguG..................................................................	1	1	Fm1
.....................acccuguagaaccgagcuaguguU..................................................................	48	1	Fm1
.....................acccuguagaaccgagcuagugua..................................................................	2	0	Fm1
.....................acccuguagaaccgagcuaguguauc................................................................	5	0	Fm1
.....................acccuguagaaccgagcuaguguaucg...............................................................	17	0	Fm1
.....................acccuguagaaccgagcuaguguaucgag.............................................................	2	0	Fm1
.....................acccuguagaaccgagcuaguguaucgagu............................................................	3	0	Fm1
.....................acccuguagaaccgagcuaguguauGgagu............................................................	1	1	Fm1
.....................acccuguagaaccgagcGaguguaucgaguu...........................................................	2	1	Fm1
.....................acccuguagaaccgagcuaguguaucgaguu...........................................................	32	0	Fm1
......................cccuguagaaccgagcua.......................................................................	3	0	Fm1
......................cccuguagaaccgagcAa.......................................................................	2	1	Fm1
......................cccuguagaaccgagcuag......................................................................	6	0	Fm1
......................cccuguagaaccgagcuCgu.....................................................................	1	1	Fm1
......................cccuguagaaccgagcuUgu.....................................................................	2	1	Fm1
......................ccUuguagaaccgagcuagu.....................................................................	1	1	Fm1
......................cccuguagaaccgagcuagu.....................................................................	221	0	Fm1
......................cccuguagaaccgGgcuagu.....................................................................	4	1	Fm1
......................cccuguagaaccgagcuagG.....................................................................	2	1	Fm1
......................cccuguagaaccAagcuagu.....................................................................	1	1	Fm1
......................cccuguagaaccgagcuaUu.....................................................................	2	1	Fm1
......................cccuguagaaccgaCcuagu.....................................................................	1	1	Fm1
......................Uccuguagaaccgagcuagu.....................................................................	2	1	Fm1
......................cccuUuagaaccgagcuagu.....................................................................	1	1	Fm1
......................cccuguaAaaccgagcuagu.....................................................................	1	1	Fm1
......................cccuguagaaccgagcuGgu.....................................................................	5	1	Fm1
......................cccuguagaaccgagcuagug....................................................................	5	0	Fm1
......................cccuguagaaccgagcuCgugu...................................................................	1	1	Fm1
......................cccuguagaaccgagcuagGgu...................................................................	1	1	Fm1
......................cccuguagaaccgagcuaguUu...................................................................	1	1	Fm1
......................cccuguagaaccgagcuagugG...................................................................	1	1	Fm1
......................cccuguagaaccgagcuagugu...................................................................	12	0	Fm1
.......................ccuguagaaccgagcuagu.....................................................................	17	0	Fm1
.......................ccuguagaaccgaAcuagu.....................................................................	1	1	Fm1
.......................ccuguagaaccgagcuagug....................................................................	9	0	Fm1
.......................ccuAuagaaccgagcuagugu...................................................................	1	1	Fm1
........................cuguagaaccgagcuagu.....................................................................	2	0	Fm1
........................cuguagaaccgagcuagugu...................................................................	2	0	Fm1
.............................................................caagccauucuacagugG................................	1	1	Fm1
.............................................................caagccauucuacagugu................................	32	0	Fm1
.............................................................caagccauucuacagugCa...............................	1	1	Fm1
.............................................................caagccauucuacagugua...............................	9	0	Fm1
.............................................................caagccauucuacaguguU...............................	2	1	Fm1
.............................................................caagccauucuacagugGa...............................	1	1	Fm1
.............................................................Uaagccauucuacaguguac..............................	2	1	Fm1
.............................................................caagccauucuacaguguac..............................	576	0	Fm1
.............................................................caagAcauucuacaguguac..............................	1	1	Fm1
.............................................................caagccauucuacagugCac..............................	2	1	Fm1
.............................................................caagccauucuacaguUuac..............................	1	1	Fm1
.............................................................caagccauucuacaguAuac..............................	2	1	Fm1
.............................................................caagccauucuacagugGac..............................	5	1	Fm1
.............................................................caagccauucuacagugAac..............................	5	1	Fm1
.............................................................caagccauucAacaguguac..............................	4	1	Fm1
.............................................................caagccauucCacaguguac..............................	1	1	Fm1
.............................................................caagccauucuacaguCuac..............................	2	1	Fm1
.............................................................caagcNauucuacaguguac..............................	1	1	Fm1
.............................................................caagccauucuacaguAuaca.............................	9	1	Fm1
.............................................................caagccauucuacaguguacU.............................	14	1	Fm1
.............................................................caagccauucuaUaguguaca.............................	3	1	Fm1
.............................................................caagccauucuaGaguguaca.............................	1	1	Fm1
.............................................................caagccauucAacaguguaca.............................	4	1	Fm1
.............................................................caagccauucuacaguguacC.............................	3	1	Fm1
.............................................................caagccauucuacaguCuaca.............................	3	1	Fm1
.............................................................caagccauucuUcaguguaca.............................	1	1	Fm1
```

```
                    Mature                                  Star
aaauguggugaguuucugugguacccuguagaaccgagcuaguguaucgaguucgaaacucacaagccauucuacaguguacaaagaaacgucgcuucuuucaacaaugaga
.............................................................caagccauucuacaguguaca.............................    2140    0    Fm1
.............................................................caagccNuucuacaguguaca.............................    2       1    Fm1
.............................................................caagccCuucuacaguguaca.............................    1       1    Fm1
.............................................................caagccauucuacaguUuaca.............................    1       1    Fm1
.............................................................caagccauucuacaguguaUa.............................    1       1    Fm1
.............................................................caagccauucuacaguguacG.............................    1       1    Fm1
.............................................................caagccauucuacagugAaca.............................    19      1    Fm1
.............................................................Uaagccauucuacaguguaca.............................    9       1    Fm1
.............................................................Naagccauucuacaguguaca.............................    1       1    Fm1
.............................................................caagccauucuacagugGaca.............................    11      1    Fm1
.............................................................caagccauucuacagugCaca.............................    9       1    Fm1
.............................................................caagccauucGacaguguaca.............................    4       1    Fm1
.............................................................caagUcauucuacaguguaca.............................    1       1    Fm1
.............................................................caagccauucuacagugAacaa............................    3       1    Fm1
.............................................................caagccauucuacaguguacaC............................    17      1    Fm1
.............................................................caagccauucuacagugCacaa............................    3       1    Fm1
.............................................................caagccauucuUcaguguacaa............................    1       1    Fm1
.............................................................caagccauucuacaguguGcaa............................    1       1    Fm1
.............................................................caagccauucuacaguguacaa............................    258     0    Fm1
.............................................................caagccauucuacaguguacaU............................    8       1    Fm1
.............................................................caagccauucuacagugCacaaa...........................    1       1    Fm1
.............................................................caagccauucuacaguguacaaU...........................    2       1    Fm1
.............................................................caagccauucuacaguguacaaa...........................    14      0    Fm1
.............................................................caagccauucuacaguguacaaC...........................    17      1    Fm1
..............................................................aagccauucuacaguguaca.............................    3       0    Fm1
..............................................................aagccauucuacagugGaca.............................    1       1    Fm1

...............cuguguacccuguagaaccgag..........................................................................    1       0    Mm3
...............cuguguacccuguagaaccgagc.........................................................................    2       0    Mm3
...............cuguguacccuguagaaccgagcu........................................................................    7       0    Mm3
................uguguacccuguagaaccgagc.........................................................................    7       0    Mm3
................uguguacccuguagaaccgagA.........................................................................    1       1    Mm3
................uguguacccuguagaaccgagcu........................................................................    44      0    Mm3
................uguguacccuguagaaccgagcuU.......................................................................    1       1    Mm3
.................guguacccuguagaaccgagcu........................................................................    1       0    Mm3
.................Cuguacccuguagaaccgagcuag......................................................................    1       1    Mm3
.................Cuguacccuguagaaccgagcuagu.....................................................................    11      1    Mm3
.................Cuguacccuguagaaccgagcuagug....................................................................    1       1    Mm3
..................uguacccuguagaaccgU...........................................................................    1       1    Mm3
..................uguacccuguagaaccga...........................................................................    3       0    Mm3
..................uguacccuguagaaccgag..........................................................................    2       0    Mm3
..................uguacccuguagaaccgagc.........................................................................    57      0    Mm3
..................uguacccuguagaaccUagcu........................................................................    1       1    Mm3
..................uguacccuguGgaaccgagcu........................................................................    1       1    Mm3
..................uguacccuguagaaccgagcA........................................................................    2       1    Mm3
..................uguacccuguCgaaccgagcu........................................................................    1       1    Mm3
..................uguacccuguagaaccCagcu........................................................................    1       1    Mm3
..................uguacccuguagaaccgagcu........................................................................    350     0    Mm3
..................uguacccuguagaGccgagcu........................................................................    1       1    Mm3
..................uguacccuguagaaccgCgcu........................................................................    5       1    Mm3
..................uguacccuguagaaccgagcG........................................................................    1       1    Mm3
..................uguacccuguUgaaccgagcu........................................................................    1       1    Mm3
..................uguacccGguagaaccgagcu........................................................................    1       1    Mm3
..................uguacccuguaUaaccgagcu........................................................................    1       1    Mm3
..................uguacUcuguagaaccgagcua.......................................................................    1       1    Mm3
..................Cguacccuguagaaccgagcua.......................................................................    1       1    Mm3
..................uguacccuguagaaccgagcuG.......................................................................    1       1    Mm3
..................uguacccuguagaaccgagcua.......................................................................    252     0    Mm3
..................uguacccuguagaaccAagcua.......................................................................    1       1    Mm3
..................uCuacccuguagaaccgagcua.......................................................................    1       1    Mm3
..................uguacccuguagaaccgCgcua.......................................................................    2       1    Mm3
..................uguacccuguagaaccgGgcua.......................................................................    1       1    Mm3
..................uguacccuguagaaccgUgcua.......................................................................    2       1    Mm3
..................uguacccuguagaaccgagcuU.......................................................................    14      1    Mm3
..................uguacccuguagaaccgagcGa.......................................................................    12      1    Mm3
..................uguacccuguagaaccCagcua.......................................................................    2       1    Mm3
..................uguacccuguagaacUgagcuag......................................................................    3       1    Mm3
..................uguacccuguagaaccgagcuGg......................................................................    5       1    Mm3
..................uguacccuguagaaccgagcCag......................................................................    1       1    Mm3
..................uguacccuguagaaccUagcuag......................................................................    4       1    Mm3
```

Mature Star

```
aaauguggugaguuucuguguacccuguagaaccgagcuaguguaucgaguucgaaacucacaagccauucuacaguguacaaagaaacgucgcuucuuucaacaaugaga
..................uguacGcuguagaaccgagcuag......................................................................  2      1  Mm3
..................uguacccugAagaaccgagcuag......................................................................  1      1  Mm3
..................uAuacccuguagaaccgagcuag......................................................................  2      1  Mm3
..................uguacccuguagaaccgagcuag......................................................................  5657   0  Mm3
..................uguacccuguagaaccCagcuag......................................................................  12     1  Mm3
..................uguacccuguCgaaccgagcuag......................................................................  8      1  Mm3
..................uguaccNuguagaaccgagcuag......................................................................  1      1  Mm3
..................uCuacccuguagaaccgagcuag......................................................................  2      1  Mm3
..................uguacccuguagaaccgaCcuag......................................................................  1      1  Mm3
..................uguacccuguagaaccgUgcuag......................................................................  19     1  Mm3
..................uguacccuguagaaccgaAcuag......................................................................  1      1  Mm3
..................uguaccUuguagaaccgagcuag......................................................................  1      1  Mm3
..................uguacUcuguagaaccgagcuag......................................................................  7      1  Mm3
..................uguacccuguagaaUcgagcuag......................................................................  1      1  Mm3
..................uUuacccuguagaaccgagcuag......................................................................  3      1  Mm3
..................uguacccuguagaaccAagcuag......................................................................  17     1  Mm3
..................uguacccuguGgaaccgagcuag......................................................................  13     1  Mm3
..................uguacccuguagaaccgagcGag......................................................................  28     1  Mm3
..................uguaUccuguagaaccgagcuag......................................................................  1      1  Mm3
..................Nguacccuguagaaccgagcuag......................................................................  3      1  Mm3
..................uguacccuUuagaaccgagcuag......................................................................  1      1  Mm3
..................uguacccuguagaaccgagcuaA......................................................................  27     1  Mm3
..................uguacccugGagaaccgagcuag......................................................................  1      1  Mm3
..................uguacAcuguagaaccgagcuag......................................................................  1      1  Mm3
..................uguacccuguagaaccgagcuUg......................................................................  2      1  Mm3
..................uguacccuguagaaccgagUuag......................................................................  2      1  Mm3
..................uguacccuguagaaccgagcuaU......................................................................  278    1  Mm3
..................uguacccuguagaaccgagAuag......................................................................  1      1  Mm3
..................uguacccuguagaaccgCgcuag......................................................................  30     1  Mm3
..................uguacccuguUgaaccgagcuag......................................................................  4      1  Mm3
..................uguacccuguagaaccgGgcuag......................................................................  17     1  Mm3
..................uguGcccuguagaaccgagcuag......................................................................  1      1  Mm3
..................uguCcccuguagaaccgagcuag......................................................................  1      1  Mm3
..................uguacccuguagaGccgagcuag......................................................................  1      1  Mm3
..................uguacccuguUgaaccgagcuagu.....................................................................  25     1  Mm3
..................uguaccNuguagaaccgagcuagu.....................................................................  1      1  Mm3
..................uguacccugCagaaccgagcuagu.....................................................................  8      1  Mm3
..................Gguacccuguagaaccgagcuagu.....................................................................  1      1  Mm3
..................uguacccuguaCaaccgagcuagu.....................................................................  3      1  Mm3
..................uguacccuguagaaccgaUcuagu.....................................................................  12     1  Mm3
..................uguUcccuguagaaccgagcuagu.....................................................................  4      1  Mm3
..................uguacccuguagaaccgagGuagu.....................................................................  1      1  Mm3
..................uguaccAuguagaaccgagcuagu.....................................................................  1      1  Mm3
..................uguacccuguaUaaccgagcuagu.....................................................................  7      1  Mm3
..................uguacccuguagaaccgUgcuagu.....................................................................  137    1  Mm3
..................uguacccAguagaaccgagcuagu.....................................................................  1      1  Mm3
..................uguacccuguagaacUgagcuagu.....................................................................  6      1  Mm3
..................uguacccuUuagaaccgagcuagu.....................................................................  1      1  Mm3
..................uguacccuguagaaccgaCcuagu.....................................................................  1      1  Mm3
..................uguacccuguCgaaccgagcuagu.....................................................................  28     1  Mm3
..................uguaccUuguagaaccgagcuagu.....................................................................  9      1  Mm3
..................uguacccuguagaacAgagcuagu.....................................................................  3      1  Mm3
..................uUuacccuguagaaccgagcuagu.....................................................................  9      1  Mm3
..................uguacccuguagaaccAagcuagu.....................................................................  69     1  Mm3
..................uguacccuguagaaccUagcuagu.....................................................................  50     1  Mm3
..................uguacccuguagaaccgagcuagu.....................................................................  33295  0  Mm3
..................uguCcccuguagaaccgagcuagu.....................................................................  3      1  Mm3
..................uguacccuguagaaccgagcuagG.....................................................................  45     1  Mm3
..................uguacccuguagaaccgagcuUgu.....................................................................  9      1  Mm3
..................uguacccuguagaaccgagcuCgu.....................................................................  4      1  Mm3
..................uguacccuguagaaccgagcuagA.....................................................................  109    1  Mm3
..................uguacNcuguagaaccgagcuagu.....................................................................  4      1  Mm3
..................uCuacccuguagaaccgagcuagu.....................................................................  16     1  Mm3
..................uguacccuguagaaccgGgcuagu.....................................................................  87     1  Mm3
..................uguacUcuguagaaccgagcuagu.....................................................................  36     1  Mm3
..................uguacccuAuagaaccgagcuagu.....................................................................  3      1  Mm3
..................uguacccuguagaaccgagcuGgu.....................................................................  28     1  Mm3
..................Aguacccuguagaaccgagcuagu.....................................................................  6      1  Mm3
..................uguaccGuguagaaccgagcuagu.....................................................................  1      1  Mm3
..................uguacccuguagaUccgagcuagu.....................................................................  5      1  Mm3
```

```
                    Mature                      Star
aaauguggugaguuucuguguacccuguagaaccgagcuaguguaucgaguucgaaacucacaagccauucuacaguguacaaagaaacgucgcuucuuucaacaaugaga
.................uguacccuguagCaccgagcuagu......................................................................    1      1    Mm3
.................uguacccuguGgaaccgagcuagu......................................................................    56     1    Mm3
.................uguacccuguagaaGcgagcuagu......................................................................    4      1    Mm3
.................uguacccugGagaaccgagcuagu......................................................................    1      1    Mm3
.................uguacccuguagGaccgagcuagu......................................................................    1      1    Mm3
.................uguacccuguagaaccgagUuagu......................................................................    8      1    Mm3
.................uguacccuguagaaccCagcuagu......................................................................    74     1    Mm3
.................uguacccuguagUaccgagcuagu......................................................................    10     1    Mm3
.................uguGcccuguagaaccgagcuagu......................................................................    2      1    Mm3
.................uguacccuguagaGccgagcuagu......................................................................    4      1    Mm3
.................uguacccCguagaaccgagcuagu......................................................................    6      1    Mm3
.................uguacccuguagaaccgaAcuagu......................................................................    3      1    Mm3
.................Cguacccuguagaaccgagcuagu......................................................................    14     1    Mm3
.................uguacccuguagaaccgCgcuagu......................................................................    150    1    Mm3
.................uguacccuguagaCccgagcuagu......................................................................    1      1    Mm3
.................uguacccuguaAaaccgagcuagu......................................................................    1      1    Mm3
.................uguacccuguagaacGgagcuagu......................................................................    1      1    Mm3
.................uguacccuguagaaccgagcuagC......................................................................    14     1    Mm3
.................uguaAccuguagaaccgagcuagu......................................................................    6      1    Mm3
.................uguacccuguagaaccgagcGagu......................................................................    696    1    Mm3
.................ugAacccuguagaaccgagcuagu......................................................................    2      1    Mm3
.................uguacAcuguagaaccgagcuagu......................................................................    5      1    Mm3
.................ugCacccuguagaaccgagcuagu......................................................................    5      1    Mm3
.................Nguacccuguagaaccgagcuagu......................................................................    17     1    Mm3
.................uguacccuguagaaccgagcuaAu......................................................................    8      1    Mm3
.................ugGacccuguagaaccgagcuagu......................................................................    5      1    Mm3
.................uguacccuguagaaccgagcCagu......................................................................    7      1    Mm3
.................uguacccuguagaaccgagAuagu......................................................................    2      1    Mm3
.................uAuacccuguagaaccgagcuagu......................................................................    18     1    Mm3
.................uguaGccuguagaaccgagcuagu......................................................................    3      1    Mm3
.................uguacGcuguagaaccgagcuagu......................................................................    1      1    Mm3
.................uguacccuguagaaAcgagcuagu......................................................................    3      1    Mm3
.................uguacccuguagaaccgagcuaUu......................................................................    46     1    Mm3
.................uguacccuguagaaccgagcuaCu......................................................................    1      1    Mm3
.................uguacccuguagaaUcgagcuagu......................................................................    5      1    Mm3
.................uguacccuguagaacUgagcuagug.....................................................................    1      1    Mm3
.................uguacccuguagaaccgagcuagGg.....................................................................    1      1    Mm3
.................uguacccuguagaaccUagcuagug.....................................................................    1      1    Mm3
.................uguacccuguagaaccCagcuagug.....................................................................    4      1    Mm3
.................uAuacccuguagaaccgagcuagug.....................................................................    1      1    Mm3
.................uguacccuguagaaccgCgcuagug.....................................................................    5      1    Mm3
.................uguacccuguagaaccgagcuagug.....................................................................    844    0    Mm3
.................uguacccuguagaaccgUgcuagug.....................................................................    1      1    Mm3
.................uguacccuguagaaccgGgcuagug.....................................................................    3      1    Mm3
.................uguacccuguagaaccgagcuaguA.....................................................................    30     1    Mm3
.................uguacccuguCgaaccgagcuagug.....................................................................    2      1    Mm3
.................uguacccuguagaaccgagcuaguU.....................................................................    1493   1    Mm3
.................uguacccuguGgaaccgagcuagug.....................................................................    1      1    Mm3
.................uguacccuguagaaccgagcuaguC.....................................................................    5      1    Mm3
.................uguacccuguagaaccgagcuGgug.....................................................................    3      1    Mm3
.................uguacccuguagaaccgagcGagug.....................................................................    3      1    Mm3
.................uguacccuguagaaccgagcuaguAu....................................................................    9      1    Mm3
.................uguacccuguagaaccAagcuagugu....................................................................    1      1    Mm3
.................uguacccuguagaaccgagcuGgugu....................................................................    1      1    Mm3
.................uguacccuguagaaccCagcuagugu....................................................................    1      1    Mm3
.................uguacccuguagaacUgagcuagugu....................................................................    2      1    Mm3
.................uguacccuguagaaccgagcuagugC....................................................................    2      1    Mm3
.................uguacccuguagaaccgagcuagugG....................................................................    22     1    Mm3
.................uguacccuguagaaccgGgcuagugu....................................................................    2      1    Mm3
.................uguacccuguagaaccgUgcuagugu....................................................................    1      1    Mm3
.................uguacccuguagaaccgagcGagugu....................................................................    2      1    Mm3
.................uguacccuguagaaccgagcuagugu....................................................................    579    0    Mm3
.................uguacccuguagaaccgagcuUgugu....................................................................    1      1    Mm3
.................uAuacccuguagaaccgagcuagugu....................................................................    2      1    Mm3
.................uguacccuguagaaccgagcuagAgu....................................................................    1      1    Mm3
.................uguacccuguagaaccgCgcuagugu....................................................................    3      1    Mm3
.................uguacGcuguagaaccgagcuagugu....................................................................    1      1    Mm3
.................uguacccuguagaaccgagcuaguUu....................................................................    118    1    Mm3
.................uguacccuguagaaccUagcuagugu....................................................................    3      1    Mm3
.................uguacccuguagaaccgagcuagugA....................................................................    4      1    Mm3
```

```
                    Mature                Star
aaauguggugaguuucugugUACCCUGUAGAACCGAGCUAGUguaucgaguucgaaacucacaagccauucuacaguguacaaagaaacgucgcuucuuucaacaaugaga
```

Note: in the printed line above, the part under "Mature" (uacccuguagaaccgagcuagu) is in red, the part after it (guaucgaguucgaaacuca) in orange and the part under "Star" (caagccauucuacaguguaca) in purple; all of it is lower case on the page.

```
..................uguacccuguagaaccgagcuaguguU..................................................................    21    1    Mm3
..................uguacccuguagaaccgagcuaguguUu.................................................................    4     1    Mm3
..................uguacccuguagaaccgagcuaguguau.................................................................    2     0    Mm3
..................uguacccuguagaaccgagcuaguguauc................................................................    2     0    Mm3
..................uguacccuguagaaccgagcuaguguaucgag.............................................................    1     0    Mm3
...................guacccuguagaaccgag..........................................................................    2     0    Mm3
...................guacccuguagaaccgagU.........................................................................    1     1    Mm3
...................Cuacccuguagaaccgagc.........................................................................    1     1    Mm3
...................Nuacccuguagaaccgagc.........................................................................    1     1    Mm3
...................guacccuguagaaccgagc.........................................................................    12    0    Mm3
...................Uuacccuguagaaccgagc.........................................................................    2     1    Mm3
...................guacccuguagaaccgagcu........................................................................    129   0    Mm3
...................guacccuguagaaccgaAcu........................................................................    1     1    Mm3
...................guacccuguagaaccgaCcu........................................................................    2     1    Mm3
...................guacccuguagaaccgagUu........................................................................    1     1    Mm3
...................guacccuguaCaaccgagcu........................................................................    1     1    Mm3
...................Auacccuguagaaccgagcu........................................................................    146   1    Mm3
...................guacccuguagaaccgagcua.......................................................................    24    0    Mm3
...................guacccuguagaaccgagcuU.......................................................................    7     1    Mm3
...................Auacccuguagaaccgagcua.......................................................................    19    1    Mm3
...................guacccuguagaaccgagcGa.......................................................................    9     1    Mm3
...................guacccuguagaaccgagcCag......................................................................    1     1    Mm3
...................guacccuguagaaccgagcGag......................................................................    20    1    Mm3
...................guacccuguagaaccAagcuag......................................................................    1     1    Mm3
...................guacccuguagaaccgagcuGg......................................................................    2     1    Mm3
...................guacccuguagaaccgCgcuag......................................................................    1     1    Mm3
...................guacccuguagaaccgaCcuag......................................................................    1     1    Mm3
...................guacccuguagaaUcgagcuag......................................................................    1     1    Mm3
...................Auacccuguagaaccgagcuag......................................................................    683   1    Mm3
...................Uuacccuguagaaccgagcuag......................................................................    1     1    Mm3
...................guaUccuguagaaccgagcuag......................................................................    1     1    Mm3
...................guacccuAuagaaccgagcuag......................................................................    1     1    Mm3
...................guacccCguagaaccgagcuag......................................................................    1     1    Mm3
...................guacccuguagaaAcgagcuag......................................................................    1     1    Mm3
...................guacccuguCgaaccgagcuag......................................................................    1     1    Mm3
...................Cuacccuguagaaccgagcuag......................................................................    8     1    Mm3
...................guacccuguagaaccgUgcuag......................................................................    1     1    Mm3
...................guacccuguagaaccgagcuag......................................................................    728   0    Mm3
...................guacccuguagaaccgagcuaA......................................................................    1     1    Mm3
...................guacccuguagaaccgaUcuag......................................................................    5     1    Mm3
...................guacccuguagaaccgagcuaU......................................................................    25    1    Mm3
...................guacUcuguagaaccgagcuag......................................................................    1     1    Mm3
...................guacccuUuagaaccgagcuag......................................................................    1     1    Mm3
...................guacccuguagaaccgaAcuag......................................................................    6     1    Mm3
...................guacccuguaAaaccgagcuag......................................................................    1     1    Mm3
...................guacccuguagaUccgagcuagu.....................................................................    1     1    Mm3
...................guaUccuguagaaccgagcuagu.....................................................................    1     1    Mm3
...................guacccuguagaaccUagcuagu.....................................................................    1     1    Mm3
...................guacccCguagaaccgagcuagu.....................................................................    2     1    Mm3
...................guacccuguagaaccgagcuUgu.....................................................................    2     1    Mm3
...................guacccuUuagaaccgagcuagu.....................................................................    1     1    Mm3
...................guacccugCagaaccgagcuagu.....................................................................    2     1    Mm3
...................guaGccuguagaaccgagcuagu.....................................................................    1     1    Mm3
...................guUcccuguagaaccgagcuagu.....................................................................    1     1    Mm3
...................guacccuguagaaccgagcuagG.....................................................................    12    1    Mm3
...................Nuacccuguagaaccgagcuagu.....................................................................    12    1    Mm3
...................guacAcuguagaaccgagcuagu.....................................................................    3     1    Mm3
...................guacccuguGgaaccgagcuagu.....................................................................    5     1    Mm3
...................guacccuguagaaccgagUuagu.....................................................................    3     1    Mm3
...................Uuacccuguagaaccgagcuagu.....................................................................    38    1    Mm3
...................guacccuguagGaccgagcuagu.....................................................................    1     1    Mm3
...................guacccuguagaaAcgagcuagu.....................................................................    2     1    Mm3
...................guacccuguagaaccgagcuGgu.....................................................................    29    1    Mm3
...................guacccuguaCaaccgagcuagu.....................................................................    9     1    Mm3
...................guacccuguaUaaccgagcuagu.....................................................................    15    1    Mm3
...................gAacccuguagaaccgagcuagu.....................................................................    4     1    Mm3
...................guaccNuguagaaccgagcuagu.....................................................................    3     1    Mm3
...................Cuacccuguagaaccgagcuagu.....................................................................    207   1    Mm3
...................guCcccuguagaaccgagcuagu.....................................................................    3     1    Mm3
...................guacccuguagaaccgCgcuagu.....................................................................    38    1    Mm3
```

Mature **Star**

aaauguggugaguuucugug**uacccuguagaaccgagcuagu**guaucgaguucgaaacuca**caagccauucuacaguguaca**aagaaacgucgcuucuuucaacaaugaga			
...................guacccuguagaaccgaCcuagu...	53	1	Mm3
...................guacccuguagUaccgagcuagu...	2	1	Mm3
...................guacUcuguagaaccgagcuagu...	5	1	Mm3
...................guacccuguagaaccgagcuagA...	15	1	Mm3
...................guacccuguagaaccgUgcuagu...	24	1	Mm3
...................gCacccuguagaaccgagcuagu...	2	1	Mm3
...................guacccuguagaaccgagcuaCu...	1	1	Mm3
...................guacccuguagaaccgagcuagu...	13171	0	Mm3
...................guacGcuguagaaccgagcuagu...	1	1	Mm3
...................guacccuguagaaccgGgcuagu...	9	1	Mm3
...................guacccuguagaaccgagcuaUu...	5	1	Mm3
...................guacccuguagaaUcgagcuagu...	2	1	Mm3
...................guacccuguagaaccgagcGagu...	364	1	Mm3
...................guaccUuguagaaccgagcuagu...	4	1	Mm3
...................Auacccuguagaaccgagcuagu...	19069	1	Mm3
...................guacccuguagaaccgagcCagu...	2	1	Mm3
...................guacccuguaAaaccgagcuagu...	13	1	Mm3
...................guacccuguagaaccgagcuCgu...	2	1	Mm3
...................guaAccuguagaaccgagcuagu...	1	1	Mm3
...................guacccuguagaaccgagcuaAu...	1	1	Mm3
...................guacccuguagaaccgaAcuagu...	50	1	Mm3
...................guacccuguagaacUgagcuagu...	3	1	Mm3
...................guacccuguagaGccgagcuagu...	5	1	Mm3
...................guacccuguagaaccgaUcuagu...	69	1	Mm3
...................gGacccuguagaaccgagcuagu...	2	1	Mm3
...................guacccuguagaaccgagcuagC...	4	1	Mm3
...................guacccuguagaaccgagcuaguU..	621	1	Mm3
...................guacccuguagaaccgagcuagAg..	1	1	Mm3
...................guacccuguagaaccgUgcuagug..	3	1	Mm3
...................guacccuguagaaccgagcuGgug..	2	1	Mm3
...................guacccuguagaaccgaAcuagug..	4	1	Mm3
...................guacccuguaCaaccgagcuagug..	1	1	Mm3
...................Auacccuguagaaccgagcuagug..	547	1	Mm3
...................guacccuguagaaccgagcuaguC..	1	1	Mm3
...................guacccuguagaaccgagcuaguA..	16	1	Mm3
...................guacccGguagaaccgagcuagug..	1	1	Mm3
...................guacccuguagaaccgagcGagug..	4	1	Mm3
...................guacccuguagaaccgagcuagug..	1064	0	Mm3
...................guacccugAagaaccgagcuagug..	1	1	Mm3
...................guacccuguagaaccgaUcuagug..	4	1	Mm3
...................guacccuguagaaccgaCcuagug..	3	1	Mm3
...................guacccuguagaaccgGgcuagug..	2	1	Mm3
...................guacccuguaAaaccgagcuagug..	1	1	Mm3
...................Cuacccuguagaaccgagcuagug..	18	1	Mm3
...................guCcccuguagaaccgagcuagug..	1	1	Mm3
...................guacccuguaUaaccgagcuagug..	1	1	Mm3
...................guacccuguagaaccgCgcuagug..	1	1	Mm3
...................Auacccuguagaaccgagcuagugu...	312	1	Mm3
...................guacccuguagaaccgagcuagugu...	760	0	Mm3
...................guacccuguagaaccgagcuagugA...	21	1	Mm3
...................guacccuguagaaccgagcGagugu...	3	1	Mm3
...................guacccuguagaaccgagcuagugC...	3	1	Mm3
...................gGacccuguagaaccgagcuagugu...	1	1	Mm3
...................guacccuguagaaccgUgcuagugu...	3	1	Mm3
...................guacccuguagaaccgagcuagugG...	43	1	Mm3
...................guacccuguagaaccgagcuaguUu...	47	1	Mm3
...................guacccuguagaaccgCgcuagugu...	2	1	Mm3
...................guacccuguagaaccgagcuaguAu...	2	1	Mm3
...................guacccuguagaaccgaAcuagugu...	4	1	Mm3
...................guacccuguagaaccgaUcuagugu...	2	1	Mm3
...................guacccuguagaaccgagcuGgugu...	5	1	Mm3
...................guacccuguagaaUcgagcuagugu...	1	1	Mm3
...................Cuacccuguagaaccgagcuagugu...	10	1	Mm3
...................guacccuguagaaccgaCcuagugu...	4	1	Mm3
...................guacccuguagaaccgagcuagCgu...	1	1	Mm3
...................Nuacccuguagaaccgagcuagugu...	1	1	Mm3
...................Uuacccuguagaaccgagcuagugu...	1	1	Mm3
...................guacccuguagaaccgagcuaguguU..	34	1	Mm3
...................guacccuguagaaccgagcuagugua..	7	0	Mm3
...................guacccuguagaaccgagcuaguguUu...	12	1	Mm3

Mature **Star**

```
aaauguggugaguuucuguguacccuguagaaccgagcuaguguaucgaguucgaaacucacaagccauucuacaguguacaaagaaacgucgcuucuuucaacaaugaga
...................guacccuguagaaccgagcuaguguau.................................................................  1     0  Mm3
...................guacccuguagaaccgagcuaguguauc................................................................  12    0  Mm3
...................Auacccuguagaaccgagcuaguguauc................................................................  1     1  Mm3
...................guacccuguagaaccgagcuaguguaucgagu............................................................  1     0  Mm3
...................guacccuguagaaccgagcuaguguaucgaguu...........................................................  1     0  Mm3
...................guacccuguagaaccgagcuaguguaucgaguuc..........................................................  1     0  Mm3
....................uacccuguagaacUgagc.........................................................................  1     1  Mm3
....................uacccuguagaaccgagc.........................................................................  330   0  Mm3
....................uacccuguagaaccgagU.........................................................................  6     1  Mm3
....................uacccuguagaacGgagc.........................................................................  1     1  Mm3
....................uacccuguagaaccgagG.........................................................................  3     1  Mm3
....................uacccuguagUaccgagc.........................................................................  1     1  Mm3
....................uacccuguagaaccgaCc.........................................................................  2     1  Mm3
....................uacccuguagaaccgagA.........................................................................  12    1  Mm3
....................uacccCguagaaccgagc.........................................................................  1     1  Mm3
....................uUcccuguagaaccgagcu........................................................................  1     1  Mm3
....................uacccuguagCaccgagcu........................................................................  1     1  Mm3
....................Nacccuguagaaccgagcu........................................................................  1     1  Mm3
....................uacccuguagGaccgagcu........................................................................  2     1  Mm3
....................uacccuguagaaccgaUcu........................................................................  4     1  Mm3
....................uacccuguagaaccgagcu........................................................................  1709  0  Mm3
....................uacccuguagaaccgaAcu........................................................................  4     1  Mm3
....................uacccuguGgaaccgagcu........................................................................  1     1  Mm3
....................uacccuguagaaccgagcA........................................................................  16    1  Mm3
....................uacccuguaUaaccgagcu........................................................................  1     1  Mm3
....................uacccugAagaaccgagcu........................................................................  1     1  Mm3
....................uaGccuguagaaccgagcu........................................................................  1     1  Mm3
....................uacccuguagaaccAagcu........................................................................  1     1  Mm3
....................uacccuguagaGccgagcu........................................................................  1     1  Mm3
....................uacccuguagaaccgagUu........................................................................  12    1  Mm3
....................uacccuguagaaccgagAu........................................................................  16    1  Mm3
....................uacAcuguagaaccgagcu........................................................................  1     1  Mm3
....................uacccugCagaaccgagcu........................................................................  1     1  Mm3
....................uacccuguagaaccgaCcu........................................................................  3     1  Mm3
....................uacccuguagaaccgagGu........................................................................  5     1  Mm3
....................uacccuguagUaccgagcua.......................................................................  3     1  Mm3
....................uacccuguagaaccgagcuG.......................................................................  3     1  Mm3
....................uacccuguagaaccgagcuU.......................................................................  196   1  Mm3
....................uacccuguagCaccgagcua.......................................................................  1     1  Mm3
....................uUcccuguagaaccgagcua.......................................................................  1     1  Mm3
....................uacccugAagaaccgagcua.......................................................................  1     1  Mm3
....................uacccuNuagaaccgagcua.......................................................................  1     1  Mm3
....................uacccuguagaUccgagcua.......................................................................  2     1  Mm3
....................uacccuguagaaccgagcAa.......................................................................  2     1  Mm3
....................Cacccuguagaaccgagcua.......................................................................  1     1  Mm3
....................uacccuguagaCccgagcua.......................................................................  1     1  Mm3
....................Aacccuguagaaccgagcua.......................................................................  1     1  Mm3
....................uacccuguagaaccgagcuC.......................................................................  1     1  Mm3
....................uacccuguagaaccgagcua.......................................................................  1093  0  Mm3
....................uacccuguagaaccgagAua.......................................................................  8     1  Mm3
....................uacccuguagaaccgagGua.......................................................................  4     1  Mm3
....................uacccuguagaaccgaUcua.......................................................................  2     1  Mm3
....................uacccuguagaaccgagUua.......................................................................  8     1  Mm3
....................uacccuguagaaUcgagcua.......................................................................  1     1  Mm3
....................uacccuguagaaccgaCcua.......................................................................  3     1  Mm3
....................uacccuUuagaaccgagcuag......................................................................  3     1  Mm3
....................uaGccuguagaaccgagcuag......................................................................  4     1  Mm3
....................uacccuguagaaccgagcuaC......................................................................  1     1  Mm3
....................uacccuguagUaccgagcuag......................................................................  20    1  Mm3
....................uacccuguagaaccgaCcuag......................................................................  75    1  Mm3
....................Nacccuguagaaccgagcuag......................................................................  22    1  Mm3
....................uacccuguagaaccgagAuag......................................................................  272   1  Mm3
....................uacccuguagaaccgaAcuag......................................................................  54    1  Mm3
....................uaccUuguagaaccgagcuag......................................................................  4     1  Mm3
....................uNcccuguagaaccgagcuag......................................................................  1     1  Mm3
....................uacccuguagaaccgagcuCg......................................................................  2     1  Mm3
....................uCcccuguagaaccgagcuag......................................................................  1     1  Mm3
....................uacAcuguagaaccgagcuag......................................................................  7     1  Mm3
....................uacccuguagCaccgagcuag......................................................................  28    1  Mm3
....................Aacccuguagaaccgagcuag......................................................................  12    1  Mm3
```

```
                    Mature                    Star
aaauguggugaguuucugug uacccuguagaaccgagcuagu guaucgaguucgaaacuca caagccauucuacaguguaca aagaaacgucgcuucuuucaacaaugaga
....................uacccAguagaaccgagcuag......................................................................  2       1  Mm3
....................uacccuguagaaccgagcCag......................................................................  2       1  Mm3
....................uacccuguagaaccgagcuaU......................................................................  836     1  Mm3
....................uacccuguagaaccgCgcuag......................................................................  3       1  Mm3
....................uacccuguagaaccgagUuag......................................................................  164     1  Mm3
....................uacccuguagaGccgagcuag......................................................................  3       1  Mm3
....................Cacccuguagaaccgagcuag......................................................................  2       1  Mm3
....................uacccuguaUaaccgagcuag......................................................................  2       1  Mm3
....................uacccuNuagaaccgagcuag......................................................................  3       1  Mm3
....................uacccuguagaaccAagcuag......................................................................  1       1  Mm3
....................uacccuguagaaccgagcuaA......................................................................  81      1  Mm3
....................uacccuguagGaccgagcuag......................................................................  40      1  Mm3
....................Gacccuguagaaccgagcuag......................................................................  1       1  Mm3
....................uacccuguUgaaccgagcuag......................................................................  2       1  Mm3
....................uaAccuguagaaccgagcuag......................................................................  4       1  Mm3
....................uacccuguagaaccgagcuUg......................................................................  10      1  Mm3
....................uacccGguagaaccgagcuag......................................................................  3       1  Mm3
....................uacccuAuagaaccgagcuag......................................................................  1       1  Mm3
....................uacccuguagaaccgagcuGg......................................................................  51      1  Mm3
....................uacccuguagaacAgagcuag......................................................................  6       1  Mm3
....................uacccugAagaaccgagcuag......................................................................  1       1  Mm3
....................uacccuCuagaaccgagcuag......................................................................  1       1  Mm3
....................uacccuguagaaccgagcAag......................................................................  3       1  Mm3
....................uacccCguagaaccgagcuag......................................................................  7       1  Mm3
....................uacUcuguagaaccgagcuag......................................................................  5       1  Mm3
....................uacccuguagaaccgUgcuag......................................................................  7       1  Mm3
....................uacccuguagaaccgGgcuag......................................................................  1       1  Mm3
....................uacccuguagaaccgaUcuag......................................................................  57      1  Mm3
....................uacGcuguagaaccgagcuag......................................................................  2       1  Mm3
....................uacccugCagaaccgagcuag......................................................................  3       1  Mm3
....................uacccuguagaaccCagcuag......................................................................  1       1  Mm3
....................uaccGuguagaaccgagcuag......................................................................  1       1  Mm3
....................uacccNguagaaccgagcuag......................................................................  10      1  Mm3
....................uGcccuguagaaccgagcuag......................................................................  4       1  Mm3
....................uacccuguGgaaccgagcuag......................................................................  2       1  Mm3
....................uacccuguagaaUcgagcuag......................................................................  10      1  Mm3
....................uacccuguagaCccgagcuag......................................................................  1       1  Mm3
....................uUcccuguagaaccgagcuag......................................................................  5       1  Mm3
....................uacccuguagaaccgagGuag......................................................................  104     1  Mm3
....................uacccuguagaacUgagcuag......................................................................  7       1  Mm3
....................uacccuguagaUccgagcuag......................................................................  26      1  Mm3
....................uacccuguagaaccUagcuag......................................................................  1       1  Mm3
....................uaUccuguagaaccgagcuag......................................................................  4       1  Mm3
....................uacccuguagaaccgagcuag......................................................................  29023   0  Mm3
....................uacccuguagaaAcgagcuag......................................................................  2       1  Mm3
....................uacccuguaAaaccgagcuagu.....................................................................  49      1  Mm3
....................uacccuAuagaaccgagcuagu.....................................................................  75      1  Mm3
....................uacccuNuagaaccgagcuagu.....................................................................  32      1  Mm3
....................uaAccuguagaaccgagcuagu.....................................................................  123     1  Mm3
....................uacccuguGgaaccgagcuagu.....................................................................  93      1  Mm3
....................uacccuguagaaccgGgcuagu.....................................................................  71      1  Mm3
....................Gacccuguagaaccgagcuagu.....................................................................  17      1  Mm3
....................uacccuguagaaccgagcuagu.....................................................................  657693  0  Mm3
....................uaGccuguagaaccgagcuagu.....................................................................  130     1  Mm3
....................uacccuguagaaccUagcuagu.....................................................................  52      1  Mm3
....................uacAcuguagaaccgagcuagu.....................................................................  198     1  Mm3
....................uacccuguagaaccgagcuCgu.....................................................................  40      1  Mm3
....................uacccuguagaaccgagGuagu.....................................................................  2244    1  Mm3
....................uacccuguagaaAcgagcuagu.....................................................................  21      1  Mm3
....................uacccuguagaaccgaAcuagu.....................................................................  1312    1  Mm3
....................uGcccuguagaaccgagcuagu.....................................................................  145     1  Mm3
....................uacccuguagaaccgagcuUgu.....................................................................  352     1  Mm3
....................uacccuguagaaccgagcuagG.....................................................................  680     1  Mm3
....................uacccugAagaaccgagcuagu.....................................................................  41      1  Mm3
....................uacccuguagaaccgagcCagu.....................................................................  34      1  Mm3
....................uacccuguaCaaccgagcuagu.....................................................................  27      1  Mm3
....................uNcccuguagaaccgagcuagu.....................................................................  4       1  Mm3
....................uacccuguagaaccgaCcuagu.....................................................................  1433    1  Mm3
....................uacccuguagCaccgagcuagu.....................................................................  592     1  Mm3
....................uaccAuguagaaccgagcuagu.....................................................................  45      1  Mm3
```

```
                   Mature                    Star
aaauguggugaguuucuguguacccuguagaaccgagcuaguguaucgaguucgaaacucacaagccauucuacaguguacaaagaaacgucgcuucuuucaacaaugaga
....................Nacccuguagaaccgagcuagu.....................................................................  316    1  Mm3
....................uacccuguaUaaccgagcuagu.....................................................................  62     1  Mm3
....................uacccuguagaaccgagcuaUu.....................................................................  157    1  Mm3
....................uacccugCagaaccgagcuagu.....................................................................  67     1  Mm3
....................uacccuguagaaUcgagcuagu.....................................................................  144    1  Mm3
....................uacccuguagaaccgaUcuagu.....................................................................  1223   1  Mm3
....................uacccuguagaacGgagcuagu.....................................................................  31     1  Mm3
....................uacccuUuagaaccgagcuagu.....................................................................  72     1  Mm3
....................uaccGuguagaaccgagcuagu.....................................................................  28     1  Mm3
....................uacUcuguagaaccgagcuagu.....................................................................  130    1  Mm3
....................uacccuguagaUccgagcuagu.....................................................................  461    1  Mm3
....................uacccCguagaaccgagcuagu.....................................................................  82     1  Mm3
....................uacccuguagaaGcgagcuagu.....................................................................  10     1  Mm3
....................uacccuguagaaccgagcNagu.....................................................................  1      1  Mm3
....................uacccuguagaacUgagcuagu.....................................................................  202    1  Mm3
....................uacccuguagaaccgagUuagu.....................................................................  3136   1  Mm3
....................uacccNguagaaccgagcuagu.....................................................................  120    1  Mm3
....................uacccuguagaaccgagcuaAu.....................................................................  52     1  Mm3
....................uacccuguagaaccgagcuagC.....................................................................  184    1  Mm3
....................Cacccuguagaaccgagcuagu.....................................................................  126    1  Mm3
....................uacccuguagaaccgagAuagu.....................................................................  6089   1  Mm3
....................uacccAguagaaccgagcuagu.....................................................................  36     1  Mm3
....................uacccuguagUaccgagcuagu.....................................................................  650    1  Mm3
....................uacccuguagGaccgagcuagu.....................................................................  734    1  Mm3
....................uacccuguagaaccAagcuagu.....................................................................  88     1  Mm3
....................uaccUuguagaaccgagcuagu.....................................................................  102    1  Mm3
....................uacccGguagaaccgagcuagu.....................................................................  26     1  Mm3
....................uacccuguagaCccgagcuagu.....................................................................  23     1  Mm3
....................uUcccuguagaaccgagcuagu.....................................................................  139    1  Mm3
....................uacccuguagaaccgUgcuagu.....................................................................  138    1  Mm3
....................uacccugGagaaccgagcuagu.....................................................................  10     1  Mm3
....................uacGcuguagaaccgagcuagu.....................................................................  55     1  Mm3
....................uacccuguagaacAgagcuagu.....................................................................  147    1  Mm3
....................uacccuguCgaaccgagcuagu.....................................................................  16     1  Mm3
....................uacccuguagaaccgCgcuagu.....................................................................  38     1  Mm3
....................uacccuCuagaaccgagcuagu.....................................................................  22     1  Mm3
....................uacccuguagaaccgagcuaCu.....................................................................  35     1  Mm3
....................uacccuguUgaaccgagcuagu.....................................................................  87     1  Mm3
....................uacccuguagaaccCagcuagu.....................................................................  21     1  Mm3
....................uacccuguagaaccgagNuagu.....................................................................  2      1  Mm3
....................uacccuguagaaccgagcAagu.....................................................................  16     1  Mm3
....................uCccuguagaaccgagcuagu.....................................................................  44     1  Mm3
....................uacccuguagaaccgagcuagA.....................................................................  859    1  Mm3
....................uacccuguagaaccgagcuGgu.....................................................................  794    1  Mm3
....................uacccuguagaGccgagcuagu.....................................................................  74     1  Mm3
....................uaUccuguagaaccgagcuagu.....................................................................  108    1  Mm3
....................uaNccuguagaaccgagcuagu.....................................................................  3      1  Mm3
....................Aacccuguagaaccgagcuagu.....................................................................  233    1  Mm3
....................uacccuguagaaccgagGuagug....................................................................  128    1  Mm3
....................uacccuguagaaccgagcAagug....................................................................  2      1  Mm3
....................uacccuguagaaccgagcuCgug....................................................................  1      1  Mm3
....................uacAcuguagaaccgagcuagug....................................................................  18     1  Mm3
....................uacccuguagaaccgagcuaguC....................................................................  15     1  Mm3
....................uacccuguagaaccgagcCagug....................................................................  1      1  Mm3
....................uacccuguagaaccgagcuaguA....................................................................  2306   1  Mm3
....................uacccuguagaaccgagAuagug....................................................................  319    1  Mm3
....................uacccuguagaaUcgagcuagug....................................................................  9      1  Mm3
....................uacccuguUgaaccgagcuagug....................................................................  7      1  Mm3
....................uacccuguaUaaccgagcuagug....................................................................  6      1  Mm3
....................uacccuguagaaccgagcuaguU....................................................................  11424  1  Mm3
....................uacccuNuagaaccgagcuagug....................................................................  2      1  Mm3
....................uacccuguagaaccgCgcuagug....................................................................  2      1  Mm3
....................uacccuguagaaccgagcuaUug....................................................................  5      1  Mm3
....................uacccuguagaUccgagcuagug....................................................................  18     1  Mm3
....................uacccuguagaaccAagcuagug....................................................................  1      1  Mm3
....................uacccuguagaaccgaAcuagug....................................................................  88     1  Mm3
....................uacccuguagaacAgagcuagug....................................................................  8      1  Mm3
....................uaUccuguagaaccgagcuagug....................................................................  7      1  Mm3
....................uacccuguagaaccgagcuaCug....................................................................  2      1  Mm3
....................uacccuguagCaccgagcuagug....................................................................  26     1  Mm3
```

```
                    Mature                                    Star
aaauguggugaguuucugugaacccuguagaaccgagcuaguguaucgaguucgaaacucacaagccauucuacaguguacaaagaaacgucgcuucuuucaacaaugaga
....................uacccuguagaaccgagUuagug....................................................................    159     1   Mm3
....................uaccAuguagaaccgagcuagug....................................................................    4       1   Mm3
....................uacccuguagaaccgUgcuagug....................................................................    5       1   Mm3
....................uacccuguaAaaccgagcuagug....................................................................    3       1   Mm3
....................uacccuguagaaccgagcuagCg....................................................................    6       1   Mm3
....................uUcccuguagaaccgagcuagug....................................................................    4       1   Mm3
....................uCcccuguagaaccgagcuagug....................................................................    1       1   Mm3
....................uacccAguagaaccgagcuagug....................................................................    5       1   Mm3
....................uacccuguCgaaccgagcuagug....................................................................    1       1   Mm3
....................uacccuguagaaccgaCcuagug....................................................................    69      1   Mm3
....................uaccGuguagaaccgagcuagug....................................................................    1       1   Mm3
....................uacccuCuagaaccgagcuagug....................................................................    1       1   Mm3
....................uacccuguagaaccgagcuagAg....................................................................    7       1   Mm3
....................uacccuguagaaccUagcuagug....................................................................    5       1   Mm3
....................uacccuguagaCccgagcuagug....................................................................    1       1   Mm3
....................uacccuguGgaaccgagcuagug....................................................................    4       1   Mm3
....................uacccuguagaaccgagcuaAug....................................................................    2       1   Mm3
....................Aacccuguagaaccgagcuagug....................................................................    11      1   Mm3
....................uGcccuguagaaccgagcuagug....................................................................    8       1   Mm3
....................uacccugGagaaccgagcuagug....................................................................    2       1   Mm3
....................Gacccuguagaaccgagcuagug....................................................................    1       1   Mm3
....................uaccUuguagaaccgagcuagug....................................................................    3       1   Mm3
....................uaAccuguagaaccgagcuagug....................................................................    10      1   Mm3
....................uacccuguagaaccCagcuagug....................................................................    1       1   Mm3
....................uacccuguagGaccgagcuagug....................................................................    40      1   Mm3
....................uacGcuguagaaccgagcuagug....................................................................    5       1   Mm3
....................uacccugCagaaccgagcuagug....................................................................    1       1   Mm3
....................uacccuAuagaaccgagcuagug....................................................................    2       1   Mm3
....................uacccuguagaaGcgagcuagug....................................................................    1       1   Mm3
....................Cacccuguagaaccgagcuagug....................................................................    3       1   Mm3
....................uacccugAagaaccgagcuagug....................................................................    1       1   Mm3
....................uacccuguagaaccgagcuUgug....................................................................    9       1   Mm3
....................uacUcuguagaaccgagcuagug....................................................................    6       1   Mm3
....................uacccuguagaGccgagcuagug....................................................................    2       1   Mm3
....................uaGccuguagaaccgagcuagug....................................................................    13      1   Mm3
....................uacccuguagaacUgagcuagug....................................................................    14      1   Mm3
....................uacccGguagaaccgagcuagug....................................................................    2       1   Mm3
....................uacccuguagaaAcgagcuagug....................................................................    3       1   Mm3
....................uacccuguagaacGgagcuagug....................................................................    2       1   Mm3
....................uacccuguagaaccgaUcuagug....................................................................    64      1   Mm3
....................uacccuguagUaccgagcuagug....................................................................    32      1   Mm3
....................uacccuguagaaccgagcuagug....................................................................    36336   0   Mm3
....................Nacccuguagaaccgagcuagug....................................................................    17      1   Mm3
....................uacccuguagaaccgagcuGgug....................................................................    62      1   Mm3
....................uacccuguagaaccgagcuagGg....................................................................    15      1   Mm3
....................uacccuguagaaccgGgcuagug....................................................................    7       1   Mm3
....................uacccuUuagaaccgagcuagug....................................................................    4       1   Mm3
....................uacccCguagaaccgagcuagug....................................................................    1       1   Mm3
....................uacccuguaCaaccgagcuagug....................................................................    1       1   Mm3
....................uacccNguagaaccgagcuagug....................................................................    9       1   Mm3
....................uacccuguagaGccgagcuagugu...................................................................    4       1   Mm3
....................uacccuguagaacUgagcuagugu...................................................................    5       1   Mm3
....................uacccuguagaCccgagcuagugu...................................................................    1       1   Mm3
....................uacccuguagaacAgagcuagugu...................................................................    7       1   Mm3
....................uacccuguagaaccgagcuaAugu...................................................................    3       1   Mm3
....................uacccuguagaUccgagcuagugu...................................................................    14      1   Mm3
....................Aacccuguagaaccgagcuagugu...................................................................    10      1   Mm3
....................uaAccuguagaaccgagcuagugu...................................................................    9       1   Mm3
....................uacccuguagaaGcgagcuagugu...................................................................    1       1   Mm3
....................uacccuguagaaccgaUcuagugu...................................................................    57      1   Mm3
....................uGcccuguagaaccgagcuagugu...................................................................    9       1   Mm3
....................uacccuguagaaccgaAcuagugu...................................................................    71      1   Mm3
....................uacccuguagaaAcgagcuagugu...................................................................    1       1   Mm3
....................uacccuguaCaaccgagcuagugu...................................................................    2       1   Mm3
....................uacccuguagaaccgagGuagugu...................................................................    104     1   Mm3
....................uacccuguagaaccgagUuagugu...................................................................    157     1   Mm3
....................uacccuguagaaccgagcuaguCu...................................................................    5       1   Mm3
....................uacccGguagaaccgagcuagugu...................................................................    1       1   Mm3
....................uacccugCagaaccgagcuagugu...................................................................    3       1   Mm3
....................uacccuguagaaccgagcGagugu...................................................................    74      1   Mm3
```

Mature **Star**

```
aaauguggugaguuucugugacccuguagaaccgagcuaguguaucgaguucgaaacucacaagccauucuacaguguacaaagaaacgucgcuucuuucaacaaugaga
```

```
aaauguggugaguuucuguguacccuguagaaccgagcuaguguaucgaguucgaaacucacaagccauucuacaguguacaaagaaacgucgcuucuuucaacaaugaga
....................uacccuguagaaccgGgcuagugu...................................................................  1      1  Mm3
....................uacccAguagaaccgagcuagugu...................................................................  2      1  Mm3
....................uacAcuguagaaccgagcuagugu...................................................................  15     1  Mm3
....................uacccuguagaaccgagcuagCgu...................................................................  3      1  Mm3
....................uacGcuguagaaccgagcuagugu...................................................................  3      1  Mm3
....................uacccuguagGaccgagcuagugu...................................................................  34     1  Mm3
....................uacccuguaAaaccgagcuagugu...................................................................  1      1  Mm3
....................uacccuguagaaccAagcuagugu...................................................................  10     1  Mm3
....................uacccuNuagaaccgagcuagugu...................................................................  1      1  Mm3
....................uacccuguagUaccgagcuagugu...................................................................  26     1  Mm3
....................uacccuguagaaccgagAuagugu...................................................................  349    1  Mm3
....................uacccuguagaaccgaCcuagugu...................................................................  75     1  Mm3
....................uacccuguagaaUcgagcuagugu...................................................................  9      1  Mm3
....................uacccuguagaaccCagcuagugu...................................................................  1      1  Mm3
....................uacccuguagaaccUagcuagugu...................................................................  6      1  Mm3
....................uacccuguaUaaccgagcuagugu...................................................................  5      1  Mm3
....................uacccuguagCaccgagcuagugu...................................................................  38     1  Mm3
....................uaUccuguagaaccgagcuagugu...................................................................  2      1  Mm3
....................uacccuguagaaccgagcuaguUu...................................................................  1455   1  Mm3
....................Nacccuguagaaccgagcuagugu...................................................................  14     1  Mm3
....................Cacccuguagaaccgagcuagugu...................................................................  2      1  Mm3
....................uaccAuguagaaccgagcuagugu...................................................................  4      1  Mm3
....................uacccuAuagaaccgagcuagugu...................................................................  1      1  Mm3
....................uacccNguagaaccgagcuagugu...................................................................  2      1  Mm3
....................uacccuguagaaccgagcuaguAu...................................................................  645    1  Mm3
....................uacccuguGgaaccgagcuagugu...................................................................  7      1  Mm3
....................uacccuguagaaccgagcuUgugu...................................................................  10     1  Mm3
....................uacccuguagaaccgagcuGgugu...................................................................  55     1  Mm3
....................uacccuUuagaaccgagcuagugu...................................................................  3      1  Mm3
....................uacccuguagaaccgagcuagugG...................................................................  1871   1  Mm3
....................uacccugAagaaccgagcuagugu...................................................................  1      1  Mm3
....................uacccuguagaaccgagcuCgugu...................................................................  1      1  Mm3
....................uaccGuguagaaccgagcuagugu...................................................................  1      1  Mm3
....................uacccuguagaaccgagcuagugC...................................................................  40     1  Mm3
....................uacccuguagaaccgagcuagAgu...................................................................  5      1  Mm3
....................uacccuguagaaccgagcuaUugu...................................................................  3      1  Mm3
....................uaGccuguagaaccgagcuagugu...................................................................  9      1  Mm3
....................uacccuguagaaccgagcuagugA...................................................................  1278   1  Mm3
....................Gacccuguagaaccgagcuagugu...................................................................  2      1  Mm3
....................uacUcuguagaaccgagcuagugu...................................................................  5      1  Mm3
....................uUccuguagaaccgagcuagugu...................................................................  10     1  Mm3
....................uaccUuguagaaccgagcuagugu...................................................................  11     1  Mm3
....................uacccuguagaaccgUgcuagugu...................................................................  8      1  Mm3
....................uCccuguagaaccgagcuagugu...................................................................  4      1  Mm3
....................uacccuguUgaaccgagcuagugu...................................................................  3      1  Mm3
....................uacccuguagaaccgagcuagugu...................................................................  35354  0  Mm3
....................uacccuguagaaccgagcuaCugu...................................................................  2      1  Mm3
....................uacccuguagaaccgagcuagugAa..................................................................  10     1  Mm3
....................uacccuguagaaccgagcuaguguG..................................................................  3      1  Mm3
....................uacccuguagCaccgagcuagugua..................................................................  1      1  Mm3
....................uacccuguagaaccgagcuaguguC..................................................................  2      1  Mm3
....................uacccuguagaaccgagcuGgugua..................................................................  1      1  Mm3
....................uacccuguagaaccgagcuagugua..................................................................  27     0  Mm3
....................uacccuguagaaccgagGuagugua..................................................................  1      1  Mm3
....................uacccuguagaaccgagcuaguguU..................................................................  750    1  Mm3
....................uacccuguagaaccgagcuaguguCu.................................................................  1      1  Mm3
....................uacccuguagaaccgagcuaguguau.................................................................  25     0  Mm3
....................uacccuguagaaccgagcuaguguUu.................................................................  69     1  Mm3
....................uacccuguagaaccgaCcuaguguauc................................................................  1      1  Mm3
....................uacccuguagCaccgagcuaguguauc................................................................  1      1  Mm3
....................uacccuguUgaaccgagcuaguguauc................................................................  1      1  Mm3
....................uacccuguagaaccgagGuaguguauc................................................................  1      1  Mm3
....................uacccuguagaaccgagcuaguguauc................................................................  113    0  Mm3
....................uacccuguagaaccgagcuaguguauA...............................................................  1      1  Mm3
....................uacccuguagaaccgagcuaguguauU...............................................................  2      1  Mm3
....................uacccuguagaaccgagcuaguguaucU..............................................................  1      1  Mm3
....................uacccuguagaaccgagcuaguguaucg..............................................................  30     0  Mm3
.....................acccuguagaaccgagcu........................................................................  16     0  Mm3
.....................acccuguagaaccgagcua.......................................................................  9      0  Mm3
.....................acccuguagaaccgagcuU.......................................................................  1      1  Mm3
```

```
                    Mature                     Star
aaauguggugaguuucuguguacccuguagaaccgagcuaguguaucgaguucgaaacucacaagccauucuacaguguacaaagaaacgucgcuucuuucaacaaugaga
.....................acccuguagaaccgagcuGg......................................................................    1     1    Mm3
.....................acccuguagaaccgagcCag......................................................................    1     1    Mm3
.....................acccuguagaUccgagcuag......................................................................    1     1    Mm3
.....................acccuguagaacUgagcuag......................................................................    1     1    Mm3
.....................acccuguaUaaccgagcuag......................................................................    1     1    Mm3
.....................acccuguagaaccgagcAag......................................................................    1     1    Mm3
.....................acccuguagaaUcgagcuag......................................................................    1     1    Mm3
.....................acccuguagaaccgagAuag......................................................................    1     1    Mm3
.....................acccuguagaaccgagcuag......................................................................    149   0    Mm3
.....................acccuguagaaccgagcuaU......................................................................    7     1    Mm3
.....................acccuUuagaaccgagcuag......................................................................    1     1    Mm3
.....................acccuguagaaccgagcuaA......................................................................    1     1    Mm3
.....................acccugCagaaccgagcuagu.....................................................................    1     1    Mm3
.....................Ucccuguagaaccgagcuagu.....................................................................    2     1    Mm3
.....................acccuguagaUccgagcuagu.....................................................................    6     1    Mm3
.....................acccCguagaaccgagcuagu.....................................................................    1     1    Mm3
.....................acccuguagaaccgagcuagA.....................................................................    5     1    Mm3
.....................acAcuguagaaccgagcuagu.....................................................................    1     1    Mm3
.....................acccuguagaaccgagcAagu.....................................................................    12    1    Mm3
.....................acccuguagaCccgagcuagu.....................................................................    11    1    Mm3
.....................acccuguagaaGcgagcuagu.....................................................................    3     1    Mm3
.....................acccuguagaaccgaUcuagu.....................................................................    1     1    Mm3
.....................acccuguagaaccgagcCagu.....................................................................    24    1    Mm3
.....................acccuguagaaccgagcuUgu.....................................................................    3     1    Mm3
.....................acccuguagaaccCagcuagu.....................................................................    2     1    Mm3
.....................acccuguagaaccgagcuCgu.....................................................................    6     1    Mm3
.....................acccuguagaaccgUgcuagu.....................................................................    1     1    Mm3
.....................acccuguagaGccgagcuagu.....................................................................    10    1    Mm3
.....................aGccuguagaaccgagcuagu.....................................................................    1     1    Mm3
.....................acccuguagaaccgagAuagu.....................................................................    7     1    Mm3
.....................acccuguagaaccgagcuaUu.....................................................................    2     1    Mm3
.....................acccuCuagaaccgagcuagu.....................................................................    1     1    Mm3
.....................aAccuguagaaccgagcuagu.....................................................................    1     1    Mm3
.....................acccuguGgaaccgagcuagu.....................................................................    1     1    Mm3
.....................acccuguagUaccgagcuagu.....................................................................    1     1    Mm3
.....................Ncccuguagaaccgagcuagu.....................................................................    2     1    Mm3
.....................acccuAuagaaccgagcuagu.....................................................................    1     1    Mm3
.....................acccuguagaaccgagcuagG.....................................................................    7     1    Mm3
.....................acccuguagaaccgagcuagu.....................................................................    3332  0    Mm3
.....................acccuguagaaccgagcuaCu.....................................................................    7     1    Mm3
.....................acccuguagaaccgagcuagC.....................................................................    2     1    Mm3
.....................acccuguaCaaccgagcuagu.....................................................................    1     1    Mm3
.....................acccuguCgaaccgagcuagu.....................................................................    2     1    Mm3
.....................acccuguagaaccgagcuGgu.....................................................................    5     1    Mm3
.....................acccuguagCaccgagcuagu.....................................................................    2     1    Mm3
.....................acccAguagaaccgagcuagu.....................................................................    1     1    Mm3
.....................acccugAagaaccgagcuagu.....................................................................    1     1    Mm3
.....................acccuguagaaccgCgcuagu.....................................................................    6     1    Mm3
.....................acccuguagaacUgagcuagu.....................................................................    2     1    Mm3
.....................acccuguagaaccgagGuagu.....................................................................    2     1    Mm3
.....................acccuguagaacAgagcuagu.....................................................................    3     1    Mm3
.....................aUccuguagaaccgagcuagu.....................................................................    1     1    Mm3
.....................acccuguaUaaccgagcuagu.....................................................................    4     1    Mm3
.....................acccuguagaaccgagcuaAu.....................................................................    2     1    Mm3
.....................acccuguagaaccgaCcuagu.....................................................................    1     1    Mm3
.....................acccuguagaaccgGgcuagu.....................................................................    3     1    Mm3
.....................acccuguagaaccgaAcuagu.....................................................................    2     1    Mm3
.....................acccuguCgaaccgagcuagug....................................................................    1     1    Mm3
.....................acccuguagaaccgagcuaguA....................................................................    15    1    Mm3
.....................acccuguagaaccgagcAagug....................................................................    1     1    Mm3
.....................acccuguagaaccgagcuGgug....................................................................    1     1    Mm3
.....................Ucccuguagaaccgagcuagug....................................................................    1     1    Mm3
.....................acccuguagaaccgagcuagug....................................................................    274   0    Mm3
.....................acccuguagaaccgagcuaguU....................................................................    70    1    Mm3
.....................accGuguagaaccgagcuagug....................................................................    1     1    Mm3
.....................acccCguagaaccgagcuagug....................................................................    1     1    Mm3
.....................acccuguagUaccgagcuagug....................................................................    1     1    Mm3
.....................acccuguagaUccgagcuagug....................................................................    1     1    Mm3
.....................acccuguagGaccgagcuagug....................................................................    1     1    Mm3
.....................acccuguagaGccgagcuagug....................................................................    1     1    Mm3
```

Mature Star

```
aaauguggugaguuucugugguacccuguagaaccgagcuaguguaucgaguucgaaacucacaagccauucuacaguguacaaagaaacgucgcuucuuucaacaaugaga
.....................acccugAagaaccgagcuagugu...................................................................	1	1	Mm3
.....................acccuguagaaccgagcuaguAu...................................................................	9	1	Mm3
.....................acccuguagaGccgagcuagugu...................................................................	2	1	Mm3
.....................acccuguagaaccgagGuagugu...................................................................	1	1	Mm3
.....................acccuguagaaccgagcuagugC...................................................................	1	1	Mm3
.....................acccuguagaaccgagAuagugu...................................................................	1	1	Mm3
.....................acccuguagaaccgagcuGgugu...................................................................	2	1	Mm3
.....................acccuguagaaccgCgcuagugu...................................................................	1	1	Mm3
.....................acGcuguagaaccgagcuagugu...................................................................	1	1	Mm3
.....................acccuguagaaccgagcAagugu...................................................................	2	1	Mm3
.....................acccuguagaaccgagcuagugA...................................................................	19	1	Mm3
.....................acccuguagaaccgagcGagugu...................................................................	1	1	Mm3
.....................acccuguagaaccgagcuagugG...................................................................	22	1	Mm3
.....................acccuguagaaccgagUuagugu...................................................................	1	1	Mm3
.....................acccuguagaaccgagcuaguUu...................................................................	5	1	Mm3
.....................acccuguagaaccgagcCagugu...................................................................	3	1	Mm3
.....................acccuguagaaccgagcuagugu...................................................................	368	0	Mm3
.....................acccuguagaaccgagcuagugua..................................................................	1	0	Mm3
.....................acccuguagaaccgagcuagugCa..................................................................	1	1	Mm3
.....................acccuguagaaccgagcuaguguU..................................................................	15	1	Mm3
.....................acccuguagaaccgagcuaguguUu.................................................................	4	1	Mm3
.....................acccuguagaaccgagcuaguguau.................................................................	1	0	Mm3
.....................acccuguagaaccgagcuaguUuauc................................................................	1	1	Mm3
.....................acccuguagaGccgagcuaguguauc................................................................	1	1	Mm3
.....................acccuguagaaccgagcuaguguauc................................................................	2	0	Mm3
.....................acccuguagaaccgagcuaguguaucg...............................................................	3	0	Mm3
......................cccuguagaaccgagcuag......................................................................	2	0	Mm3
......................cccuguagaaccgaCcuagu.....................................................................	1	1	Mm3
......................cccuguagaaccgagcuaUu.....................................................................	1	1	Mm3
......................cccuguagaaccgagcuGgu.....................................................................	1	1	Mm3
......................cccuguagaaccgagcuCgu.....................................................................	1	1	Mm3
......................Accuguagaaccgagcuagu.....................................................................	1	1	Mm3
......................cccuguagaaccgagcuagu.....................................................................	64	0	Mm3
......................cccuUuagaaccgagcuagu.....................................................................	1	1	Mm3
......................cccuguagaaGcgagcuagug....................................................................	1	1	Mm3
......................cccuguagaaccgagcuaguU....................................................................	1	1	Mm3
......................cccuguagaaccgagcuagug....................................................................	7	0	Mm3
......................cccuguagaaccgagcuagugA...................................................................	1	1	Mm3
......................cccuguagaaccgagcuagugu...................................................................	4	0	Mm3
......................cccuguagaaccgagcuGgugu...................................................................	1	1	Mm3
.......................ccuguagaaccgagcuagu.....................................................................	14	0	Mm3
.......................ccuguagaaccgagcuUgu.....................................................................	1	1	Mm3
.......................Ucuguagaaccgagcuagu.....................................................................	1	1	Mm3
.......................ccuguagaaccgagcuaguU....................................................................	1	1	Mm3
.......................ccuguagaaccgagcuagug....................................................................	1	0	Mm3
.......................ccuguagaaccgagcuagugG...................................................................	1	1	Mm3
........................cuguagaaccgagcuagu.....................................................................	1	0	Mm3
.............................................................caagccauucuacagugu................................	55	0	Mm3
.............................................................caagccauucuacagugA................................	2	1	Mm3
.............................................................caagccauucuacaguAu................................	1	1	Mm3
.............................................................caagccauucuacagugG................................	3	1	Mm3
.............................................................caagccauucuacagugCa...............................	1	1	Mm3
.............................................................caagccauucuacaguguU...............................	8	1	Mm3
.............................................................caagccauucCacagugua...............................	1	1	Mm3
.............................................................caagccauucuacagugua...............................	25	0	Mm3
.............................................................caagccauucuacaguguac..............................	682	0	Mm3
.............................................................caagccauucuacaguUuac..............................	2	1	Mm3
.............................................................caagccauucuacaguguaU..............................	1	1	Mm3
.............................................................caagccauuUuacaguguac..............................	1	1	Mm3
.............................................................caagccauucuacaCuguac..............................	1	1	Mm3
.............................................................caGgccauucuacaguguac..............................	1	1	Mm3
.............................................................caagAcauucuacaguguac..............................	1	1	Mm3
.............................................................caagccauucuacagugGac..............................	5	1	Mm3
.............................................................caagccauucuacagugCac..............................	6	1	Mm3
.............................................................cCagccauucuacaguguac..............................	1	1	Mm3
.............................................................caagccauucuacaguCuac..............................	6	1	Mm3
.............................................................caagccauucAacaguguac..............................	2	1	Mm3
.............................................................caagccauucuGcaguguac..............................	1	1	Mm3
.............................................................caagccauucGacaguguac..............................	2	1	Mm3
.............................................................caagccauucuacaguguaA.............................	1	1	Mm3
```

Mature Star

```
aaauguggugaguuucugugguacccuguagaaccgagcuaguguaucgaguucgaaacucacaagccauucuacaguguacaaagaaacgucgcuucuuucaacaaugaga
.............................................................caagccauCcuacaguguac..............................	1	1	Mm3
.............................................................Uaagccauucuacaguguac..............................	4	1	Mm3
.............................................................caagccauucuacagugAac..............................	2	1	Mm3
.............................................................caagccauucuacaguguaca.............................	6411	0	Mm3
.............................................................cCagccauucuacaguguaca.............................	2	1	Mm3
.............................................................caagccauucuacaguguacU.............................	22	1	Mm3
.............................................................caagccauucuacaguguacG.............................	10	1	Mm3
.............................................................caagccauucuCcaguguaca.............................	1	1	Mm3
.............................................................cUagccauucuacaguguaca.............................	2	1	Mm3
.............................................................caaUccauucuacaguguaca.............................	1	1	Mm3
.............................................................caagccauucCacaguguaca.............................	1	1	Mm3
.............................................................caagccauucuacaguguaUa.............................	1	1	Mm3
.............................................................caagccauucuacUguguaca.............................	1	1	Mm3
.............................................................caagccauucuacaguAuaca.............................	18	1	Mm3
.............................................................caagccauucuacaguguaAa.............................	1	1	Mm3
.............................................................caagccauucuacaguCuaca.............................	16	1	Mm3
.............................................................caagccauucuacGguguaca.............................	1	1	Mm3
.............................................................caagccUuucuacaguguaca.............................	1	1	Mm3
.............................................................caagccauucuacagugCaca.............................	34	1	Mm3
.............................................................caagccauucuacaguguacC.............................	2	1	Mm3
.............................................................caagccauucGacaguguaca.............................	6	1	Mm3
.............................................................caagAcauucuacaguguaca.............................	1	1	Mm3
.............................................................caagccGuucuacaguguaca.............................	1	1	Mm3
.............................................................Naagccauucuacaguguaca.............................	4	1	Mm3
.............................................................caUgccauucuacaguguaca.............................	1	1	Mm3
.............................................................caagUcauucuacaguguaca.............................	3	1	Mm3
.............................................................caagccauucuacaguUuaca.............................	2	1	Mm3
.............................................................caCgccauucuacaguguaca.............................	2	1	Mm3
.............................................................caagccauuUuacaguguaca.............................	4	1	Mm3
.............................................................cGagccauucuacaguguaca.............................	2	1	Mm3
.............................................................caagccauucuacagAguaca.............................	1	1	Mm3
.............................................................caagccauucuGcaguguaca.............................	1	1	Mm3
.............................................................caagcNauucuacaguguaca.............................	1	1	Mm3
.............................................................caagccauucuaUaguguaca.............................	1	1	Mm3
.............................................................caagcGauucuacaguguaca.............................	1	1	Mm3
.............................................................caagccauucAacaguguaca.............................	8	1	Mm3
.............................................................Uaagccauucuacaguguaca.............................	10	1	Mm3
.............................................................caagccauCcuacaguguaca.............................	3	1	Mm3
.............................................................caagccauucuacagugAaca.............................	23	1	Mm3
.............................................................caagcUauucuacaguguaca.............................	2	1	Mm3
.............................................................caagccauucuacagugGaca.............................	23	1	Mm3
.............................................................caaAccauucuacaguguaca.............................	2	1	Mm3
.............................................................caGgccauucuacaguguaca.............................	3	1	Mm3
.............................................................caagccauucuacaguUuacaa............................	4	1	Mm3
.............................................................caagccauucGacaguguacaa............................	1	1	Mm3
.............................................................caagccauucuacaguguacaU............................	17	1	Mm3
.............................................................Uaagccauucuacaguguacaa............................	3	1	Mm3
.............................................................caagccauucuacaUuguacaa............................	2	1	Mm3
.............................................................caagccauucuacagugCacaa............................	18	1	Mm3
.............................................................caagUcauucuacaguguacaa............................	1	1	Mm3
.............................................................caagccauuGuacaguguacaa............................	1	1	Mm3
.............................................................caagccauucCacaguguacaa............................	2	1	Mm3
.............................................................caagccauucuacaguguaUaa............................	3	1	Mm3
.............................................................caCgccauucuacaguguacaa............................	1	1	Mm3
.............................................................caagccauucuacagugAacaa............................	8	1	Mm3
.............................................................caagccauucuacaguguacaG............................	1	1	Mm3
.............................................................caagccauucuacaguCuacaa............................	8	1	Mm3
.............................................................caagccauucuacaguguacaC............................	114	1	Mm3
.............................................................caagccauucuacaguguacaa............................	2191	0	Mm3
.............................................................caagccauucuUcaguguacaa............................	1	1	Mm3
.............................................................caagccauucuaUaguguacaa............................	1	1	Mm3
.............................................................caagccauucuacagugGacaa............................	10	1	Mm3
.............................................................caagccauucuacaguAuacaa............................	4	1	Mm3
.............................................................caagccauucAacaguguacaa............................	3	1	Mm3
.............................................................caagccauucuacagCguacaa............................	1	1	Mm3
.............................................................caaAccauucuacaguguacaa............................	1	1	Mm3
.............................................................Naagccauucuacaguguacaa............................	1	1	Mm3
.............................................................caagccauucuacagAguacaa............................	1	1	Mm3
.............................................................caagccauucAacaguguacaaa...........................	1	1	Mm3
.............................................................caagccauucuacaguguacaaa...........................	114	0	Mm3
```

```
                     Mature                                 Star
aaauguggugagauuucugugtacccuguagaaccgagcuaguguaucgaguucgaaacucacaagccauucuacaguguacaaagaaacgucgcuucuuucaacaaugaga
..............................................................caagccauucuacaguAuacaaa...........................   1     1   Mm3
..............................................................caagccauucuacaguguacaaU...........................   109   1   Mm3
..............................................................caagccauucuacaguguacaaC...........................   20    1   Mm3
..............................................................caagccauucuacaguCuacaaa...........................   1     1   Mm3
..............................................................caagccauucuacaguguacaaG...........................   4     1   Mm3
..............................................................caagccauucuacagugGacaaa...........................   2     1   Mm3
..............................................................caagccauucuacaguguacaaaA..........................   2     1   Mm3
..............................................................caagccauucuacaguguacaaaU..........................   6     1   Mm3
...............................................................aagccauucuacaguguac..............................   5     0   Mm3
...............................................................aagccauucuacagugGaca.............................   1     1   Mm3
...............................................................aagccauucuacaguguaca.............................   32    0   Mm3
...............................................................aagccauuUuacaguguacaa............................   1     1   Mm3
...............................................................Cagccauucuacaguguacaa............................   1     1   Mm3
...............................................................aagccauucuacaguguacCa............................   1     1   Mm3
...............................................................aagccauucuacaguguacaa............................   5     0   Mm3
...............................................................aagccauucuacaguguacaaa...........................   1     0   Mm3
................................................................agccauucuacaguguacaa............................   1     0   Mm3
..................................................................ccauucuacaguguacaaaAa.........................   2     1   Mm3

aaauguggugagauuucugug...........................................................................................   1     0   Fm3
................cuguguacccuguagaaccga...........................................................................   1     0   Fm3
................cuguguacccuguagaaccgagc.........................................................................   4     0   Fm3
................cuguguacccuguagaaccgagcu........................................................................   14    0   Fm3
.................uguguacccuguagaacGga...........................................................................   1     1   Fm3
.................uguguacccuguagaaccga...........................................................................   2     0   Fm3
.................uguguacccuguagaaccgag..........................................................................   1     0   Fm3
.................uguguacccuguaAaaccgagc.........................................................................   1     1   Fm3
.................uguguacccuguagaaccgagc.........................................................................   52    0   Fm3
.................uguguacccuguagaacUgagc.........................................................................   1     1   Fm3
.................uguguacccuguagaacUgagcu........................................................................   1     1   Fm3
.................uguguacccuguagaaccgagcu........................................................................   151   0   Fm3
.................uguguacccuguagaacAgagcu........................................................................   1     1   Fm3
.................uguguacccuguagaaccgagcuU.......................................................................   1     1   Fm3
.................uguguacccuguagaaccgagcuag......................................................................   1     0   Fm3
..................guguacccuguagaaccgagc.........................................................................   1     0   Fm3
..................Cuguacccuguagaaccgagc.........................................................................   2     1   Fm3
..................guguacccuguagaaccgagcu........................................................................   6     0   Fm3
..................Cuguacccuguagaaccgagcuag......................................................................   2     1   Fm3
..................guguacccuguagaaccgagcuaU......................................................................   1     1   Fm3
..................guguacccuguagaaccgagcuagu.....................................................................   5     0   Fm3
..................Cuguacccuguagaaccgagcuagu.....................................................................   2     1   Fm3
..................guguacccuguagaaccgagcuagugu...................................................................   1     0   Fm3
...................uguacUcuguagaaccga...........................................................................   1     1   Fm3
...................uguacccuguagaaccgG...........................................................................   1     1   Fm3
...................uguacccuguagaaccga...........................................................................   50    0   Fm3
...................uguaUccuguagaaccga...........................................................................   1     1   Fm3
...................ugAacccuguagaaccga...........................................................................   1     1   Fm3
...................uguacccuguagaaccgU...........................................................................   1     1   Fm3
...................uguacccuguagaaccgaA..........................................................................   1     1   Fm3
...................uguacccuguagaaccgag..........................................................................   25    0   Fm3
...................uguacccuguagaaccCagc.........................................................................   1     1   Fm3
...................uguacccuguagaaccUagc.........................................................................   1     1   Fm3
...................uguacccugCagaaccgagc.........................................................................   1     1   Fm3
...................uguacccuguagaaccgGgc.........................................................................   1     1   Fm3
...................uguacccuguUgaaccgagc.........................................................................   1     1   Fm3
...................uguacccuguagaaccgCgc.........................................................................   3     1   Fm3
...................uguacccuguagaaccgagU.........................................................................   4     1   Fm3
...................uguacccuguagaGccgagc.........................................................................   1     1   Fm3
...................uguacccuguagaaccgagc.........................................................................   243   0   Fm3
...................uAuacccuguagaaccgagcu........................................................................   1     1   Fm3
...................uguacccuguagaaccgGgcu........................................................................   2     1   Fm3
...................uguacccuguagaaUcgagcu........................................................................   3     1   Fm3
...................uguacccuguagaaccgUgcu........................................................................   2     1   Fm3
...................uguaAccuguagaaccgagcu........................................................................   1     1   Fm3
...................uguacccuguagGaccgagcu........................................................................   2     1   Fm3
...................uguacccuguUgaaccgagcu........................................................................   1     1   Fm3
...................ugCacccuguagaaccgagcu........................................................................   1     1   Fm3
...................uguacccuguCgaaccgagcu........................................................................   1     1   Fm3
...................uguacccuguagaaccUagcu........................................................................   1     1   Fm3
...................uUuacccuguagaaccgagcu........................................................................   1     1   Fm3
```

```
                    Mature                               Star
aaauguggugaguuucugugUACCCUGUAGAACCGAGCUAGU
```

```
Mature: uacccuguagaaccgagcuagu    Star: caagccauucuacaguguaca
aaauguggugaguuucuguguacccuguagaaccgagcuaguguaucgaguucgaaacucacaagccauucuacaguguacaaagaaacgucgcuucuuucaacaaugaga
..................uguUcccuguagaaccgagcu........................................................................ 1 1 Fm3
..................uguaGccuguagaaccgagcu........................................................................ 1 1 Fm3
..................uguacccuUuagaaccgagcu........................................................................ 1 1 Fm3
..................uguacUcuguagaaccgagcu........................................................................ 3 1 Fm3
..................uguacccuguagaaccgagcu........................................................................ 846 0 Fm3
..................uguacccuguagaacUgagcu........................................................................ 1 1 Fm3
..................uguacccuguagaaccAagcu........................................................................ 2 1 Fm3
..................uguacccuguagaaccCagcu........................................................................ 2 1 Fm3
..................uguacccuguagaaccgCgcu........................................................................ 5 1 Fm3
..................uguacccuguUgaaccgagcua....................................................................... 1 1 Fm3
..................uguacccuguagaaccgagUua....................................................................... 1 1 Fm3
..................uguacccuguaUaaccgagcua....................................................................... 1 1 Fm3
..................uguacccuguagaaccgagcAa....................................................................... 2 1 Fm3
..................uguacccuguagaaccCagcua....................................................................... 2 1 Fm3
..................uguacccuguagaaccgagcua....................................................................... 498 0 Fm3
..................uguacccuguagaaccgCgcua....................................................................... 3 1 Fm3
..................uguacccuguagaaccAagcua....................................................................... 1 1 Fm3
..................uguacccuguagGaccgagcua....................................................................... 1 1 Fm3
..................uguacccuguagaaccgagcuU....................................................................... 9 1 Fm3
..................uguacccuguagaaccgGgcua....................................................................... 1 1 Fm3
..................uguacccuguagaaccgUgcua....................................................................... 2 1 Fm3
..................uguaccUuguagaaccgagcuag...................................................................... 2 1 Fm3
..................uguacccuguagaaccgGgcuag...................................................................... 23 1 Fm3
..................uguacccugAagaaccgagcuag...................................................................... 1 1 Fm3
..................uCuacccuguagaaccgagcuag...................................................................... 2 1 Fm3
..................uguacccuguagaaccgagcuaA...................................................................... 57 1 Fm3
..................uguacccuguagaaccgagcuGg...................................................................... 6 1 Fm3
..................uguacccuguagUaccgagcuag...................................................................... 2 1 Fm3
..................uguacccuguagaUccgagcuag...................................................................... 2 1 Fm3
..................Aguacccuguagaaccgagcuag...................................................................... 2 1 Fm3
..................uguacccuguagaaccgagcuaU...................................................................... 319 1 Fm3
..................uguacccuguagaaccUagcuag...................................................................... 16 1 Fm3
..................ugCacccuguagaaccgagcuag...................................................................... 2 1 Fm3
..................uguacccAguagaaccgagcuag...................................................................... 1 1 Fm3
..................uAuacccuguagaaccgagcuag...................................................................... 5 1 Fm3
..................uguacccuguagaaccAagcuag...................................................................... 37 1 Fm3
..................uguaAccuguagaaccgagcuag...................................................................... 2 1 Fm3
..................uguaccAuguagaaccgagcuag...................................................................... 8 1 Fm3
..................uUuacccuguagaaccgagcuag...................................................................... 5 1 Fm3
..................uguacccuguagaaccCagcuag...................................................................... 20 1 Fm3
..................uguacccCguagaaccgagcuag...................................................................... 1 1 Fm3
..................uguacNcuguagaaccgagcuag...................................................................... 3 1 Fm3
..................uguacccuguaUaaccgagcuag...................................................................... 2 1 Fm3
..................uguacccugCagaaccgagcuag...................................................................... 2 1 Fm3
..................uguacccuguagaGccgagcuag...................................................................... 4 1 Fm3
..................uguacccuCuagaaccgagcuag...................................................................... 2 1 Fm3
..................uguacccuguagaaccgaAcuag...................................................................... 1 1 Fm3
..................uguacccuguagaaccgagAuag...................................................................... 1 1 Fm3
..................uguacccuguagaaUcgagcuag...................................................................... 4 1 Fm3
..................uguacccuguGgaaccgagcuag...................................................................... 12 1 Fm3
..................uguacccuguCgaaccgagcuag...................................................................... 10 1 Fm3
..................uguacccuguagaaccgCgcuag...................................................................... 48 1 Fm3
..................uguacccuUuagaaccgagcuag...................................................................... 2 1 Fm3
..................uguacccuguagaaccgagUuag...................................................................... 47 1 Fm3
..................ugGacccuguagaaccgagcuag...................................................................... 2 1 Fm3
..................uguacGcuguagaaccgagcuag...................................................................... 1 1 Fm3
..................uguacccGguagaaccgagcuag...................................................................... 1 1 Fm3
..................ugAacccuguagaaccgagcuag...................................................................... 3 1 Fm3
..................uguacccuguagaaccgUgcuag...................................................................... 38 1 Fm3
..................uguacccuguagaaccgagcuUg...................................................................... 1 1 Fm3
..................uguaccGuguagaaccgagcuag...................................................................... 1 1 Fm3
..................uguacAcuguagaaccgagcuag...................................................................... 4 1 Fm3
..................uguacUcuguagaaccgagcuag...................................................................... 9 1 Fm3
..................uguacccuguUgaaccgagcuag...................................................................... 8 1 Fm3
..................Nguacccuguagaaccgagcuag...................................................................... 4 1 Fm3
..................uguacccuAuagaaccgagcuag...................................................................... 1 1 Fm3
..................uguaUccuguagaaccgagcuag...................................................................... 4 1 Fm3
..................uguacccuguagaacUgagcuag...................................................................... 3 1 Fm3
..................uguacccuguagaacAgagcuag...................................................................... 1 1 Fm3
..................uguacccuguagaaccgagcuag...................................................................... 10052 0 Fm3
```

Mature **Star**

```
aaauguggugaguuucugug uacccuguagaaccgagcuagu guaucgaguucgaaacuca caagccauucuacaguguaca aagaaacgucgcuucuuucaacaaugaga
..................uguacccuguagaaccgagcGag......................................................................    5      1    Fm3
..................uguCcccuguagaaccgagcuag......................................................................    1      1    Fm3
..................uguacccuguaAaaccgagcuag......................................................................    1      1    Fm3
..................uguacccuAuagaaccgagcuagu.....................................................................    12     1    Fm3
..................uguacccuguagaaccgagcuaAu.....................................................................    11     1    Fm3
..................uguacccuguagaaccgagcuagu.....................................................................    52461  0    Fm3
..................uguacccuguagaaccgagcuUgu.....................................................................    6      1    Fm3
..................uCuacccuguagaaccgagcuagu.....................................................................    25     1    Fm3
..................uguaccAuguagaaccgagcuagu.....................................................................    22     1    Fm3
..................uguacccuguagCaccgagcuagu.....................................................................    3      1    Fm3
..................uguacccuguaAaaccgagcuagu.....................................................................    1      1    Fm3
..................uguCcccuguagaaccgagcuagu.....................................................................    3      1    Fm3
..................uguaccUuguagaaccgagcuagu.....................................................................    11     1    Fm3
..................uguacccuguagaaccgagGuagu.....................................................................    3      1    Fm3
..................uguaccGuguagaaccgagcuagu.....................................................................    3      1    Fm3
..................uguaUccuguagaaccgagcuagu.....................................................................    11     1    Fm3
..................uguacccuguagaaGcgagcuagu.....................................................................    3      1    Fm3
..................uAuacccuguagaaccgagcuagu.....................................................................    33     1    Fm3
..................uguacccuguaUaaccgagcuagu.....................................................................    14     1    Fm3
..................uguacccuguagaaccgagcuagC.....................................................................    8      1    Fm3
..................ugGacccuguagaaccgagcuagu.....................................................................    5      1    Fm3
..................uguacccuguagaaccgagcAagu.....................................................................    3      1    Fm3
..................uguacccuguagaaccgagcCagu.....................................................................    9      1    Fm3
..................uguacccuguagaaccgagcuagA.....................................................................    321    1    Fm3
..................uguacccuguagaaccgCgcuagu.....................................................................    209    1    Fm3
..................uguacccuguagaaccgaCcuagu.....................................................................    7      1    Fm3
..................uguacccuguagUaccgagcuagu.....................................................................    9      1    Fm3
..................uguacccuguagaaccgagcuagG.....................................................................    5      1    Fm3
..................uguacccugGagaaccgagcuagu.....................................................................    1      1    Fm3
..................uguacccuguagaaccUagcuagu.....................................................................    101    1    Fm3
..................uNuacccuguagaaccgagcuagu.....................................................................    1      1    Fm3
..................uguacccuguagGaccgagcuagu.....................................................................    3      1    Fm3
..................uguacGcuguagaaccgagcuagu.....................................................................    4      1    Fm3
..................uguacccuguagaacUgagcuagu.....................................................................    23     1    Fm3
..................uguacccuguagaaccgGgcuagu.....................................................................    130    1    Fm3
..................uguacccuguGgaaccgagcuagu.....................................................................    66     1    Fm3
..................uguacccuguagaaccCagcuagu.....................................................................    100    1    Fm3
..................uguacccGuagaaccgagcuagu......................................................................    2      1    Fm3
..................uguacccuguagaaccgagcuCgu.....................................................................    1      1    Fm3
..................uguacccuguCgaaccgagcuagu.....................................................................    49     1    Fm3
..................ugCacccuguagaaccgagcuagu.....................................................................    11     1    Fm3
..................uguacccugAagaaccgagcuagu.....................................................................    7      1    Fm3
..................uguUcccuguagaaccgagcuagu.....................................................................    4      1    Fm3
..................uguacccuguagaaccgagcuaUu.....................................................................    38     1    Fm3
..................uUuacccuguagaaccgagcuagu.....................................................................    9      1    Fm3
..................uguacccuguagaUccgagcuagu.....................................................................    13     1    Fm3
..................uguacccuguaCaaccgagcuagu.....................................................................    3      1    Fm3
..................uguacccAguagaaccgagcuagu.....................................................................    7      1    Fm3
..................uguacccuguagaacAgagcuagu.....................................................................    11     1    Fm3
..................uguacccugCagaaccgagcuagu.....................................................................    9      1    Fm3
..................uguGcccuguagaaccgagcuagu.....................................................................    5      1    Fm3
..................uguaccNuguagaaccgagcuagu.....................................................................    1      1    Fm3
..................uguacccuguagaaccgagAuagu.....................................................................    6      1    Fm3
..................uguacNcuguagaaccgagcuagu.....................................................................    8      1    Fm3
..................uguacccuUuagaaccgagcuagu.....................................................................    7      1    Fm3
..................uguacccuguagaaccgagcuGgu.....................................................................    34     1    Fm3
..................uguaGccuguagaaccgagcuagu.....................................................................    7      1    Fm3
..................uguacAcuguagaaccgagcuagu.....................................................................    23     1    Fm3
..................Aguacccuguagaaccgagcuagu.....................................................................    10     1    Fm3
..................uguacccuguagaaAcgagcuagu.....................................................................    5      1    Fm3
..................Nguacccuguagaaccgagcuagu.....................................................................    29     1    Fm3
..................Cguacccuguagaaccgagcuagu.....................................................................    16     1    Fm3
..................uguacccuguagaaccgaUcuagu.....................................................................    13     1    Fm3
..................uguacccCguagaaccgagcuagu.....................................................................    7      1    Fm3
..................uguacccuguagaGccgagcuagu.....................................................................    8      1    Fm3
..................uguacccuguagaaUcgagcuagu.....................................................................    21     1    Fm3
..................uguacUcuguagaaccgagcuagu.....................................................................    82     1    Fm3
..................uguacccuguagaaccgagcuagN.....................................................................    1      1    Fm3
..................uguacccuCuagaaccgagcuagu.....................................................................    9      1    Fm3
..................uguacccuguagaCccgagcuagu.....................................................................    3      1    Fm3
```

Mature **Star**

```
aaauguggugaguuucuguguacccuguagaaccgagcuaguguaucgaguucgaaacucacaagccauucuacaguguacaaagaaacgucgcuucuuucaacaaugaga
..................uguacccuguagaaccgagUuagu.....................................................................   66     1   Fm3
..................uguacccuguagaaccgagcuaCu.....................................................................   1      1   Fm3
..................uguacccuguagaaccgagcGagu.....................................................................   72     1   Fm3
..................ugAacccuguagaaccgagcuagu.....................................................................   14     1   Fm3
..................uguacccuguUgaaccgagcuagu.....................................................................   43     1   Fm3
..................uguaAccuguagaaccgagcuagu.....................................................................   7      1   Fm3
..................uguacccuguagaaccgUgcuagu.....................................................................   176    1   Fm3
..................uguacccuguagaaccgaAcuagu.....................................................................   5      1   Fm3
..................uguacccuguagaaccAagcuagu.....................................................................   137    1   Fm3
..................uguacccuguagaaccAagcuagug....................................................................   2      1   Fm3
..................Nguacccuguagaaccgagcuagug....................................................................   3      1   Fm3
..................uguaGccuguagaaccgagcuagug....................................................................   1      1   Fm3
..................uguacccuguagaaccgagcuagug....................................................................   1757   0   Fm3
..................uAuacccuguagaaccgagcuagug....................................................................   2      1   Fm3
..................uguacccuguGgaaccgagcuagug....................................................................   1      1   Fm3
..................uguaccAuguagaaccgagcuagug....................................................................   1      1   Fm3
..................uguacccuguagaaccgagcGagug....................................................................   2      1   Fm3
..................uguacccuguCgaaccgagcuagug....................................................................   1      1   Fm3
..................uguacccuguUgaaccgagcuagug....................................................................   1      1   Fm3
..................uguacccuguagaaccgUgcuagug....................................................................   3      1   Fm3
..................uguacccuguagaaccgagcuaguC....................................................................   2      1   Fm3
..................uguacccuguagaaccCagcuagug....................................................................   4      1   Fm3
..................uguacccuguagCaccgagcuagug....................................................................   1      1   Fm3
..................uguacccuUuagaaccgagcuagug....................................................................   1      1   Fm3
..................uguacUcuguagaaccgagcuagug....................................................................   2      1   Fm3
..................uguacccuguagaaccgGgcuagug....................................................................   1      1   Fm3
..................uUuacccuguagaaccgagcuagug....................................................................   1      1   Fm3
..................uguacccuguagaGccgagcuagug....................................................................   1      1   Fm3
..................uguacccuguagaaccgagcuGgug....................................................................   2      1   Fm3
..................uguacccuguagaaccgagcuaguU....................................................................   1863   1   Fm3
..................uguacccuguagaaccgagcuaguA....................................................................   78     1   Fm3
..................uguacccuguagaaccUagcuagug....................................................................   5      1   Fm3
..................uguacccuguagaaccgCgcuagug....................................................................   10     1   Fm3
..................uguacccuguagaaccgagcuagCgu...................................................................   1      1   Fm3
..................uguacccuguagaaccgagcuagugC...................................................................   9      1   Fm3
..................uguacccuguagaacUgagcuagugu...................................................................   1      1   Fm3
..................uguacccuAuagaaccgagcuagugu...................................................................   1      1   Fm3
..................uguaccNuguagaaccgagcuagugu...................................................................   1      1   Fm3
..................uguacccuguagaaccgCgcuagugu...................................................................   5      1   Fm3
..................uguaUccuguagaaccgagcuagugu...................................................................   1      1   Fm3
..................uguacccuguagaaccCagcuagugu...................................................................   4      1   Fm3
..................uguacccuguagaaccgagcCagugu...................................................................   1      1   Fm3
..................uguacccuguagaaccUagcuagugu...................................................................   1      1   Fm3
..................uguacccuguUgaaccgagcuagugu...................................................................   1      1   Fm3
..................uCuacccuguagaaccgagcuagugu...................................................................   2      1   Fm3
..................Cguacccuguagaaccgagcuagugu...................................................................   1      1   Fm3
..................Nguacccuguagaaccgagcuagugu...................................................................   1      1   Fm3
..................uguacccuguagaaccgagcuagugG...................................................................   2      1   Fm3
..................uguacccuguagaaccgagcuagAgu...................................................................   1      1   Fm3
..................uguacccuguagaaccgagcuagugA...................................................................   40     1   Fm3
..................uguacUcuguagaaccgagcuagugu...................................................................   2      1   Fm3
..................uguacccuguagCaccgagcuagugu...................................................................   1      1   Fm3
..................uguacccuguagaaccgGgcuagugu...................................................................   5      1   Fm3
..................uguacccuguagaaccAagcuagugu...................................................................   6      1   Fm3
..................uAuacccuguagaaccgagcuagugu...................................................................   1      1   Fm3
..................uguacccuguagaaccgagcuaguAu...................................................................   30     1   Fm3
..................uguacccuguagaaccgagcuaguCu...................................................................   2      1   Fm3
..................uguacccCguagaaccgagcuagugu...................................................................   1      1   Fm3
..................uguacccuguagaaccgagcuagugu...................................................................   1456   0   Fm3
..................ugCacccuguagaaccgagcuagugu...................................................................   2      1   Fm3
..................uguacccuguagaaccgUgcuagugu...................................................................   3      1   Fm3
..................uguacccuguagaaccgagcuaguUu...................................................................   48     1   Fm3
..................uguacccuguGgaaccgagcuagugu...................................................................   3      1   Fm3
..................uguaccUuguagaaccgagcuagugu...................................................................   1      1   Fm3
..................uguacccuguagaaccgagcuaguguU..................................................................   16     1   Fm3
..................uguacccuguagaaccgagcuaguCua..................................................................   1      1   Fm3
..................uguacccuguagaaccgagcuaguguau.................................................................   1      0   Fm3
...................Uuacccuguagaaccgag..........................................................................   1      1   Fm3
...................guacccuguagaaccgag..........................................................................   3      0   Fm3
...................guacccuguagaaccgagU.........................................................................   1      1   Fm3
```

```
                     Mature                  Star
aaauguggugaguuucuguguacccuguagaaccgagcuaguguaucgaguucgaaacucacaagccauucuacaguguacaaagaaacgucgcuucuuucaacaaugaga
...................Cuacccuguagaaccgagc.........................................................................    1     1    Fm3
...................Nuacccuguagaaccgagc.........................................................................    1     1    Fm3
...................Uuacccuguagaaccgagc.........................................................................    9     1    Fm3
...................guacccuguagaaccgagc.........................................................................    13    0    Fm3
...................guacccuguagaaccgagcA........................................................................    1     1    Fm3
...................Auacccuguagaaccgagcu........................................................................    259   1    Fm3
...................guacccuguagaaccgagcu........................................................................    116   0    Fm3
...................guacccuguagaaccgaCcu........................................................................    1     1    Fm3
...................Cuacccuguagaaccgagcu........................................................................    1     1    Fm3
...................guacccuguagaaccgaAcu........................................................................    1     1    Fm3
...................guacccuguagaaccgaUcu........................................................................    1     1    Fm3
...................guacccuguagaaccUagcu........................................................................    1     1    Fm3
...................guacccuguUgaaccgagcu........................................................................    1     1    Fm3
...................guacccuguagCaccgagcu........................................................................    1     1    Fm3
...................guacccuguagaaccgagcGa.......................................................................    15    1    Fm3
...................guacccuguagaaUcgagcua.......................................................................    1     1    Fm3
...................Auacccuguagaaccgagcua.......................................................................    13    1    Fm3
...................guaccAuguagaaccgagcua.......................................................................    1     1    Fm3
...................guacccuguagaaccgagcua.......................................................................    33    0    Fm3
...................Cuacccuguagaaccgagcua.......................................................................    1     1    Fm3
...................guacccuguagaaccgGgcuag......................................................................    1     1    Fm3
...................guacccuguagaaccgagcuag......................................................................    1130  0    Fm3
...................guacccuguagaaccgagAuag......................................................................    1     1    Fm3
...................Auacccuguagaaccgagcuag......................................................................    70    1    Fm3
...................guacccuguagaaccgagUuag......................................................................    1     1    Fm3
...................guacccuguagaaccgaAcuag......................................................................    7     1    Fm3
...................guacccuguagaaccgaUcuag......................................................................    2     1    Fm3
...................guacccuguagaaUcgagcuag......................................................................    5     1    Fm3
...................Cuacccuguagaaccgagcuag......................................................................    21    1    Fm3
...................guacccuguagaacGgagcuag......................................................................    1     1    Fm3
...................guacccuguagaaccgUgcuag......................................................................    3     1    Fm3
...................guacccuguagaaccgagcuaA......................................................................    1     1    Fm3
...................guacccuguagaaccgagcGag......................................................................    21    1    Fm3
...................guacUcuguagaaccgagcuag......................................................................    1     1    Fm3
...................Nuacccuguagaaccgagcuag......................................................................    1     1    Fm3
...................guacccuguaAaaccgagcuag......................................................................    1     1    Fm3
...................guacccuguagaaccgagcuaU......................................................................    19    1    Fm3
...................guaUccuguagaaccgagcuag......................................................................    1     1    Fm3
...................gAacccuguagaaccgagcuag......................................................................    1     1    Fm3
...................guaccAuguagaaccgagcuag......................................................................    1     1    Fm3
...................guacccuguagaaccgaCcuag......................................................................    3     1    Fm3
...................guacccuguagaaccgCgcuag......................................................................    2     1    Fm3
...................guacccugCagaaccgagcuag......................................................................    1     1    Fm3
...................guacccuguagaaccgagcuGgu.....................................................................    24    1    Fm3
...................guacccuguagaaccgagGuagu.....................................................................    1     1    Fm3
...................guacccuguagaaAcgagcuagu.....................................................................    6     1    Fm3
...................guaGccuguagaaccgagcuagu.....................................................................    1     1    Fm3
...................guacccuguagCaccgagcuagu.....................................................................    2     1    Fm3
...................guacccuguagaaccgUgcuagu.....................................................................    25    1    Fm3
...................guacccuguagaaccgagcuagC.....................................................................    1     1    Fm3
...................guacccuguagaaccgagcuagG.....................................................................    1     1    Fm3
...................guacccuguagaaccgaUcuagu.....................................................................    63    1    Fm3
...................guacccuguagaaccgagcCagu.....................................................................    2     1    Fm3
...................Nuacccuguagaaccgagcuagu.....................................................................    7     1    Fm3
...................guacccuguagaaccUagcuagu.....................................................................    5     1    Fm3
...................guacccNguagaaccgagcuagu.....................................................................    1     1    Fm3
...................guacUcuguagaaccgagcuagu.....................................................................    2     1    Fm3
...................guacccuguaCaaccgagcuagu.....................................................................    15    1    Fm3
...................Cuacccuguagaaccgagcuagu.....................................................................    327   1    Fm3
...................guacccuUuagaaccgagcuagu.....................................................................    1     1    Fm3
...................guCcccuguagaaccgagcuagu.....................................................................    1     1    Fm3
...................guacccuguagaaccAagcuagu.....................................................................    4     1    Fm3
...................guacccGguagaaccgagcuagu.....................................................................    1     1    Fm3
...................guacccuguagaCccgagcuagu.....................................................................    1     1    Fm3
...................guacccuguagaaccgagcuagA.....................................................................    69    1    Fm3
...................guacccuguagUaccgagcuagu.....................................................................    3     1    Fm3
...................Uuacccuguagaaccgagcuagu.....................................................................    20    1    Fm3
...................guacGcuguagaaccgagcuagu.....................................................................    4     1    Fm3
...................guacccuguagaacGgagcuagu.....................................................................    1     1    Fm3
...................gCacccuguagaaccgagcuagu.....................................................................    4     1    Fm3
```

```
                 Mature                    Star
aaauguggugaguuucugugguacccuguagaaccgagcuaguguaucgaguucgaaacucacaagccauucuacaguguacaaagaaacgucgcuucuuucaacaaugaga
```

```
...................guacccuguagaaUcgagcuagu.....................................................................   4      1   Fm3
...................guacccuguagaaccgagcuaUu.....................................................................   5      1   Fm3
...................guacccCguagaaccgagcuagu.....................................................................   3      1   Fm3
...................guacccuguagaaccgagcuaAu.....................................................................   1      1   Fm3
...................guacccuguagaaccgGgcuagu.....................................................................   6      1   Fm3
...................guacccuguagaacUgagcuagu.....................................................................   7      1   Fm3
...................guacccuguagaaccgaAcuagu.....................................................................   65     1   Fm3
...................guaccNuguagaaccgagcuagu.....................................................................   2      1   Fm3
...................guacccugCagaaccgagcuagu.....................................................................   1      1   Fm3
...................guacccuguaAaccgagcuagu......................................................................   20     1   Fm3
...................guacccuCuagaaccgagcuagu.....................................................................   4      1   Fm3
...................guacccuguaUaccgagcuagu......................................................................   17     1   Fm3
...................gGacccuguagaaccgagcuagu.....................................................................   1      1   Fm3
...................guacccuguUgaaccgagcuagu.....................................................................   2      1   Fm3
...................guacAcuguagaaccgagcuagu.....................................................................   6      1   Fm3
...................guaccUuguagaaccgagcuagu.....................................................................   3      1   Fm3
...................guacccuguagaacAgagcuagu.....................................................................   2      1   Fm3
...................Auacccuguagaaccgagcuagu.....................................................................   1790   1   Fm3
...................guacccuguagaGccgagcuagu.....................................................................   2      1   Fm3
...................guacccugAagaaccgagcuagu.....................................................................   1      1   Fm3
...................guacccuguagaaccgaCcuagu.....................................................................   56     1   Fm3
...................guaUccuguagaaccgagcuagu.....................................................................   3      1   Fm3
...................guacccuAuagaaccgagcuagu.....................................................................   3      1   Fm3
...................guaAccuguagaaccgagcuagu.....................................................................   1      1   Fm3
...................guacccuguagaaccgagUuagu.....................................................................   26     1   Fm3
...................guacccuguagGaccgagcuagu.....................................................................   2      1   Fm3
...................guacccuguGgaaccgagcuagu.....................................................................   3      1   Fm3
...................guacccuguagaaccgCgcuagu.....................................................................   38     1   Fm3
...................guacccuguagaaccgagAuagu.....................................................................   2      1   Fm3
...................gAacccuguagaaccgagcuagu.....................................................................   7      1   Fm3
...................guacccuguagaaccgagcGagu.....................................................................   229    1   Fm3
...................guGcccuguagaaccgagcuagu.....................................................................   5      1   Fm3
...................guacccuguagaaccgagcuagu.....................................................................   13251  0   Fm3
...................guaccAuguagaaccgagcuagu.....................................................................   5      1   Fm3
...................guacccuguagaaccgagcuGgug....................................................................   4      1   Fm3
...................gCacccuguagaaccgagcuagug....................................................................   1      1   Fm3
...................guacccuguagaaccgagcuaguU....................................................................   593    1   Fm3
...................guacccuguagaaccgUgcuagug....................................................................   5      1   Fm3
...................guacccuguagaaccgaAcuagug....................................................................   6      1   Fm3
...................guacccuguagaaccgagcuaguA....................................................................   41     1   Fm3
...................Nuacccuguagaaccgagcuagug....................................................................   1      1   Fm3
...................guacccuguaAaccgagcuagug.....................................................................   2      1   Fm3
...................guCccuguagaaccgagcuagug.....................................................................   1      1   Fm3
...................Auacccuguagaaccgagcuagug....................................................................   33     1   Fm3
...................guacccuguagaaUcgagcuagug....................................................................   1      1   Fm3
...................guacccuguagaaccgagcCagug....................................................................   1      1   Fm3
...................guaccAuguagaaccgagcuagug....................................................................   1      1   Fm3
...................guacccugCagaaccgagcuagug....................................................................   1      1   Fm3
...................guacccuguagaaccgCgcuagug....................................................................   2      1   Fm3
...................guacccuguagaaccgagcuagug....................................................................   1672   0   Fm3
...................guacccuguaCaccgagcuagug.....................................................................   2      1   Fm3
...................guacccuguagaUccgagcuagug....................................................................   1      1   Fm3
...................Uuacccuguagaaccgagcuagug....................................................................   2      1   Fm3
...................guacGcuguagaaccgagcuagug....................................................................   1      1   Fm3
...................guacccuguagaaccgagcGagug....................................................................   4      1   Fm3
...................Cuacccuguagaaccgagcuagug....................................................................   33     1   Fm3
...................guacccuguagGaccgagcuagug....................................................................   1      1   Fm3
...................guacccuguagaacUgagcuagug....................................................................   2      1   Fm3
...................guacccuguaUaccgagcuagug.....................................................................   1      1   Fm3
...................guacccuguagaaccgaCcuagug....................................................................   6      1   Fm3
...................guacccuguagaaccgaUcuagug....................................................................   8      1   Fm3
...................guacccuguagaacAgagcuagug....................................................................   1      1   Fm3
...................guacccuguagaaccgagUuagug....................................................................   7      1   Fm3
...................guaGccuguagaaccgagcuagug....................................................................   1      1   Fm3
...................guGcccuguagaaccgagcuagug....................................................................   2      1   Fm3
...................guacccuguagaaccAagcuagug....................................................................   1      1   Fm3
...................guacccuguagaacUgagcuagugu...................................................................   1      1   Fm3
...................guacccuguagaaccgaAcuagugu...................................................................   1      1   Fm3
...................guacccuguagaaccgUgcuagugu...................................................................   1      1   Fm3
...................Auacccuguagaaccgagcuagugu...................................................................   37     1   Fm3
```

Mature Star

```
aaauguggugaguuucugugguacccuguagaaccgagcuaguguaucgaguucgaaacucacaagccauucuacaguguacaaagaaacgucgcuucuuucaacaaugaga
```

```
aaauguggugaguuucuguguacccuguagaaccgagcuaguguaucgaguucgaaacucacaagccauucuacaguguacaaagaaacgucgcuucuuucaacaaugaga
...................guacccuguagaaccgagcuaguUu...................................................................  21    1  Fm3
...................guacccuguagaaccgagcuagugC...................................................................  2     1  Fm3
...................guacccuguaAaaccgagcuagugu...................................................................  2     1  Fm3
...................Uuacccuguagaaccgagcuagugu...................................................................  3     1  Fm3
...................guaUccuguagaaccgagcuagugu...................................................................  1     1  Fm3
...................guacccuguagaaccgagcuagCgu...................................................................  1     1  Fm3
...................gAacccuguagaaccgagcuagugu...................................................................  1     1  Fm3
...................guaGccuguagaaccgagcuagugu...................................................................  1     1  Fm3
...................Cuacccuguagaaccgagcuagugu...................................................................  24    1  Fm3
...................guacccuguagaaccgagcuagugu...................................................................  1100  0  Fm3
...................guacccuguagaaccgagcuGgugu...................................................................  3     1  Fm3
...................guacccuguaUaaccgagcuagugu...................................................................  1     1  Fm3
...................guacccuguagaaccgCgcuagugu...................................................................  4     1  Fm3
...................guacccuguagaaccgaUcuagugu...................................................................  3     1  Fm3
...................guacccuguagaaccgagcuaguAu...................................................................  6     1  Fm3
...................guacccuguagaGccgagcuagugu...................................................................  1     1  Fm3
...................guaccGuguagaaccgagcuagugu...................................................................  1     1  Fm3
...................guacccuguagaaccgagcuagugA...................................................................  74    1  Fm3
...................guacccuguagaaccgaCcuagugu...................................................................  5     1  Fm3
...................guacccuguagaaccgagcuaguguU..................................................................  26    1  Fm3
...................guacccuguagaaccgagcuagugua..................................................................  3     0  Fm3
...................guacccuguagaaccgagcuaguguUu.................................................................  2     1  Fm3
...................guacccuguagaaccgagcuaguguaucga..............................................................  1     0  Fm3
....................uacccuguagaaccgaUc.........................................................................  2     1  Fm3
....................uacccuguagaaccgagA.........................................................................  33    1  Fm3
....................uaUccuguagaaccgagc.........................................................................  1     1  Fm3
....................uacccuguagaaccgagG.........................................................................  3     1  Fm3
....................Aacccuguagaaccgagc.........................................................................  12    1  Fm3
....................uaccUuguagaaccgagc.........................................................................  1     1  Fm3
....................uacccuguagaacUgagc.........................................................................  7     1  Fm3
....................uacccuguagaaccgagc.........................................................................  971   0  Fm3
....................uacccuguagaaccgagU.........................................................................  25    1  Fm3
....................uacccuguagGaccgagc.........................................................................  1     1  Fm3
....................uacccuguagaaccgaCc.........................................................................  1     1  Fm3
....................uacccuguagUaccgagc.........................................................................  1     1  Fm3
....................uacUcuguagaaccgagc.........................................................................  1     1  Fm3
....................uGcccuguagaaccgagc.........................................................................  2     1  Fm3
....................uUcccuguagaaccgagc.........................................................................  1     1  Fm3
....................uacccuguagaaccgaAcu........................................................................  12    1  Fm3
....................uacccuguagaaccgagcC........................................................................  4     1  Fm3
....................uaUccuguagaaccgagcu........................................................................  1     1  Fm3
....................uacccuguagaacAgagcu........................................................................  3     1  Fm3
....................Aacccuguagaaccgagcu........................................................................  8     1  Fm3
....................uacAcuguagaaccgagcu........................................................................  2     1  Fm3
....................uacccugAagaaccgagcu........................................................................  1     1  Fm3
....................uacccuguagGaccgagcu........................................................................  3     1  Fm3
....................uacccuguagaaccgagcA........................................................................  41    1  Fm3
....................uacccuguGgaaccgagcu........................................................................  2     1  Fm3
....................uacccuguagaUccgagcu........................................................................  1     1  Fm3
....................uacccuguagaaGcgagcu........................................................................  1     1  Fm3
....................uacccuguagaaUcgagcu........................................................................  18    1  Fm3
....................uacccuguagaaccgGgcu........................................................................  1     1  Fm3
....................uacccuguagaaccgaCcu........................................................................  7     1  Fm3
....................Cacccuguagaaccgagcu........................................................................  1     1  Fm3
....................uacccuguagaacUgagcu........................................................................  3     1  Fm3
....................uacccuCuagaaccgagcu........................................................................  1     1  Fm3
....................uacccuguagaaccgaUcu........................................................................  14    1  Fm3
....................uacccuguagaaccgagAu........................................................................  64    1  Fm3
....................uacccuguagaaccAagcu........................................................................  1     1  Fm3
....................uaGccuguagaaccgagcu........................................................................  2     1  Fm3
....................uaccAuguagaaccgagcu........................................................................  5     1  Fm3
....................uacccuguagaaccgagGu........................................................................  19    1  Fm3
....................uGcccuguagaaccgagcu........................................................................  1     1  Fm3
....................uaAccuguagaaccgagcu........................................................................  1     1  Fm3
....................uacccuguaUaaccgagcu........................................................................  4     1  Fm3
....................uacccuguagCaccgagcu........................................................................  4     1  Fm3
....................uacccuguagUaccgagcu........................................................................  4     1  Fm3
....................uacccuguagaaccgagcu........................................................................  5211  0  Fm3
....................uacccuguagaaccgagUu........................................................................  19    1  Fm3
....................uacccuguaAaaccgagcu........................................................................  2     1  Fm3
```

Mature **Star**

```
aaauguggugaguuucugugtuacccuguagaaccgagcuaguguaucgaguucgaaacucacaagccauucuacaguguacaaagaaacgucgcuucuuucaacaaugaga
....................uacccAguagaaccgagcu........................................................................ 1 1 Fm3
....................uacccuguagaaccgagcuG....................................................................... 6 1 Fm3
....................uacccCguagaaccgagcua....................................................................... 1 1 Fm3
....................uacccugAagaaccgagcua....................................................................... 1 1 Fm3
....................uacccuguagaaccgagcuC....................................................................... 1 1 Fm3
....................uacUcuguagaaccgagcua....................................................................... 1 1 Fm3
....................uacccuguagaaccgagAua....................................................................... 24 1 Fm3
....................uacccuguagaaccgagcAa....................................................................... 22 1 Fm3
....................uacccuguagaUccgagcua....................................................................... 1 1 Fm3
....................uacccuUuagaaccgagcua....................................................................... 1 1 Fm3
....................uacccuguagaacUgagcua....................................................................... 3 1 Fm3
....................uacccuguagaaccgaCcua....................................................................... 3 1 Fm3
....................uaccAuguagaaccgagcua....................................................................... 1 1 Fm3
....................Nacccuguagaaccgagcua....................................................................... 1 1 Fm3
....................uacAcuguagaaccgagcua....................................................................... 1 1 Fm3
....................uacccuguagaaccgagUua....................................................................... 11 1 Fm3
....................uacccuguagaaccgagGua....................................................................... 7 1 Fm3
....................uacccuguagUaccgagcua....................................................................... 3 1 Fm3
....................uGcccuguagaaccgagcua....................................................................... 1 1 Fm3
....................uaUccuguagaaccgagcua....................................................................... 1 1 Fm3
....................uacccuguagaaccgagcuU....................................................................... 77 1 Fm3
....................uacccuguagaaccgaUcua....................................................................... 3 1 Fm3
....................uacccuguagaacGgagcua....................................................................... 1 1 Fm3
....................uacccuguagaaccgagcua....................................................................... 2156 0 Fm3
....................uacccuguagaaccCagcua....................................................................... 1 1 Fm3
....................uacccuguagGaccgagcua....................................................................... 2 1 Fm3
....................uacccNguagaaccgagcua....................................................................... 1 1 Fm3
....................uacccuCuagaaccgagcua....................................................................... 1 1 Fm3
....................uacccuguagaaccgaAcua....................................................................... 5 1 Fm3
....................uacccuguagaaccgUgcua....................................................................... 1 1 Fm3
....................uacccuguagaaccgCgcua....................................................................... 1 1 Fm3
....................uacccuguagaaUcgagcua....................................................................... 18 1 Fm3
....................uUcccuguagaaccgagcua....................................................................... 2 1 Fm3
....................Gacccuguagaaccgagcuag...................................................................... 1 1 Fm3
....................uacccuguCgaaccgagcuag...................................................................... 1 1 Fm3
....................uacccuguagaaccgaAcuag...................................................................... 133 1 Fm3
....................Nacccuguagaaccgagcuag...................................................................... 33 1 Fm3
....................uacccuguagaaccCagcuag...................................................................... 2 1 Fm3
....................uacccuguagCaccgagcuag...................................................................... 62 1 Fm3
....................Cacccuguagaaccgagcuag...................................................................... 17 1 Fm3
....................uacccuguagaaccgagAuag...................................................................... 657 1 Fm3
....................uacccuguaUaaccgagcuag...................................................................... 11 1 Fm3
....................Aacccuguagaaccgagcuag...................................................................... 7 1 Fm3
....................uacccuguaAaaccgagcuag...................................................................... 5 1 Fm3
....................uGcccuguagaaccgagcuag...................................................................... 10 1 Fm3
....................uacGcuguagaaccgagcuag...................................................................... 10 1 Fm3
....................uaGccuguagaaccgagcuag...................................................................... 14 1 Fm3
....................uacccuguagaaccgagcuUg...................................................................... 16 1 Fm3
....................uacccuguagaaAcgagcuag...................................................................... 2 1 Fm3
....................uacccuguagaaccgUgcuag...................................................................... 12 1 Fm3
....................uaAccuguagaaccgagcuag...................................................................... 18 1 Fm3
....................uacccuCuagaaccgagcuag...................................................................... 12 1 Fm3
....................uaccGuguagaaccgagcuag...................................................................... 4 1 Fm3
....................uacccuguagaaccgaCcuag...................................................................... 150 1 Fm3
....................uacccuAuagaaccgagcuag...................................................................... 14 1 Fm3
....................uacccuguagaaccgagcuCg...................................................................... 2 1 Fm3
....................uacccuguagaaccgCgcuag...................................................................... 2 1 Fm3
....................uacccuguagUaccgagcuag...................................................................... 76 1 Fm3
....................uacccuguagaaUcgagcuag...................................................................... 79 1 Fm3
....................uacccuguagaUccgagcuag...................................................................... 18 1 Fm3
....................uacccuguagaGccgagcuag...................................................................... 8 1 Fm3
....................uacUcuguagaaccgagcuag...................................................................... 16 1 Fm3
....................uacccuguagaaccgagcCag...................................................................... 12 1 Fm3
....................uaccUuguagaaccgagcuag...................................................................... 9 1 Fm3
....................uacccuguagaacAgagcuag...................................................................... 39 1 Fm3
....................uacccuguagaaccgGgcuag...................................................................... 10 1 Fm3
....................uacccNguagaaccgagcuag...................................................................... 9 1 Fm3
....................uacccuguagaacGgagcuag...................................................................... 5 1 Fm3
....................uacccuguUgaaccgagcuag...................................................................... 10 1 Fm3
....................uacccuguagaaccgagcuaU...................................................................... 1363 1 Fm3
```

Mature **Star**

```
aaauguggugaguuucugugtuacccuguagaaccgagcuaguguaucgaguucgaaacucacaagccauucuacaguguacaaagaaacgucgcuucuuucaacaaugaga
```

```
....................uacccuguagaaccgagcuag......................................................................  66172  0  Fm3
....................uacccAguagaaccgagcuag......................................................................  3  1  Fm3
....................uacccuguagaaGcgagcuag......................................................................  3  1  Fm3
....................uaccAuguagaaccgagcuag......................................................................  40  1  Fm3
....................uacccugCagaaccgagcuag......................................................................  7  1  Fm3
....................uCcccuguagaaccgagcuag......................................................................  1  1  Fm3
....................uacccuguagaaccgagcuaA......................................................................  209  1  Fm3
....................uacccGguagaaccgagcuag......................................................................  7  1  Fm3
....................uUcccuguagaaccgagcuag......................................................................  10  1  Fm3
....................uacccuguagaacUgagcuag......................................................................  34  1  Fm3
....................uacccuguagaaccAagcuag......................................................................  11  1  Fm3
....................uacccuguagGaccgagcuag......................................................................  65  1  Fm3
....................uacccuguGgaaccgagcuag......................................................................  6  1  Fm3
....................uacccuguagaaccgagcuaC......................................................................  1  1  Fm3
....................uacccuguagaaccgagGuag......................................................................  248  1  Fm3
....................uacccuguagaaccUagcuag......................................................................  13  1  Fm3
....................uacccuguaCaaccgagcuag......................................................................  2  1  Fm3
....................uacccuguagaaccgagUuag......................................................................  345  1  Fm3
....................uacccuUuagaaccgagcuag......................................................................  8  1  Fm3
....................uacccCguagaaccgagcuag......................................................................  5  1  Fm3
....................uacccuguagaaccgagcuGg......................................................................  60  1  Fm3
....................uaUccuguagaaccgagcuag......................................................................  11  1  Fm3
....................uacccuguagaaccgaUcuag......................................................................  117  1  Fm3
....................uacAcuguagaaccgagcuag......................................................................  45  1  Fm3
....................uacccugAagaaccgagcuag......................................................................  4  1  Fm3
....................uacccuNuagaaccgagcuag......................................................................  3  1  Fm3
....................uacccugGagaaccgagcuag......................................................................  1  1  Fm3
....................uacccuguagaaccgagcAag......................................................................  2  1  Fm3
....................uacccuguagaaccgUgcuagu.....................................................................  329  1  Fm3
....................uaNccuguagaaccgagcuagu.....................................................................  4  1  Fm3
....................uaccUuguagaaccgagcuagu.....................................................................  191  1  Fm3
....................uacccuguaUaaccgagcuagu.....................................................................  251  1  Fm3
....................uNcccuguagaaccgagcuagu.....................................................................  7  1  Fm3
....................uacccuguagaaccgagcuGgu.....................................................................  1563  1  Fm3
....................uacccuguagaaccUagcuagu.....................................................................  271  1  Fm3
....................uacccuguaCaaccgagcuagu.....................................................................  57  1  Fm3
....................uacccuCuagaaccgagcuagu.....................................................................  162  1  Fm3
....................uacccuguagGaccgagcuagu.....................................................................  1445  1  Fm3
....................uacccuguCgaaccgagcuagu.....................................................................  16  1  Fm3
....................uacccuguaAaaccgagcuagu.....................................................................  110  1  Fm3
....................uacccuguagaaGcgagcuagu.....................................................................  55  1  Fm3
....................uGcccuguagaaccgagcuagu.....................................................................  338  1  Fm3
....................uacccuguagaaccgagcuaCu.....................................................................  32  1  Fm3
....................Cacccuguagaaccgagcuagu.....................................................................  300  1  Fm3
....................uacccuguagaaccgagcuCgu.....................................................................  63  1  Fm3
....................uaUccuguagaaccgagcuagu.....................................................................  266  1  Fm3
....................uaccGuguagaaccgagcuagu.....................................................................  67  1  Fm3
....................uacccuUuagaaccgagcuagu.....................................................................  167  1  Fm3
....................uacccuNuagaaccgagcuagu.....................................................................  66  1  Fm3
....................uacccAguagaaccgagcuagu.....................................................................  74  1  Fm3
....................uacGcuguagaaccgagcuagu.....................................................................  137  1  Fm3
....................uacccuguagaaccgagNuagu.....................................................................  1  1  Fm3
....................uacccuguagaaccgagAuagu.....................................................................  12644  1  Fm3
....................uacccuguagaaccgGgcuagu.....................................................................  178  1  Fm3
....................uacccuguagaUccgagcuagu.....................................................................  716  1  Fm3
....................uacccuguagaacUgagcuagu.....................................................................  704  1  Fm3
....................uacccuguagaaccgagcuagC.....................................................................  118  1  Fm3
....................Nacccuguagaaccgagcuagu.....................................................................  696  1  Fm3
....................uacccuguagaaccgagcuaAu.....................................................................  108  1  Fm3
....................uacUcuguagaaccgagcuagu.....................................................................  285  1  Fm3
....................uacccuguagUaccgagcuagu.....................................................................  1282  1  Fm3
....................uacccuguagaaccgaUcuagu.....................................................................  2401  1  Fm3
....................Aacccuguagaaccgagcuagu.....................................................................  107  1  Fm3
....................uacccuguagaaccgaCcuagu.....................................................................  2889  1  Fm3
....................uacccuguagaaccgagcuagA.....................................................................  4139  1  Fm3
....................uaGccuguagaaccgagcuagu.....................................................................  373  1  Fm3
....................uacccuguagaaccgagcuagG.....................................................................  54  1  Fm3
....................uacccuguagaaccgaAcuagu.....................................................................  2781  1  Fm3
....................uacccuguagCaccgagcuagu.....................................................................  1217  1  Fm3
....................uacccuAuagaaccgagcuagu.....................................................................  244  1  Fm3
```

```
                    Mature                           Star
aaauguggugaguuucuguguacccuguagaaccgagcuaguguaucgaguucgaaacucacaagccauucuacaguguacaaagaaacgucgcuucuuucaacaaugaga
....................uacccCguagaaccgagcuagu.....................................................................   174       1    Fm3
....................uacccugGagaaccgagcuagu.....................................................................   36        1    Fm3
....................uacccuguagaaccgagcuaUu.....................................................................   218       1    Fm3
....................uacccuguagaCccgagcuagu.....................................................................   35        1    Fm3
....................uacccuguagaaAcgagcuagu.....................................................................   46        1    Fm3
....................uacccuguagaacAgagcuagu.....................................................................   426       1    Fm3
....................uacccuguagaaccgagcCagu.....................................................................   178       1    Fm3
....................uacccuguagaaccgagGuagu.....................................................................   4445      1    Fm3
....................uacccuguagaGccgagcuagu.....................................................................   152       1    Fm3
....................uacccNguagaaccgagcuagu.....................................................................   209       1    Fm3
....................uacccugCagaaccgagcuagu.....................................................................   254       1    Fm3
....................uacccGguagaaccgagcuagu.....................................................................   103       1    Fm3
....................Gacccuguagaaccgagcuagu.....................................................................   33        1    Fm3
....................uacccuguagaaccgCgcuagu.....................................................................   70        1    Fm3
....................uacccuguagaaccgagcuUgu.....................................................................   482       1    Fm3
....................uacccuguUgaaccgagcuagu.....................................................................   122       1    Fm3
....................uacccuguagaaccAagcuagu.....................................................................   363       1    Fm3
....................uaccAuguagaaccgagcuagu.....................................................................   730       1    Fm3
....................uacccuguagaaUcgagcuagu.....................................................................   655       1    Fm3
....................uacccuguagaaccgagcuagu.....................................................................   1313777   0    Fm3
....................uacccuguagaaccCagcuagu.....................................................................   80        1    Fm3
....................uacccugAagaaccgagcuagu.....................................................................   93        1    Fm3
....................uaAccuguagaaccgagcuagu.....................................................................   289       1    Fm3
....................uacccuguagaaccgagUuagu.....................................................................   6674      1    Fm3
....................uUcccuguagaaccgagcuagu.....................................................................   239       1    Fm3
....................uacccuguagaaccgagcAagu.....................................................................   47        1    Fm3
....................uacAcuguagaaccgagcuagu.....................................................................   717       1    Fm3
....................uacccuguagaacGgagcuagu.....................................................................   63        1    Fm3
....................uCcccuguagaaccgagcuagu.....................................................................   71        1    Fm3
....................uacccuguGgaaccgagcuagu.....................................................................   209       1    Fm3
....................Gacccuguagaaccgagcuagug....................................................................   1         1    Fm3
....................uacGcuguagaaccgagcuagug....................................................................   7         1    Fm3
....................uacccGguagaaccgagcuagug....................................................................   14        1    Fm3
....................uNcccuguagaaccgagcuagug....................................................................   1         1    Fm3
....................uacccuguaCaaccgagcuagug....................................................................   4         1    Fm3
....................uacccuguagaaccUagcuagug....................................................................   29        1    Fm3
....................uacccuguagaacGgagcuagug....................................................................   9         1    Fm3
....................uacccugGagaaccgagcuagug....................................................................   1         1    Fm3
....................uacUcuguagaaccgagcuagug....................................................................   27        1    Fm3
....................uacccuguagaaccgGgcuagug....................................................................   7         1    Fm3
....................uaAccuguagaaccgagcuagug....................................................................   28        1    Fm3
....................uacccAguagaaccgagcuagug....................................................................   5         1    Fm3
....................uacccuguagaCccgagcuagug....................................................................   3         1    Fm3
....................uacccuguagaaccgagcuaCug....................................................................   2         1    Fm3
....................uaccGuguagaaccgagcuagug....................................................................   11        1    Fm3
....................uacccuguagaaccgagcAagug....................................................................   7         1    Fm3
....................uacccuguagaaccgagcuCgug....................................................................   2         1    Fm3
....................uacccugAagaaccgagcuagug....................................................................   6         1    Fm3
....................uacccuguaUaaccgagcuagug....................................................................   19        1    Fm3
....................uacccuguagUaccgagcuagug....................................................................   112       1    Fm3
....................uacccuguagaaccgagcuGgug....................................................................   130       1    Fm3
....................uacccuguagaaccgagcuaguU....................................................................   20704     1    Fm3
....................uUcccuguagaaccgagcuagug....................................................................   22        1    Fm3
....................uacccuguagaaccgagcuaUug....................................................................   8         1    Fm3
....................uacccuguagaaGcgagcuagug....................................................................   6         1    Fm3
....................uacccuguagaaccgagcuagCg....................................................................   21        1    Fm3
....................uacccuguagaaccgagAuagug....................................................................   1110      1    Fm3
....................uacccuguagaaccAagcuagug....................................................................   32        1    Fm3
....................uacccuguagaaccCagcuagug....................................................................   7         1    Fm3
....................uaUccuguagaaccgagcuagug....................................................................   23        1    Fm3
....................uacccuguagGaccgagcuagug....................................................................   134       1    Fm3
....................uacccuguagaaccgagGuagug....................................................................   393       1    Fm3
....................uacccuguagaaccgaUcuagug....................................................................   230       1    Fm3
....................uacccuguagaaccgaAcuagug....................................................................   235       1    Fm3
....................uacccuguagCaccgagcuagug....................................................................   124       1    Fm3
....................Cacccuguagaaccgagcuagug....................................................................   20        1    Fm3
....................uacccuAuagaaccgagcuagug....................................................................   26        1    Fm3
....................uacccuUuagaaccgagcuagug....................................................................   13        1    Fm3
....................uacccuguagaaccgUgcuagug....................................................................   17        1    Fm3
....................uacccuguGgaaccgagcuagug....................................................................   25        1    Fm3
```

```
                    Mature                                  Star
aaauguggugaguuucugugguacccuguagaaccgagcuaguguaucgaguucgaaacucacaagccauucuacaguguacaaagaaacgucgcuucuuucaacaaugaga
....................uacccuguagaaccgagcuagAg....................................................................	18	1	Fm3
....................uacccuguagaGccgagcuagug....................................................................	16	1	Fm3
....................Nacccuguagaaccgagcuagug....................................................................	71	1	Fm3
....................uacccuguagaaccgagcuagGg....................................................................	1	1	Fm3
....................uacccuCuagaaccgagcuagug....................................................................	13	1	Fm3
....................uacccuguagaacAgagcuagug....................................................................	49	1	Fm3
....................uacccuguagaaccgagcuaguC....................................................................	47	1	Fm3
....................uacccuguagaaccgaCcuagug....................................................................	276	1	Fm3
....................uacccuguagaUccgagcuagug....................................................................	22	1	Fm3
....................uacccuguagaaccgagcuaguA....................................................................	7493	1	Fm3
....................uCccuguagaaccgagcuagug....................................................................	15	1	Fm3
....................uaccAuguagaaccgagcuagug....................................................................	56	1	Fm3
....................uacAcuguagaaccgagcuagug....................................................................	64	1	Fm3
....................uacccugCagaaccgagcuagug....................................................................	19	1	Fm3
....................uacccuguagaaUcgagcuagug....................................................................	24	1	Fm3
....................uacccNguagaaccgagcuagug....................................................................	18	1	Fm3
....................uacccuguagaacUgagcuagug....................................................................	31	1	Fm3
....................uacccuguagaaccgCgcuagug....................................................................	2	1	Fm3
....................uacccCguagaaccgagcuagug....................................................................	18	1	Fm3
....................uacccuguagaaccgagUuagug....................................................................	634	1	Fm3
....................uGcccuguagaaccgagcuagug....................................................................	24	1	Fm3
....................uacccuguagaaccgagcuaAug....................................................................	3	1	Fm3
....................uaGccuguagaaccgagcuagug....................................................................	31	1	Fm3
....................uacccuguUgaaccgagcuagug....................................................................	19	1	Fm3
....................uacccuguagaaccgagcCagug....................................................................	19	1	Fm3
....................uacccuguaAaaccgagcuagug....................................................................	6	1	Fm3
....................uaNccuguagaaccgagcuagug....................................................................	2	1	Fm3
....................uacccuNuagaaccgagcuagug....................................................................	5	1	Fm3
....................uacccuguagaaAcgagcuagug....................................................................	7	1	Fm3
....................uacccuguagaaccgagcuagug....................................................................	116234	0	Fm3
....................uaccUuguagaaccgagcuagug....................................................................	19	1	Fm3
....................uacccuguagaaccgagcuUgug....................................................................	21	1	Fm3
....................Aacccuguagaaccgagcuagug....................................................................	11	1	Fm3
....................uacccuguagaaccgagcCagugu...................................................................	9	1	Fm3
....................Nacccuguagaaccgagcuagugu...................................................................	61	1	Fm3
....................uaUccuguagaaccgagcuagugu...................................................................	35	1	Fm3
....................uacccuguagaaUcgagcuagugu...................................................................	32	1	Fm3
....................uaccUuguagaaccgagcuagugu...................................................................	13	1	Fm3
....................uacccuCuagaaccgagcuagugu...................................................................	17	1	Fm3
....................uaAccuguagaaccgagcuagugu...................................................................	28	1	Fm3
....................uacccuguagaaccgagcuagugC...................................................................	29	1	Fm3
....................uacccugCagaaccgagcuagugu...................................................................	26	1	Fm3
....................uacccNguagaaccgagcuagugu...................................................................	14	1	Fm3
....................uacccuguagaaccgagcGagugu...................................................................	41	1	Fm3
....................uacccuguagaaccgagcuaguUu...................................................................	1315	1	Fm3
....................uacUcuguagaaccgagcuagugu...................................................................	26	1	Fm3
....................uaccAuguagaaccgagcuagugu...................................................................	56	1	Fm3
....................Aacccuguagaaccgagcuagugu...................................................................	13	1	Fm3
....................uacccuguagaaccgagcuUgugu...................................................................	13	1	Fm3
....................uacccuguagGaccgagcuagugu...................................................................	157	1	Fm3
....................uacccuguagaaccgagcAagugu...................................................................	5	1	Fm3
....................uacccuguagaaccgagcuagAgu...................................................................	8	1	Fm3
....................uacGcuguagaaccgagcuagugu...................................................................	11	1	Fm3
....................uGcccuguagaaccgagcuagugu...................................................................	36	1	Fm3
....................uaNccuguagaaccgagcuagugu...................................................................	1	1	Fm3
....................uacccCguagaaccgagcuagugu...................................................................	20	1	Fm3
....................uacccuguagaaGcgagcuagugu...................................................................	4	1	Fm3
....................uacccuguagUaccgagcuagugu...................................................................	93	1	Fm3
....................uacccAguagaaccgagcuagugu...................................................................	2	1	Fm3
....................uacccuNuagaaccgagcuagugu...................................................................	6	1	Fm3
....................uacccuguagaaccgGgcuagugu...................................................................	14	1	Fm3
....................uacccuguaUaaccgagcuagugu...................................................................	14	1	Fm3
....................uacccuguagaaccgagAuagugu...................................................................	1104	1	Fm3
....................uacccuguagaaccgagcuaUugu...................................................................	7	1	Fm3
....................Cacccuguagaaccgagcuagugu...................................................................	22	1	Fm3
....................uacAcuguagaaccgagcuagugu...................................................................	83	1	Fm3
....................uacccugGagaaccgagcuagugu...................................................................	5	1	Fm3
....................uacccuguagaaccCagcuagugu...................................................................	6	1	Fm3
....................uCcccuguagaaccgagcuagugu...................................................................	7	1	Fm3
....................uacccuguagaaccgagcuagugA...................................................................	6426	1	Fm3
```

```
                    Mature                                    Star
aaauguggugaguuucugugguacccuguagaaccgagcuaguguaucgaguucgaaacucacaagccauucuacaguguacaaagaaacgucgcuucuuucaacaaugaga
....................uacccuguagaaccAagcuagugu...................................................................  35      1  Fm3
....................uacccuguagaaccgagcuagGgu...................................................................  5       1  Fm3
....................uacccuguagaaAcgagcuagugu...................................................................  5       1  Fm3
....................uacccuguagaGccgagcuagugu...................................................................  14      1  Fm3
....................uacccuguagaaccgagGuagugu...................................................................  402     1  Fm3
....................uUcccuguagaaccgagcuagugu...................................................................  26      1  Fm3
....................uacccuguagaaccgaAcuagugu...................................................................  235     1  Fm3
....................uacccuguUgaaccgagcuagugu...................................................................  25      1  Fm3
....................uacccuguagaaccgagcuaAugu...................................................................  6       1  Fm3
....................uacccuguagaaccgUgcuagugu...................................................................  15      1  Fm3
....................uacccuguagaaccgaUcuagugu...................................................................  203     1  Fm3
....................uacccuguaCaaccgagcuagugu...................................................................  2       1  Fm3
....................uacccuguGgaaccgagcuagugu...................................................................  28      1  Fm3
....................uacccuguagaaccUagcuagugu...................................................................  29      1  Fm3
....................uacccuguagaacAgagcuagugu...................................................................  48      1  Fm3
....................uaccGuguagaaccgagcuagugu...................................................................  7       1  Fm3
....................uacccuguagaaccgagcuagugG...................................................................  42      1  Fm3
....................uaGccuguagaaccgagcuagugu...................................................................  48      1  Fm3
....................uacccuguagaaccgaCcuagugu...................................................................  219     1  Fm3
....................uacccuguCgaaccgagcuagugu...................................................................  3       1  Fm3
....................uacccuguagaaccgagcuCgugu...................................................................  4       1  Fm3
....................uacccuguagaaccgagcuaguCu...................................................................  14      1  Fm3
....................uNcccuguagaaccgagcuagugu...................................................................  2       1  Fm3
....................uacccuAuagaaccgagcuagugu...................................................................  18      1  Fm3
....................uacccuguagCaccgagcuagugu...................................................................  107     1  Fm3
....................uacccuguagaaccgagcuagugu...................................................................  117437  0  Fm3
....................uacccuguagaUccgagcuagugu...................................................................  15      1  Fm3
....................uacccuguagaacUgagcuagugu...................................................................  58      1  Fm3
....................uacccuguaAaaccgagcuagugu...................................................................  5       1  Fm3
....................uacccuguagaaccgagcuGgugu...................................................................  113     1  Fm3
....................uacccGguagaaccgagcuagugu...................................................................  8       1  Fm3
....................uacccuUuagaaccgagcuagugu...................................................................  12      1  Fm3
....................uacccuguagaaccgagcuaCugu...................................................................  1       1  Fm3
....................uacccuguagaaccgagcuaguAu...................................................................  1714    1  Fm3
....................uacccuguagaaccgagcuagCgu...................................................................  11      1  Fm3
....................uacccuguagaaccgagUuagugu...................................................................  626     1  Fm3
....................uacccuguagaacGgagcuagugu...................................................................  7       1  Fm3
....................uacccugAagaaccgagcuagugu...................................................................  2       1  Fm3
....................uacccuguagaCccgagcuagugu...................................................................  5       1  Fm3
....................Gacccuguagaaccgagcuagugu...................................................................  3       1  Fm3
....................uacccuguagaaccgagcuagugCa..................................................................  1       1  Fm3
....................uacccuguagaaccgagcuaguguU..................................................................  798     1  Fm3
....................uacccuguagaaccgagcuaguguG..................................................................  4       1  Fm3
....................uacccuguagaaccgagcuagugua..................................................................  37      0  Fm3
....................uacccuguagaaccgagGuagugua..................................................................  1       1  Fm3
....................uacccuguagaaccgagcuagugAa..................................................................  37      1  Fm3
....................uacccuguagaaccgagcuaguUua..................................................................  2       1  Fm3
....................uacccuguagaaccgagcuaguguC..................................................................  3       1  Fm3
....................uacccuguagaaccgagcuagugAau.................................................................  1       1  Fm3
....................uacccuguagaaccgagcuaguguau.................................................................  46      0  Fm3
....................uacccuguagaaccgagcuaguguUu.................................................................  56      1  Fm3
....................uacccuguagaaccgagcuaguguaA.................................................................  1       1  Fm3
....................uacccuguagGaccgagcuaguguauc................................................................  1       1  Fm3
....................uacccuguagaaccgagcuaguguauc................................................................  27      0  Fm3
....................uacccuguagaacAgagcuaguguauc................................................................  1       1  Fm3
....................uacccuguagaaccgagcuaguguaucg...............................................................  36      0  Fm3
....................uacccuguagaaccgagcuaguguaucU...............................................................  3       1  Fm3
....................uacccuguagaaccgagcuaguguauAg...............................................................  1       1  Fm3
....................uacccuguagaaccgagcuaguguaucga..............................................................  4       0  Fm3
....................uacccuguagaaccgagcuaguguaucgag.............................................................  1       0  Fm3
....................uacccuguagaaccgagcuaguguaucgagu............................................................  3       0  Fm3
....................uacccuguagaaccgagcuaguguaucgaguu...........................................................  2       0  Fm3
.....................acccuguagaaccgagcA........................................................................  1       1  Fm3
.....................acccuguagaaccgagcu........................................................................  27      0  Fm3
.....................acccuguagaaccgagcua.......................................................................  13      0  Fm3
.....................acccuguagaaccgagcuU.......................................................................  1       1  Fm3
.....................acccuguagaaAcgagcua.......................................................................  1       1  Fm3
.....................acccuguaCaaccgagcuag......................................................................  1       1  Fm3
.....................acccuguagaaccgagcAag......................................................................  3       1  Fm3
.....................acccuguagaaccgaCcuag......................................................................  1       1  Fm3
```

```
                    Mature                Star
aaauguggugaguuucugugacccuguagaaccgagcuaguguaucgaguucgaaacucacaagccauucuacaguguacaaagaaacgucgcuucuuucaacaaugaga
.....................acccuguagaaccgagcCag......................................................................   7     1   Fm3
.....................acccuguagCaccgagcuag......................................................................   1     1   Fm3
.....................acccuguagaaccgagcuaC......................................................................   1     1   Fm3
.....................acccuguaUaaccgagcuag......................................................................   1     1   Fm3
.....................acccuguagaaccUagcuag......................................................................   1     1   Fm3
.....................acccuguagaaUcgagcuag......................................................................   1     1   Fm3
.....................acccuguagaacUgagcuag......................................................................   2     1   Fm3
.....................acccuguagaaccgagcuaA......................................................................   3     1   Fm3
.....................acccuguagaaccgagAuag......................................................................   1     1   Fm3
.....................acccuguagaGccgagcuag......................................................................   2     1   Fm3
.....................acccuguagaaccgagcuaU......................................................................   7     1   Fm3
.....................acGcuguagaaccgagcuag......................................................................   1     1   Fm3
.....................acccuguagaaccgagcuag......................................................................   363   0   Fm3
.....................acccuUuagaaccgagcuagu.....................................................................   6     1   Fm3
.....................acccuguagCaccgagcuagu.....................................................................   7     1   Fm3
.....................acccuguagUaccgagcuagu.....................................................................   2     1   Fm3
.....................acccuguagaaUcgagcuagu.....................................................................   7     1   Fm3
.....................acccuguagaaccgagcAagu.....................................................................   20    1   Fm3
.....................acccuguagaacAgagcuagu.....................................................................   4     1   Fm3
.....................acccuNuagaaccgagcuagu.....................................................................   1     1   Fm3
.....................acccuAuagaaccgagcuagu.....................................................................   4     1   Fm3
.....................acccuguagaaccgagcuaAu.....................................................................   8     1   Fm3
.....................acccuguagaaccgagcuCgu.....................................................................   8     1   Fm3
.....................acccuguagaaccgaUcuagu.....................................................................   2     1   Fm3
.....................acccuguagaaccUagcuagu.....................................................................   6     1   Fm3
.....................acccuguagaaccgCgcuagu.....................................................................   8     1   Fm3
.....................acccugCagaaccgagcuagu.....................................................................   7     1   Fm3
.....................Ncccuguagaaccgagcuagu.....................................................................   5     1   Fm3
.....................acccuguagaaccgUgcuagu.....................................................................   5     1   Fm3
.....................acccuguagaUccgagcuagu.....................................................................   18    1   Fm3
.....................acccuguaCaaccgagcuagu.....................................................................   6     1   Fm3
.....................acccuguagaaccgaAcuagu.....................................................................   3     1   Fm3
.....................acccuguagaaccgagcuagu.....................................................................   7974  0   Fm3
.....................accGuguagaaccgagcuagu.....................................................................   2     1   Fm3
.....................acccuguagaGccgagcuagu.....................................................................   28    1   Fm3
.....................acccugAagaaccgagcuagu.....................................................................   4     1   Fm3
.....................acccuguagaaccgagUuagu.....................................................................   7     1   Fm3
.....................acccuguagaaccgagcuagC.....................................................................   1     1   Fm3
.....................Ucccuguagaaccgagcuagu.....................................................................   6     1   Fm3
.....................acccuguagaaccgGgcuagu.....................................................................   5     1   Fm3
.....................acccuguGgaaccgagcuagu.....................................................................   1     1   Fm3
.....................acccCguagaaccgagcuagu.....................................................................   3     1   Fm3
.....................acGcuguagaaccgagcuagu.....................................................................   2     1   Fm3
.....................acccuguagaaccgagAuagu.....................................................................   13    1   Fm3
.....................acccuguaUaaccgagcuagu.....................................................................   8     1   Fm3
.....................acccGguagaaccgagcuagu.....................................................................   3     1   Fm3
.....................accUuguagaaccgagcuagu.....................................................................   3     1   Fm3
.....................acccuguagaacUgagcuagu.....................................................................   6     1   Fm3
.....................acccugGagaaccgagcuagu.....................................................................   4     1   Fm3
.....................acccuguagaaccAagcuagu.....................................................................   6     1   Fm3
.....................acccuguagaaAcgagcuagu.....................................................................   3     1   Fm3
.....................acccuguagaaccgagcuagA.....................................................................   20    1   Fm3
.....................acccuguaAaaccgagcuagu.....................................................................   5     1   Fm3
.....................acccuguagaaGcgagcuagu.....................................................................   2     1   Fm3
.....................aUccuguagaaccgagcuagu.....................................................................   1     1   Fm3
.....................acAcuguagaaccgagcuagu.....................................................................   3     1   Fm3
.....................acccuguagaaccCagcuagu.....................................................................   1     1   Fm3
.....................acccuguUgaaccgagcuagu.....................................................................   2     1   Fm3
.....................acccuCuagaaccgagcuagu.....................................................................   2     1   Fm3
.....................accAuguagaaccgagcuagu.....................................................................   10    1   Fm3
.....................acccuguagaaccgagcuUgu.....................................................................   12    1   Fm3
.....................acccuguagaaccgagGuagu.....................................................................   2     1   Fm3
.....................acccuguagaaccgagcCagu.....................................................................   47    1   Fm3
.....................acccuguagaaccgagcuGgu.....................................................................   14    1   Fm3
.....................acUcuguagaaccgagcuagu.....................................................................   3     1   Fm3
.....................acccuguagGaccgagcuagu.....................................................................   2     1   Fm3
.....................acccuguagaaccgagcuaUu.....................................................................   9     1   Fm3
.....................acccuguagaaccgagcuaCu.....................................................................   7     1   Fm3
.....................acccuguagaaccgaCcuagu.....................................................................   4     1   Fm3
.....................acccuguagaCccgagcuagu.....................................................................   17    1   Fm3
```

```
                    Mature                                      Star
aaauguggugaguuucugugu acccuguagaaccgagcuaguguaucgaguucgaaacucacaagccauucuacaguguacaaagaaacgucgcuucuuucaacaaugaga
.....................aAccuguagaaccgagcuagu.....................................................................  6     1  Fm3
.....................acccAguagaaccgagcuagu.....................................................................  2     1  Fm3
.....................acccuguaCaaccgagcuagug....................................................................  1     1  Fm3
.....................acccuguagaaccgaUcuagug....................................................................  2     1  Fm3
.....................acccuguagaaccgagcCagug....................................................................  6     1  Fm3
.....................acccuguagaaccgagcAagug....................................................................  8     1  Fm3
.....................Ucccuguagaaccgagcuagug....................................................................  2     1  Fm3
.....................Ncccuguagaaccgagcuagug....................................................................  2     1  Fm3
.....................acccuguagGaccgagcuagug....................................................................  1     1  Fm3
.....................acccuguagaaccgagcuaguA....................................................................  59    1  Fm3
.....................acccuguagaaAcgagcuagug....................................................................  1     1  Fm3
.....................acccuguagaaccgagUuagug....................................................................  1     1  Fm3
.....................acccuguagaaccgagcuCgug....................................................................  2     1  Fm3
.....................acccuguagaGccgagcuagug....................................................................  2     1  Fm3
.....................acccuguagaaccAagcuagug....................................................................  1     1  Fm3
.....................acccuguagaaccgagcuUgug....................................................................  3     1  Fm3
.....................acccugGagaaccgagcuagug....................................................................  1     1  Fm3
.....................acccuguagaaccgagAuagug....................................................................  3     1  Fm3
.....................acccugCagaaccgagcuagug....................................................................  4     1  Fm3
.....................acccuguagUaccgagcuagug....................................................................  1     1  Fm3
.....................acccuguagaaccgagcuaguU....................................................................  114   1  Fm3
.....................acccuguaAaaccgagcuagug....................................................................  1     1  Fm3
.....................acccuguagaaccgagcuagug....................................................................  1187  0  Fm3
.....................acccuguagaaccgUgcuagug....................................................................  1     1  Fm3
.....................accAuguagaaccgagcuagug....................................................................  4     1  Fm3
.....................acccuguagaaccgagAuagugu...................................................................  7     1  Fm3
.....................acccuguagaaccgagcuaguUu...................................................................  10    1  Fm3
.....................acccuguagUaccgagcuagugu...................................................................  1     1  Fm3
.....................acccuguagaaccgagcAagugu...................................................................  3     1  Fm3
.....................acccuguagaaccgagcGagugu...................................................................  11    1  Fm3
.....................acccuguagaaccgaAcuagugu...................................................................  1     1  Fm3
.....................acccuguagaUccgagcuagugu...................................................................  2     1  Fm3
.....................acccuguagaaccgagcuagugC...................................................................  4     1  Fm3
.....................acccuguagaacUgagcuagugu...................................................................  1     1  Fm3
.....................acccCguagaaccgagcuagugu...................................................................  1     1  Fm3
.....................acccuguagaCccgagcuagugu...................................................................  2     1  Fm3
.....................accAuguagaaccgagcuagugu...................................................................  2     1  Fm3
.....................acccuguagaaccgagcuUgugu...................................................................  2     1  Fm3
.....................acccuguagaaccgagcuCgugu...................................................................  2     1  Fm3
.....................acccuguagaaccgCgcuagugu...................................................................  1     1  Fm3
.....................acccuguagaaccgagcuaguAu...................................................................  12    1  Fm3
.....................acccuguagaaccgagcCagugu...................................................................  4     1  Fm3
.....................acccuguagaaccgagcuGgugu...................................................................  1     1  Fm3
.....................acccuguagaaccgagcuaUugu...................................................................  1     1  Fm3
.....................acccuguagaaccgagcuagugA...................................................................  128   1  Fm3
.....................acccuguagGaccgagcuagugu...................................................................  1     1  Fm3
.....................acccuguagaaccUagcuagugu...................................................................  2     1  Fm3
.....................acccuguagaaccgagcuagugG...................................................................  1     1  Fm3
.....................acccuguagaaUcgagcuagugu...................................................................  1     1  Fm3
.....................acccuguagaGccgagcuagugu...................................................................  1     1  Fm3
.....................acccuNuagaaccgagcuagugu...................................................................  2     1  Fm3
.....................acccuguagaaccgagUuagugu...................................................................  4     1  Fm3
.....................acccuguagaaccAagcuagugu...................................................................  2     1  Fm3
.....................acccuguagaaccgagcuaguCu...................................................................  1     1  Fm3
.....................Gcccuguagaaccgagcuagugu...................................................................  1     1  Fm3
.....................acccuguagaaccgaCcuagugu...................................................................  1     1  Fm3
.....................acAcuguagaaccgagcuagugu...................................................................  2     1  Fm3
.....................acccuAuagaaccgagcuagugu...................................................................  1     1  Fm3
.....................acccuguagaaccgagcuaAugu...................................................................  1     1  Fm3
.....................acccuguagaaccgagcuagugu...................................................................  1661  0  Fm3
.....................acccuUuagaaccgagcuagugu...................................................................  1     1  Fm3
.....................acccuguagaaccgagcuagugAa..................................................................  1     1  Fm3
.....................acccuguagaaccgagcuaguguU..................................................................  20    1  Fm3
.....................acccuguagaaccgagcuaguguUu.................................................................  2     1  Fm3
.....................acccuguagaaccgagcuaguguau.................................................................  4     0  Fm3
.....................acccuguagaaccgagcuaguguauc................................................................  3     0  Fm3
.....................acccuguagaaccgagcuaguguaucg...............................................................  6     0  Fm3
.....................acccuguagaaccgagcuaguguaGcgag.............................................................  1     1  Fm3
.....................acccuguagaaccgagcuaguguaucgag.............................................................  8     0  Fm3
.....................acccuguagaaccgagcuaguguaucgagu............................................................  9     0  Fm3
```

Mature **Star**

aaauguggugaguuucugug**uacccuguagaaccgagcuagu**guaucgaguucgaaacuca**caagccauucuacaguguaca**aagaaacgucgcuucuuucaacaaugaga			
.....................acccuguagaacGgagcuaguguaucgagu..	1	1	Fm3
......................cccuguagaaccgagcuaU..	2	1	Fm3
......................cccuguagaaccgagcuag..	9	0	Fm3
......................cccuguagaaccgGgcuagu...	2	1	Fm3
......................cccuguagaaccgagcuGgu...	3	1	Fm3
......................Gccuguagaaccgagcuagu...	1	1	Fm3
......................ccAuguagaaccgagcuagu...	1	1	Fm3
......................cccuguagaaccgagcAagu...	1	1	Fm3
......................cccuguagaaccgaAcuagu...	1	1	Fm3
......................cccuguagaaUcgagcuagu...	1	1	Fm3
......................cccuguagaCccgagcuagu...	1	1	Fm3
......................cccuguagaaccgagcuagu...	225	0	Fm3
......................Uccuguagaaccgagcuagu...	1	1	Fm3
......................cccuguagaaccgagcuaUu...	4	1	Fm3
......................cccuguagaaccgaCcuagu...	5	1	Fm3
......................cccuguagaaccgagcuagug..	18	0	Fm3
......................cccuUuagaaccgagcuagug..	1	1	Fm3
......................cccuguagaaccgagcuaguA..	1	1	Fm3
......................cccuguagaaccgagcuaguU..	7	1	Fm3
......................cccuguaAaaccgagcuagug..	1	1	Fm3
......................cccuguagaaccgagcuaguUu...	2	1	Fm3
......................cccuguagaaccgagcuagugu...	29	0	Fm3
......................cccuguagaaccgagcuagugA...	2	1	Fm3
......................cccCguagaaccgagcuagugu...	1	1	Fm3
......................cccuguagaaccgagcuaguAu...	1	1	Fm3
......................cccuguagaaccgagcuaguguau...	1	0	Fm3
.......................ccuguagaaccgagcuag..	2	0	Fm3
.......................ccuguagaaccgagcuagu...	35	0	Fm3
.......................ccuguagaaccAagcuagu...	1	1	Fm3
.......................ccuguagaaccgagcuagug..	4	0	Fm3
.......................ccuguagaaccgagcuaguU..	2	1	Fm3
.......................ccuguagaaccgagcuagugu...	1	0	Fm3
........................cuguagaaccgagcuagu...	1	0	Fm3
....ucacaagccauucuacagugua...............................	4	0	Fm3
....ucacaagccauucuacaAugua...............................	1	1	Fm3
....Ccaagccauucuacaguguac..............................	1	1	Fm3
....caagccaCucuacagugu................................	1	1	Fm3
....caagccauucuacaguUu................................	1	1	Fm3
....Naagccauucuacagugu................................	1	1	Fm3
....Uaagccauucuacagugu................................	3	1	Fm3
....caagccauucuacaguCu................................	7	1	Fm3
....caagccauucuacaguAu................................	1	1	Fm3
....caagccauucuacaUugu................................	1	1	Fm3
....caagccUuucuacagugu................................	1	1	Fm3
....caagAcauucuacagugu................................	1	1	Fm3
....caagccauucuUcagugu................................	1	1	Fm3
....caCgccauucuacagugu................................	1	1	Fm3
....caagccauucuacagCgu................................	1	1	Fm3
....caagccauucuacagugu................................	1611	0	Fm3
....caagccauucuGcagugu................................	1	1	Fm3
....caagccauucGacagugu................................	5	1	Fm3
....caUgccauucuacagugu................................	1	1	Fm3
....caagccauucuacagugA................................	27	1	Fm3
....caagccauucuacagugG................................	6	1	Fm3
....caagccauucuacagugC................................	16	1	Fm3
....caagcNauucuacagugu................................	1	1	Fm3
....caagccauucAacagugu................................	2	1	Fm3
....caagccauucCacagugu................................	1	1	Fm3
....caagccauucuacaguguC...............................	1	1	Fm3
....caagccauucuacaguguU...............................	51	1	Fm3
....caagccauucuacaguUua...............................	1	1	Fm3
....cGagccauucuacagugua...............................	1	1	Fm3
....caagccauucCacagugua...............................	1	1	Fm3
....caagccauucuacaguCua...............................	1	1	Fm3
....caagccauucuacagugAa...............................	5	1	Fm3
....caagccauucuacagugCa...............................	1	1	Fm3
....Uaagccauucuacagugua...............................	1	1	Fm3
....caagccauucuacagugua...............................	212	0	Fm3
....caCgccauucuacaguguac..............................	3	1	Fm3
....caagccauucuacaAuguac..............................	2	1	Fm3

Mature   Star

```
aaauguggugaguuucugugguacccuguagaaccgagcuaguguaucgaguucgaaacucacaagccauucuacaguguacaaagaaacgucgcuucuuucaacaaugaga
.............................................................caagcNauucuacaguguac..............................	3	1	Fm3
.............................................................caagccauucAacaguguac..............................	14	1	Fm3
.............................................................caagccauucGacaguguac..............................	16	1	Fm3
.............................................................Aaagccauucuacaguguac..............................	3	1	Fm3
.............................................................caagccauucuacaguAuac..............................	28	1	Fm3
.............................................................caaAccauucuacaguguac..............................	2	1	Fm3
.............................................................caGgccauucuacaguguac..............................	4	1	Fm3
.............................................................caagccGuucuacaguguac..............................	4	1	Fm3
.............................................................caagccauucuacaguUuac..............................	9	1	Fm3
.............................................................caagccauucuacaguCuac..............................	28	1	Fm3
.............................................................caagccauuAuacaguguac..............................	1	1	Fm3
.............................................................caagccauucuGcaguguac..............................	3	1	Fm3
.............................................................caagccauucuacaguguac..............................	10517	0	Fm3
.............................................................caagccauucuacUguguac..............................	1	1	Fm3
.............................................................caagccauuUuacaguguac..............................	3	1	Fm3
.............................................................caaUccauucuacaguguac..............................	5	1	Fm3
.............................................................caagccauucCacaguguac..............................	13	1	Fm3
.............................................................caagccauucuacGguguac..............................	4	1	Fm3
.............................................................caagccauucuacagugCac..............................	78	1	Fm3
.............................................................Naagccauucuacaguguac..............................	5	1	Fm3
.............................................................caagUcauucuacaguguac..............................	2	1	Fm3
.............................................................caagccauucuacaguguGc..............................	1	1	Fm3
.............................................................caagccauucuaUaguguac..............................	5	1	Fm3
.............................................................caagccauucuacaguguaA..............................	16	1	Fm3
.............................................................caagccaCucuacaguguac..............................	3	1	Fm3
.............................................................caagccaGucuacaguguac..............................	1	1	Fm3
.............................................................caagccauucuUcaguguac..............................	1	1	Fm3
.............................................................Uaagccauucuacaguguac..............................	13	1	Fm3
.............................................................caagAcauucuacaguguac..............................	5	1	Fm3
.............................................................caagccauucuacaCuguac..............................	1	1	Fm3
.............................................................caagccauucuacagugAac..............................	59	1	Fm3
.............................................................caUgccauucuacaguguac..............................	3	1	Fm3
.............................................................cCagccauucuacaguguac..............................	2	1	Fm3
.............................................................caagccauucuacaguguCc..............................	1	1	Fm3
.............................................................caagccauAcuacaguguac..............................	1	1	Fm3
.............................................................caagcGauucuacaguguac..............................	1	1	Fm3
.............................................................cUagccauucuacaguguac..............................	2	1	Fm3
.............................................................caagccauucuacaguguaU..............................	21	1	Fm3
.............................................................caagccauucuacagugGac..............................	51	1	Fm3
.............................................................caagcUauucuacaguguac..............................	13	1	Fm3
.............................................................caagccauuGuacaguguac..............................	1	1	Fm3
.............................................................caagccUuucuacaguguac..............................	4	1	Fm3
.............................................................caagccauucuacaguguacU.............................	180	1	Fm3
.............................................................caagUcauucuacaguguaca.............................	3	1	Fm3
.............................................................caagccauucuacaguguaca.............................	57041	0	Fm3
.............................................................caagccauucuacagugGaca.............................	241	1	Fm3
.............................................................caagccauucuacaguAuaca.............................	143	1	Fm3
.............................................................caagccauucuGcaguguaca.............................	5	1	Fm3
.............................................................cGagccauucuacaguguaca.............................	11	1	Fm3
.............................................................caagccauucuacaUuguaca.............................	10	1	Fm3
.............................................................caagccauucuacaguguUca.............................	3	1	Fm3
.............................................................caagccauucGacaguguaca.............................	93	1	Fm3
.............................................................caagccauucuacagugAaca.............................	326	1	Fm3
.............................................................caagccauucuacaguUuaca.............................	51	1	Fm3
.............................................................caagccauucuacaguguaUa.............................	13	1	Fm3
.............................................................caagccauucuaUaguguaca.............................	9	1	Fm3
.............................................................caagccauucuacaCuguaca.............................	2	1	Fm3
.............................................................caagccGuucuacaguguaca.............................	9	1	Fm3
.............................................................caagccauucuacaguguaAa.............................	1	1	Fm3
.............................................................caUgccauucuacaguguaca.............................	11	1	Fm3
.............................................................caaUccauucuacaguguaca.............................	26	1	Fm3
.............................................................caaAccauucuacaguguaca.............................	7	1	Fm3
.............................................................caagccauucuacUguguaca.............................	3	1	Fm3
.............................................................caagccauCcuacaguguaca.............................	6	1	Fm3
.............................................................caCgccauucuacaguguaca.............................	12	1	Fm3
.............................................................Aaagccauucuacaguguaca.............................	15	1	Fm3
.............................................................cCagccauucuacaguguaca.............................	5	1	Fm3
.............................................................Uaagccauucuacaguguaca.............................	76	1	Fm3
.............................................................caagGcauucuacaguguaca.............................	5	1	Fm3
.............................................................caagccauGcuacaguguaca.............................	5	1	Fm3
```

Mature **Star**

```
aaauguggugaguuucugugguacccuguagaaccgagcuaguguaucgaguucgaaacucacaagccauucuacaguguacaaagaaacgucgcuucuuucaacaaugaga
.............................................................caagccUuucuacaguguaca.............................  16    1  Fm3
.............................................................caagcUauucuacaguguaca.............................  31    1  Fm3
.............................................................caagcAauucuacaguguaca.............................  12    1  Fm3
.............................................................caagccauucuacCguguaca.............................  1     1  Fm3
.............................................................caagccauucuacaAuguaca.............................  4     1  Fm3
.............................................................caagccauucuaGaguguaca.............................  5     1  Fm3
.............................................................caagccCuucuacaguguaca.............................  1     1  Fm3
.............................................................Naagccauucuacaguguaca.............................  18    1  Fm3
.............................................................caagccauucuacagCguaca.............................  7     1  Fm3
.............................................................caNgccauucuacaguguaca.............................  1     1  Fm3
.............................................................caagccauucuacagugCaca.............................  360   1  Fm3
.............................................................caagccauuAuacaguguaca.............................  4     1  Fm3
.............................................................caaCccauucuacaguguaca.............................  4     1  Fm3
.............................................................caagccaCucuacaguguaca.............................  5     1  Fm3
.............................................................caagcGauucuacaguguaca.............................  4     1  Fm3
.............................................................caagccauucCacaguguaca.............................  56    1  Fm3
.............................................................caagccauucuacaguguacG.............................  5     1  Fm3
.............................................................caagccauucuacagAguaca.............................  4     1  Fm3
.............................................................caagccauuGuacaguguaca.............................  1     1  Fm3
.............................................................caagccauucuacagGguaca.............................  1     1  Fm3
.............................................................caagccauucAacaguguaca.............................  101   1  Fm3
.............................................................caagccauucuacGguguaca.............................  9     1  Fm3
.............................................................caagccauAcuacaguguaca.............................  5     1  Fm3
.............................................................cUagccauucuacaguguaca.............................  10    1  Fm3
.............................................................caagccauucuaAaguguaca.............................  1     1  Fm3
.............................................................caagccaGucuacaguguaca.............................  2     1  Fm3
.............................................................caagccauucuacaguCuaca.............................  131   1  Fm3
.............................................................caagcNauucuacaguguaca.............................  11    1  Fm3
.............................................................caagAcauucuacaguguaca.............................  14    1  Fm3
.............................................................caagccauucuacaguguacC.............................  5     1  Fm3
.............................................................caGgccauucuacaguguaca.............................  18    1  Fm3
.............................................................caagccauucuUcaguguaca.............................  14    1  Fm3
.............................................................caagccauuUuacaguguaca.............................  20    1  Fm3
.............................................................caagccauucuacaguguGca.............................  7     1  Fm3
.............................................................Gaagccauucuacaguguaca.............................  3     1  Fm3
.............................................................caagccNuucuacaguguaca.............................  2     1  Fm3
.............................................................cUagccauucuacaguguacaa............................  2     1  Fm3
.............................................................caagccauucuacaguguaUaa............................  7     1  Fm3
.............................................................caagccauucuacagAguacaa............................  1     1  Fm3
.............................................................caagccauucuacaguguacaa............................  7658  0  Fm3
.............................................................caagccUuucuacaguguacaa............................  2     1  Fm3
.............................................................caagccauCcuacaguguacaa............................  2     1  Fm3
.............................................................Naagccauucuacaguguacaa............................  3     1  Fm3
.............................................................caaUccauucuacaguguacaa............................  2     1  Fm3
.............................................................caagccauucuacaUuguacaa............................  1     1  Fm3
.............................................................caagccauucuacaguCuacaa............................  20    1  Fm3
.............................................................caagccauucuacaguguacUa............................  3     1  Fm3
.............................................................caagccauucuacaguguacCa............................  9     1  Fm3
.............................................................caagccauGcuacaguguacaa............................  1     1  Fm3
.............................................................caagcAauucuacaguguacaa............................  1     1  Fm3
.............................................................caagccauucuacagCguacaa............................  1     1  Fm3
.............................................................caCgccauucuacaguguacaa............................  1     1  Fm3
.............................................................caagccauAcuacaguguacaa............................  1     1  Fm3
.............................................................Aaagccauucuacaguguacaa............................  2     1  Fm3
.............................................................caagccauucAacaguguacaa............................  16    1  Fm3
.............................................................Uaagccauucuacaguguacaa............................  2     1  Fm3
.............................................................caagcUauucuacaguguacaa............................  3     1  Fm3
.............................................................caagccauucuacUguguacaa............................  1     1  Fm3
.............................................................caagccauucuacaguguacaC............................  873   1  Fm3
.............................................................caagccauucuacaguUuacaa............................  5     1  Fm3
.............................................................caagccauucuacagugAacaa............................  32    1  Fm3
.............................................................caagccauucuacagugGacaa............................  26    1  Fm3
.............................................................caagccauucuGcaguguacaa............................  1     1  Fm3
.............................................................caagccauucuacaguguacGa............................  7     1  Fm3
.............................................................caagccauucuaUaguguacaa............................  1     1  Fm3
.............................................................caagccGuucuacaguguacaa............................  2     1  Fm3
.............................................................caagccauucuacGguguacaa............................  2     1  Fm3
.............................................................caagUcauucuacaguguacaa............................  2     1  Fm3
.............................................................caGgccauucuacaguguacaa............................  3     1  Fm3
.............................................................caagccauucuacaguguUcaa............................  1     1  Fm3
```

Mature Star

```
aaauguggugaguuucugugaaacccuguagaaccgagcuaguguaucgaguucgaaacucacaagccauucuacaguguacaaagaaacgucgcuucuuucaacaaugaga
```

```
aaauguggugaguuucuguguacccuguagaaccgagcuaguguaucgaguucgaaacucacaagccauucuacaguguacaaagaaacgucgcuucuuucaacaaugaga
.............................................................caagccauucuacaguguGcaa............................   1    1   Fm3
.............................................................caagccauucuacaguAuacaa............................   18   1   Fm3
.............................................................caagccauucGacaguguacaa............................   10   1   Fm3
.............................................................caagccauucuacaguguacaU............................   121  1   Fm3
.............................................................caagccauucCacaguguacaa............................   7    1   Fm3
.............................................................cCagccauucuacaguguacaa............................   1    1   Fm3
.............................................................caagccauucuacagugCacaa............................   50   1   Fm3
.............................................................caagccauucuaGaguguacaa............................   1    1   Fm3
.............................................................caagccauuGuacaguguacaa............................   1    1   Fm3
.............................................................cGagccauucuacaguguacaa............................   2    1   Fm3
.............................................................caagccauucuacagugGacaaa...........................   2    1   Fm3
.............................................................caagccauucuacagugCacaaa...........................   1    1   Fm3
.............................................................caagccauucuacagugAacaaa...........................   2    1   Fm3
.............................................................caagccauucuacaguguacaCa...........................   4    1   Fm3
.............................................................caagccauucuacaguguacaaG...........................   8    1   Fm3
.............................................................Aaagccauucuacaguguacaaa...........................   1    1   Fm3
.............................................................caagccauucuacaguguacaaC...........................   48   1   Fm3
.............................................................caagccauucCacaguguacaaa...........................   1    1   Fm3
.............................................................caagccauucuacaguguacaaa...........................   300  0   Fm3
.............................................................caagccauucuacaguguacaaU...........................   318  1   Fm3
.............................................................caagccauucGacaguguacaaa...........................   1    1   Fm3
.............................................................Uaagccauucuacaguguacaaa...........................   1    1   Fm3
.............................................................caagcUauucuacaguguacaaa...........................   1    1   Fm3
.............................................................caagccauucuacaguCuacaaa...........................   1    1   Fm3
.............................................................caagccauucuacaguguacaaaU..........................   2    1   Fm3
.............................................................caagccauucuacaguguacaaaA..........................   5    1   Fm3
..............................................................aagccauucuacagugua...............................   4    0   Fm3
..............................................................aCgccauucuacagugua...............................   1    1   Fm3
..............................................................Cagccauucuacaguguac..............................   1    1   Fm3
..............................................................aagccauucuacaguguGc..............................   1    1   Fm3
..............................................................aagccauucuacaguguac..............................   62   0   Fm3
..............................................................aagccauucuCcaguguaca.............................   1    1   Fm3
..............................................................aagccauucuacaguguGca.............................   2    1   Fm3
..............................................................aagccauucuacaguguaUa.............................   2    1   Fm3
..............................................................aagccauucuacagGguaca.............................   1    1   Fm3
..............................................................Cagccauucuacaguguaca.............................   2    1   Fm3
..............................................................aagccauucuacaCuguaca.............................   1    1   Fm3
..............................................................aagccauucuacagugGaca.............................   1    1   Fm3
..............................................................aagccauucuacaguguCca.............................   2    1   Fm3
..............................................................aagccauucuacaguguUca.............................   1    1   Fm3
..............................................................aagccauucuGcaguguaca.............................   3    1   Fm3
..............................................................aagccauAcuacaguguaca.............................   1    1   Fm3
..............................................................Uagccauucuacaguguaca.............................   1    1   Fm3
..............................................................aagccauucuacaguguaca.............................   395  0   Fm3
..............................................................aagccauucuacagugCacaa............................   1    1   Fm3
..............................................................aagccauucuacaguguacaa............................   86   0   Fm3
..............................................................Uagccauucuacaguguacaa............................   1    1   Fm3
..............................................................aagccauucuacaguguacaC............................   7    1   Fm3
..............................................................aagccauucuacaguguacaaU...........................   2    1   Fm3
...............................................................agccauucuacaguguaca.............................   5    0   Fm3
.................................................................ccauucuacaguguacaaaga.........................   8    0   Fm3
.................................................................ccauucuacaguguacaaaAa.........................   6    1   Fm3
.................................................................ccauucuacaguguacaaagaa........................   11   0   Fm3
```
